# Enantioselective Synthesis of 3‐Fluorochromanes via Iodine(I)/Iodine(III) Catalysis

**DOI:** 10.1002/anie.202005181

**Published:** 2020-06-09

**Authors:** Jérôme C. Sarie, Christian Thiehoff, Jessica Neufeld, Constantin G. Daniliuc, Ryan Gilmour

**Affiliations:** ^1^ Organisch Chemisches Institut Westfälische Wilhelms-Universität Münster Corrensstraße 40 48149 Münster Germany

**Keywords:** chromanes, fluorine, *gauche* effect, organocatalysis, stereospecificity

## Abstract

The chromane nucleus is common to a plenum of bioactive small molecules where it is frequently oxidized at position 3. Motivated by the importance of this position in conferring efficacy, and the prominence of bioisosterism in drug discovery, an iodine(I)/iodine(III) catalysis strategy to access enantioenriched 3‐fluorochromanes is disclosed (up to 7:93 *e.r*.). In situ generation of ArIF_2_ enables the direct fluorocyclization of allyl phenyl ethers to generate novel scaffolds that manifest the stereoelectronic *gauche* effect. Mechanistic interrogation using deuterated probes confirms a stereospecific process consistent with a type II_inv_ pathway.

## Graphical abstract

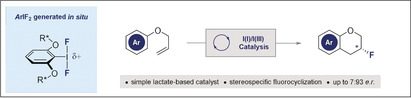

Molecular editing with fluorine is a powerful strategy to realize clinical efficacy whilst mitigating perceived metabolic or toxicological liabilities.^1^ The flexibility to invert localized partial charge (H^*δ*+^→F^*δ*−^),^2^ or delete single hydrogen bonds whilst preserving the electronic environment (OH→F)^3^ has a negligible steric penalty, thus rendering this approach expansive. Fluorine bioisosterism is particularly apposite in molecule classes that are pre‐disposed to oxidation at a specified site, but effective structure optimization is contingent on the synthesis arsenal.^4^ The venerable chromane nucleus inherent to a spectrum of bioactive drugs and natural products is an exemplar of this synergy. Despite the prevalence of the parent (H) and 3‐hydroxy scaffolds (OH), catalysis‐based strategies to access 3‐fluorochromanes remain sparse. Stoichiometric, racemic routes have been reported that rely on XeF_2_, bromofluorination, or additions to 3‐fluorobutenone,^5^ whilst catalytic, enantioselective routes have been reported to generate 3‐fluoro‐4‐spiro‐chromanes. Pertinent examples under the auspices of chiral phase transfer catalysis,^6^ and phosphoramidate catalyzed bromo‐ and iodocyclizations are noteworthy.^7^ To complement these elegant solutions, efficient entry to the parent 3‐fluorochromane scaffold is needed to complete the bioisosterism continuum [H≈F≈OH] (Figure 1, center)^8^ and explore the potential of the unsubstituted ring system in drug discovery. The *vicinal* relationship of fluorine to the ring oxygen will result in stabilizing hyperconjugative interactions (σ_C‐H_→σ_C‐F_*),^9^ that manifest themselves in the conformation of these drug modules. The broad spectrum of biological activities mediated by chromanes is a powerful motivator to address this deficiency in contemporary catalysis. Pertinent examples include the antidiabetic agent Englitazone (**1**),^10^ Xiamenmicin (**2**) which exhibits anti‐inflammatory properties,^11^ and the venerable antioxidant Tocopherol (vitamin E) (**3**).^12^ The chemotherapeutic potential of (+)‐Catechin (**4**)^13^ further adds to this clinical diversity (Figure 1, top). To reconcile the clinical importance of chromanes, and the potential of bioisosterism, with the value of catalysis‐based strategies to access enantioenriched 3‐fluoro scaffolds, a formal 6‐*endo‐trig* fluorocyclization^14^, ^15^ of simple allyl phenyl ethers via I^I^/I^III^ catalysis^16^ was envisaged (Figure 1, bottom). This conceptually simple entry point would likely accelerate investigation of the physicochemical profile inherent to this intriguing 3D drug module.^17^

**Figure 1:**
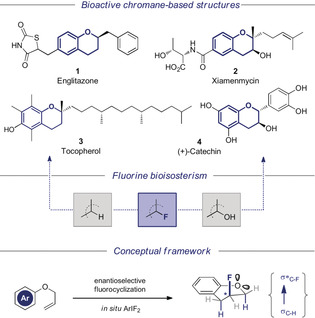
Bioactive chromanes, and a catalysis‐based strategy to enable the synthesis of fluorinated systems.

The oxidative functionalization of π‐bonds through hypervalent iodine‐based catalysis platforms has been intensively pursued.^18^ Contemporaneous reports by this laboratory^19^ and the Jacobsen laboratory^20^ have demonstrated that simple aryl iodide/ HF combinations efficiently support the difluorination of alkenes in the presence of a stoichiometric oxidant. Enabled by the in situ generation of hypervalent ArI^III^F_2_ intermediates,^21^ these transformations have also been translated to an enantioselective paradigm^22^, ^23^ using *C*_2_‐symmetric Ishihara–Muñiz scaffolds.^24^ Encouraged by the effectiveness of this platform the fluorocyclization of simple 1‐(allyloxy)‐4‐bromobenzene (**5**) was explored as described in Table 1. The bromo‐substrate was specifically chosen to enable downstream functionalization by cross coupling technologies, and mitigate catalyst sequestration.^25^ Reactions were performed in dichloromethane at ambient temperature using an initial amine:HF ratio of 1:5 (please see Table 2). Selectfluor^®^ was employed as a terminal oxidant and reactions were quenched after 24 h. A process of catalyst structural editing was performed on a generic *C*_2_‐symmetric iodoresorcinol derivative functionalized with methyl lactate groups (Table 1, entry 1). Although this catalyst scaffold enabled the fluorocyclization of **5** to **6**, the product was racemic and formation of the *vicinal* difluoride **6 a** was detected. To enhance catalyst performance, the terminal methyl esters were initially modified. Substituting the methyl esters (X=OMe to OBn) did not translate to an enhancement of selectivity (Table 1, entry 2). However, repeating this at position “R” proved to be encouraging, resulting in an enhanced *e.r*. from 56:44 to 24:76 (Table 1, entry 3).

**Table 1:** Catalyst optimization.^[a]^ 
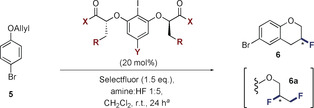

Entry	Y	R	X	Yield [%] (**6**:**6 a**)^[b]^	*e.r*. (**6**)
1	H	H	OMe	47 (91:9)	52:48
2	H	H	OBn	48 (89:11)	56:44
3	H	Ph	OMe	51 (93:7)	24:76
4^[c]^	H	Ph	OMe	56 (90:10)	38:62
5	**H**	**Ph**	**NHMe**	**72 (93:7)**	**13:87**
6^[c]^	H	Ph	NHMe	69 (93:7)	27:73
7	H	Ph	NH_2_	72 (93:7)	12:88
8	H	Ph	NMe_2_	71 (89:11)	34:66
9	H	Cy	NHMe	47 (90:10)	28:72
10	Me	Ph	NHMe	60 (92:8)	12:88
11	CO_2_Me	Ph	NHMe	60 (93:7)	12:88

[a] Standard reaction conditions: **5** (0.2 mmol), catalyst (20 mol %), Selectfluor^®^ (1.5 equiv), solvent (0.5 mL), amine:HF 1:5 (0.5 mL), ambient temperature, 24 h. [b] Determined by ^19^F NMR spectroscopy of the crude reaction mixture using ethyl fluoracetate as internal standard. [c] *C*_1_‐symmetric catalyst.

**Table 2:** Reaction optimization.^[a]^ 
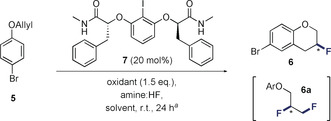

Entry	Oxidant	Amine:HF	Solvent	Yield [%] (**6**:**6 a**)^[b]^	*e.r*. (**6**)
1	Selectfluor^®^	1:4.5	CH_2_Cl_2_	52 (87:13)	12:88
**2**	**Selectfluor** ^®^	**1:5**	**CH_2_Cl_2_**	**72 (93:7)**	**13:87**
3	Selectfluor^®^	1:6	CH_2_Cl_2_	54 (95:5)	13:87
4	Selectfluor^®^	1:7.5	CH_2_Cl_2_	19 (100:0)	18:82
5	Selectfluor^®^	1:5	C_2_H_4_Cl_2_	64 (90:10)	13:87
6	Selectfluor^®^	1:5	CHCl_3_	66 (91:9)	14:86
7	Selectfluor^®^	1:5	toluene	52 (90:10)	15:85
8	Selectfluor^®^	1:5	CH_3_CN	22 (77:23)	11:89
9	*m‐*CPBA	1:5	CH_2_Cl_2_	55 (88:12)	29:71
10	gr. Oxone	1:5	CH_2_Cl_2_	16 (66:34)	13:87
11^[c]^	Selectfluor^®^	1:5	CH_2_Cl_2_	62 (92:8)	14:86
12^[d]^	Selectfluor^®^	1:5	CH_2_Cl_2_	5 (>95:5)	–:–
13^[e]^	Selectfluor^®^	1:5	CH_2_Cl_2_	<5 (–:–)	–:–

[a] Standard reaction conditions: **5** (0.2 mmol), catalyst **7** (20 mol %), Selectfluor^®^ (1.5 equiv), solvent (0.5 mL), amine:HF 1:5 (0.5 mL), ambient temperature, 24 h. [b] Combined yield for **6** and **6 a** determined by ^19^F NMR spectroscopy of the crude reaction mixture using ethyl fluoracetate as internal standard. [c] 10 mol % of **7**. [d] 0 °C. [e] Reaction in the absence of catalyst **7**.

As a control experiment to validate the importance of *C*_2_‐symmetry, the *C*_1_‐analog was evaluated which gave an *e.r*. of 38:62 (Table 1, entry 4). To explore the potential of intramolecular hydrogen bonds in pre‐organizing the intermediate I^III^ species, incorporation of secondary amine units was then explored.^26^ This led to a notable increase in yield from 51 % to 72 % and *e.r*. from 24:76 to 13:87 when using the methyl amide‐containing catalyst (Table 1, entries 3 and 5). Again, the *C*_1_‐symmetric control catalyst was prepared and, whilst this led to an improvement in yield and enantioselection compared to the ester (Table 1, entries 4 and 6), the requirement for *C*_2_‐symmetry is clearly apparent (Table 1, entries 5 and 6). The primary amide catalyst (Table 1, entry 7) displayed similar activity, whereas dimethylation (Table 1, entry 8) caused a notable drop in *e.r*. To explore the possible involvement of non‐covalent aromatic interactions in catalysis,^27^ the peripheral substituent R was replaced by a cyclohexyl motif (Table 1, entry 9). This proved to be highly detrimental to efficiency (47 % yield versus 72 %). Since the *para*‐position on the iodoarene provides a handle to regulate the I^I^/I^III^ oxidation,21a editing at this site was systematically investigated. Although the introduction of a 4‐methyl or methyl ester substituent had no discernible impact on enantioselectivity, a substantial erosion of the overall yield was observed (Table 1, entries 10 and 11).

The remainder of the study was conducted with catalyst **7** (X=NHMe, R=Ph, Y=H). It was possible to obtain crystals of the 4‐methyl‐substituted catalyst that were suitable for X‐ray structure analysis (Figure 2). Salient features of this analysis include the shielding influence of the pendant aryl rings above the iodine center, and the direction of the N−H bonds that would conceivably enable intramolecular interactions in the I^III^ species.

**Figure 2:**
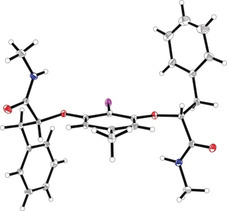
X‐ray analysis of the 4‐methyl‐substituted catalyst (X=NHMe, R=Ph, Y=Me). Deposition number 199518 contain(s) the supplementary crystallographic data for this paper. These data are provided free of charge by the joint Cambridge Crystallographic Data Centre and Fachinformationszentrum Karlsruhe Access Structures service.

In the context of our alkene difluorination studies, we established the importance of Brønsted acidity in regulating regio‐selectivity (*geminal* versus *vicinal*) in I^I^/I^III^ catalysis.19b, 22 Whereas lower amine:HF ratios (ca. 1:4.5) favored 1,2‐difluorination of terminal alkenes, Olah's reagent (ca. 1:9 pyridine:HF) induced engagement of the aryl ring leading to the 1,1‐product. In an effort to suppress competing 1,2‐difluorination and facilitate cyclization, the effect of the amine:HF ratio, as a mixture of triethylamine trihydrofluoride and Olah′s reagent, was investigated (Table 2).

As indicated in entries 1—4 in Table 2, a ratio of 1:5 proved optimal (*e.r*. 13:87). An examination of reaction media revealed chlorinated solvents to suitable (Table 2, entries 5–8), whilst Selectfluor^®^ was found to be the most effective oxidant (Table 2, entries 9 and 10). Lowering catalyst loading to 10 mol % was well tolerated (Table 2, entry 11), but reactivity was suppressed at 0 °C (Table 2, entry 12). Finally, the control experiment in the absence of catalyst supports the notion of an I^I^/I^III^ cycle (Table 2, entry 13).

Prior to exploring substrate scope, it was desirable to identify potential trends that would link substrate structural features or Brønsted acidity with enantioselection. To that end, electronically diverse aryl allyl ethers were subjected to the optimized reaction conditions given in Table 2 (Figure 3).

**Figure 3:**
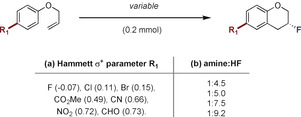
Strategy to evaluate Brønsted acid effect.

A plot of the Hammett *σ*_p_^+^ value against the log *e.r*. revealed that enantioselectivity is essentially substituent independent, which bodes well for scope expansion (Figure 4 a). As expected, however, the amine:HF ratio plays a crucial role in the relay of the chiral information (Figure 4 b) Whereas lower Brønsted acidities (1:4.5 to 1:5) induce more favorable *e.r* values, higher ratios (up to 1:9.2) compromise chiral induction.

**Figure 4:**
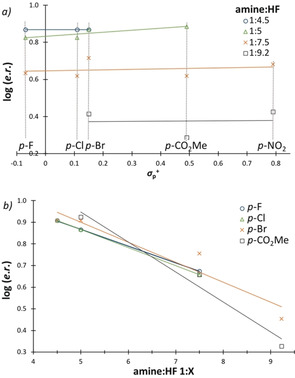
Effect of arene electron‐density and Brønsted acidity on enantioinduction.

Plotting the yield against the *σ*_p_^+^ value for defined amine:HF ratios served an important practical purpose in allowing individual substrate optimization (Figure 5 a). The optimized amine:HF ratio of 1:5 is evident from Figure 5 b with the exception of the *para*‐nitro derivative where a ratio of 1:9.2 proved to be optimal. It is pertinent to note that the effect of deactivating groups under HF/SbF_5_ super‐acid conditions has been shown to influences the relative stabilities of the Wheland intermediates.^28^

**Figure 5:**
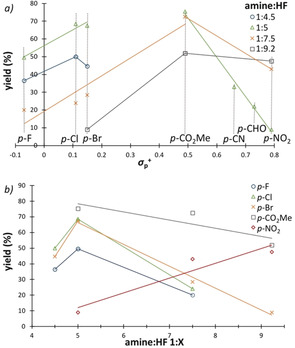
Effect of arene electron‐density and Brønsted acidity on yield.

These data indicate that synthetically useful levels of efficiency will only be reached for the most electron‐withdrawing systems (e.g. CN, NO_2_, CHO) under increased Brønsted acidic conditions. It logically follows that this will negatively impact on enantioselectivity. To enable the generation of diversely functionalized chromanes, two fluorocyclization protocols were established: Method A with an amine:HF ratio of 1:5, and Method B with a 1:7.5 ratio. Using Method A, 6‐bromo‐3‐fluorochromane (**6**) could be isolated in a synthetically useful yield of 55 % with an *e.r*. of 13:87 (Scheme 1). The halogen series showed similar results with the 3‐fluoro‐6‐chloro‐ (**8**) and 3,6‐difluoro‐chromanes (**9**) being isolated in 56 % and 44 % yield (*e.r*. 11:89 and 12:88, respectively).

**Scheme 1:**
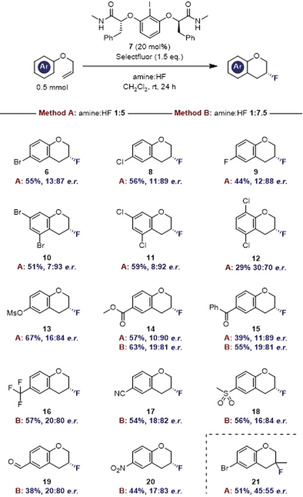
Exploring the scope of the reaction. Standard reaction conditions: allyl aryl ether (0.5 mmol), **7** (20 mol %), Selectfluor^®^ (1.5 equiv), CH_2_Cl_2_ (1.25 mL), amine:HF 1:5 (Method A) or 1:7.5 (Method B) (1.25 mL), ambient temperature, 24 h. Isolated yield of the chromane is indicated.

Exposing *meta*‐disubstituted phenyl allyl ethers to Method A provided the dibrominated and dichlorinated compounds **10** and **11** in similar yields (51 % and 59 %) and with enantiomeric ratios of 7:93 and 8:92, respectively. In contrast, the regioisomeric *ortho*‐*meta*‐dichlorinated phenyl ether furnished the desired chromane **12** with significant erosion of both yield and *e.r*. (29 %, 30:70). Mesylation of 4‐(allyloxy)phenol and subsequent fluorocyclization was an efficient strategy to access **13** in 67 % yield and 16:84 *e.r*. with the electron sink supressing formation of a potential charge‐transfer complex.^29^ In stark contrast the 4‐methoxy derivative proved recalcitrant to cyclization (see Scheme 2, **22**). The methyl ester **14** could be isolated in 57 % with an *e.r*. of 10:90 using amine:HF 1:5. When using amine:HF 1:7.5, the *e.r*. dropped to 19:81 albeit with a slight improvement in yield (63 %). The increased acidity proved crucial for phenyl ketone **15** (39 % using method A to 55 % using method B). Additional electron‐deficient substrates could also be processed to the corresponding chromanes, including the trifluoromethyl‐analog **16**, nitrile **17** and sulfone **18** (up to 57 % yield, up to 16:84 *e.r*.). A representative benzaldehyde derivative was compatible with the catalysis protocol, to furnish **19** with an *e.r*. of 20:80. The nitro derivatives **20** was isolated in 44 % yield and 17:83 *e.r*., thereby providing potential entry into amino‐chromanes.^30^ Exposure of an isobutene substrate to Method A generated chromane **21** in 51 %, but with complete loss of chiral information (45:55 *e.r*.). Taken together with the striking differences in enantioselection observed with regioisomers **11** and **12**, it is evident that the highly pre‐organized I^III^ intermediate is susceptible to subtle structural changes.

**Scheme 2:**
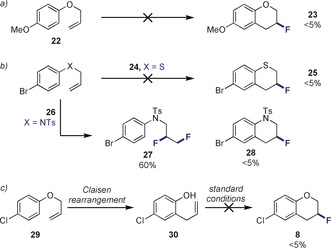
Control experiments. Standard reaction conditions: allyl aryl ether (0.2 mmol), **7** (20 mol %), Selectfluor^®^ (1.5 equiv), CH_2_Cl_2_ (0.5 mL), amine:HF 1:5 or 1:7.5 (0.5 mL), ambient temperature, 24 h. Yield determined by ^19^F NMR spectroscopy of the crude reaction mixture using ethyl fluoroacetate as internal standard.

Gratifyingly, derivative **21**, together with **17** and **20** were crystalline and it was possible to unequivocally establish structure by X‐ray analyses (Figure 6). In the case of **17** and **20**, the new C(sp^3^)−F center was determined to be (*R*)‐configured (Figure 6). Moreover, the solid‐state structures of **17** and **20** adopt a half‐chair conformation in which the stereoelectronic *gauche* effect manifests itself (σ_C‐H_→σ*_C‐F_). Torsion angles of (Φ_FCCO_) of −66.2° and −62.2° were determined in which the fluorine substituent is *pseudo*‐axial and *antiperiplanar* to neighboring donor orbitals (i.e. σ_C‐H_ bonds). In the racemic product **21**, a similar half‐chair conformation is observed with the methyl substituent *pseudo*‐equatorial. The alignment of the *pseudo*‐axial C(sp^3^)−F bond with three σ_C‐H_ bonds is a conspicuous feature. In all cases, the donor C(sp^3^)−H/CH_3_ bonds on the fluorine‐bearing carbon are *antiperiplanar* to the ring C(sp^3^)−O thereby fulfilling the stereoelectrionic requirements of this effect.^9^

**Figure 6:**
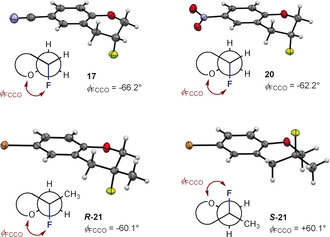
Deposition numbers 1995719 (for **17**), 1995720 for (for **20**) and 1995721 (for **21**) contain(s) the supplementary crystallographic data for this paper. These data are provided free of charge by the joint Cambridge Crystallographic Data Centre and Fachinformationszentrum Karlsruhe Access Structures service.

To further establish the scope and limitations of the reaction, and lend support to the working hypothesis, a series of control experiments were conducted (Scheme 2). As previously indicated, the electron rich *p*‐OMe derivative **22** was not compatible with these conditions (**23**, <5 %).^31^ Attempts to substitute the ether linker by a thioether, such as **24**, or by an amine (e.g. **26**) were unsuccessful (<5 %). In the case of **26**, efficient *vicinal* difluorination was observed (**27**, 60 %). To discount the possibility of an in situ Claisen rearrangement/ fluorocyclization sequence giving rise to the target 3‐fluorochromane, substrate **29** was converted to **30**. Exposure to the general catalysis conditions did not generate the product **8** with the efficiency described in Scheme 1 (Method A: 56 %).

Finally, to interrogate stereospecificity in the title fluorocyclization, the deuterated *Z*‐ and *E*‐configured alkenes **30** and **31** were prepared (Scheme 3). Upon independently exposing these substrates to the general conditions using catalyst **7** (please see the Supporting Information), **30** was smoothly converted to **32**, whereas **31** was processed to **33**. The coupling constants ^3^ *J*_FD_ and ^3^ *J*_FH_^a^, revealed the relative *anti*‐configuration for **32** (derived from the *Z*‐alkene **30**) with a large ^3^ *J*_FD_ of 5.3 Hz (equivalent to a ^3^ *J*_FH_ of 34.5 Hz) and a small ^3^ *J*_FH_^a^ of 16.7 Hz. By comparison, the *syn*‐configured chromane **33** displayed a small ^3^ *J*_FD_ of 2.3 Hz (equivalent to a ^3^ *J*_FH_ of 15.0 Hz) and a larger ^3^ *J*_FH_^a^ of 35.4 Hz.^32^ This analysis was conducted with both the (*R*,*R*)‐catalyst and the (*S*,*S*)‐catalyst for completeness.

**Scheme 3:**
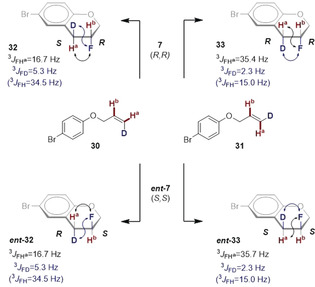
Exploring stereospecificity with deuterated probes.

The stereospecific relay of information [*Z*→*anti* and *E*→*syn*] allow this transformation to be characterized according to the nomenclature established by Denmark and co‐workers for *vicinal* dihalogenation reactions.^33^ It is conceivable that the fluorocyclization of aryl allyl ethers under the auspices of I^I^/I^III^ catalysis might follow a type II_*inv*_ pathway (Scheme 4).

**Scheme 4:**
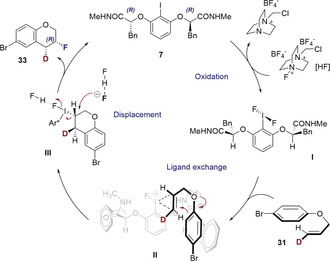
A tentative catalytic cycle and induction model invoking a type II_inv_ pathway.

Initially, Selectfluor^®^‐mediated oxidation of catalyst **7** enables in situ formation of the transient difluoro(aryl)‐λ^3^‐iodane **I** in which H‐bonding is important in orchestrating induction (please see Table 1, entries 3 and 5). Ligand exchange at the iodine center with the substrate alkene and concomitant cyclization (**II**) is likely pre‐organized by stabilizing aromatic interactions. This is supported by the results disclosed in Table 1, entries 5 and 9. Nucleophilic displacement (**III**) and closure of the catalytic cycle provides a rationale for the relative *syn*‐configuration and stereospecificity that was determined by detailed NMR analysis (**31**→**33**).

In conclusion, an operationally simple, direct fluoro‐cyclization of aryl allyl ethers to access biologically relevant enantio‐enriched 3‐fluorochromanes is disclosed. Selectivities up to 7:93 *e.r*. can be obtained using a simple *C*_2_‐symmetric iodoresorcinol catalyst in combination with Selectfluor^®^ and simple amine:HF combinations. X‐ray crystallographic analyses of representative products reveal conformations that enable stabilizing stereoelectronic interactions. This physicochemical consideration renders these materials potentially valuable as drug discovery modules. Mechanistic interrogation of the reaction using deuterated probes reveals that the process is stereospecific and likely follows a type II_inv_ pathway.

## Experimental Section

Full details are provided in the Supporting Information.

## Conflict of interest

The authors declare no conflict of interest.

## Supporting information

As a service to our authors and readers, this journal provides supporting information supplied by the authors. Such materials are peer reviewed and may be re‐organized for online delivery, but are not copy‐edited or typeset. Technical support issues arising from supporting information (other than missing files) should be addressed to the authors.

Supplementary
