# Supplementary material for: Enantioselective Synthesis of 3‐Fluorochromanes via Iodine(I)/Iodine(III) Catalysis
Source: Angew Chem Int Ed Engl. 2020 Jun 9;59(35):15069–75. doi: 10.1002/anie.202005181 (PMC7496101; doi:10.1002/anie.202005181)
Supplement: Supplementary file 1 — Supplementary [file ANIE-59-15069-s001.pdf]

Angewandte Chemie
Eine Zeitschrift der Gesellschaft Deutscher Chemiker

Supporting Information

Enantioselective Synthesis of 3-Fluorochromanes via Iodine(I)/Iodine(III) Catalysis

*Jérôme C. Sarie, Christian Thiehoff, Jessica Neufeld, Constantin G. Daniliuc, and Ryan Gilmour**

anie_202005181_sm_miscellaneous_information.pdf

Contents

1. General Information 3
2. Synthesis of 3-Fluorochromanes 4
   a. Preparation of Precatalysts 4
   b. Synthesis of Starting Materials 14
   c. Synthesis of Chromanes 28
3. Mechanistic Study 43
   a. Electronic Modification of Aryl Substituent 43
   b. Substrate Modification Experiments 44
   c. Deuteration Experiments 45
      1. From Catalyst {*R*,*R*}-5 45
      2. From Catalyst {*S*,*S*}-5 48
4. HPLC traces 51
5. X-ray Analysis 69
6. NMR Spectra 72
   a. NMR Spectra of Catalysts and Phenyl Allyl Ethers 72
   b. NMR Spectra of 3-Fluorochromanes 88
7. References 128

1. General Information

All chemicals were purchased as reagent grade and used as received. Dry solvents were dried by a Grubbs purification system including columns packed with molecular sieves and aluminium oxide. Solvents for extraction or purification were purchased as technical grade and distilled on the rotary evaporator prior to use. Column chromatography was performed using silica gel (40-63 μm; *VWR Chemicals*) as stationary phase. Reaction monitoring was achieved by analytical thin layer chromatography (TLC) on aluminum foil pre-coated with silica gel 60 F_{254} (*Merck*). Compounds were visualised with UV light (254 nm) or by chemical staining using solution of $KMnO_4$ ($KMnO_4$ (10 g), K_2CO_3 (65 g), NaOH (1 N, 15 mL) in water (1 L)) followed by heating. Concentration *in vacuo* was performed at ~10 mbar at 40 °C unless otherwise stated. NMR spectra were measured by the NMR service in the Organisch-Chemisches Institut, Westfälische Wilhelms-Universität Münster on a *Bruker Avance II 300*, a *Bruker Avance II 400,* an *Agilent DD2 500* or an *Agilent DD2 600* spectrometer at 298 K. ^{1}H NMR chemical shifts are given relative to TMS and are referenced to the residual solvent peak as internal standard. Spectra of other nuclides as ^{13}C and ^{19}F are referenced according to the proton resonance of TMS as the primary reference for the unified chemical shift scale. ^{1}H NMR spectra are reported as follows: chemical shift δ in ppm (multiplicity, coupling constant J_{FH} and J_{HH} in Hz, number of protons, assignment of proton). ^{13}C NMR spectra are reported as follows: chemical shift δ in ppm (multiplicity, coupling constant J_{FC} in Hz, number of carbons, assignment of carbon). ^{19}F NMR spectra are reported as follows: chemical shift δ in ppm (multiplicity, coupling constant J_{FH} in Hz, number of fluorines, assignment of fluorine). The resonance multiplicity is abbreviated as s (singlet), d (doublet), t (triplet), q (quartet), p (pentet) or m (multiplet). Assignments of unknown compounds are based on COSY, HMBC and HSQC spectra. Melting points were measured on a *Büchi B-545* melting point apparatus in open capillaries and are uncorrected. IR spectra were recorded on a *Perkin-Elmer 100 FT-IR* spectrometer. Absorption bands are reported in wave numbers (cm^{-1}) and the intensities are reported as w (weak), m (medium), s (strong). Mass spectra were measured by the MS service of the Organisch-Chemisches Institut, Westfälische Wilhelms-Universität on a *Bruker Daltonics MicroTof* (ESI-EM), *Triplequad Quattro Micro GC* (EI), *Trace 1310* with *ISQ 7000 Single Quad Mass Spectrometer (Thermo Fisher Scientific)* (EI) or a *Trace 1310* with *GC Exactive Orbitrap (Thermo Fisher Scientific)* (EI-EM). Enantiomeric ratios were determined on an Agilent Infinity 1260 HPLC system using a diode array detector (DAD). The chiral stationary phases were OJ-H, AS-H, NR, OM and AM. The eluent of *n*-hexane and *i*-propanol is specified for each compound. The column temperature measured 25 to 35 °C.

HF sources

Et_3N:3HF = amine:HF / 1:3.0

Pyr:$(HF)_x$ (*Olah*'s reagent) = amine:HF / 1:9.23 (calculated based on the physical data provided by the supplier, *Sigma-Aldrich*)

A mixture of amine:HF / 1:4.5 was obtained by mixing 0.159 mL of Pyr:$(HF)_x$ and 0.341 mL of Et_3N:3HF.

A mixture of amine:HF / 1:5.0 was obtained by mixing 0.205 mL of Pyr:$(HF)_x$ and 0.295 mL of Et_3N:3HF.

A mixture of amine:HF / 1:7.5 was obtained by mixing 0.402 mL of Pyr:$(HF)_x$ and 0.098 mL of Et_3N:3HF.

2. Synthesis of 3-Fluorochromanes

a. Preparation of Precatalysts

2-Iodobenzene-1,3-diol (**S1**)

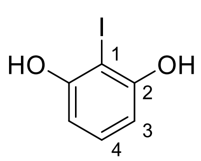

Prepared according to a modified literature procedure.[1] $NaHCO_3$ (4.66 g, 55.5 mmol, 1.11 equiv.) was added at 0 °C in small portions to a solution of resorcinol (5.50 g, 50.0 mmol, 1.0 equiv.) and I_2 (13.58 g, 53.5 mmol, 1.07 equiv.) in H_2O (40 mL). The mixture was stirred at room temperature for an additional 40 min and was then extracted with EtOAc (3 x 50 mL). The combined organic layers were washed with an aqueous solution of $Na_2S_2O_3$ (10%), brine, dried over Na_2SO_4, filtered and concentrated under reduced pressure. The residue was purified by trituration with ice-cold $CHCl_3$ affording the desired product as a cream-coloured solid (5.77 g, 49%).

M.p.: 102 – 103 °C.

^{1}H NMR (400 MHz, acetone-*d$_6$*): *δ* 8.84 (br, 2H, O-H), 7.00 (t, *J* = 8.0 Hz, 1H, H-C4), 6.46 (d, *J* = 8.1 Hz, 2H, H-C3).

^{13}C{^{1}H} NMR (101 MHz, acetone-*d$_6$*): *δ* 158.9 (2C, C2), 130.4 (1C, C4), 107.0 (2C, C3), 75.3 (1C, C1).

ESI-EM-MS: (*m/z*) required: $[(C_6H_4IO_2)]^-$ = 234.9261; (*m/z*) found: $[(C_6H_4IO_2)\text{-}H]^-$ = 234.9263.

FT-IR (ATR) ($\tilde{\nu}$ = cm^{-1}): 3450 (m), 3343 (m), 1919 (w), 1593 (m), 1579 (m), 1490 (m), 1453 (s), 1354 (m), 1303 (m), 1276 (m), 1246 (m), 1176 (s), 1157 (s), 1021 (m), 990 (s), 861 (m), 773 (s), 701 (s).

Analytical data in agreement with literature.[2]

2-Iodo-5-methylbenzene-1,3-diol (**S2**)

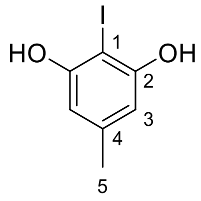

Prepared according to a modified literature procedure.[3] 5-Methylresorcinol (1.24 g, 10.0 mmol, 1.0 equiv.) was dissolved in MeCN (20 mL) and NIS (2.25 g, 10.0 mmol, 1.0 equiv.) was added at 0 °C. The mixture was stirred at 0 °C for 15 min and then quenched by the addition of a saturated aqueous solution of $Na_2S_2O_3$ (20 mL). The aqueous layer was extracted with EtOAc (3 x 20 mL) and the combined organic layers were washed with brine, dried over Na_2SO_4, filtered and concentrated under reduced pressure. Purification by column chromatography on silica gel (*n*-pentane:EtOAc 1:1) afforded the desired product as a white solid (2.34 g, 93%).

R$_f$ = 0.53 (*n*-pentane:EtOAc 1:1).

M.p.: 105 – 107 °C.

^{1}H NMR (400 MHz, acetone-*d$_6$*): *δ* 8.63 (br, 2H, O-H), 6.32 (s, 2H, H-C3), 2.15 (s, 3H, H-C5).

^{13}C{^{1}H} NMR (101 MHz, acetone-*d$_6$*): *δ* 158.5 (2C, C2), 140.6 (1C, C4), 108.1 (2C, C3), 71.4 (1C, C1), 21.1 (1C, C5).

ESI-EM-MS: (*m/z*) required: $[(C_7H_6IO_2)]^-$ = 248.9418; (*m/z*) found: $[(C_7H_6IO_2)]^-$ = 248.9457.

FT-IR (ATR) ($\tilde{\nu}$ = cm^{-1}): 3459 (m), 3262 (m), 2915 (w), 1609 (m), 1584 (m), 1495 (m), 1425 (m), 1374 (w), 1336 (m), 1275 (m), 1250 (m), 1170 (s), 1044 (m), 1020 (m), 984 (m), 863 (m), 824 (s), 704 (m).

Analytical data in agreement with literature.[4]

Methyl 3,5-dihydroxy-4-iodobenzoate (S3)

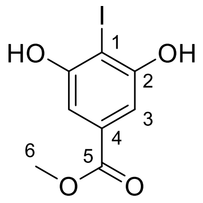

This compound was prepared according to a modified procedure.[1] Methyl-3,5-dihydroxybenzoate (500 mg, 2.97 mmol, 1.0 eq.) and sodium bicarbonate (750 mg, 8.92 mmol, 3.0 eq.) were dissolved in water (5 mL) and THF (1.5 mL). The solution was cooled to 0 °C and a solution of iodine (1.5 g, 5.94 mmol, 2.0 eq.) in THF (3.6 mL) was added dropwise over 30 minutes. The reaction mixture was stirred at 0 °C for 1 h. Upon completion, MTBE (2 mL) and water (1.5 mL) were added to the reaction mixture which was then quenched by addition of a saturated solution of sodium thiosulfate. The aqueous phase was extracted with MTBE (2x) and the combined organic layers were washed with brine, dried over $MgSO_4$, filtered and evaporated under *vacuo* to afford the desired product as an off-white solid (838 mg, 96%).

M.p.: 211 °C (dec.).

^{1}H NMR (300 MHz, DMSO-*d*$_6$): δ 10.53 (s, 2H, H-O), 6.93 (s, 2H, H-C3), 3.80 (s, 3H, H-C6).

ESI-EM-MS: (*m/z*) required: $[(C_8H_7IO_4)Na]^+$ = 316.9281; (*m/z*) found: $[(C_8H_7IO_4)Na]^+$ = 316.9276.

Analytical data in agreement with literature.[3]

(*S*)-2-Hydroxy-3-phenylpropanoic acid (S4)

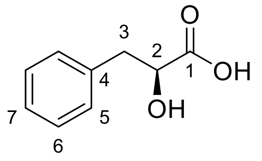

Prepared according to a modified literature procedure.[4] L-Phenylalanine (16.5 g, 100 mmol, 1.0 equiv.) was dissolved in H_2SO_4 (0.5 M, 400 mL) and a solution of $NaNO_2$ (34.5 g, 500 mmol, 5.0 equiv.) in H_2O (150 mL) was added over 2 h at 0 °C. Stirring was continued for 45 h while warming to room temperature. The suspension was then extracted with Et_2O (3 x 400 mL) and the combined organic layers were washed with brine, dried over $MgSO_4$, filtered and concentrated under reduced pressure. The obtained residue was dissolved in boiling Et_2O and then precipitated by addition of *n*-pentane to afford the desired product as a white crystalline solid (14.1 g, 85%).

M.p.: 125 – 126 °C.

$[\alpha]_D^{25}$: -27.7° (*c* = 1.00, acetone).

^{1}H NMR (400 MHz, $CDCl_3$): δ 7.33 – 7.20 (m, 5H, H-C5/6/7), 4.41 (dd, $^3J_{HH}$ = 7.4, 4.2 Hz, 1H, H-C2), 4.15 (br, 2H, O-H/COO-H), 3.16 (dd, $^2J_{HH}$ = 14.0, $^3J_{HH}$ = 4.2 Hz, 1H, H-C3), 2.94 (dd, $^2J_{HH}$ = 14.0, $^3J_{HH}$ = 7.4 Hz, 1H, H-C3).

^{13}C{^{1}H} NMR (101 MHz, $CDCl_3$): δ 176.3 (1C, C1), 136.7 (1C, C4), 129.6 (2C, C5), 128.5 (2C, C6), 126.9 (1C, C7), 71.1 (1C, C2), 40.4 (1C, C3).

ESI-EM-MS: (*m/z*) required: $[(C_9H_9O_3)]^-$ = 165.0557; (*m/z*) found: $[(C_9H_9O_3)]^-$ = 165.0551.

FT-IR (ATR) ($\tilde{\nu}$ = cm^{-1}): 3439 (w), 2928 (w), 1724 (m), 1495 (w), 1456 (w), 1432 (w), 1307 (m), 1240 (m), 1191 (m), 1090 (s), 1067 (m), 1002 (w), 912 (m), 880 (m), 795 (m), 739 (m), 762 (w), 700 (s).

Analytical data in agreement with literature.[4]

Methyl (*S*)-2-hydroxy-3-phenylpropanoate (S5)

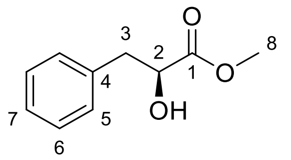

Prepared according to a modified literature procedure.[5] Thionyl chloride (2.65 mL, 36.5 mmol, 1.0 equiv.) was carefully added to a solution of **S4** (6.06 g, 36.5 mmol, 1.0 equiv.) in MeOH (90 mL) at 0 °C. After heating the solution to 75 °C for 4 h the mixture was evaporated to dryness and the residue was purified by column chromatography on silica gel (CyH:EtOAc 4:1) affording the desired product as a white solid (5.84 g, 93%).

R*f* = 0.23 (CyH:EtOAc 4:1).

M.p.: 46 – 48 °C.

$[\alpha]_{\mathrm{D}}^{25}$: -7.9° (*c* = 1.00, $CHCl_3$).

^{1}H NMR (400 MHz, $CDCl_3$): *δ* 7.33 – 7.27 (m, 2H, H-C6), 7.27 – 7.23 (m, 1H, H-C7), 7.23 – 7.19 (m, 2H, H-C5), 4.46 (ddd, $^3J_{HH}$ = 6.8, 4.4, 3.6 Hz, 1H, H-C2), 3.77 (s, 3H, H-C8), 3.13 (dd, $^2J_{HH}$ = 13.9, $^3J_{HH}$ = 4.4 Hz, 1H, H-C3), 2.96 (dd, $^2J_{HH}$ = 13.9, $^3J_{HH}$ = 6.8 Hz, 1H, H-C3), 2.74 (d, $^3J_{HH}$ = 3.6 Hz, 1H, O-H).

^{13}C{^{1}H} NMR (101 MHz, $CDCl_3$): *δ* 174.7 (1C, C1), 136.4 (1C, C4), 129.6 (2C, C5), 128.6 (2C, C6), 127.0 (1C, C7), 71.4 (1C, C2), 52.6 (1C, C8), 40.7 (1C, C3).

ESI-EM-MS: (*m/z*) required: $[(C_{10}H_{12}O_3)Na]^+$ = 203.0679; (*m/z*) found: $[(C_{10}H_{12}O_3)Na]^+$ = 203.0687.

FT-IR (ATR) ($\tilde{\nu}$ = cm^{-1}): 3267 (w), 3024 (w), 2949 (w), 1749 (s), 1732 (s), 1604 (w), 1496 (w), 1456 (m), 1430 (m), 1335 (w), 1276 (m), 1260 (s), 1212 (m), 1174 (m), 1099 (s), 1020 (m), 995 (m), 976 (m), 928 (w), 877 (w), 866 (m), 828 (m), 756 (s), 744 (m), 701 (s), 676 (s).

Analytical data in agreement with literature.[2]

Methyl (*R*)-2-(2-iodophenoxy)-3-phenylpropanoate (S6)

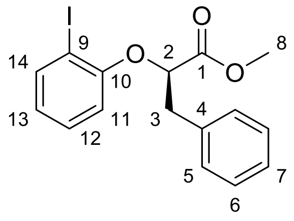

Prepared according to a modified literature procedure.[1] PPh_3 (3.15 g, 12.0 mmol, 1.2 equiv.) was added to a solution of 2-iodophenol (2.20 g, 10.0 mmol, 1.0 equiv.) and **S5** (1.98 g, 11.0 mmol, 1.1 equiv.) in THF (20 mL) under argon. Then, DIAD (2.56 mL, 13.0 mmol, 1.3 equiv.) was slowly added causing an exothermic reaction. The mixture was stirred at room temperature for 48 h and then evaporated to dryness. Purification by column chromatography on silica gel (CyH:EtoAC 20:1) afforded the desired product as a colourless oil (2.98 g, 78%).

R*f* = 0.43 (CyH:EtOAc 10:1);

$[\alpha]_{\mathrm{D}}^{25}$: +39.7° (*c* = 1.00, $CHCl_3$);

^{1}H NMR (400 MHz, $CDCl_3$): *δ* 7.77 (dd, $^3J_{HH}$ = 7.8 Hz, $^4J_{HH}$ = 1.6 Hz, 1H, H-C14), 7.44 – 7.40 (m, 2H, H-C5), 7.34 – 7.28 (m, 2H, H-C6), 7.28 – 7.23 (m, 1H, H-C7), 7.20 (ddd, $^3J_{HH}$ = 8.3 Hz, *J* = 7.4 Hz, $^4J_{HH}$ = 1.6 Hz, 1H, H-C12), 6.70 (td, $^3J_{HH}$ = 7.8 Hz, *J* = 4.8 Hz, 1H, H-C13), 6.57 (dd, $^3J_{HH}$ = 8.3 Hz, *J* = 1.3 Hz, 1H, H-C11), 4.83 (dd, $^3J_{HH}$ = 7.8, 4.8 Hz, 1H, H-C2), 3.70 (s, 3H, H-C8), 3.36 (dd, $^2J_{HH}$ = 14.0 Hz, $^3J_{HH}$ = 7.8 Hz, 1H, H-C3), 3.30 (dd, $^2J_{HH}$ = 14.0 Hz, $^3J_{HH}$ = 4.7 Hz, 1H, H-C3).

^{13}C{^{1}H} NMR (101 MHz, $CDCl_3$): *δ* 171.1 (1C, C1), 156.6 (1C, C10), 140.0 (1C, C14), 136.1 (1C, C4), 130.0 (2C, C5), 129.4 (1C, C12), 128.6 (2C, C6), 127.2 (1C, C7), 123.5 (1C, C13), 112.6 (1C, C11), 86.8 (1C, C9), 78.9 (1C, C2), 52.5 (1C, C8), 39.3 (1C, C3).

ESI-EM-MS: (*m/z*) required: [($C_{16}H_{15}IO_3$)Na]$^+$ = 404.9958; (*m/z*) found: [($C_{16}H_{15}IO_3$)Na]$^+$ = 404.9959.

FT-IR (ATR) ($\tilde{\nu}$ = cm^{-1}): 3030 (w), 2951 (w), 1756 (m), 1733 (m), 1582 (w), 1571 (w), 1497 (w), 1470 (s), 1455 (w), 1438 (m), 1359 (w), 1276 (m), 1238 (m), 1196 (m), 1175 (m), 1122 (w), 1082 (m), 1017 (s), 977 (w), 930 (w), 896 (w), 834 (w), 795 (w), 743 (s), 698 (s).

(*R*)-2-(2-Iodophenoxy)-*N*-methyl-3-phenylpropanamide (**S7**)

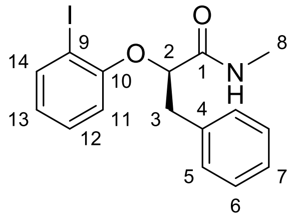

Prepared according to a modified literature procedure.[6] A solution of **S6** (191 mg, 0.5 mmol, 1.0 equiv.) in $MeNH_2$ in EtOH (33 wt%, 5 mL, 40.0 mmol, 80.0 equiv.) was stirred at room temperature for 2 h and then evaporated to dryness. The obtained residue was dissolved in acetone and precipitated by addition of *n*-pentane affording the desired product as a white solid (137 mg, 72%).

M.p.: 124 – 125 °C.

$[\alpha]_D^{25}$: +13.3° (*c* = 1.00, $CHCl_3$).

^{1}H NMR (500 MHz, $CDCl_3$): δ 7.76 (dd, *J* = 7.8, 1.6 Hz, 1H, H-C14), 7.33 – 7.29 (m, 2H, H-C5), 7.29 – 7.25 (m, 2H, H-C6), 7.24 – 7.20 (m, 2H, H-C7/12), 6.73 (td, *J* = 7.6, 1.3 Hz, 1H, H-C13), 6.62 (dd, *J* = 8.4, 1.3 Hz, 1H, H-C11), 6.35 (br, 1H, NH), 4.89 (dd, *J* = 6.6, 3.6 Hz, 1H, H-C2), 3.36 (dd, *J* = 14.2, 3.6 Hz, 1H, H-C3), 3.24 (dd, *J* = 14.2, 6.5 Hz, 1H, H-C3), 2.73 (d, *J* = 4.9 Hz, 3H, H-C8).

^{13}C{^{1}H} NMR (126 MHz, $CDCl_3$): δ 170.6 (1C, C1), 155.7 (1C, C10), 139.9 (1C, C14), 136.1 (1C, C4), 130.2 (2C, C5), 129.8 (1C, C12), 128.4 (2C, C6), 127.0 (1C, C7), 123.7 (1C, C13), 113.0 (1C, C11), 86.7 (1C, C9), 80.4 (1C, C2), 38.7 (1C, C3), 26.0 (1C, C8).

ESI-EM-MS: (*m/z*) required: [($C_{16}H_{16}INO_2$)Na]$^+$ = 404.0118; (*m/z*) found: [($C_{16}H_{16}INO_2$)Na]$^+$ = 404.0125.

FT-IR (ATR) ($\tilde{\nu}$ = cm^{-1}): 3257 (w), 3086 (w), 3032 (w), 2941 (w), 1655 (m), 1575 (m), 1496 (w), 1469 (m), 1453 (m), 1437 (m), 1412 (w), 1375 (w), 1275 (m), 1261 (m), 1239 (m), 1212 (w), 1197 (w), 1161 (m), 1120 (m), 1087 (m), 1069 (w), 1040 (m), 1029 (m), 1017 (m), 995 (w), 943 (w), 919 (w), 892 (w), 841 (w), 790 (w), 757 (m), 744 (m), 695 (s).

Dimethyl 2,2'-((2-iodo-1,3-phenylene)bis(oxy))(2*R*,2'*R*)-dipropionate (**S8**)

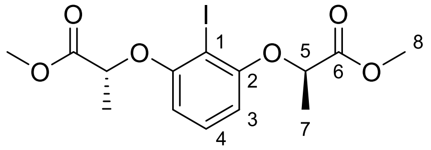

Prepared according to a modified literature procedure.[7] A flame-dried flask was charged with **S1** (1.18 g, 5.0 mmol, 1.0 equiv.) and PPh_3 (3.02 g, 11.5 mmol, 2.3 equiv.). Dry THF (10 mL) and methyl-(*S*)-(-)-lactate (1.05 mL, 11.0 mmol, 2.2 equiv.) were added under argon. Then, DIAD (2.36 mL, 12.0 mmol, 2.4 equiv.) was added dropwise and the reaction was stirred at room temperature for 18 h. The mixture was concentrated under reduced pressure. Purification by column chromatography on silica gel (CyH:EtOAc 5:1, dry load) afforded the desired product as a white solid (1.32 g, 65%).

R*f* = 0.45 (CyH/EtOAc 5:1).

M.p.: 48 – 49 °C.

$[\alpha]_D^{25}$: -19.8° (*c* = 1.00, $CHCl_3$).

^{1}H NMR (400 MHz, $CDCl_3$): δ 7.13 (t, $^3J_{HH}$ = 8.3 Hz, 1H, H-C4), 6.36 (d, $^3J_{HH}$ = 8.3 Hz, 2H, H-C3), 4.76 (q, $^3J_{HH}$ = 6.8 Hz, 2H, H-C5), 3.74 (s, 6H, H-C8), 1.70 (d, $^3J_{HH}$ = 6.8 Hz, 6H, H-C7).

^{13}C{^{1}H} NMR (101 MHz, $CDCl_3$): δ 172.3 (2C, C6), 158.4 (2C, C2), 129.8 (1C, C4), 107.0 (2C, C3), 80.8 (1C, C1), 74.3 (2C, C5), 52.5 (2C, C8), 18.8 (2C, C7).

ESI-EM-MS: (*m/z*) required: $[(C_{14}H_{17}IO_6)Na]^+$ = 430.9962; (*m/z*) found: $[(C_{14}H_{17}IO_6)Na]^+$ = 430.9953.

FT-IR (ATR) ($\tilde{\nu}$ = cm^{-1}): 2957 (w), 1727 (s), 1692 (w), 1584 (m), 1569 (w), 1456 (s), 1439 (m), 1373 (w), 1278 (s), 1247 (s), 1179 (w), 1131 (s), 1097 (s), 1063 (s), 1020 (s), 973 (m), 958 (m), 929 (w), 877 (w), 838 (m), 831 (m), 770 (m), 752 (s), 704 (m).

Analytical data in agreement with literature.[8]

(2*R*,2'*R*)-2,2'-((2-Iodo-1,3-phenylene)bis(oxy))dipropionic acid (S9)

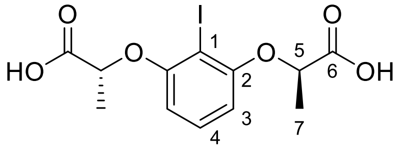

Prepared according to a modified literature procedure.[9] To a solution of **S8** (612 mg, 1.50 mmol, 1.0 equiv.) in a mixture of THF (5 mL) and MeOH (5 mL) was added an aqueous solution of NaOH (2 M, 4.13 mL). After stirring at room temperature for 5 h, the reaction was acidified with HCl (1 M) and then extracted with EtOAc (3 x 30 mL). The combined organic layers were washed with H_2O, dried over Na_2SO_4, filtered and concentrated under reduced pressure affording the desired product as an off-white solid (570 mg, quant.).

M.p.: 96 – 98 °C.

$[\alpha]_D^{25}$: -12.1° (*c* = 1.00, THF).

^{1}H NMR (400 MHz, $CDCl_3$): δ 7.12 (t, $^3J_{HH}$ = 8.3 Hz, 1H, H-C4), 6.38 (d, $^3J_{HH}$ = 8.4 Hz, 2H, H-C3), 4.70 (q, $^3J_{HH}$ = 6.8 Hz, 2H, H-C5), 3.48 (br, 2H, OH), 1.67 (d, $^3J_{HH}$ = 6.9Hz, 6H, H-C7).

^{13}C{^{1}H} NMR (101 MHz, $CDCl_3$): δ 174.0 (2C, C6), 158.2 (2C, C2), 129.7 (1C, C4), 107.1 (2C, C3), 80.5 (1C, C1), 74.1 (2C, C5), 18.6 (2C, C7).

ESI-EM-MS: (*m/z*) required: $[(C_{12}H_{12}IO_6)]^-$ = 378.9673; (*m/z*) found: $[(C_{12}H_{12}IO_6)]^-$ = 378.9677.

FT-IR (ATR) ($\tilde{\nu}$ = cm^{-1}): 2942 (w), 2533 (w), 1698 (m), 1581 (m), 1458 (m), 1418 (w), 1375 (w), 1322 (w), 1249 (m), 1226 (m), 1181 (m), 1134 (s), 1100 (s), 1069 (m), 1022 (m), 1007 (m), 940 (m), 832 (w), 759 (m), 698 (m).

Analytical data in agreement with literature.[9]

Dimethyl 2,2'-((2-iodo-5-methyl-1,3-phenylene)bis(oxy))(2*R*,2'*R*)-dipropionate (S10)

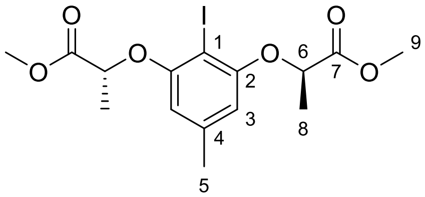

Prepared according to a modified literature procedure.[10] A flame-dried flask was charged with **S2** (500 mg, 2.0 mmol, 1.0 equiv.) and PPh_3 (1.21 g, 4.6 mmol, 2.3 equiv.). Dry THF (10 mL) and methyl-(*S*)-(-)-lactate (0.42 mL, 4.4 mmol, 2.2 equiv.) were added under argon. Then, DIAD (0.95 mL, 4.8 mmol, 2.4 equiv.) was added dropwise and the reaction was stirred at room temperature for 16 h. The mixture was concentrated under reduced pressure.

Purification by column chromatography on silica gel (CyH:EtOAc 10:1 → 7:1, dry load) afforded the desired product as a white solid (629 mg, 74%).

R$_f$ = 0.24 (CyH:EtOAc 5:1).

M.p.: 56 – 58 °C.

$[\alpha]_D^{25}$: -3.5° (*c* = 1.00, $CHCl_3$).

^{1}H NMR (400 MHz, $CDCl_3$): δ 6.20 (s, 2H, H-C3), 4.74 (q, $^3J_{HH}$ = 6.8 Hz, 2H, H-C6), 3.75 (s, 6H, H-C9), 2.25 (s, 3H, H-C5), 1.68 (d, $^3J_{HH}$ = 6.7 Hz, 6H, H-C8).

^{13}C{^{1}H} NMR (101 MHz, $CDCl_3$): δ 172.4 (2C, C7), 158.1 (C2, C2), 140.3 (1C, C4), 108.3 (2C, C3), 76.9 (1C, C1), 74.3 (2C, C6), 52.5 (2C, C9), 22.0 (1C, C5), 18.8 (2C, C8).

ESI-EM-MS: (*m/z*) required: $[(C_{15}H_{19}IO_6)Na]^+$ = 445.0119; (*m/z*) found: $[(C_{15}H_{19}IO_6)Na]^+$ = 445.0120.

FT-IR (ATR) ($\tilde{\nu}$ = cm^{-1}): 2958 (w), 2924 (w), 1725 (s), 1575 (m), 1439 (m), 1374 (w), 1330 (m), 1307 (m), 1276 (s), 1238 (s), 1134 (s), 1107 (s), 1070 (s), 1035 (s), 1022 (m), 1007 (m), 960 (m), 920 (w), 885 (w), 835 (m), 808 (s), 754 (m), 712 (w), 666 (m).

Analytical data in agreement with literature.[10]

Dimethyl 2,2'-((2-iodo-5-(methoxycarbonyl)-1,3-phenylene)bis(oxy))(2*R*,2'*R*)-bis(3-phenylpropanoate) (S11)

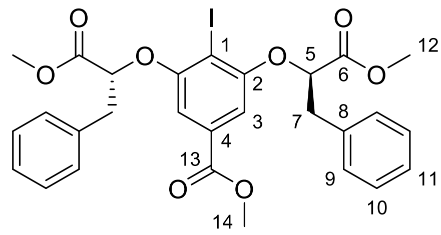

A flame-dried flask was charged with **S3** (294 mg, 1.00 mmol, 1.0 equiv.), **S5** (378 mg, 2.10 mmol, 2.1 equiv.) and PPh_3 (708 mg, 2.70 mmol, 2.7 equiv.) and THF (10 mL) was added under argon. The solution was cooled to 0 °C and DIAD (452 µL, 2.30 mmol, 2.3 equiv.) was added dropwise. The ice-bath was removed and the mixture was stirred at room temperature for 17 h. The mixture was evaporated to dryness. Purification by column chromatography on silica gel and aluminium oxide (1:1 w/w, aluminium oxide on top of silica gel, CyH:EtOAc 4:1) afforded a white foam (329 mg, 53%).

R$_f$ = 0.25 (CyH:EtOAc 4:1).

M.p.: 52 – 54 °C.

$[\alpha]_D^{25}$: +54.7° (*c* = 0.50, $CHCl_3$).

^{1}H NMR (300 MHz, $CDCl_3$): δ 7.46 – 7.39 (m, 4H, H-C9), 7.35 – 7.22 (m, 6H, H-C10, H-C11), 6.86 (s, 2H, H-C3), 4.93 (dd, *J* = 7.4, 5.0 Hz, 2H, H-C5), 3.83 (s, 3H, H-C14), 3.69 (s, 6H, H-C12), 3.41 – 3.27 (m, 4H, H-C7).

ESI-EM-MS: (*m/z*) required: $[(C_{28}H_{27}IO_8)Na]^+$ = 641.0643; (*m/z*) found: $[(C_{28}H_{27}IO_8)Na]^+$ = 641.0639.

Analytical data in agreement with literature.[11]

Dimethyl 2,2'-((2-iodo-1,3-phenylene)bis(oxy))(2*R*,2'*R*)-bis(3-phenylpropanoate) (**S12**)

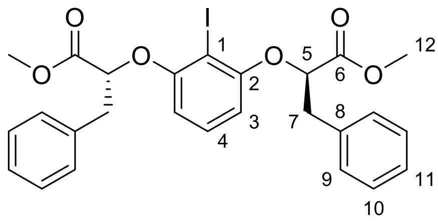

A flame-dried flask was charged with **S1** (484 mg, 2.05 mmol, 1.0 equiv.), **S5** (811 mg, 4.50 mmol, 2.2 equiv.) and PPh_3 (1.24 g, 4.70 mmol, 2.3 equiv.) and dry THF (5 mL) was added under argon. The solution was cooled to 0 °C and DIAD (0.97 mL, 4.90 mmol, 2.4 equiv.) was added dropwise. The ice-bath was removed and the mixture was stirred at room temperature for 16 h. The mixture was evaporated to dryness. Purification by column chromatography on silica gel and aluminium oxide (1:1 w/w, aluminium oxide on top of silica gel, CyH:EtOAc 20:1 → 5:1) afforded a white solid (662 mg, 58%). If further purification is necessary the product can be dissolved in boiling Et_2O and precipitated by addition of *n*-pentane.

$\mathbf{R}_f$ = 0.41 (CyH:EtOAc 4:1).

M.p.: 115 – 116 °C.

$[\alpha]_D^{25}$: +65.4° (*c* = 1.00, $CHCl_3$).

^{1}H NMR (400 MHz, $CDCl_3$): δ 7.45 – 7.41 (m, 4H, H-C9), 7.34 – 7.28 (m, 4H, H-C10), 7.28 – 7.22 (m, 2H, H-C11), 7.04 (t, $^3J_{HH}$ = 8.3 Hz, 1H, H-C4), 6.21 (d, $^3J_{HH}$ = 8.3 Hz, 2H, H-C3), 4.81 (dd, $^3J_{HH}$ = 8.0, 4.5 Hz, 2H, H-C5), 3.67 (s, 6H, H-C12), 3.36 (dd, $^2J_{HH}$ = 14.0, $^3J_{HH}$ = 8.0 Hz, 2H, H-C7), 3.29 (dd, $^2J_{HH}$ = 14.0, $^2J_{HH}$ = 4.5 Hz, 2H, H-C7).

^{13}C{^{1}H} NMR (101 MHz, $CDCl_3$): δ 171.1 (2C, C6), 158.2 (2C, C2), 136.2 (2C, C8), 130.0 (4C, C9), 129.6 (1C, C4), 128.5 (4C, C10), 127.2 (2C, C11), 106.0 (2C, C3), 79.5 (1C, C1), 79.0 (2C, C5), 52.5 (2C, C12), 39.3 (2C, C7).

ESI-EM-MS: (*m/z*) required: $[(C_{26}H_{25}IO_6)Na]^+$ = 583.0588; (*m/z*) found: $[(C_{26}H_{25}IO_6)Na]^+$ = 583.0576.

FT-IR (ATR) ($\tilde{\nu}$ = cm^{-1}): 3028 (w), 2952 (w), 1753 (m), 1729 (m), 1602 (w), 1589 (w), 1575 (w), 1497 (m), 1462 (m), 1434 (m), 1361 (w), 1342 (w), 1291 (m), 1246 (m), 1212 (m), 1195 (m), 2275 (m), 1099 (s), 1052 (w), 1023 (m), 1001 (m), 966 (m), 930 (w), 917 (w), 904 (w), 890 (w), 865 (w), 829 (w), 755 (s), 737 (m), 694 (s), 650 (m).

(2*R*,2'*R*)-2,2'-((2-Iodo-1,3-phenylene)bis(oxy))bis(3-phenylpropanoic acid) (**S13**)

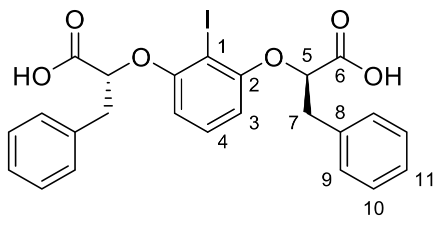

Prepared according to a modified literature procedure.[10] **S12** (1.12 g, 2.0 mmol, 1.0 equiv.) was dissolved in a mixture of THF (8 mL) and MeOH (8 mL) and an aqueous solution of NaOH (2 M, 5.5 mL, 11.0 mmol, 5.5 equiv.) was added. After stirring the mixture vigorously at room temperature for 4 h, TLC (CyH:EtOAc 5:1) indicated full conversion. The reaction mixture was acidified with aqueous HCl (5%) to pH ≈ 2 and was extracted with EtOAc (3 x 50 mL). The combined organic layers were dried over Na_2SO_4 and concentrated under reduced pressure to afford the desired product as an off-white solid (1.1 g, quant.) which was used without further purification.

M.p.: 162 – 163 °C.

$[\alpha]_D^{25}$: +75.3° (*c* = 1.00, acetone).

^{1}H NMR (599 MHz, DMSO-*d*$_6$): δ 13.19 (br s, 2H, H-O), 7.45 (m, 4H, H-C9), 7.29 (m, 4H, H-C10), 7.22 (tt, $^3J_{HH}$ = 7.2 Hz, $^4J_{HH}$ = 1.2 Hz, 2H, H-C11), 7.14 (t, $^3J_{HH}$ = 8.3 Hz, 1H, H-C4), 6.35 (d, $^3J_{HH}$ = 8.4 Hz, 2H, H-C3),

4.94 (dd, $^3J_{HH}$ = 8.2, 4.1 Hz, 2H, H-C5), 3.25 (dd, $^2J_{HH}$ = 14.1, $^3J_{HH}$ = 4.1 Hz, 2H, H-C7), 3.19 (dd, $^2J_{HH}$ = 14.1, $^3J_{HH}$ = 8.1 Hz, 2H, H-C7).

^{13}C{^{1}H} NMR (151 MHz, DMSO-d_6): δ 171.2 (2C, C6), 157.6 (2C, C2), 136.6 (2C, C8), 129.8 (4C, C9), 129.5 (1C, C4), 128.1 (4C, C10), 126.6 (2C, C11), 105.4 (2C, C3), 78.5 (1C, C1), 77.4 (2C, C5), 38.2 (2C, C7).

ESI-EM-MS: (*m/z*) required: [($C_{24}H_{21}IO_6$)Na]$^+$ = 555.0275; (*m/z*) found: [($C_{24}H_{21}IO_6$)Na]$^+$ = 555.0274.

FT-IR (ATR) ($\tilde{\nu}$ = cm^{-1}): 3030 (w), 2537 (w), 1705 (m), 1583 (m), 1497 (m), 1458 (s), 1327 (w), 1292 (w), 1248 (m), 1193 (m), 1104 (s), 1023 (m), 940 (m), 858 (w), 808 (w), 749 (m), 697 (s).

(2*R*,2'*R*)-2,2'-((2-Iodo-1,3-phenylene)bis(oxy))bis(*N*-methyl-3-phenylpropanamide) (**7**)

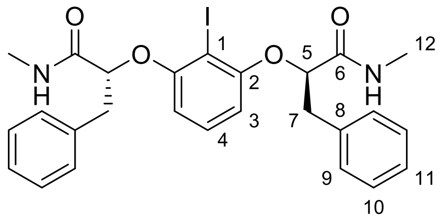

A suspension of **S12** (1.07 g, 2.0 mmol, 1.0 equiv.) in $MeNH_2$ (≈ 8 M in EtOH, 4.0 mL, 32.0 mmol, 16.0 equiv.) was stirred at ambient temperature for 12 h. Purification by column chromatography on silica gel (CyH:EtOAc 1:1 → 1:3, dry load) afforded the desired product as a white solid (924 mg, 83%).

R$_f$ = 0.21 (CyH:EtOAc 1:3).

M.p.: 193 – 194 °C.

$[\alpha]_D^{25}$: +5.2° (*c* = 1.00, $CHCl_3$).

^{1}H NMR (500 MHz, $CDCl_3$): δ 7.33 – 7.29 (m, 4H, H-C9), 7.28 – 7.26 (m, 4H, H-C10), 7.24 – 7.20 (m, 2H, H-C11), 7.10 (t, $^3J_{HH}$ = 8.3 Hz, 1H, H-C4), 6.32 (br q, $^3J_{HH}$ = 4.9 Hz, 2H, NH), 6.30 (d, $^3J_{HH}$ = 8.4 Hz, 2H, H-C3), 4.88 (dd, $^3J_{HH}$ = 6.6, 3.6 Hz, 2H, H-C5), 3.35 (dd, $^2J_{HH}$ = 14.3 Hz, $^3J_{HH}$ = 3.6 Hz, 2H, H-C7), 3.23 (dd, $^2J_{HH}$ = 14.2 Hz, $^3J_{HH}$ = 6.6 Hz, 2H, H-C7), 2.73 (d, $^3J_{HH}$ = 4.9 Hz, 6H, H-C12).

^{13}C{^{1}H} NMR (125 MHz, $CDCl_3$): δ 170.5 (2C, C6), 157.3 (2C, C2), 136.1 (2C, C8), 130.5 (1C, C4), 130.2 (4C, C9), 128.3 (4C, C10), 127.0 (2C, C11), 106.7 (2C, C3), 80.7 (2C, C5), 79.3 (1C, C1), 38.8 (2C, C7), 26.0 (2C, C12).

ESI-EM-MS: (*m/z*) required: [($C_{26}H_{27}IN_2O_4$)Na]$^+$ = 581.0908; (*m/z*) found: [($C_{26}H_{27}IN_2O_4$)Na]$^+$ = 581.0895.

FT-IR (ATR) ($\tilde{\nu}$ = cm^{-1}): 3327 (w), 3031 (w), 2942 (w), 1654 (s), 1571 (m), 1541 (m), 1496 (m), 1452 (s), 1405 (m), 1337 (m), 1271 (w), 1233 (m), 1205 (w), 1188 (m), 1153 (m), 1078 (m), 1057 (s), 1034 (m), 989 (m), 933 (w), 904 (w), 880 (w), 837 (w), 807 (w), 788 (m), 778 (w), 747 (m), 699 (s), 670 (m).

(2*S*,2'*S*)-2,2'-((2-Iodo-1,3-phenylene)bis(oxy))bis(*N*-methyl-3-phenylpropanamide) (***ent*-7**)

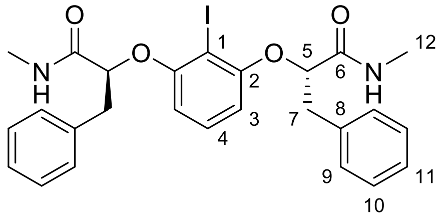

A solution of **S1** (149 mg, 0.63 mmol, 1.0 equiv.), methyl *D*-3-phenyllactate (251 mg, 1.39 mmol, 2.2 equiv.) and PPh_3 (380 mg, 1.45 mmol, 2.3 equiv.) in THF (1.3 mL) under argon was cooled to 0 °C. After dropwise addition of DIAD (0.30 mL, 1.51 mmol, 2.4 equiv.), the ice-bath was removed and the reaction was stirred at room temperature for 15 h. The mixture was evaporated to dryness and purification by column chromatography on silica gel (CyH:EtOAc 5:1) afforded a white solid. This residue was dissolved in EtOH (2.0 mL) and $MeNH_2$ (≈ 8 M in EtOH, 1.17 mL,

9.36 mmol, 16.0 equiv.). After stirring at room temperature overnight, the solvent was removed under reduced pressure and the product was purified by recrystallisation from EtOH (5mL). The resulting white solid was washed with cold EtOH, dissolved in CH_2Cl_2, dried over $MgSO_4$, filtered and evaporated under *vacuo* to afford the desired compound (166 mg, 0.30 mmol, 47% over 2 steps).

R*f* = 0.21 (CyH:EtOAc 1:3).

M.p.: 193 – 194 °C.

$[\alpha]_D^{25}$: -5.6° (*c* = 0.20, $CHCl_3$).

^{1}H NMR (599 MHz, $CDCl_3$): *δ* 7.33 – 7.30 (m, 4H, H-C9), 7.28 – 7.25 (m, 4H, H-C10), 7.24 – 7.20 (m, 2H, H-C11), 7.10 (t, $^3J_{HH}$ = 8.3 Hz, 1H, H-C4), 6.31 (br q, $^3J_{HH}$ = 4.9 Hz, 2H, NH), 6.30 (d, $^3J_{HH}$ = 8.4 Hz, 2H, H-C3), 4.88 (dd, $^3J_{HH}$ = 6.6, 3.6 Hz, 2H, H-C5), 3.35 (dd, $^2J_{HH}$ = 14.3 Hz, $^3J_{HH}$ = 3.6 Hz, 2H, H-C7), 3.24 (dd, $^2J_{HH}$ = 14.2 Hz, $^3J_{HH}$ = 6.6 Hz, 2H, H-C7), 2.73 (d, $^3J_{HH}$ = 4.9 Hz, 6H, H-C12).

^{13}C{^{1}H} NMR (151 MHz, $CDCl_3$): *δ* 170.5 (2C, C6), 157.3 (2C, C2), 136.1 (2C, C8), 130.5 (1C, C4), 130.3 (4C, C9), 128.4 (4C, C10), 127.0 (2C, C11), 106.7 (2C, C3), 80.7 (2C, C5), 79.3 (1C, C1), 38.8 (2C, C7), 26.0 (2C, C12).

ESI-EM-MS: (*m/z*) required: $[(C_{26}H_{27}IN_2O_4)Na]^+$ = 581.0908; (*m/z*) found: $[(C_{26}H_{27}IN_2O_4)Na]^+$ = 581.0917.

(2*R*,2'*R*)-2,2'-((2-iodo-1,3-phenylene)bis(oxy))bis(*N*,*N*-dimethyl-3-phenylpropanamide) (**S14**)

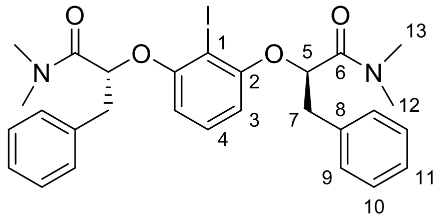

Based on a modified literature procedure.[1] Thionyl chloride (0.2 M) was added to **S13** (106 mg, 0.2 mmol, 1.0 equiv.) and the mixture was heated to 80 °C for 1 h under argon. The solvent was removed under reduced pressure and the residue was co-evaporated with benzene (2x). The obtained solid was dissolved in CH_2Cl_2 (dry, 0.1 M) under argon. The solution was cooled to 0 °C and aqueous Me_2NH (40 wt%, 0.76 mL, 6.0 mmol, 30.0 equiv.) were added slowly. The reaction mixture was then allowed to warm up to ambient temperature and was stirred for 15 h. Purification by column chromatography on silica gel (CyH:EtOAc 1:3, dry load) afforded the desired product as a white solid (102 mg, 87%).

R*f* = 0.29 (CyH:EtOAc 1:3).

M.p.: 197 – 199 °C.

$[\alpha]_D^{25}$: -35.6° (*c* = 1.00, $CHCl_3$).

^{1}H NMR (400 MHz, $CDCl_3$): *δ* 7.46 – 7.41 (m, 4H, H-C9), 7.33 – 7.28 (m, 4H, H-C10), 7.27 – 7.22 (m, 2H, H-C11), 7.06 (t, $^3J_{HH}$ = 8.3 Hz, 1H, H-C4), 6.34 (d, $^3J_{HH}$ = 8.4 Hz, 2H, H-C3), 4.97 (dd, $^3J_{HH}$ = 8.2, 4.6 Hz, 2H, H-C5), 3.37 (dd, $^2J_{HH}$ = 14.0, $^3J_{HH}$ = 8.2 Hz, 2H, H-C7), 3.27 (dd, $^2J_{HH}$ = 14.0, $^3J_{HH}$ = 4.6 Hz, 2H, H-C7), 2.90 (s, 6H, H-C13), 2.87 (s, 6H, H-C12).

^{13}C{^{1}H} NMR (101 MHz, $CDCl_3$): *δ* 169.7 (2C, C6), 157.6 (2C, C2), 136.7 (2C, C8), 130.3 (1C, C4), 130.1 (4C, C9), 128.5 (4C, C10), 127.1 (2C, C11), 106.1 (2C, C3), 81.2 (2C, C5), 78.3 (1C, C1), 38.8 (2C, C7), 36.7 (4C, C12/13).

ESI-MS: (*m/z*) required: $[(C_{28}H_{31}IN_2O_4)Na]^+$ = 609.1221; (*m/z*) found: $[(C_{28}H_{31}IN_2O_4)Na]^+$ = 609.1217.

FT-IR (ATR) ($\tilde{\nu}$ = cm^{-1}): 2926 (w), 1644 (s), 1586 (m), 1568 (m), 1497 (m), 1459 (m), 1398 (m), 1363 (w), 1338 (w), 1293 (w), 1272 (m), 1246 (m), 1206 (m), 1173 (w), 1143 (w), 1092 (s), 1020 (m), 989 (w), 972 (m), 926 (w), 889 (w), 873 (w), 847 (w), 799 (w), 769 (m), 757 (m), 733 (m), 718 (m), 698 (s), 679 (m).

Methyl 4-iodo-3,5-bis(((*R*)-1-(methylamino)-1-oxo-3-phenylpropan-2-yl)oxy)benzoate (**S15**)

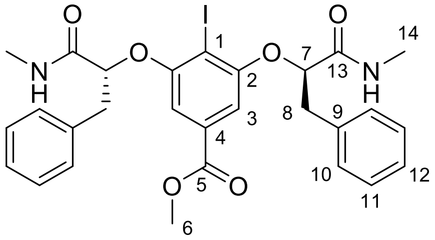

A suspension of **S11** (99 mg, 0.16 mmol, 1.0 equiv.) in EtOH (1 mL) was treated with $MeNH_2$ (≈ 8 M in EtOH, 0.6 mL, 4.80 mmol, 30 equiv.). The reaction mixture was stirred at ambient temperature for 6.5 h. Purification by column chromatography on silica gel (CyHEtOAc 1:1) afforded the desired product as a white solid (45 mg, 46%).

R$_f$ = 0.14 (CyH:EtOAc 1:1).

M.p.: 143 – 145 °C.

$[\boldsymbol{\alpha}]_{\mathbf{D}}^{\mathbf{25}}$: -0.6° (*c* = 0.10, $CHCl_3$).

^{1}H NMR (599 MHz, $CDCl_3$): δ 7.28 – 7.23 (m, 8H, H-C10/H-C11), 7.22 – 7.19 (m, 2H, H-C12), 6.97 (s, 2H, H-C3), 6.32 (q, $^3J_{HH}$ = 4.9 Hz, 2H, H-N), 5.01 (dd, $^3J_{HH}$ = 6.4, 3.7 Hz, 2H, H-C7), 3.87 (s, 3H, H-C6), 3.38 (dd, $^2J_{HH}$ = 14.3 Hz, $^3J_{HH}$ = 3.7 Hz, 2H, H-C8), 3.27 (dd, $^2J_{HH}$ = 14.3 Hz, $^3J_{HH}$ = 6.4 Hz, 2H, H-C8), 2.75 (d, $^3J_{HH}$ = 4.9 Hz, 6H, H-C14).

^{13}C{^{1}H} NMR (151 MHz, $CDCl_3$): δ = 169.9 (2C, C13), 165.6 (1C, C5), 157.2 (2C, C2), 135.8 (2C, C9), 132.8 (1C, C4), 130.2 (4C, C10), 128.4 (4C, C11), 127.1 (2C, C12), 107.2 (2C, C3), 85.8 (1C, C1), 80.6 (2C, C7), 52.8 (1C, C6), 38.6 (2C, C8), 26.1 (2C, C14).

ESI-EM-MS: (*m/z*) requires: $[(C_{28}H_{29}IN_2O_6)Na]^+$ = 639.0963, (*m/z*) found: $[(C_{28}H_{29}IN_2O_6)Na]^+$ = 639.0959.

FT-IR ($\tilde{\nu}$ = cm^{-1}): 3303 (w), 3087 (w), 3031 (w), 2948 (w), 1725 (m), 1660 (s), 1579 (m), 1537 (m), 1497 (w), 1455 (w), 1435 (w), 1416 (s), 1314 (m), 1237 (s), 1202 (w), 1151 (w), 1096 (s), 1012 (m), 914 (w), 862 (w), 760 (s), 702 (s).

(2*R*,2'*R*)-2,2'-((2-Iodo-5-methyl-1,3-phenylene)bis(oxy))bis(*N*-methyl-3-phenyl-propanamide) (S16)

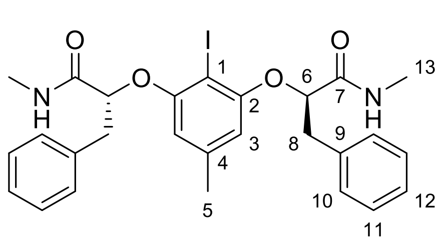

A solution of **S2** (1.25 g, 5.0 mmol, 1.0 equiv.), **S5** (1.89 g, 10.5 mmol, 2.1 equiv.) and PPh_3 (2.89 g, 11.0 mmol, 2.2 equiv.) in THF (dry, 15 mL) under argon was cooled to 0 °C. After dropwise addition of DIAD (2.26 mL, 11.5 mmol, 2.3 equiv.), the ice-bath was removed and the reaction was stirred at room temperature for 96 h. The mixture was evaporated to dryness and the residue was dissolved in $MeNH_2$ (≈ 8 M in EtOH, 25 mL, 200.0 mmol, 40.0 equiv.). After stirring at room temperature for 4 h, the solvent was removed under reduced pressure. Purification by column chromatography on silica gel (CyH:EtOAc 1:1 → 1:3, dry load) afforded the desired product as a white solid (1.24 g, 43% over 2 steps).

R$_f$ = 0.37 (CyH:EtOAc 1:3).

M.p.: 140 – 141 °C.

$[\alpha]_D^{25}$: -0.7° (c = 1.00, $CHCl_3$).

^{1}H NMR (599 MHz, $CDCl_3$): δ 7.33 – 7.30 (m, 4H, H-C10), 7.28 – 7.24 (m, 4H, H-C11), 7.23 – 7.20 (m, 2H, H-C12), 6.32 (q, $^3J_{HH}$ = 5.0 Hz, 2H, N-H), 6.09 (s, 2H, H-C3), 4.85 (dd, $^3J_{HH}$ = 6.8, 3.5 Hz, 2H, H-C6), 3.34 (dd, $^2J_{HH}$ = 14.2, $^3J_{HH}$ =3.5 Hz, 2H, H-C8), 3.22 (dd, $^2J_{HH}$ = 14.2, $^3J_{HH}$ = 6.8 Hz, 2H, H-C8), 2.73 (d, $^3J_{HH}$ = 4.9 Hz, 6H, H-C13), 2.17 (s, 3H, H-C5).

^{13}C{^{1}H} NMR (151 MHz, $CDCl_3$): δ 170.6 (2C, C7), 156.9 (1C, C1), 141.3 (1C, C4), 136.2 (2C, C9), 130.2 (4C, C10), 128.3 (4C, C11), 127.0 (2C, C12), 107.7 (2C, C3), 80.5 (2C, C6), 75.2 (2C, C2), 38.9 (2C, C8), 26.0 (2C, C13), 21.9 (1C, C5).

ESI-EM-MS: (*m/z*) required: $[(C_{27}H_{29}IN_2O_4)Na]^+$ = 595.1064; (*m/z*) found: $[(C_{27}H_{29}IN_2O_4)Na]^+$ = 595.1069.

FT-IR ($\tilde{\nu}$ = cm^{-1}): 3320 (w), 3063 (w), 3030 (w), 2938 (w), 1660 (s), 1578 (m), 1538 (m), 1497 (w), 1454 (m), 1409 (m), 1336 (w), 1224 (w), 1234 (m), 1202 (w), 1155 (w), 1100 (s), 1021 (m), 930 (w), 810 (m), 750 (m), 699 (s), 666 (m).

b. Synthesis of Starting Materials

General Procedure A: Allylation of Phenols

Based on a modified literature procedure.[12] The corresponding phenol (1.0 equiv.) was dissolved in acetone (0.5 M) and K_2CO_3 (1.3 equiv.) and the corresponding allyl bromide derivative (1.1 equiv.) were added. The suspension was heated to 65 °C for the indicated time and filtered after reaching room temperature. The filtrate was concentrated under reduced pressure and the obtained residue was submitted to column chromatography.

1-(Allyloxy)-4-bromobenzene (5)

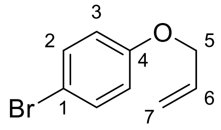

Prepared according to General Procedure **A** from 4-bromophenol (2.60 g, 15.0 mmol, 1.0 equiv.) and allyl bromide (1.43 mL, 16.5 mmol, 1.1 equiv.) in 16 h. Purification by column chromatography on silica gel (*n*-pentane) afforded the desired product as a colourless liquid (2.90 g, 91%).

R_f = 0.32 (*n*-pentane).

^{1}H NMR (300 MHz, $CDCl_3$): δ 7.44 – 7.30 (m, 2H, H-C2), 6.89 – 6.72 (m, 2H, H-C3), 6.03 (ddt, $^3J_{HH}$ = 17.3, 10.5, 5.3 Hz, 1H, H-C6), 5.40 (dq, $^3J_{HH}$ = 17.3, $^2J_{HH}$ = $^4J_{HH}$ = 1.6 Hz, 1H, H-C7), 5.30 (dq, $^3J_{HH}$ = 10.5, $^2J_{HH}$ = $^4J_{HH}$ = 1.4 Hz, 1H, H-C7), 4.51 (dt, $^3J_{HH}$ = 5.3, $^4J_{HH}$ = 1.6 Hz, 2H, H-C5).

^{13}C{^{1}H} NMR (75 MHz, $CDCl_3$): δ 157.8 (1C, C4), 133.0 (1C, C6), 132.4 (2C, C2), 118.1 (1C, C7), 116.7 (2C, C3), 113.1 (1C, C1), 69.1 (1C, C5).

EI-MS: (*m/z*) required: $[(C_9H_9BrO)]^+$ = 212.0; (*m/z*) found: $[(C_9H_9BrO)]^+$ = 212.0.

FT-IR ($\tilde{\nu}$ = cm^{-1}): 3084 (w), 2916 (w), 2870 (w), 1648 (w), 1589 (w), 1578 (w), 1486 (s), 1456 (w), 1424 (w), 1384 (w), 1362 (w), 1284 (m), 1238 (s), 1225 (s), 1171 (m), 1114 (w), 1102 (w), 1072 (m), 1020 (m), 995 (m), 924 (m), 818 (s), 801 (m), 696 (w), 663 (m).

Analytical data in agreement with literature.[13]

1-(Allyloxy)-4-chlorobenzene (**29**)

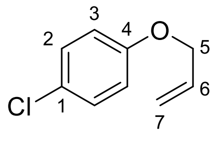

Prepared according to General Procedure **A** from 4-chlorophenol (514 mg, 4.0 mmol, 1.0 equiv.) and allyl bromide (0.38 mL, 4.4 mmol, 1.1 equiv.) in 6.5 h. Purification by column chromatography on silica gel (*n*-pentane) afforded the desired product as a colourless liquid (602 mg, 89%).

R*f* = 0.42 (*n*-pentane).

^{1}H NMR (400 MHz, $CDCl_3$): *δ* 7.26 – 7.19 (m, 2H, H-C2), 6.89 – 6.81 (m, 2H, H-C3), 6.04 (ddt, $^{3}J_{HH}$ = 17.7, 10.6, 5.3 Hz, 1H, H-C6), 5.41 (dt, $^{3}J_{HH}$ = 17.3, $^{2}J_{HH}$ = $^{4}J_{HH}$ = 1.6 Hz, 1H, H-C7), 5.30 (dd, $^{3}J_{HH}$ = 10.5, $^{2}J_{HH}$ = $^{4}J_{HH}$ = 1.9 Hz, 1H, H-C7), 4.51 (dd, $^{3}J_{HH}$ = 5.3, $^{4}J_{HH}$ = 1.8 Hz, 2H, H-C5).

^{13}C{^{1}H} NMR (101 MHz, $CDCl_3$): *δ* 157.3 (1C, C4), 133.1 (1C, C6), 129.4 (2C, C2), 125.8 (1C, C1), 118.0 (1C, C7), 116.2 (2C, C3), 69.2 (1C, C5).

EI-MS: (*m/z*) required: $[(C_9H_9ClO)]^+$ = 168.0; (*m/z*) found: $[(C_9H_9ClO)]^+$ = 168.1.

FT-IR ($\tilde{\nu}$ = cm^{-1}): 2875 (w), 1866 (w), 1649 (w), 1595 (w), 1581 (w), 1489 (s), 1456 (w), 1424 (w), 1363 (w), 1286 (m), 1238 (s), 1225 (s), 1170 (m), 1091 (m), 1020 (m), 1005 (m), 995 (m), 925 (m), 820 (s), 649 (m).

Analytical data in agreement with literature.[14]

1-(Allyloxy)-4-fluorobenzene (**S17**)

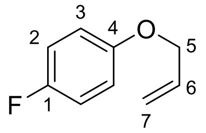

Prepared according to General Procedure **A** from 4-fluorophenol (1.35 g, 12.0 mmol, 1.0 equiv.) and allyl bromide (1.14 ml, 13.2 mmol, 1.1 equiv.) at ambient temperature in 16 h. Purification by column chromatography on silica gel (*n*-pentane) afforded the desired product as a colourless liquid (1.35 g, 74%).

R*f* = 0.20 (*n*-pentane).

^{1}H NMR (400 MHz, $CDCl_3$): *δ* 6.97 (m, 2H, H-C2), 6.86 (m, 2H, H-C3), 6.04 (ddt, $^{3}J_{HH}$ = 17.3, 10.5, 5.3 Hz, 1H, H-C6), 5.41 (dq, $^{3}J_{HH}$ = 17.3 Hz, $^{2}J_{HH}$ = $^{4}J_{HH}$ = 1.6 Hz, 1H, H-C7), 5.29 (dq, $^{3}J_{HH}$ = 10.5 Hz, $^{2}J_{HH}$ = $^{4}J_{HH}$ = 1.4 Hz, 1H, H-C7), 4.50 (dt, $^{3}J_{HH}$ = 5.3 Hz, $^{4}J_{HH}$= 1.5 Hz, 2H, H-C5).

EI-MS: (*m/z*) required: $[(C_9H_9FO)]^+$ = 152.1; (*m/z*) found: $[(C_9H_9FO)]^+$ = 152.1.

Analytical data in agreement with literature.[15]

Methyl 4-(allyloxy)benzoate (**S18**)

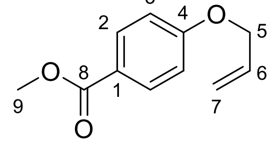

Thionyl chloride (0.37 mL, 5.09 mmol, 1.02 equiv.) was added dropwise to a suspension of 4-hydroxybenzoic acid (691 mg, 5.00 mmol, 1.00 equiv.) in MeOH (15 mL). After heating the mixture to 75 °C for 2.5 h it was evaporated to dryness and the residue was dissolved in acetone (10 mL). Then, K_2CO_3 (898 mg, 6.50 mmol, 1.30 equiv.) and ally bromide (0.48 mL, 5.50 mmol, 1.10 equiv.) were added and the suspension was heated to 70 °C for 4.5 h. The mixture was filtered and the filtrate was concentrated under reduced pressure. Purification by column chromatography on silica gel (CyH:EtOAc 15:1) afforded the desired product as a colourless liquid (882 mg, 92%).

R*f* = 0.30 (CyH:EtOAc 15:1).

^{1}H NMR (400 MHz, $CDCl_3$): δ 8.01 – 7.95 (m, 2H, H-C2), 6.95 – 6.89 (m, 2H, H-C3), 6.04 (ddt, $^3J_{HH}$ = 17.4, 10.5, 5.3 Hz, 1H, H-C6), 5.42 (dq, $^3J_{HH}$ = 17.2, $^2J_{HH}$ = $^4J_{HH}$ =1.6 Hz, 1H, H-C7), 5.31 (dq, $^3J_{HH}$ = 10.5, $^2J_{HH}$ = $^4J_{HH}$ = 1.4 Hz, 1H, H-C7), 4.58 (dq, $^3J_{HH}$ = 5.3, $^4J_{HH}$ = 1.5 Hz, 2H, H-C5), 3.88 (s, 3H, H-C9).

^{13}C{^{1}H} NMR (101 MHz, $CDCl_3$): δ 166.9 (1C, C8), 162.4 (1C, C4), 132.7 (1C, C6), 131.7 (2C, C2), 122.8 (1C, C1), 118.2 (1C, C7), 114.4 (2C, C3), 69.0 (1C, C5), 52.0 (1C, C9).

ESI-EM-MS: (*m/z*) required: $[(C_{11}H_{12}O_3)Na]^+$ = 215.0679; (*m/z*) found: $[(C_{11}H_{12}O_3)Na]^+$ = 215.0681.

FT-IR ($\tilde{\nu}$ = cm^{-1}): 2952 (w), 1713 (s), 1650 (w), 1604 (s), 1579 (w), 1509 (m), 1435 (m), 1363 (w), 1315 (w), 1276 (s), 1247 (s), 1192 (m), 1167 (s), 1103 (s), 1011 (m), 995 (m), 927 (m), 845 (s), 804 (w), 768 (s), 695 (m), 667 (m).

Analytical data in agreement with literature.[16]

Allyl 4-(allyloxy)benzoate (**S19**)

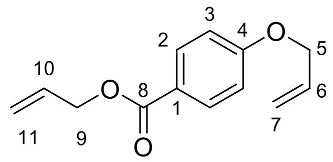

4-Hydroxybenzoic acid (2.0 g, 14.4 mmol, 1.00 equiv.) was dissolved in DMF (20 mL). K_2CO_3 (4.0 g, 28.8 mmol, 2.00 equiv.) and allyl bromide (1.3 mL, 15.0 mmol, 1.05 equiv.) were added. The suspension was heated to 70 °C for 3.5 h and stirred at 40 °C for 14 h. TLC analysis showed unreacted starting material. Allyl bromide (1.0 mL, 11.6 mmol, 0.80 equiv.) was added and the reaction mixture was stirred at 70°C for 1 h. The reaction mixture was diluted with EtOAc and washed with water (5x) and once with brine (1x). The organic layer was dried with $MgSO_4$, filtered and evaporated under *vacuo*. The residue was purified by column chromatography (CyH:EtOAc 9:1) to afford the desired product as a colourless oil (2.94 g, 13.5 mmol, 94%).

R$_f$ = 0.48 (CyH:EtOAc 9:1);

^{1}H NMR (300 MHz, $CDCl_3$): δ 8.01 (m, 2H), 6.93 (m, 2H), 6.03 (m, 2H), 5.44 (dq, *J* = 8.2, 1.6 Hz, 1H), 5.38 (dq, *J* = 8.2, 1.6 Hz, 1H), 5.30 (m, 2H), 4.80 (dt, *J* = 5.6, 1.4 Hz, 2H), 4.59 (dt, *J* = 5.3, 1.5 Hz, 2H).

ESI-EM-MS: (*m/z*) required: $[(C_{13}H_{14}O_3)Na]^+$ = 241.0835; (*m/z*) found: $[(C_{13}H_{14}O_3)Na]^+$ = 241.0834.

Analytical data in agreement with literature.[17]

4-(Allyloxy)benzoic acid (**S20**)

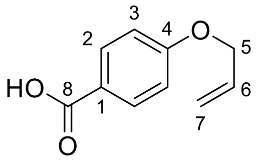

To a mixture of **S19** (1.76 g, 8.0 mmol, 1.0 equiv.) and water (50 mL) was added sodium hydroxide (677 mg, 17.0 mmol, 2.1 equiv.). The resulting mixture was refluxed for 6 h. Upon completion, the reaction mixture was cooled to ambient temperature and extracted with EtOAc. The aqueous layer was then acidified using conc. HCl (pH 0) and extracted with EtOAc (3x). The latter organic layers were combined, dried over $MgSO_4$, filtered and evaporated under *vacuo* to afford the desired product as a white solid (1.36 g, 7.6 mmol, 95%).

M.p.: 162 – 164 °C.

^{1}H NMR (400 MHz, $CDCl_3$): δ 12.17 (brs, 1H, COO-H), 8.06 (m, 2H, H-C2/3), 6.96 (m, 2H, H-C2/3), 6.06 (ddt, $^3J_{HH}$ = 17.3, 10.5, 5.3 Hz, 1H, H-C6), 5.44 (dq, $^3J_{HH}$ = 17.3 Hz, $^2J_{HH}$ = $^4J_{HH}$ = 1.6 Hz, 1H, H-C7), 5.33 (dq, $^3J_{HH}$ = 10.5 Hz, $^2J_{HH}$ = $^4J_{HH}$ = 1.6 Hz, 1H, H-C7), 4.62 (dt, $^3J_{HH}$ = 5.3 Hz, $^4J_{HH}$ = 1.6 Hz, 2H, H-C5).

ESI-EM-MS: (*m/z*) required: $[(C_{10}H_{10}O_3)Na]^+$ = 201.0522; (*m/z*) found: $[(C_{10}H_{10}O_3)Na]^+$ = 201.0524.

Analytical data in agreement with literature.[18]

(4-(Allyloxy)phenyl)(phenyl)methanone (**S21**)

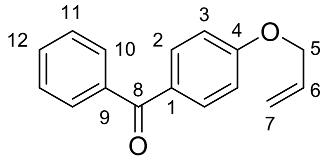

S20 (1.2 g, 6.7 mmol, 1.0 equiv.) was dissolved in thionyl chloride (4 mL) and stirred at 70 °C for 5 h. The reaction mixture was then evaporated under *vacuo* and the residue was dissolved in THF (6 mL) to afford a solution of the corresponding acyl chloride. An aliquot of 2 mL of this solution was cooled to -78 °C (dry ice/acetone). Phenyl magnesiumbromide (2.40 mL, 2.4 mmol, 1.2 equiv., 1 M in THF) was added dropwise to the reaction mixture which was then allowed to slowly warm up to ambient temperature over 16 h. The reaction mixture was quenched with water and extracted with EtOAc (3x). The combined organic layer were dried over $MgSO_4$, filtered and evaporated under *vacuo*. Purification by column chromatography (CyH:EtOAc 19:1) afforded the desired product as a white solid (451 mg, 92%).

R*f* = 0.16 (CyH:EtOAc 19:1).

M.p.: 77 – 78 °C.

^{1}H NMR (500 MHz, $CDCl_3$): δ 7.82 (m, 2H, H-C2), 7.76 (m, 2H, H-C10), 7.56 (m, 1H, H-C12), 7.47 (m, 2H, H-C11), 6.98 (m, 2H, H-C3), 6.07 (ddt, $^3J_{HH}$ = 17.3, 10.5, 5.3 Hz, 1H, H-C6), 5.44 (dq, $^3J_{HH}$ = 17.3 Hz, $^2J_{HH}$ = $^4J_{HH}$ = 1.6 Hz, 1H, H-C7), 5.33 (dq, $^3J_{HH}$ = 10.5 Hz, $^2J_{HH}$ = $^4J_{HH}$ = 1.4 Hz, 1H, H-C7), 4.63 (dt, $^3J_{HH}$ = 5.3 Hz, $^4J_{HH}$ = 1.5 Hz, 2H, H-C5).

^{13}C{^{1}H} NMR (126 MHz, $CDCl_3$): δ 195.7 (1C, C8), 162.4 (1C, C4), 138.4 (1C, C9), 132.7 (2C, C2), 132.7 (1C, C6), 132.0 (1C, C12), 130.4 (1C, C1), 129.9 (2C, C10), 128.3 (2C, C11), 118.3 (1C, C7), 114.4 (2C, C3), 69.07 (1C, C5).

ESI-EM-MS: (*m/z*) required: $[(C_{16}H_{14}O_2)Na]^+$ = 261.0886; (*m/z*) found: $[(C_{16}H_{14}O_2)Na]^+$ = 261.0890.

Analytical data in agreement with literature.[19]

1-(Allyloxy)-3,5-difluorobenzene (**S22**) [20]

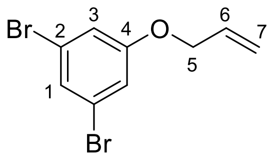

Prepared according to general procedure **A** from 3,5-dibromophenol (504 mg, 2.0 mmol, 1.0 equiv.), K_2CO_3 (415 mg, 3.0 mmol, 1.5 equiv.), allylbromide (190 µL, 2.2 mmol, 1.1 equiv.) and acetone (5 mL) in 16 h at ambient temperature. Purification by column chromatography on silica gel (*n*-pentane:EtOAc 9:1) afforded the desired product as a colorless oil (582 mg, quant.)

R*f* = 0.43 (*n*-pentane).

^{1}H NMR (400 MHz, $CDCl_3$) δ 7.25 (t, $^4J_{HH}$ = 1.6 Hz, 1H, H-C1), 7.01 (d, $^4J_{HH}$ = 1.6 Hz, 2H, H-C3), 6.00 (ddt, $^3J_{HH}$ = 17.3, 10.5, 5.3 Hz, 1H, H-C6), 5.40 (dq, $^3J_{HH}$ = 17.3 Hz, $^2J_{HH}$ = $^4J_{HH}$ = 1.6 Hz, 1H, H-C7), 5.32 (dq, $^3J_{HH}$ = 10.5 Hz, $^2J_{HH}$ = $^4J_{HH}$ = 1.4 Hz, 1H, H-C7), 4.50 (dt, $^3J_{HH}$ = 5.3 Hz, $^4J_{HH}$ = 1.5 Hz, 2H, H-C5).

^{13}C{^{1}H} NMR (101 MHz, $CDCl_3$) δ 159.9 (1C, C4), 132.3 (1C, C6), 126.7 (1C, C4), 123.2 (2C, C2), 118.5 (1C, C7), 117.4 (2C, C3), 69.40 (1C, C5).

EI-EM-MS: (*m/z*) required: $[C_9H_8Br_2O]^{+\bullet}$ = 291.8916; (*m/z*) found: $[C_9H_8Br_2O]^{+\bullet}$ = 291.8918.

FT-IR (ATR) ($\tilde{\nu}$ = cm^{-1}): 3082 (w), 2923 (w), 1650 (w), 1582 (s), 1557 (s), 1436 (m), 1417 (m), 1381 (w), 1361 (w), 1297 (m), 1255 (m), 1224 (m), 1089 (w), 1020 (m), 1003 (m), 987 (m), 926 (m), 892 (m), 858 (m), 827 (s), 743 (s), 667 (m).

1-(Allyloxy)-3,5-dichlorobenzene (**S23**)

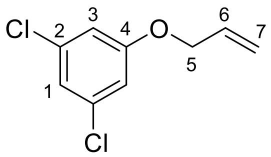

Prepared according to general procedure **A** from 3,5-dichlorophenol (815 mg, 5.0 mmol, 1.0 equiv.), K_2CO_3 (1.04 g, 7.5 mmol, 1.5 equiv.), allylbromide (475 µL, 5.5 mmol, 1.1 equiv.) and acetone (15 mL) in 16 h at ambient temperature. Purification by column chromatography on silica gel (*n*-pentane) afforded the desired product as a colorless oil (900 mg, 89%).

R$_f$ = 0.46 (*n*-pentane).

^{1}H NMR (400 MHz, $CDCl_3$): δ 6.95 (t, $^4J_{HH}$ = 1.8 Hz, 1H, H-C1), 6.81 (d, $^4J_{HH}$ = 1.8 Hz, 2H, H-C3), 6.01 (ddt, $^3J_{HH}$ = 17.3, 10.5, 5.3 Hz, 1H, H-C6), 5.41 (dq, $^3J_{HH}$ = 17.2 Hz, $^2J_{HH}$ = $^4J_{HH}$ = 1.6 Hz, 1H, H-C7), 5.32 (dq, $^3J_{HH}$ = 10.5 Hz, $^2J_{HH}$ = $^4J_{HH}$ = 1.4 Hz, 1H, H-C7), 4.51 (dt, $^3J_{HH}$ = 5.3 Hz, $^4J_{HH}$ = 1.6 Hz, 2H, H-C5).

^{13}C{^{1}H} NMR (151 MHz, $CDCl_3$): δ 159.8 (1C, C4), 135.5 (2C, C2), 132.3 (1C, C6), 121.3 (1C, C1), 118.5 (1C, C7), 114.0 (2C, C3), 69.4 (1C, C5).

EI-EM-MS: (*m/z*) required: $[C_9H_8Cl_2O]^{+\bullet}$ = 201.9947; (*m/z*) found: $[C_9H_8Cl_2O]^{+\bullet}$ = 201.9952.

FT-IR ($\tilde{\nu}$ = cm^{-1}): 3087 (w), 2863 (w), 1650 (w), 1588 (s), 1569 (s), 1442 (m), 1421 (m), 1382 (w), 1362 (s), 1304 (w), 1259 (m), 1239 (w), 1225 (m), 1106 (m), 1092 (m), 1066 (w), 1026 (s), 992 (m), 927 (m), 913 (m), 856 (w), 829 (s), 800 (s), 668 (m).

2-(Allyloxy)-1,4-dichlorobenzene (**S24**)

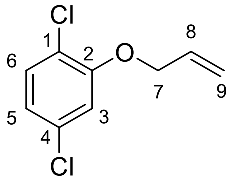

Prepared according to general procedure **A** from 2,5-dichlorophenol (815 mg, 5.0 mmol, 1.0 equiv.), K_2CO_3 (1.04 g, 7.5 mmol, 1.5 equiv.), allylbromide (475 µL, 5.5 mmol, 1.1 equiv.) and acetone (15 mL) in 16 h at ambient temperature. Purification by column chromatography on silica gel (*n*-pentane) afforded the desired product as a colorless oil (956 mg, 4.7 mmol, 94%).

R$_f$ = 0.30 (*n*-pentane).

^{1}H NMR (400 MHz, $CDCl_3$) δ 7.28 (d, $^3J_{HH}$ = 8.2 Hz, 1H, H-C6), 6.90 (d, $^4J_{HH}$ = 2.2 Hz, 1H, H-C3), 6.88 (dd, $^3J_{HH}$ = 8.2 Hz, $^4J_{HH}$ = 2.2 Hz, 1H, H-C5), 6.05 (ddt, $^3J_{HH}$ = 17.3, 10.4, 5.1 Hz, 1H, H-C8), 5.48 (dq, $^3J_{HH}$ = 17.2 Hz, $^2J_{HH}$ = $^4J_{HH}$ = 1.6 Hz, 1H, H-C9), 5.34 (dq, $^3J_{HH}$ = 10.6 Hz, $^2J_{HH}$ = $^4J_{HH}$ = 1.5 Hz, 1H, H-C9), 4.60 (dt, $^3J_{HH}$ = 5.1 Hz, $^4J_{HH}$ = 1.6 Hz, 2H, H-C7).

^{13}C{^{1}H} NMR (151 MHz, $CDCl_3$) δ 154.7 (1C, C2), 133.1 (1C, C4), 132.1 (1C, C8), 130.9 (1C, C6), 121.7 (1C, C1), 121.6 (1C, C5), 118.4 (1C, C9), 114.4 (1C, C3), 70.0 (1C, C7).

EI-EM-MS: (*m/z*) required: $[C_9H_8Cl_2O]^{+\bullet}$ = 201.9947; (*m/z*) found: $[C_9H_8Cl_2O]^{+\bullet}$ = 201.9934.

FT-IR ($\tilde{\nu}$ = cm^{-1}): 3089 (w), 2871 (w), 1864 (w), 1648 (w), 1583 (m), 1478 (s), 1423 (w), 1401 (m), 1381 (w), 1364 (w), 1264 (m), 1236 (m), 1133 (m), 1094 (m), 1059 (m), 998 (m), 927 (m), 895 (m), 867 (w), 836 (m), 800 (m), 725 (w), 708 (w).

1-(Allyloxy)-4-(trifluoromethyl)benzene (**S25**)

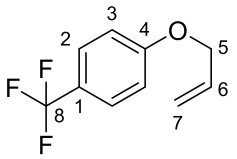

Prepared according to general procedure **A** from 4-trifluoromethylphenol (1.95 g, 12.0 mmol, 1.0 equiv.), K_2CO_3 (2.49 g, 18.0 mmol, 1.5 equiv.), allylbromide (1.14 mL, 13.2 mmol, 1.1 equiv.) and acetone (36 mL) in 20 h at ambient temperature and refluxing for 30 min. Purification by column chromatography on silica gel (*n*-pentane) afforded the desired product as a colorless oil (1.72 g, 8.5 mmol, 71%).

R*f* = 0.35 (*n*-pentane).

^{1}H NMR (300 MHz, $CDCl_3$): *δ* 7.54 (m, 2H, H-C2), 6.98 (m, 2H, H-C3), 6.05 (ddt, $^3J_{HH}$ = 17.3, 10.5, 5.3 Hz, 1H, H-C6), 5.43 (dq, $^3J_{HH}$ = 17.3 Hz, $^2J_{HH}$ = $^4J_{HH}$ = 1.6 Hz, 1H, H-C7), 5.32 (dq, $^3J_{HH}$ = 10.5 Hz, $^2J_{HH}$ = $^4J_{HH}$ = 1.4 Hz, 1H, H-C7), 4.59 (dt, $^3J_{HH}$ = 5.2 Hz, $^4J_{HH}$ = 1.6 Hz, 2H, H-C5).

EI-MS: (*m/z*) required: $[(C_{10}H_9F_3O)]^+$ = 202.1; (*m/z*) found: $[(C_{10}H_9F_3O)]^+$ = 202.1.

Analytical data in agreement with literature.[21]

4-(Allyloxy)benzonitrile (**S26**)

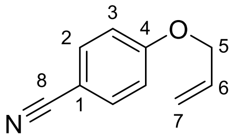

Prepared according to general procedure **A** from 4-hydroxybenzonitrile (1.43 g, 12.0 mmol, 1.0 equiv.), K_2CO_3 (2.49 g, 18.0 mmol, 1.5 equiv.), allylbromide (1.14 mL, 13.2 mmol, 1.1 equiv.) and acetone (36 mL) in 18 h at ambient temperature and refluxing for 2 h. Purification by column chromatography on silica gel (*n*-pentane:EtOAc 4:1) afforded the desired product as a white solid (1.87 g, 11.7 mmol, 98%).

R*f* = 0.83 (*n*-pentane:EtOAc 1:1).

M.p.: 43 – 44 °C.

^{1}H NMR (300 MHz, $CDCl_3$): *δ* 7.57 (m, 2H, H-C2), 6.96 (m, 2H, H-C3), 6.03 (ddt, $^3J_{HH}$ = 17.3, 10.5, 5.3 Hz, 1H, H-C6), 5.41 (dq, $^3J_{HH}$ = 17.3 Hz, $^2J_{HH}$ = $^4J_{HH}$ = 1.6 Hz, 1H, H-C7), 5.33 (dq, $^3J_{HH}$ = 10.5 Hz, $^2J_{HH}$ = $^4J_{HH}$ = 1.4 Hz, 1H, H-C7), 4.58 (dt, $^3J_{HH}$ = 5.3 Hz, $^4J_{HH}$ = 1.5 Hz, 2H, H-C5).

ESI-EM-MS: (*m/z*) required: $[C_{10}H_9NNaO]^+$ = 182.0576; (*m/z*) found: $[C_{10}H_9NNaO]^+$ = 182.0582.

Analytical data in agreement with literature.[21]

4-(Allyloxy)benzaldehyde (**S27**)

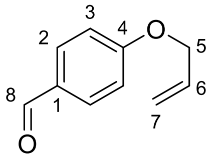

Prepared according to a literature procedure.[22] To a mixture of 4-hydroxybenzaldehyde (545 mg, 4.5 mmol, 1.0 equiv.) and K_2CO_3 (1.23 g, 8.9 mmol, 2.0 equiv.) in DMF (2 mL) was added allylbromide (463 µL, 5.4 mmol, 1.2 equiv.). The reaction mixture was stirred at ambient temperature for 16 h. The reaction mixture was poured onto a saturated solution of ammonium chloride and extracted with EtOAc. The organic layer was then washed with water (4x) and brine (1x), dried over $MgSO_4$, filtered and evaporated under *vacuo*. Purification by column chromatography on silica gel (CyH:EtOAc 9:1) afforded the desired product as a colourless liquid (641 mg, 87%).

R*f* = 0.33 (CyH:EtOAc 9:1).

^{1}H NMR (400 MHz, $CDCl_3$): δ 9.88 (s, 1H, H-C8), 7.83 (m, 2H, H-C2/3), 7.02 (m, 2H, H- C2/3), 6.05 (ddt, $^3J_{HH}$ = 17.3, 10.6, 5.3 Hz, 1H, H-C6), 5.43 (dq, $^3J_{HH}$ = 17.3 Hz, $^2J_{HH}$ = $^4J_{HH}$ = 1.6 Hz, 1H, H-C7), 5.33 (dq, $^3J_{HH}$ = 10.5, $^2J_{HH}$ = $^4J_{HH}$ = 1.4 Hz, 1H, H-C7), 4.63 (dt, $^3J_{HH}$ = 5.3 Hz, $^4J_{HH}$ = 1.5 Hz, 2H, H-C5).

EI-MS: (*m/z*) required: $[(C_{10}H_{10}O_2)]^{+\bullet}$ = 162.1; (*m/z*) found: $[(C_{10}H_{10}O_2)]^{+\bullet}$ = 162.1.

Analytical data in agreement with literature.[23]

4-(Methylthio)phenol (**S28**)

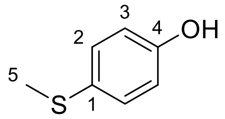

Prepared according to a literature procedure.[24] To a suspension of K_2CO_3 (1.45 g, 10.5 mmol, 1.05 equiv.) in acetone (20 mL) 4-mercaptophenol (1.00 mL, 10.0 mmol, 1.00 equiv.) and methyl iodide (0.69 mL, 11.0 mmol, 1.10 equiv.) were added under argon and the mixture was stirred at room temperature for 72 h. The suspension was filtered over a plug of Celite and the filtrate was concentrated under reduced pressure. Purification by column chromatography on silica gel (CyH:EtOAc 10:1, dry load) afforded the desired product as a white powder (939 mg, 67%).

R*f* = 0.25 (CyH:EtOAc 4:1).

M.p.: 85 – 86 °C.

^{1}H NMR (400 MHz, $CDCl_3$): δ 7.25 – 7.20 (m, 2H, H-C2), 6.81 – 6.76 (m, 2H, H-C3), 5.07 (br, 1H, O-H), 2.44 (s, 3H, H-C5).

^{13}C{^{1}H} NMR (101 MHz, $CDCl_3$): δ 154.1 (1C, C4), 130.5 (1C, C1), 129.0 (2C, C2), 116.2 (2C, C3), 18.2 (1C, C5).

ESI-EM-MS: (*m/z*) required: $[(C_7H_8OS)HCO_2]^-$ = 185.0278; (*m/z*) found: $[(C_7H_8OS)HCO_2]^-$ = 185.0286.

FT-IR (ATR) ($\tilde{\nu}$ = cm^{-1}): 3376 (w), 2918 (w), 1886 (w), 1601 (w), 1588 (w), 1492 (m), 1429 (m), 1362 (w), 1328 (w), 1287 (w), 1245 (m), 1176 (w), 1104 (m), 1010 (w), 977 (w), 956 (m), 817 (m), 721 (w).

Analytical data in agreement with literature.[25]

4-(Methylsulfonyl)phenol (**S29**)

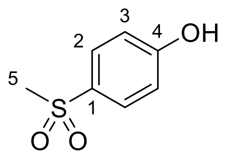

Prepared according to a modified literature procedure.[26] To a solution of **S28** (525 mg, 3.75 mmol, 1.00 equiv.) in MeOH (10 mL) $(NH_4)_6(Mo_7O_{24})\bullet 4H_2O$ (232 mg, 0.19 mmol, 0.05 equiv.) and aqueous H_2O_2 (30 wt%, 1.53 mL, 15.0 mmol, 4.00 equiv.) were added. After stirring at room temperature for 90 min the solvent was removed under reduced pressure and the residue was treated with an aqueous saturated solution of $NaHCO_3$. The mixture was extracted with Et_2O (2x) and the combined organic layers were dried over Na_2SO_4, filtered and concentrated under reduced pressure. The residue was dissolved in boiling Et_2O and then precipitated by addition of *n*-pentane affording the desired product as a white crystalline solid (531 mg, 82%).

M.p.: 94 – 95 °C.

^{1}H NMR (400 MHz, $CDCl_3$): δ 7.83 – 7.72 (m, 2H, H-C2), 7.04 – 6.92 (m, 2H, H-C3), 3.06 (s, 3H, H-C5).

^{13}C{^{1}H} NMR (101 MHz, $CDCl_3$): δ 161.2 (1C, C4), 131.4 (1C, C1), 129.8 (2C, C2), 116.4 (2C, C3), 45.1 (1C, C5).

ESI-EM-MS: (*m/z*) required: $[(C_7H_8O_3S)Na]^+$ = 195.0086; (*m/z*) found: $[(C_7H_8O_3S)Na]^+$ = 195.0068.

FT-IR (ATR) ($\tilde{\nu}$ = cm^{-1}): 3397 (m), 3032 (w), 3012 (w), 2931 (w), 1603 (m), 1586 (m), 1505 (m), 1438 (m), 1405 (w), 1375 (w), 1355 (w), 1328 (w), 1270 (s), 1218 (m), 1175 (w), 1133 (s), 1109 (s), 1086 (s), 1009 (w), 961 (s), 843 (s), 822 (s), 773 (s), 712 (m).

Analytical data in agreement with the literature.[27]

1-(Allyloxy)-4-(methylsulfonyl)benzene (**S30**)

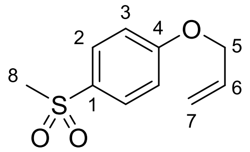

Prepared according to a modified literature procedure.[12] **S29** (241 mg, 1.40 mmol, 1.0 equiv.) was dissolved in DMF (3 mL) and K_2CO_3 (213 mg, 1.54 mmol, 1.1 equiv.) and allyl bromide (0.14 mL, 1.54 mmol, 1.1 equiv.) were added. The suspension was heated to 75 °C for 16 h. After reaching room temperature, the mixture was diluted with H_2O and then extracted with EtOAc (4 x 10 mL). The combined organic layers were washed with brine, dried over Na_2SO_4, filtered and concentrated under reduced pressure. Purification by column chromatography on silica gel (CyH:EtOAc 1:1) afforded the desired product as a white solid (287 mg, 97%).

$\mathbf{R}_f$ = 0.33 (CyH:EtOAc 1:1).

M.p.: 71 – 72 °C.

^{1}H NMR (599 MHz, $CDCl_3$): δ 7.87 – 7.83 (m, 2H, H-C2), 7.04 – 7.01 (m, 2H, H-C3), 6.03 (ddt, $^3J_{HH}$ = 17.3, 10.5 , 5.3 Hz, 1H, H-C6), 5.42 (dq, $^3J_{HH}$ = 17.2, $^2J_{HH}$ = $^4J_{HH}$ = 1.3 Hz, 1H, H-C7), 5.33 (dq, $^3J_{HH}$ = 10.5, $^2J_{HH}$ = $^4J_{HH}$ = 1.3 Hz, 1H, H-C7), 4.61 (dt, $^3J_{HH}$ = 5.3, $^4J_{HH}$ = 1.6 Hz, 2H, H-C5), 3.02 (s, 3H, H-C8).

^{13}C{^{1}H} NMR (151 MHz, $CDCl_3$): δ 162.8 (1C, C4), 132.6 (1C, C1), 132.2 (1C, C6), 129.6 (2C, C2), 118.6 (1C, C7), 115.3 (2C, C3), 69.3 (1C, C5), 45.0 (1C, C8).

ESI-EM-MS: (*m/z*) required: $[(C_{10}H_{12}O_3S)Na]^+$ = 235.0399; (*m/z*) found: $[(C_{10}H_{12}O_3S)Na]^+$ = 235.0400.

FT-IR (ATR) ($\tilde{\nu}$ = cm^{-1}): 3025 (w), 2934 (w), 1591 (m), 1578 (m), 1491 (m), 1465 (w), 1430 (m), 1412 (m), 1372 (w), 1329 (w), 1310 (s), 1289 (s), 1245 (s), 1179 (w), 1138 (s), 1111 (m), 1092 (s), 993 (m), 968 (m), 941 (s), 833 (s), 767 (s), 712 (m), 685 (m).

Analytical data in agreement with literature.[28]

1-(Allyloxy)-4-nitrobenzene (**S31**)

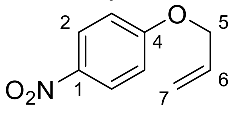

Prepared according to General Procedure **A** from 4-nitrophenol (1.39 g, 10.0 mmol, 1.0 equiv.) and allyl bromide (0.95 ml, 11.0 mmol, 1.1 equiv.) in 6 h. Purification by column chromatography on silica gel (CyH:EtOAc 5:1) afforded the desired product as a yellow liquid (1.75 g, 98%).

$\mathbf{R}_f$ = 0.59 (CyH:EtOAc 4:1).

^{1}H NMR (400 MHz, $CDCl_3$): δ 8.23 – 8.16 (m, 2H, H-C2), 7.01 – 6.92 (m, 2H, H-C3), 6.04 (ddt, $^3J_{HH}$ = 16.2, 10.6, 5.3 Hz, 1H, H-C6), 5.43 (dt, $^3J_{HH}$ = 17.3, $^2J_{HH}$ = $^4J_{HH}$ = 1.6 Hz, 1H, H-C7), 5.35 (dt, $^3J_{HH}$ = 10.6, $^2J_{HH}$ = $^4J_{HH}$ = 1.9 Hz, 1H, H-C7), 4.63 (dd, $^3J_{HH}$ = 5.3, $^4J_{HH}$ = 1.7 Hz, 2H, H-C5).

^{13}C{^{1}H} NMR (101 MHz, $CDCl_3$): δ 163.7 (1C, C4), 141.7 (1C, C1), 132.0 (1C, C6), 126.0 (2C, C2), 118.8 (1C, C7), 114.8 (2C, C3), 69.5 (1C, C5).

ESI-EM-MS: (*m/z*) required: [($C_9H_9NO_3$)Na]$^+$ = 202.0474; (*m/z*) found: [($C_9H_9NO_3$)Na]$^+$ = 202.0472.

FT-IR ($\tilde{\nu}$ = cm^{-1}): 3086 (w), 2931 (w), 1649 (w), 1607 (m), 1590 (s), 1508 (s), 1494 (s), 1456 (w), 1423 (w), 1338 (s), 1330 (s), 1298 (m), 1255 (s), 1233 (s), 1172 (m), 1110 (s), 990 (s), 929 (m), 861 (m), 842 (s), 786 (w), 751 (s), 689 (m), 672 (m).

Analytical data in agreement with literature.[13]

4-(Allyloxy)phenol (**S32**)

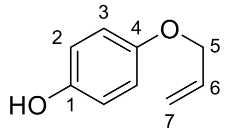

Prepared according to general procedure **A** from hydroquinone (2.20 g, 20 mmol, 4.0 equiv.), K_2CO_3 (0.69 g, 5.0 mmol, 1.0 equiv.), allylbromide (605 µL, 5.0 mmol, 1.0 equiv.) and acetone (60 mL) refluxing for 2 h. Purification by column chromatography on silica gel (CyH:EtOAc 9:1) afforded the desired product as an orange solid (0.23 g, 31%).

R*f* = 0.18 (CyH:EtOAc 9:1).

M.p.: 38 – 40 °C.

^{1}H NMR (400 MHz, $CDCl_3$): δ 6.81 (m, 2H, H-C2/3), 6.76 (m, 2H, H-C2/3), 6.04 (ddt, $^3J_{HH}$ = 17.3, 10.6, 5.3 Hz, 1H, H-C6), 5.40 (dq, $^3J_{HH}$ = 17.3 Hz, $^2J_{HH}$ = $^4J_{HH}$ = 1.6 Hz, 1H, H-C7), 5.27 (dq, $^3J_{HH}$ = 10.5 Hz, $^2J_{HH}$ = $^4J_{HH}$ = 1.4 Hz, 1H, H-C7), 4.48 (dt, $^3J_{HH}$ = 5.3 Hz, $^4J_{HH}$ = 1.5 Hz, 2H, H-C5), 4.45 (s, 1H, H-O).

EI-MS: (*m/z*) required: [($C_9H_{10}O_2$)]$^+$ = 150.1; (*m/z*) found: [($C_9H_{10}O_2$)]$^+$ = 150.1.

FT-IR (ATR) ($\tilde{\nu}$ = cm^{-1}): 3346 (s), 3019 (w), 2884 (w), 1857 (w), 1735 (w), 1648 (w), 1605 (w), 1506 (s), 1447 (m), 1408 (w), 1368 (w), 1297 (w), 1206 (s), 1111 (m), 1022 (m), 991 (m), 929 (m), 919 (m), 825 (s), 809 (m), 776 (m), 735 (w).

Analytical data in agreement with literature.[29]

4-(Allyloxy)phenyl methanesulfonate (**S33**)

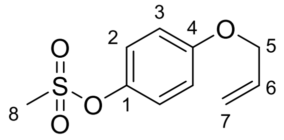

Prepared according to a modified literature procedure,[30] a solution of **S32** (200 mg, 1.3 mmol) and triethylamine (362 µL, 2.6 mmol, 2.0 equiv.) in EtOAc (1 mL) was cooled to 0°C and treated with methanesulfonyl chloride (126 µL, 1.6 mmol, 1.25 equiv.). The resulting reaction mixture was stirred at ambient temperature for 2 h. Upon completion, the reaction was quenched with water and extracted with EtOAc (3x), The combined organic layers were dried over $MgSO_4$, filtered and evaporated under *vacuo*. Purification by column chromatography on silica gel (CyH/EtOAc 7:3) afforded the desired product as a yellow oil (293 mg, 99%).

R*f* = 0.37 (CyH:EtOAc 7:3).

^{1}H NMR (599 MHz, $CDCl_3$): δ 7.19 (m, 2H, H-C2), 6.92 (m, 2H, H-C3), 6.03 (ddt, $^3J_{HH}$ = 17.2, 10.5, 5.3 Hz, 1H, H-C6), 5.41 (dq, $^3J_{HH}$ = 17.2 Hz, $^2J_{HH}$ = $^4J_{HH}$ = 1.6 Hz, 1H, H-C7), 5.30 (dq, $^3J_{HH}$ = 10.5 Hz, $^2J_{HH}$ = $^4J_{HH}$ = 1.4 Hz, 1H, H-C7), 4.53 (dt, $^3J_{HH}$ = 5.3 Hz, $^4J_{HH}$ = 1.6 Hz, 2H, H-C5), 3.11 (s, 3H, H-C8).

^{13}C{^{1}H} NMR (151 MHz, $CDCl_3$): δ 157.7 (1C, C4), 142.9 (1C, C1), 133.0 (1C, C6), 123.2 (2C, C2), 118.1 (1C, C7), 115.9 (2C, C3), 69.4 (1C, C5), 37.2 (1C, C8).

ESI-EM-MS: (*m/z*) required: [($C_{10}H_{12}O_4S$)Na]$^+$ = 251.0349; (*m/z*) found: [($C_{10}H_{12}O_4S$)Na]$^+$ = 251.0362.

FT-IR (ATR) ($\tilde{\nu}$ = cm^{-1}): 3028 (w), 2939 (w), 1875 (w), 1649 (w), 1594 (w), 1498 (s), 1461 (w), 1427 (w), 1361 (m), 1331 (m), 1297 (w), 1246 (s), 1194 (m), 1166 (s), 1148 (s), 1106 (w), 996 (m), 968 (m), 931 (m), 867 (s), 837 (s), 824 (s), 804 (s), 781 (m), 722 (w), 694 (w).

1-Bromo-4-((2-methylallyl)oxy)benzene (S34)

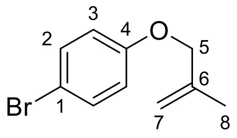

Prepared according to General Procedure **A** from 4-bromophenol (865 mg, 5.0 mmol, 1.0 equiv.) and 3-bromo-2-methylprop-1-ene (0.56 mL, 5.5 mmol, 1.1 equiv.) in 6.5 h. Purification by column chromatography on silica gel (*n*-pentane) afforded the desired product as a colourless liquid (1.05 g, 92%).

$\mathbf{R_f}$ = 0.36 (*n*-pentane).

^{1}H NMR (400 MHz, $CDCl_3$): δ 7.40 – 7.33 (m, 2H, H-C2), 6.84 – 6.78 (m, 2H, H-C3), 5.08 (s, 1H, H-C7), 5.00 (s, 1H, H-C7), 4.40 (s, 2H, H-C5), 1.82 (s, 3H, H-C8).

^{13}C{^{1}H} NMR (101 MHz, $CDCl_3$): δ 158.0 (1C, C4), 140.7 (1C, C6), 132.3 (2C, C2), 116.8 (2C, C3), 113.10 (1C, C7), 113.08 (1C, C1), 72.1 (1C, C5), 19.5 (1C, C8).

EI-MS: (*m/z*) required: $[(C_{10}H_{11}BrO)]^+$ = 226.0; (*m/z*) found: $[(C_{10}H_{11}BrO)]^+$ = 226.0.

FT-IR (ATR) ($\tilde{\nu}$ = cm^{-1}): 3078 (w), 2977 (w), 2916 (w), 2859 (w), 1660 (w), 1589 (w), 1578 (w), 1486 (s), 1453 (m), 1404 (w), 1376 (w), 1283 (m), 1238 (s), 1220 (s), 1170 (m), 1113 (w), 1102 (w), 1073 (m), 1054 (m), 1015 (m), 1001 (m), 967 (w), 900 (w), 855 (w), 818 (s), 800 (m), 695 (w).

Analytical data in agreement with literature.[31]

Allyl(4-bromophenyl)sulfane (24)

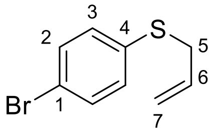

Prepared according to general procedure **A** from 4-bromothiophenol (945 mg, 5.0 mmol, 1.0 equiv.), K_2CO_3 (1.04 g, 7.5 mmol, 1.5 equiv.), allylbromide (475 µL, 5.5 mmol, 1.1 equiv.) and acetone (15 mL) in 16 h from 0 °C to ambient temperature. Purification by column chromatography on silica gel (CyH) afforded the desired product as a colorless oil (1.04 g, 4.5 mmol, 90%).

$\mathbf{R_f}$ = 0.47 (CyH).

^{1}H NMR (300 MHz, $CDCl_3$): δ 7.39 (m, 2H, H-C2/3), 7.20 (m, 2H, H-C2/3), 5.85 (ddt, $^3J_{HH}$ = 16.9, 10.0, 6.8 Hz, 1H, H-C6), 5.11 (m, 2H, H-C7), 3.52 (dt, $^3J_{HH}$ = 6.9 Hz, J_{HH} = 1.2 Hz, 2H, H-C5).

EI-MS: (*m/z*) required: $[(C_9H_9BrS)]^{+\bullet}$ = 227.96; (*m/z*) found: $[(C_{10}H_{10}O_2)]^{+\bullet}$ = 227.95.

Analytical data in agreement with literature.[32]

N-(4-Bromophenyl)-4-methylbenzenesulfonamide (S35)

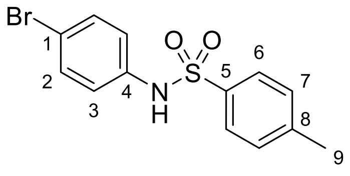

Prepared according to a modified literature procedure.[10] Tosyl chloride (953 mg, 5.0 mmol, 1.0 equiv.) was added to a solution of 4-bromoaniline (860 mg, 5.0 mmol, 1.0 equiv.) and pyridine (0.85 mL, 10.5 mmol, 2.1 equiv.) in CH_2Cl_2 (25 mL) at 0 °C. After stirring at 0 °C for 30 min, the ice-bath was removed, and the reaction was stirred at

room temperature for 3 h. The reaction was quenched with aqueous HCl (0.1 M, 25 mL) and the aqueous layer was extracted with CH_2Cl_2 (3 x 25 mL). The combined organic layers were dried over Na_2SO_4 and were concentrated under reduced pressure. Purification by column chromatography on silica gel (CyH:EtOAc 4:1) afforeded the desired product as an off-white solid (1.57 g, 96%).

R*f* = 0.53 (CyH:EtOAc 2:1).

M.p.: 101 – 102 °C.

^{1}H NMR (400 MHz, $CDCl_3$): *δ* 7.70 – 7.65 (m, 2H, H-C6), 7.36 – 7.28 (m, 3H, H-C2, N-H), 7.25 – 7.21 (m, 2H, H-C7), 7.01 – 6.95 (m, 2H, H-C3), 2.38 (s, 3H, H-C9).

^{13}C{^{1}H} NMR (101 MHz, $CDCl_3$): *δ* 144.4 (1C, C8), 135.8 (1C, C4), 135.7 (1C, C5), 132.5 (2C, C2), 129.9 (2C, C7), 127.4 (2C, C6), 123.1 (2C, C3), 118.6 (1C, C1), 21.7 (1C, C9).

ESI-EM-MS: (*m/z*) required: $[(C_{13}H_{12}BrNO_2S)Na]^+$ = 349.9644; (*m/z*) found: $[(C_{13}H_{12}BrNO_2S)Na]^+$ = 349.9674.

FT-IR (ATR) ($\tilde{\nu}$ = cm^{-1}): 3244 (m), 3101 (w), 1597 (w), 1484 (m), 1335 (m), 1377 (m), 1332 (s), 1305 (w), 1290 (m), 1265 (w), 1222 (m), 1186 (w), 1176 (w), 1161 (s), 1119 (w), 1090 (m), 1069 (m), 1020 (w), 1001 (m), 968 (w), 937 (w), 903 (m), 851 (w), 828 (w), 813 (m), 797 (m), 710 (s), 682 (s), 658 (s).

Analytical data in agreement with literature.[10]

***N*-Allyl-*N*-(4-bromophenyl)-4-methylbenzenesulfonamide** (**26**)

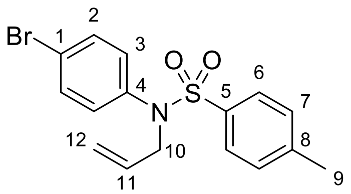

Prepared according to General Procedure **A** from **S35** (652 g, 2.0 mmol, 1.0 equiv.) and allyl bromide (0.19 ml, 2.2 mmol, 1.1 equiv.) in 8 h. Purification by column chromatography on silica gel (CyH:EtOAc 4:1) afforded the desired product as a colourless liquid (232 mg, 93%) which solidified upon standing.

R*f* = 0.58 (CyH:EtOAc 3:1).

M.p.: 66 – 68 °C.

^{1}H NMR (500 MHz, $CDCl_3$): *δ* 7.49 – 7.46 (m, 2H, H-C6), 7.42 – 7.39 (m, 2H, H-C2), 7.28 – 7.24 (m, 2H, H-C7), 6.93 – 6.89 (m, 2H, H-C3), 5.70 (ddt, *J* = 17.7, 9.8, 6.3 Hz, 1H, H-C11), 5.08 – 5.04 (m, 2H, H-C12), 4.14 (dt, *J* = 6.3, 1.4 Hz, 2H, H-C10), 2.43 (s, 3H, H-C9);

^{13}C{^{1}H} NMR (126 MHz, $CDCl_3$): *δ* 143.9 (1C, C8), 138.3 (1C, C4), 135.2 (1C, C5), 132.6 (1C, C11), 132.2 (2C, C2), 130.5 (2C, C3), 129.7 (2C, C7), 127.8 (2C, C6), 121.8 (1C, C1), 119.3 (1C, C12), 53.5 (1C, C10), 21.7 (1C, C9).

ESI-EM-MS: (*m/z*) required: $[(C_{16}H_{16}BrNO_2S)Na]^+$ = 389.9957; (*m/z*) found: $[(C_{16}H_{16}BrNO_2S)Na]^+$ = 389.9965.

FT-IR (ATR) ($\tilde{\nu}$ = cm^{-1}): 1645 (w), 1597 (w), 1485 (m), 1453 (w), 1419 (w), 1400 (w), 1346 (s), 1305 (w), 1291 (w), 1267 (w), 1218 (w), 1185 (w), 1160 (s), 1091 (m), 1071 (m), 1011 (m), 987 (w), 862 (s), 813 (m), 748 (m), 712 (s), 660 (s).

Analytical data in agreement with literature.[33]

2-Allyl-4-chlorophenol (30)

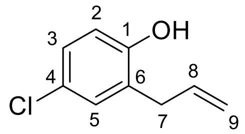

In a *Biotage microwave vial (0.2 - 0.5 mL*) was irradiated neat **27** (400 mg, 2.4 mmol, 1.0 equiv.) at 210 °C for 15 hours in a *Biotage Initiator 2.0*. The reaction mixture was absorbed on silica gel and the residue was purified by silica column chromatography (*n*-pentane:EtOAc 9:1) to afford the desired product as a yellow oil (340 mg, 2.0 mmol, 85%).

$\mathbf{R_f}$ = 0.45 (*n*-pentane:EtOAc 9:1).

^{1}H NMR (599 MHz, $CDCl_3$): δ 7.10 – 7.07 (m, 2H, H-C3, H-C5), 6.74 (dd, J = 8.0, 0.8 Hz, 1H, H-C2), 5.98 (ddt, $^3J_{HH}$ = 17.2, 10.2, 6.4 Hz, 1H, H-C8), 5.19 (dq, $^3J_{HH}$ = 10.2 Hz, $^4J_{HH}$ = 1.6 Hz, 1H, H-C9), 5.17 (dq, $^3J_{HH}$ = 17.2 Hz, $^4J_{HH}$ = 1.6 Hz, 1H), 4.96 (s, 1H, H-O), 3.37 (dt, $^3J_{HH}$ = 6.3 Hz, $^4J_{HH}$ = 1.6 Hz, 2H, H-C7).

^{13}C{^{1}H} NMR (151 MHz, $CDCl_3$): δ 152.8 (1C, C1), 135.6 (1C, C8), 130.2 (1C, C5), 127.7 (1C, C3), 127.3 (1C, C6), 125.7 (1C, C4), 117.3 (1C, C9), 117.2 (1C, C2), 35.0 (1C, C7).

EI-MS: (*m/z*) required: $[(C_9H_9ClO)]^{+\bullet}$ = 168.03; (*m/z*) found: $[(C_9H_9ClO)]^{+\bullet}$ = 168.07.

Analytical data in agreement with literature.[34]

1-Bromo-4-(prop-2-yn-1-yloxy)benzene (S36)

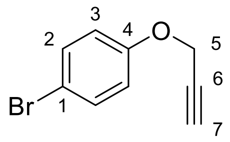

Prepared according to General Procedure **A** from 4-bromophenol (519 mg, 3.0 mmol, 1.0 equiv.), propargyl bromide (0.40 ml, 80% in Toluene, 3.6 mmol, 1.2 equiv.), K_2CO_3 (829 mg, 6.0 mmol, 2 equiv.) in refluxing acetone (9 mL) for 2 h. Purification by column chromatography on silica gel (*n*-pentane) afforded the desired product as a colourless liquid (561 mg, 89%).

$\mathbf{R_f}$ = 0.12 (*n*-pentane).

^{1}H NMR (400 MHz, $CDCl_3$): δ 7.40 (m, 2H), 6.87 (m, 2H), 4.67 (d, $^4J_{HH}$ = 2.4 Hz, 2H, H-C5), 2.53 (t, $^4J_{HH}$ = 2.4 Hz, 1H, H-C7).

EI-MS: (*m/z*) required: $[(C_9H_7BrO)]^{+\bullet}$ = 210.0; (*m/z*) found: $[(C_9H_7BrO)]^{+\bullet}$ = 210.0.

Analytical data in agreement with literature. [35]

1-Bromo-4-((prop-2-yn-1-yl-3-d)oxy)benzene (S37)

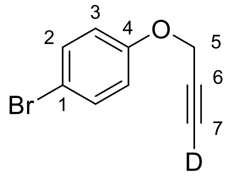

In a flame-dried Schlenk tube was combined **S36** (1.60 g, 7.6 mmol, 1.0 equiv.) and dry THF (15 mL). The resulting solution was cooled to -78°C (dry ice/acetone) and was treated dropwise by a solution of EtMgBr (3M in Et_2O, 5.1 mL, 15.3 mmol, 2.0 equiv.). The reaction mixture was stirred at -78°C for 30 min. and was allowed to warm up to ambient temperature over 1 h. The reaction mixture was then quenched with D_2O and extracted with EtOAc. The combined organic layers were washed with water, dried over $MgSO_4$, filtered and concentrated under reduced pressure to afford the desired product as a colorless oil (1.56 g, 97%, >95% of D-incorporation by ^{1}H NMR).

$\mathbf{R_f}$ = 0.26 (*n*-pentane).

^{1}H NMR (400 MHz, $CDCl_3$): δ 7.40 (m, 2H, H-C2), 6.87 (m, 2H, H-C3), 4.67 (s, 2H, H-C5).

$^{13}C\{^1H\}$ NMR (101 MHz, $CDCl_3$): δ 156.8 (1C, C4), 132.5 (2C, C2), 116.9 (2C, C3), 114.0 (1C, C1), 77.8 (t, $^2J_{CD}$ = 7.5 Hz, 1C, C6), 75.8 (t, $^1J_{CD}$ = 38.7 Hz, 1C, C7), 56.1 (1C, C5).

EI-EM-MS: (*m/z*) required: $[C_9H_6OBrD]^{+\bullet}$ = 210.9738; (*m/z*) found: $[C_9H_6OBrD]^{+\bullet}$ = 210.9738.

FT-IR (ATR) ($\tilde{\nu}$ = cm^{-1}): 2591 (w), 1986 (w), 1579 (w), 1485 (s), 1453 (w), 1405 (w), 1373 (w), 1287 (m), 1259 (w), 1217 (s), 1173 (m), 1115 (w), 1102 (w), 1072 (m), 1024 (s), 1003 (s), 913 (w), 818 (s), 787 (w), 695 (w).

(*Z*)-1-((Allyl-3-d)oxy)-4-bromobenzene (**30**)

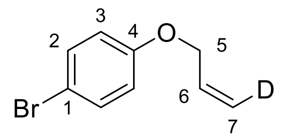

In a flame-dried Schlenk tube was combined bis(cyclopentadienyl) zirconium(IV) chloride hydride (774 mg, 3.0 mmol, 1.2 equiv.) and dry CH_2Cl_2 (6 mL). To this white suspension was added a solution of **S37** (530 mg, 2.5 mmol, 1.0 equiv.) in CH_2Cl_2 (2 mL). The resulting reaction mixture was stirred at ambient temperature for 3 h. Then, water (5 mL) was added and the reaction mixture was stirred at ambient temperature for 16 h. The reaction mixture was extracted with CH_2Cl_2, washed with HCl (1M), a saturated solution of $NaHCO_3$ and brine. The organic layer was dried over $MgSO_4$, filtered and evaporated under reduced pressure. Purification by column chromatography on silica gel (*n*-pentane) afforded the desired product as a colorless oil (106 mg, 19%).

R_f = 0.37 (*n*-pentane).

^1H NMR (300 MHz, $CDCl_3$): δ 7.37 (m, 2H, H-C2), 6.79 (m, 2H, H-C3), 6.02 (dtt, $^3J_{HH}$(cis) = 10.4 Hz, $^3J_{HH}$ = 5.2 Hz, $^3J_{HD}$(trans) = 2.6 Hz, 1H, H-C6), 5.28 (dt, $^3J_{HH}$(cis) = 10.5 Hz, $^4J_{HH}$ = 1.4 Hz, 1H, H-C7), 4.51 (dd, $^3J_{HH}$ = 5.3 Hz, $^4J_{HH}$ = 1.4 Hz, 2H, H-C5).

$^{13}C\{^1H\}$ NMR (101 MHz, $CDCl_3$): δ 157.9 (1C, C4), 132.9 (1C, C6), 132.4 (2C, C2), 117.8 (t, $^1J_{CD}$ = 24.0 Hz, 1C, C7), 116.7 (2C, C3), 113.2 (1C, C1), 69.1 (1C, C5).

EI-EM-MS: (*m/z*) required: $[C_9H_8OBrD]^{+\bullet}$ = 212.9894; (*m/z*) found: $[C_9H_8OBrD]^{+\bullet}$ = 212.9893.

FT-IR (ATR) ($\tilde{\nu}$ = cm^{-1}): 3059 (w), 2919 (w), 2859 (w), 1590 (w), 1578 (w), 1486 (s), 1456 (w), 1405 (w), 1384 (w), 1341 (w), 1285 (m), 1238 (s), 1226 (s), 1171 (m), 1114 (w), 1102 (w), 1071 (m), 1019 (m), 1001 (m), 937 (w), 818 (s), 799 (s), 695 (w).

(*E*)-1-((Allyl-3-d)oxy)-4-bromobenzene (**31**) [36]

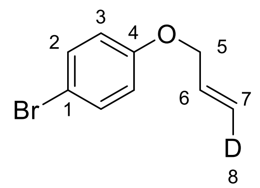

In a flame-dried Schlenk tube was combined 1-bromo-4-(prop-2-yn-1-yloxy)benzene (211 mg, 1.0 mmol, 1.0 equiv.) and dry CH_2Cl_2 (2.0 mL). To this solution was added bis(cyclopentadienyl) zirconium(IV) chloride hydride (309 mg, 1.2 mmol, 1.2 equiv.) in one portion followed by the addition of CH_2Cl_2 (1.0 mL). The resulting reaction mixture was stirred at ambient temperature for 30 min. The reaction mixture was cooled to 0°C (ice/water), quenched with D_2O (0.5 mL) and then stirred at ambient temperature for 1 h. The resulting mixture was extracted with CH_2Cl_2, washed with water, dried over $MgSO_4$, filtered and evaporated under reduced pressure. Purification by column chromatography on silica gel (*n*-pentane) afforded the desired product as a colorless oil (169 mg, 79%, 90% of D-incorporation by ^1H NMR).

R_f = 0.22 (*n*-pentane).

^{1}H NMR (500 MHz, $CDCl_3$): δ 7.37 (m, 2H, H-C2), 6.80 (m, 2H, H-C3), 6.03 (dtt, $^{3}J_{HH}$(trans) = 17.3 Hz, $^{3}J_{HH}$ = 5.3 Hz, $^{3}J_{HD}$(cis) = 1.4 Hz, 1H, H-C6), 5.39 (dt, $^{3}J_{HH}$(trans) = 17.3 Hz, $^{4}J_{HH}$ = 1.7 Hz, 1H, H-C7), 4.51 (dd, $^{3}J_{HH}$ = 5.3 Hz, $^{4}J_{HH}$ = 1.7 Hz, 2H, H-C5).

^{13}C{^{1}H} NMR (126 MHz, $CDCl_3$): δ 157.8 (1C, C4), 132.9 (1C, C6), 132.4 (2C, C2), 117.8 (t, $^{1}J_{CD}$ = 24.9, 24.2 Hz, 1C, C7), 116.7 (2C, C3), 69.1 (1C, C5).

EI-EM-MS: (*m/z*) required: $[C_9H_8OBrD]^{+\bullet}$ = 212.9894; (*m/z*) found: $[C_9H_8OBrD]^{+\bullet}$ = 212.9893.

FT-IR (ATR) ($\tilde{\nu}$ = cm^{-1}): 3041 (w), 2859 (w), 1629 (w), 1589 (w), 1578 (w), 1486 (s), 1459 (w), 1405 (w), 1376 (w), 1284 (m), 1238 (s), 1224 (s), 1171 (m), 1114 (w), 1102 (w), 1071 (m), 1018 (m), 1001 (m), 972 (m), 936 (w), 880 (w), 818 (s), 696 (w).

c. Synthesis of Chromanes

General Procedure B: Based on a modified literature procedure.[37] A Teflon® vessel was charged with the specified allyloxy benzene (0.5 mmol, 1.0 equiv.), catalyst **7** (56 mg, 0.1 mmol, 0.2 equiv.) and CH_2Cl_2 (1.25 mL). Then, the stated amine:HF mixture (1.25 mL) and Selectfluor (266 mg, 0.75 mmol, 1.5 equiv.) were added. The reaction vessel was then sealed with a Teflon® screw cap. After stirring at 350 rpm at room temperature for 24 h, the mixture was poured into an aqueous saturated solution of $NaHCO_3$ (25 mL). Once the gas evolution has ceased the aqueous layer was extracted with CH_2Cl_2 (3 x 25 mL). The combined organic layers were dried over $MgSO_4$, filtered and concentrated under reduced pressure.

6-Bromo-3-fluorochromane (6)

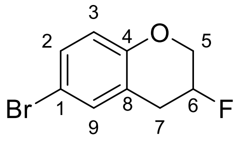

This compound was synthesised from **5** (107 mg, 0.50 mmol) with amine:HF 1:5 according to General Procedure **B**. Purification by column chromatography on silica gel (*n*-pentane/CH_2Cl_2 1:0 → 10:1, dry load) afforded the desired product as a white solid (63 mg, 55%, *e.r.* 13:87).

R*f* = 0.29 (CyH/EtOAc 10:1).

$[\alpha]_D^{25}$: +35.9° (*c* = 1.00, $CHCl_3$ for sample with *e.r.* 13:87).

M.p.: 74 – 75 C.

^{1}H NMR (599 MHz, $CDCl_3$): δ 7.22 (ddt, $^3J_{HH}$ = 8.7 Hz, $^4J_{HH}$ = 2.5 Hz, J = 0.7 Hz, 1H, H-C2), 7.19 (dd, $^4J_{HH}$ = 2.3 Hz, J = 1.1 Hz, 1H, H-C9), 6.75 (d, $^3J_{HH}$ = 8.6 Hz, 1H, H-C3), 5.10 (dqd, $^2J_{FH}$ = 47.3 Hz, $^3J_{HH}$ = 4.1, 1.4 Hz, 1H, H-C6), 4.36 (dddd, $^2J_{HH}$ = 12.1 Hz, $^3J_{FH}$ = 9.1 Hz, $^3J_{HH}$ = 4.0 Hz, $^4J_{HH}$ = 2.9 Hz, 1H, H-C5), 4.09 (ddt, $^3J_{FH}$ = 31.7 Hz, $^2J_{HH}$ = 12.2 Hz, $^4J_{HH}$ = 0.9 Hz, 1H, H-C5), 3.12 (ddt, $^3J_{FH}$ = 34.9 Hz, $^2J_{HH}$ = 17.5 Hz, $^3J_{HH}$ = 4.3 Hz, $^4J_{HH}$ = 0.9 Hz, 1H, H-C7), 3.03 (tt, $^3J_{FH}$ = $^2J_{HH}$ = 17.4 Hz, $^3J_{HH}$ = $^4J_{HH}$ = 3.7 Hz, 1H, H-C7).

^{13}C{^{1}H} NMR (151 MHz, $CDCl_3$): δ 152.9 (1C, C4), 132.6 (1C, C9), 130.9 (1C, C2), 120.4 (d, $^3J_{FC}$ = 1.8 Hz, 1C, C8), 118.6 (1C, C3), 113.3 (1C, C1), 83.7 (d, $^1J_{FC}$ = 175.6 Hz, 1C, C6), 67.2 (d, $^2J_{FC}$ = 21.9 Hz, 1C, C5), 30.5 (d, $^2J_{FC}$ = 23.1 Hz, 1C, C7).

^{19}F NMR (564 MHz, $CDCl_3$): δ -187.18 (ddddd, $^2J_{FH}$ = 47.4 Hz, $^3J_{FH}$ = 34.9, 31.8, 17.9, 9.1 Hz, 1F, F-C6).

EI-EM-MS: (*m/z*) required: $[C_9H_8OFBr]^{+\bullet}$ = 229.9737; (*m/z*) found: $[C_9H_8OFBr]^{+\bullet}$ = 229.9737.

FT-IR (ATR) ($\tilde{\nu}$ = cm^{-1}): 2983 (w), 1677 (w), 1481 (s), 1456 (m), 1432 (w), 1415 (m), 1385 (w), 1350 (w), 1341 (w), 1315 (w), 1292 (w), 1259 (m), 1236 (s), 1198 (w), 1182 (s), 1124 (m), 1088 (m), 1071 (m), 1053 (s), 1002 (m), 944 (s), 903 (w), 876 (m), 862 (m), 830 (m), 814 (m), 802 (s), 738 (m), 707 (w), 666 (w).

The *e.r.* of the product was determined by HPLC analysis using a DAICEL Chiral OJ-H column (5 µm, 250 x 4.6 mm) as the stationary phase and hexanes:*i*-PrOH (99.5:0.5) as eluent system at a flow rate of 0.5 mL·min^{-1}. Detection took place at λ = 230 nm. t_R(minor) = 57.99 min; t_R(major) = 72.69 min.

Analytical data in agreement with literature.[37]

6-Chloro-3-fluorochromane (8)

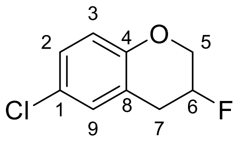

This compound was synthesised from **29** (84 mg, 0.50 mmol) with amine:HF 1:5 according to General Procedure **B**. Purification by column chromatography on silica gel (*n*-pentane/CH_2Cl_2 1:0 → 10:1, dry load) afforded the desired product as a white solid (52 mg, 56%, *e.r.* 11:89).

R*f* = 0.36 (CyH:EtOAc 10:1).

$[\alpha]_D^{25}$: +41.2° (*c* = 1.00, $CHCl_3$ for sample with *e.r.* 11:89).

M.p.: 66 – 68 °C.

^{1}H NMR (599 MHz, $CDCl_3$): *δ* 7.08 (ddt, $^3J_{HH}$ = 8.7 Hz, $^4J_{HH}$ = 2.6 Hz, *J* = 0.8 Hz, 1H, H-C2), 7.04 (dt, $^4J_{HH}$ = 2.3 Hz, *J* = 1.0 Hz, 1H, H-C9), 6.80 (d, $^3J_{HH}$ = 8.7 Hz, 1H, H-C3), 5.10 (ddtd, $^2J_{FH}$ = 47.3 Hz, $^3J_{HH}$ = 4.3, 3.7, 1.6 Hz, 1H, H-C6), 4.37 (dddd, $^2J_{HH}$ = 12.1 Hz, $^3J_{FH}$ = 9.1 Hz, $^3J_{HH}$ = 4.0 Hz, $^4J_{HH}$ = 2.3 Hz, 1H, H-C5), 4.09 (ddtd, $^3J_{FH}$ = 32.0 Hz, $^2J_{HH}$ = 12.1 Hz, $^3J_{HH}$ = 1.6 Hz, $^4J_{HH}$ = 0.9 Hz, 1H, H-C5), 3.10 (dddd, $^3J_{FH}$ = 35.0 Hz, $^2J_{HH}$ = 17.4 Hz, $^3J_{HH}$ = 4.4 Hz, $^4J_{HH}$ = 0.9 Hz, 1H, H-C7), 3.03 (tddd, $^3J_{FH}$ = $^2J_{HH}$ = 17.5 Hz, $^3J_{HH}$ = 4.0 Hz, $^4J_{HH}$ = 2.7 Hz, 0.9 Hz, 1H, H-C7).

^{13}C{^{1}H} NMR (151 MHz, $CDCl_3$): *δ* 152.3 (1C, C4), 129.6 (1C, C9), 128.0 (1C, C2), 126.0 (1C, C1), 119.8 (d, $^3J_{FC}$ = 1.8 Hz, 1C, C8), 118.2 (1C, C3), 83.7 (d, $^1J_{FC}$ = 175.5 Hz, 1C, C6), 67.2 (d, $^2J_{FC}$ = 21.8 Hz, 1C, C5), 30.5 (d, $^2J_{FC}$ = 23.1 Hz, 1C, C7).

^{19}F NMR (564 MHz, $CDCl_3$): *δ* -187.2 (ddddd, $^2J_{FH}$ = 47.3 Hz, $^3J_{FH}$ = 34.9, 32.0, 17.5, 9.1 Hz, 1F, F-C6).

EI-MS: (*m/z*) required: $[(C_9H_8ClFO)]^+$ = 185.9; (*m/z)* found: $[(C_9H_8ClFO)]^+$ = 185.9.

FT-IR (ATR) ($\tilde{\nu}$ = cm^{-1}): 2983 (w), 2951 (w), 2893 (w), 1870 (w), 1746 (w), 1627 (w), 1605 (w), 1579 (w), 1486 (m), 1475 (m), 1457 (m), 1417 (m), 1386 (m), 1351 (m), 1314 (w), 1294 (m), 1259 (m), 1249 (s), 1238 (s), 1201 (m), 1184 (s), 1122 (m), 1088 (m), 1055 (s), 1004 (m), 945 (s), 904 (w), 861 (m), 846 (m), 808 (s), 739 (m).

The *e.r.* of the product was determined by HPLC analysis using a DAICEL Chiral OJ-H column (5 µm, 250 x 4.6 mm) as the stationary phase and hexanes:*i*-PrOH (99.5:0.5) as eluent system at a flow rate of 0.5 mL·min^{-1}. Detection took place at λ = 290 nm. t_R(minor) = 47.00 min; t_R(major) = 55.68 min.

3,6-difluorochromane (9)

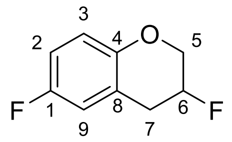

This compound was synthesised from **S17** (76 mg, 0.50 mmol) with amine:HF 1:5 according to General Procedure **B**. Purification by column chromatography on silica gel (*n*-pentane:CH_2Cl_2, 7:3) afforded the desired product as a white solid (38 mg, 44%, *e.r.* 12:88).

R*f* = 0.38 (n-pentane:CH_2Cl_2 7:3).

$[\boldsymbol{\alpha}]_{\mathbf{D}}^{\mathbf{25}}$: +30.8° (*c* = 0.10, $CHCl_3$ for sample with *e.r.* 12:88).

M.p.: 44 – 45 °C.

^{1}H NMR (400 MHz, $CDCl_3$): δ 6.83 (td, $^3J_{HH}$ = $^3J_{FH}$ = 8.5 Hz, $^4J_{HH}$ = 2.9 Hz, 1H, H-C2), 6.81 (dd, $^3J_{HH}$ = 9.0 Hz, $^4J_{FH}$ = 5.2 Hz, 1H, H-C3), 6.77 (dd, $^3J_{FH}$ = 8.8 Hz, $^4J_{HH}$ = 2.9 Hz, 1H, H-C9), 5.10 (dqd, $^2J_{FH}$ = 47.5 Hz, $^3J_{HH}$ = 4.1, 1.6 Hz, 1H, H-C6), 4.35 (dddd, $^2J_{HH}$ = 11.6 Hz, $^3J_{FH}$ = 9.0 Hz, $^3J_{HH}$ = 4.0 Hz, $^4J_{HH}$ = 2.2 Hz, 1H, H-C5), 4.07 (dddt, $^3J_{FH}$ = 31.7 Hz, $^2J_{HH}$ = 12.1 Hz, $^3J_{HH}$ = 1.5 Hz, $^4J_{HH}$ = 0.9 Hz, 1H, H-C5), 3.15 (dtdd, $^3J_{FH}$ = 34.6 Hz, $^2J_{HH}$ = 17.6 Hz, $^3J_{HH}$ = 4.2 Hz, $^4J_{HH}$ = 0.9 Hz, 1H, H-C7), 3.04 (tddd, $^3J_{FH}$ = $^2J_{HH}$ = 17.6 Hz, $^3J_{HH}$ = 3.7 Hz, $^4J_{HH}$ = 2.3, 0.9 Hz, 1H, H-C7).

^{13}C{^{1}H} NMR (151 MHz, $CDCl_3$): δ 157.4 (d, $^1J_{FC}$ = 239.0 Hz, 1C, C1), 149.7 (d, $^4J_{FC}$ = 2.2 Hz, 1C, C4), 119.4 (dd, $^3J_{FC}$ = 7.7, 1.9 Hz, 1C, C8), 117.8 (d, $^3J_{FC}$ = 8.1 Hz, 1C, C3), 115.9 (d, $^2J_{FC}$ = 23.1 Hz, 1C, C9), 114.8 (d, $^2J_{FC}$ = 23.3 Hz, 1C, C2), 83.9 (d, $^1J_{FC}$ = 175.3 Hz, 1C, C6), 67.1 (d, $^2J_{FC}$ = 21.9 Hz, 1C, C5), 30.9 (dd, $^2J_{FC}$ = 23.2 Hz, $^4J_{FC}$ = 1.4 Hz, 1C, C7).

^{19}F NMR (564 MHz, $CDCl_3$): δ -123.2 (td, $^3J_{FH}$ = 8.3 Hz, $^4J_{FH}$ = 5.0 Hz, 1F, F-C1), -187.0 (ddddd, $^2J_{FH}$ = 47.7 Hz, $^3J_{FH}$ = 34.6, 31.8, 18.1, 9.2 Hz, 1F, F-C6).

ESI-EM-MS: (*m/z*) required: $[(C_9H_8OF_2)Na]^+$ = 193.0435; (*m/z*) found: $[(C_9H_8OF_2)Na]^+$ = 193.0441.

FT-IR ($\tilde{\nu}$ = cm^{-1}): 2967 (w), 1734 (w), 1623 (w), 1495 (s), 1455 (w), 1436 (w), 1421 (w), 1393 (w), 1345 (w), 1321 (w), 1303 (w), 1248 (m), 1210 (s), 1196 (s), 1138 (m), 1106 (w), 1091 (m), 1059 (m), 1005 (m), 949 (m), 935 (m), 895 (w), 860 (m), 812 (s), 760 (w), 741 (m), 705 (s).

The *e.r.* of the product was determined by HPLC analysis using a DAICEL Chiral OJ-H column (5 µm, 250 x 4.6 mm) as the stationary phase and hexanes:*i*-PrOH (95:5) as eluent system at a flow rate of 0.5 mL·min^{-1}. Detection took place at λ = 290 nm. t_R(minor) = 21.83 min; t_R(major) = 23.35 min.

5,7-Dibromo-3-fluorochromane (10)

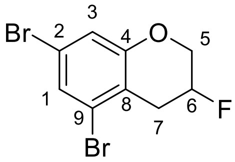

This compound was synthesised from **S22** (146 mg, 0.50 mmol) with amine:HF 1:5 according to General Procedure **B**. Purification by column chromatography on silica gel (*n*-pentane:CH_2Cl_2 1:0 → 99:1) afforded the desired product as a white solid (79 mg, 51%, *e.r.* 7:93).

R*f* = 0.14 (*n*-pentane).

$[\alpha]_D^{27}$: +31.8° (*c* = 0.10, $CHCl_3$ for sample with *e.r.* 7:93).

M.p.: 92 C.

^{1}H NMR (599 MHz, $CDCl_3$): δ 7.34 (d, $^4J_{HH}$ = 1.9 Hz, 1H), 7.03 (d, $^4J_{HH}$ = 1.9 Hz, 1H), 5.14 (dddt, $^2J_{FH}$ = 47.0 Hz, $^3J_{HH}$ = 4.7, 3.3, 1.7 Hz, 1H), 4.38 (dddd, $^2J_{HH}$ = 11.8 Hz, $^3J_{FH}$ = 9.1 Hz, $^3J_{HH}$ = 3.7 Hz, $^4J_{HH}$ = 2.5 Hz, 1H), 4.03 (ddd, $^3J_{FH}$ = 32.4 Hz, $^2J_{HH}$ = 12.1 Hz, $^4J_{HH}$ = 1.3 Hz, 1H), 3.07 (tt, $^2J_{HH}$ = $^3J_{FH}$ = 18.2 Hz, $^4J_{HH}$ = 2.7 Hz, 1H), 2.92 (dddd, $^3J_{FH}$ = 36.5 Hz, $^2J_{HH}$ = 18.1 Hz, $^3J_{HH}$ = 4.7 Hz, $^4J_{HH}$ = 1.4 Hz, 1H).

^{13}C{^{1}H} NMR (151 MHz, $CDCl_3$): δ 155.4 (1C, C4), 127.7 (1C, C1), 125.8 (1C, C9), 120.9 (1C, C2), 119.5 (1C, C3), 118.1 (d, $^3J_{FC}$ = 0.9 Hz, 1C, C8), 83.5 (d, $^1J_{FC}$ = 175.5 Hz, 1C, C6), 67.0 (d, $^2J_{FC}$ = 21.7 Hz, 1C, C7), 31.7 (d, $^2J_{FC}$ = 23.6 Hz, 1C, C5).

^{19}F NMR (564 MHz, $CDCl_3$): δ -185.9 – -186.1 (m, 1F, F-C6).

EI-EM-MS (*m/z*) required: $[(C_9H_7Br_2FO)]^+$ = 307.8842; (*m/z)* found: $[(C_9H_7Br_2FO)]^+$ = 307.8842.

FT-IR (ATR) ($\tilde{\nu}$ = cm^{-1}): 3084 (w), 2998 (w), 1587 (m), 1556 (m), 1462 (m), 1455 (m), 1416 (m), 1404 (m), 1378 (m), 1346 (m), 1321 (w), 1278 (m), 1251 (w), 1231 (m), 1203 (w), 1190 (w), 1180 (w), 1083 (m), 1062 (m), 1008 (m), 951 (m), 897 (m), 875 (m), 862 (m), 855 (m), 845 (m), 805 (s), 790 (s), 692 (w).

The *e.r.* of the product was determined by HPLC analysis using a DAICEL Chiral OJ-H column (5 µm, 250 x 4.6 mm) as the stationary phase and hexanes:*i*-PrOH (99.5:0.5) as eluent system at a flow rate of 0.5 mL·min^{-1}. Detection took place at λ = 290 nm. t_R(minor) = 14.81 min; t_R(major) = 16.20 min.

5,7-Dichloro-3-fluorochromane (11)

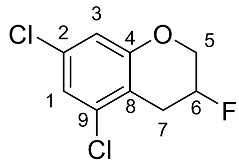

This compound was synthesised from **S23** (101 mg, 0.50 mmol) with amine:HF 1:5 according to General Procedure **B**. Purification by column chromatography on silica gel (*n*-pentane:EtOAc 4:1) afforded the desired product as a white solid (65 mg, 59%, *e.r.* 8:92).

$\mathbf{R}_f$ = 0.17 (*n*-pentane).

$[\alpha]_D^{27}$: +43.9° (c = 0.10, $CHCl_3$ for sample with *e.r.* 8:92).

M.p.: 92 C.

^{1}H NMR (599 MHz, $CDCl_3$): δ 7.02 (d, $^4J_{HH}$ = 2.0 Hz, 1H, H-C1), 6.83 (d, $^4J_{HH}$ = 2.0 Hz, 1H, H-C3), 5.15 (ddddd, $^2J_{FH}$ = 47.0 Hz, $^3J_{HH}$ = 4.6, 3.7, 2.6, 0.9 Hz, 1H, H-C6), 4.40 (dddd, $^2J_{HH}$ = 12.2 , $^3J_{FH}$ = 9.0 Hz, $^3J_{HH}$ = 3.7 Hz, $^4J_{HH}$ = 2.6 Hz, 1H, H-C5), 4.04 (ddq, $^3J_{FH}$ = 32.5 Hz, $^2J_{HH}$ = 12.2 Hz, $^3J_{HH}$ = 0.9 Hz, 1H, H-C5), 3.12 (tt, $^3J_{FH}$ = $^2J_{HH}$ = 18.1 Hz, $^3J_{HH}$ = $^4J_{HH}$ = 2.6 Hz, $^4J_{HH}$ = 0.9 Hz, 1H, H-C7), 2.96 (dddd, $^3J_{FH}$ = 36.3 Hz, $^2J_{HH}$ = 18.1 Hz, $^3J_{HH}$ = 4.7 Hz, $^4J_{HH}$ = 0.9 Hz, 1H, H-C7).

^{13}C{^{1}H} NMR (151 MHz, $CDCl_3$): δ 155.2 (1C, C4), 135.5 (1C, C2), 133.2 (1C, C9), 122.0 (1C, C1), 115.9 (2C, C3, C8), 83.3 (d, $^1J_{FC}$ = 175.5 Hz, 1C, C6), 67.00 (d, $^2J_{FC}$ = 21.6 Hz, 1C, C5), 28.86 (d, $^2J_{FC}$ = 23.6 Hz, 1C, C7).

^{19}F NMR (564 MHz, $CDCl_3$): δ -186.2 (ddddd, $^2J_{FH}$ = 46.9 Hz, $^3J_{FH}$ = 36.4, 32.3, 18.2, 9.0 Hz, 1F, F-C6).

EI-EM-MS: (*m/z*) required: $[C_9H_7Cl_2FO]^{+\bullet}$ = 219.9853; (*m/z*) found: $[C_9H_7Cl_2FO]^{+\bullet}$ = 219.9853.

FT-IR ($\tilde{\nu}$ = cm^{-1}): 3093 (w), 2999 (w), 2950 (w), 1699 (w), 1598 (m), 1571 (s), 1460 (m), 1443 (m), 1421 (m), 1408 (m), 1381 (m), 1349 (m), 1323 (m), 1304 (w), 1283 (m), 1237 (m), 1204 (w), 1188 (m), 1088 (s), 1062 (s), 1015 (s), 976 (w), 954 (s), 905 (m), 878 (m), 846 (s), 832 (s), 807 (s), 695 (m), 668 (m).

The *e.r.* of the product was determined by HPLC analysis using a Reprosil Chiral OM column (10 µm, 250 x 4.6 mm) as the stationary phase and hexanes as eluent system at a flow rate of 0.3 mL·min^{-1}. Detection took place at λ = 210 nm. t_R(minor) = 55.05 min; t_R(major) = 57.51 min.

5,8-Dichloro-3-fluorochromane (12)

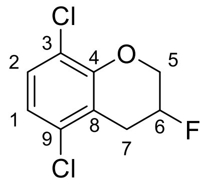

This compound was synthesised from **S24** (102 mg, 0.50 mmol) with amine:HF 1:5 according to General Procedure **B**. Purification by column chromatography on silica gel (*n*-pentane:EtOAc 9:1) afforded the desired product as a white solid (32 mg, 29%, *e.r.* 30:70).

R*f* = 0.09 (*n*-pentane).

$[\alpha]_D^{27}$: +20.9° (*c* = 0.10, $CHCl_3$ for sample with *e.r.* 30:70).

M.p.: 92 °C.

^{1}H NMR (400 MHz, $CDCl_3$): δ 7.19 (d, $^3J_{HH}$ = 8.6 Hz, 1H, H-C2), 6.95 (d, $^3J_{HH}$ = 8.5 Hz, 1H, H-C1), 5.19 (ddddd, $^2J_{FH}$ = 46.9 Hz, $^3J_{HH}$ =4.7, 3.7, 2.6, 0.9 Hz, 1H, H-C6), 4.55 (dddd, $^2J_{HH}$ = 12.1 Hz, $^3J_{FH}$ = 9.1 Hz, $^3J_{HH}$ = 3.7 Hz, $^4J_{HH}$ = 2.6 Hz, 1H, H-C5), 4.13 (ddq, $^3J_{FH}$ = 32.3 Hz, $^2J_{HH}$ = 12.1 Hz, $^3J_{HH}$ = $^4J_{HH}$ = 0.9 Hz, 1H, H-C5), 3.18 (ttd, $^3J_{FH}$ = $^2J_{HH}$ = 18.1 Hz, $^3J_{HH}$ = $^4J_{HH}$ = 2.6 Hz, $^4J_{HH}$ = 0.9 Hz, 1H, H-C7), 3.02 (dddt, $^3J_{FH}$ = 36.4 Hz, $^2J_{HH}$ = 18.1 Hz, $^3J_{HH}$ = 4.7 Hz, $^4J_{HH}$ = 0.9 Hz, 1H, H-C7).

^{13}C{^{1}H} NMR (151 MHz, $CDCl_3$): δ 150.5 (1C, C4), 133.2 (1C, C9), 128.7 (1C, C2), 122.0 (1C, C1), 120.5 (1C, C3), 118.76 (d, $^3J_{FC}$ = 1.1 Hz, 1C, C8), 83.12 (d, $^1J_{FC}$ = 175.7 Hz, 1C, C6), 67.43 (d, $^2J_{FC}$ = 21.8 Hz, 1C, C5), 29.50 (d, $^2J_{FC}$ = 23.7 Hz, 1C, C7).

^{19}F NMR (564 MHz, $CDCl_3$): δ -186.1 (ddddd, $^2J_{FH}$ = 46.9 Hz, $^3J_{FH}$ = 36.4, 32.3, 18.1, 9.1 Hz, 1F, F-C6).

ESI-EM-MS: (*m/z*) required: $[(C_9H_7OCl_2F)_2H_2ONa]^+$ = 480.9714;(*m/z*) found: $[(C_9H_7OCl_2F)_2H_2ONa]^+$ = 480.9715.

FT-IR (ATR) ($\tilde{\nu}$ = cm^{-1}): 3083 (w), 2988 (w), 1589 (m), 1567 (m), 1460 (m), 1435 (m), 1410 (w), 1381 (m), 1357 (w), 1341 (m), 1323 (m), 1305 (w), 1276 (m), 1247 (m), 1204 (w), 1191 (w), 1170 (s), 1132 (w), 1090 (w), 1062 (s), 1007 (m), 955 (s), 915 (m), 891 (m), 870 (m), 834 (m), 798 (s), 780 (m), 719 (w).

The *e.r.* of the product was determined by HPLC analysis using a DAICEL Chiral OJ-H column (5 µm, 250 x 4.6 mm) as the stationary phase and hexanes:*i*-PrOH (99.9:0.1) as eluent system at a flow rate of 1.0 mL·min^{-1}. Detection took place at λ = 210 nm. t_R(major) = 24.16 min; t_R(minor) = 27.42 min.

3-Fluorochroman-6-yl methanesulfonate (13)

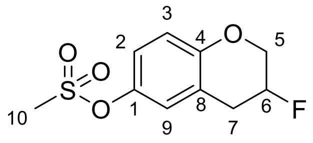

This compound was synthesised from **S33** (114 mg, 0.50 mmol) with amine:HF 1:5 according to General Procedure **B**. Purification by column chromatography on silica gel (*n*-pentane:EtOAc 1:1) afforded a light pink oil (83 mg, 67%, *e.r.* 16:84).

$\mathbf{R}_f$ = 0.57 (*n*-pentane:EtOAc 1:1).

$[\boldsymbol{\alpha}]_{\mathbf{D}}^{\mathbf{27}}$: +26.3° (*c* = 0.10, $CHCl_3$ for sample with *e.r.* 16:84).

^{1}H NMR (599 MHz, $CDCl_3$): δ 7.04 (ddt, $^3J_{HH}$ = 8.5 Hz, $^4J_{HH}$ = 2.9 Hz, J =0.7 Hz, 1H, H-C2), 7.03 (dt, $^4J_{HH}$ = 2.9 Hz, 0.9 Hz, 1H, H-C9), 6.88 (d, $^3J_{HH}$ = 8.5 Hz, 1H, H-C3), 5.12 (dqd, $^2J_{FH}$ = 47.3 Hz, $^3J_{HH}$ = 3.8, 1.3 Hz, 1H, H-C6), 4.40 (dddd, $^2J_{HH}$ = 12.2 Hz, $^3J_{FH}$ = 9.3 Hz, $^3J_{HH}$ = 3.8 Hz, $^4J_{HH}$ = 2.5 Hz, 1H, H-C5), 4.10 (ddtd, $^3J_{FH}$ = 32.3 Hz, $^2J_{HH}$ = 12.3 Hz, $^3J_{HH}$ = $^4J_{HH}$ = 1.2, 0.8 Hz, 1H, H-C5), 3.14 (dddt, $^3J_{FH}$ =35.6 Hz, $^2J_{HH}$ = 17.3 Hz, $^3J_{HH}$ = 3.8 Hz, $^4J_{HH}$ = 0.9 Hz, 1H, H-C7) , 3.11 (s, 3H, H-C10), 3.07 (tdddd, $^2J_{HH}$ = $^3J_{FH}$ = 17.4 Hz, $^3J_{HH}$ = 3.8 Hz, $^4J_{HH}$ = 2.4, 1.2, 0.9 Hz, 1H, H-C7).

^{13}C{^{1}H} NMR (151 MHz, $CDCl_3$): δ 152.7 (1C, C4), 142.9 (1C, C1), 123.5 (1C, C9), 121.6 (1C, C2), 119.7 (d, $^3J_{FC}$ = 1.7 Hz, 1C, C8), 118.1 (1C, C3), 83.5 (d, $^1J_{FC}$ = 175.7 Hz, 1C, C6), 67.2 (d, $^2J_{FC}$ = 21.8 Hz, 1C, C5), 37.3 (1C, C10), 30.7 (d, $^2J_{FC}$ = 23.1 Hz, 1C, C7).

^{19}F NMR (564 MHz, $CDCl_3$): δ -185.80 – -188.45 (m, 1F, F-C6).

ESI-EM-MS: (*m/z*) required: $[(C_{10}H_{11}O_4SF)Na]^+$ = 269.0254; (*m/z)* found: $[(C_{10}H_{11}O_4SF)Na]^+$ = 269.0266.

FT-IR (ATR) ($\tilde{\nu}$ = cm^{-1}): 2926 (w), 1591 (w), 1490 (m), 1433 (w), 1360 (m), 1302 (w), 1257 (m), 1211 (m), 1197 (m), 1170 (s), 1128 (s), 1090 (m), 1061 (m), 1032 (w), 1007 (w), 969 (m), 948 (m), 932 (m), 902 (m), 871 (m), 827 (s), 804 (s), 763 (m), 734 (m),680 (m).

The *e.r.* of the product was determined by HPLC analysis using a DAICEL Chiral OJ-H column (5 µm, 250 x 4.6 mm) as the stationary phase and hexanes:*i*-PrOH (7:3) as eluent system at a flow rate of 1.0 mL·min^{-1}. Detection took place at λ = 230 nm. t_R(minor) = 53.84 min; t_R(major) = 66.31 min.

Methyl 3-fluorochromane-6-carboxylate (14)

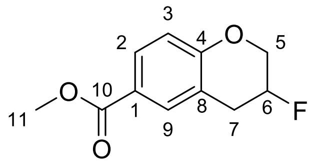

This compound was synthesised from **S18** (96 mg, 0.50 mmol) according to General Procedure **B** with :

- amine:HF 1:5. Purification by column chromatography on silica gel (*n*-pentane:EtOAc 4:1) afforded the desired product as an off-white solid (60 mg, 57%, *e.r.* 10:90).
- amine:HF 1:7.5. Purification by column chromatography on silica gel (*n*-pentane/CH_2Cl_2/acetone 10:1:1) afforded the desired product as an off-white solid (66 mg, 63%, *e.r.* 19:81).

R$_f$ = 0.39 (*n*-pentane/CH_2Cl_2/acetone 10:1:1).

$[\alpha]_D^{26}$: +56.1° (*c* = 0.10, $CHCl_3$ for sample with *e.r.* 10:90).

M.p.: 63 – 65 °C.

^{1}H NMR (500 MHz, $CDCl_3$): δ 7.81 (ddt, $^3J_{HH}$ = 8.5 Hz, $^4J_{HH}$ = 2.1 Hz, J = 0.6 Hz, 1H, H-C2), 7.79 (dt, $^4J_{HH}$ = 2.1, 1.1 Hz, 1H, H-C9), 6.87 (dt, $^3J_{HH}$ = 8.5 Hz, 1H, H-C3), 5.13 (dqd, $^2J_{FH}$ = 47.3 Hz, $^3J_{HH}$ = 3.9, 1.6 Hz, 1H, H-C6), 4.43(dddd, $^2J_{HH}$ = 12.2 Hz, $^3J_{FH}$ = 9.3 Hz, $^3J_{HH}$ = 3.9 Hz, $^4J_{HH}$ = 2.3, 0.6 Hz, 1H, H-C5), 4.16 (dddt, $^3J_{FH}$ = 32.4 Hz, $^2J_{HH}$ = 12.2 Hz, $^3J_{HH}$ = 1.6 Hz, $^4J_{HH}$ = 1.1 Hz, 1H, H-C5), 3.87 (s, 3H, H-C11), 3.14 (dddt, $^3J_{FH}$ = 34.2 Hz, $^2J_{HH}$ = 18.1 Hz, $^3J_{HH}$ = 3.9 Hz, $^4J_{HH}$ = 1.0 Hz, 1H, H-C7), 3.09 (tdddd, $^2J_{HH}$ = $^3J_{FH}$ = 18.1 Hz, $^3J_{HH}$ = 3.9 Hz, $^4J_{HH}$ = 2.5, 1.1, 0.9 Hz, 1H, H-C7).

^{13}C{^{1}H} NMR (126 MHz, $CDCl_3$): δ 166.8 (1C, C10), 157.6 (1C, C4), 132.3 (1C, C9), 129.8 (1C, C2), 123.2 (1C, C1), 118.0 (d, $^3J_{FC}$ = 1.7 Hz, 1C, C8), 116.8 (1C, C3), 83.6 (d, $^1J_{FC}$ = 175.5 Hz, 1C, C6), 67.4 (d, $^2J_{FC}$ = 21.9 Hz, 1C, C5), 52.0 (1C, C11), 30.5 (d, $^2J_{FC}$ = 23.0 Hz, 1C, C7).

^{19}F NMR (470 MHz, $CDCl_3$): δ -187.2 – 187.5 (m, 1F, F-C6).

ESI-EM-MS: (*m/z*) required: $[(C_{11}H_{11}O_3F)Na]^+$ = 233.0584; (*m/z)* found: $[(C_{11}H_{11}O_3F)Na]^+$ = 223.0581.

FT-IR (ATR) ($\tilde{\nu}$ = cm^{-1}): 2956 (w), 1707 (s), 1615 (m), 1583 (m), 1497 (m), 1436 (m), 1395 (w), 1348 (w), 1326 (m), 1311 (m), 1283 (s), 1252 (s), 1197 (s), 1173 (s), 1135 (s), 1108 (m), 1084 (m), 1057 (s), 1004 (w), 988 (m), 941 (m), 897 (m), 876 (m), 854 (m), 836 (m), 817 (s), 783 (w), 769 (s), 740 (m), 708 (m).

The *e.r.* of the product was determined by HPLC analysis using a DAICEL Chiral OJ-H column (5 µm, 250 x 4.6 mm) as the stationary phase and hexanes:*i*-PrOH (9:1) as eluent system at a flow rate of 0.5 mL·min^{-1}. Detection took place at λ = 254 nm. t_R(minor) = 53.58 min; t_R(major) = 66.84 min.

(3-Fluorochroman-6-yl)(phenyl)methanone (15)

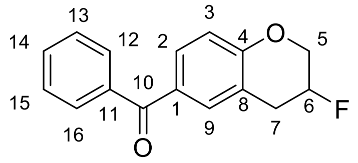

This compound was synthesised from **S21** (119 mg, 0.50 mmol) according to General Procedure **B** with :

- amine:HF 1:5. Purification by column chromatography on silica gel (*n*-pentane:EtOAc 4:1) afforded the desired product as an off-white solid (50 mg, 39%, *e.r.* 11:89).
- amine:HF 1:7.5. Purification by column chromatography on silica gel (*n*-pentane:EtOAc 4:1) afforded the desired product as an off-white solid (71 mg, 55%, *e.r.* 19:81).

R$_f$ = 0.31 (*n*-pentane:EtOAc 4:1).

$[\boldsymbol{\alpha}]_{\mathbf{D}}^{\mathbf{26}}$: +45.6° (*c* = 0.10, $CHCl_3$ for sample with *e.r.* 11:89).

^{1}H NMR (500 MHz, $CDCl_3$): δ 7.76 – 7.73 (m, 2H, H-C12, H-C16), 7.65 – 7.61 (m, 2H, H-C2, H-C9), 7.56 (m, 1H, H-C14), 7.49 – 7.45 (m, 2H, H-C13, H-C15), 6.92 (m, 1H, H-C3), 5.16 (dqd, $^{2}J_{FH}$ = 47.2 Hz, $^{3}J_{HH}$ = 3.8, 1.6 Hz, 1H, H-C6), 4.46 (dddd, $^{2}J_{HH}$ = 12.3 Hz, $^{3}J_{FH}$ = 9.2 Hz, $^{3}J_{HH}$ = 3.9 Hz, $^{4}J_{HH}$ = 2.4, 0.6 Hz, 1H, H-C5), 4.18 (dddt, $^{3}J_{FH}$ = 32.8 Hz, $^{2}J_{HH}$ = 12.3 Hz, $^{3}J_{HH}$ = 1.6 Hz, $^{4}J_{HH}$ = 0.9 Hz, 1H, H-C5), 3.15 (ddddd, $^{3}J_{FH}$ = 34.1 Hz, $^{2}J_{HH}$ = 17.3 Hz, $^{3}J_{HH}$ = 3.8 Hz, $^{4}J_{HH}$ = 0.9, 0.6 Hz, 1H, H-C7), 3.11 (tddd, $^{2}J_{HH}$ = $^{3}J_{FH}$ = 17.2 Hz, $^{3}J_{HH}$ = 3.6 Hz, $^{4}J_{HH}$ = 2.4, 0.9 Hz, 1H, H-C7).

^{13}C{^{1}H} NMR (126 MHz, $CDCl_3$): δ 195.6 (1C, C10), 157.6 (1C, C4), 138.3 (1C, C11), 133.0 (1C, C9), 132.1 (1C, C14), 130.9 (1C, C2), 130.8 (1C, C1), 129.8 (2C, C12, C16), 128.3 (2C, C13, C15), 118.1 (d, $^{3}J_{FC}$ = 1.6 Hz; 1C, C8), 116.6 (1C, C3), 83.6 (d, $^{1}J_{FC}$ = 175.5 Hz, 1C, C6), 67.5 (d, $^{2}J_{FC}$ = 21.8 Hz, 1C, C5), 30.5 (d, $^{2}J_{FC}$ = 22.9 Hz, 1C, C7).

^{19}F NMR (470 MHz, $CDCl_3$): δ -187.3 (m, 1F, F-C6).

ESI-EM-MS: (*m/z*) required: $[(C_{16}H_{13}O_2F)Na]^+$ = 279.0792; (*m/z)* found: $[(C_{16}H_{13}O_2F)Na]^+$ = 279.0807.

FT-IR (ATR) ($\tilde{\nu}$ = cm^{-1}): 2925 (w), 1734 (w), 1649 (m), 1605 (m), 1576 (m), 1497 (m), 1446 (w), 1423 (w), 1389 (w), 1346 (w), 1311 (m), 1280 (m), 1258 (s), 1192 (m), 1177 (m), 1139 (w), 1119 (s), 1090 (m), 1062 (m), 1029 (w), 1003 (m), 977 (w), 961 (w), 946 (m), 869 (m), 833 (m), 816 (m), 806 (m), 792 (m), 736 (s), 700 (s), 682 (m).

The *e.r.* of the product was determined by HPLC analysis using a DAICEL Chiral OJ-H column (5 µm, 250 x 4.6 mm) as the stationary phase and hexanes:*i*-PrOH (9:1) as eluent system at a flow rate of 1.0 mL·min^{-1}. Detection took place at λ = 290 nm. t_R(major) = 27.03 min; t_R(minor) = 29.37 min.

3-Fluoro-6-(trifluoromethyl)chromane (16)

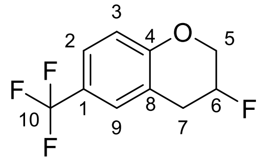

This compound was synthesised from **S25** (101 mg, 0.50 mmol) with amine:HF 1:7.5 according to General Procedure **B**. Purification by column chromatography on silica gel (*n*-pentane:EtOAc 9:1) afforded the desired product as a white solid (63 mg, 57%, *e.r.* 20:80).

R$_f$ = 0.12 (*n*-pentane).

$[\boldsymbol{\alpha}]_{\mathbf{D}}^{\mathbf{27}}$: +30.8° (*c* = 0.10, $CHCl_3$ for sample with *e.r.* 20:80).

M.p.: 39 – 40 °C.

^{1}H NMR (599 MHz, $CDCl_3$): δ 7.39 (ddq, $^3J_{HH}$ = 8.6 Hz, $^4J_{HH}$ = 2.3 Hz, $^4J_{FH}$ = 0.8 Hz, 1H, H-C2), 7.34 (dh, $^4J_{HH}$ = 2.3 Hz, 0.8 Hz, 1H, H-C9), 6.94 (d, $^3J_{HH}$ = 8.6 Hz, 1H, H-C3), 5.15 (ddtd, $^2J_{FH}$ = 47.4 Hz, $^3J_{HH}$ = 4.2, 3.7, 1.2 Hz, 1H, H-C6), 4.44 (dddd, $^2J_{HH}$ = 12.5 Hz, $^3J_{FH}$ = 9.6 Hz, $^3J_{HH}$ = 3.8 Hz, $^4J_{HH}$ = 2.9 Hz, 1H, H-C5), 4.16 (dddt, $^3J_{FH}$ = 32.5 Hz, $^2J_{HH}$ = 12.5 Hz, $^3J_{HH}$ = 1.2 Hz, $^4J_{HH}$ = 0.9 Hz, 1H, H-C5), 3.15 (dddt, $^3J_{FH}$ = 34.6 Hz, $^2J_{HH}$ = 17.3 Hz, $^3J_{HH}$ = 4.2 Hz, $^4J_{HH}$ = 0.9 Hz, 1H, H-C7), 3.10 (tddt, $^3J_{FH}$ = $^2J_{HH}$ = 17.3 Hz, $^3J_{HH}$ = 3.6 Hz, $^4J_{HH}$ = 2.9, 0.9 Hz, 1H, H-C7).

^{13}C{^{1}H} NMR (151 MHz, $CDCl_3$): δ 156.3 (d, $^4J_{FC}$ = 1.0 Hz, 1C, C4), 127.6 (q, $^3J_{FC}$ = 3.8 Hz, 1C, C9), 125.2 (q, $^3J_{FC}$ = 3.7 Hz, 1C, C2), 124.4 (q, $^1J_{FC}$ = 271.2 Hz, 1C, C10), 123.6 (q, $^2J_{FC}$ = 32.7 Hz, 1C, C1), 118.5 (d, $^3J_{FC}$ = 1.7 Hz, 1C, C8), 117.2 (1C, C3), 83.5 (d, $^1J_{FC}$ = 175.7 Hz, 1C, C6), 67.4 (d, $^2J_{FC}$ = 21.8 Hz, 1C, C5), 30.5 (d, $^2J_{FC}$ = 23.1 Hz, 1C, C7).

^{19}F NMR (564 MHz, $CDCl_3$): δ -61.7 (s, 3F, F-C10), -187.3 (ddddd, $^2J_{FH}$ = 47.3 Hz, $^3J_{FH}$ = 34.4, 32.4, 17.7, 9.2 Hz, 1F, F-C6).

EI-EM-MS: (*m/z*) required: $[C_{10}H_8F_4O]^{+\bullet}$ = 220.0506; (*m/z*) found: $[C_{10}H_8F_4O]^{+\bullet}$ = 220.0498.

FT-IR (ATR) ($\tilde{\nu}$ = cm^{-1}): 2987 (w), 1621 (w), 1595 (w), 1508 (m), 1459 (w), 1437 (w), 1424 (w), 1392 (w), 1349 (w), 1325 (s), 1266 (m), 1253 (m), 1199 (w), 1180 (m), 1155 (m), 1127 (m), 1101 (s), 1072 (s), 1056 (s), 1001 (m), 946 (m), 913 (s), 891 (m), 873 (m), 852 (w), 827 (m), 813 (s), 744 (w), 736 (w).

The *e.r.* of the product was determined by HPLC analysis using a DAICEL Chiral OJ-H column (5 µm, 250 x 4.6 mm) as the stationary phase and hexanes:*i*-PrOH (99.5:0.5) as eluent system at a flow rate of 0.5 mL·min^{-1}. Detection took place at λ = 230 nm. t_R(minor) = 31.73 min; t_R(major) = 39.77 min.

3-Fluorochromane-6-carbonitrile (**17**)

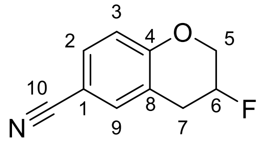

This compound was synthesised from **S26** (80 mg, 0.50 mmol) with amine:HF 1:7.5 according to General Procedure **B**. Purification by column chromatography on silica gel (*n*-pentane:EtOAc 1:1) afforded the desired product as a white solid (57 mg, 54%, *e.r.* 18:82).

R*f* = 0.25 (*n*-pentane:EtOAc 4:1).

$[\boldsymbol{\alpha}]_{\mathbf{D}}^{27}$: +49.9° (*c* = 0.10, $CHCl_3$ for sample with *e.r.* 18:82).

M.p.: 89 – 92 °C.

^{1}H NMR (500 MHz, $CDCl_3$): δ 7.41 (m, 1H, H-C2), 7.37 (dt, $^{4}J_{HH}$ = 2.1 Hz, 0.9 Hz, 1H, H-C9), 6.91 (d, $^{3}J_{HH}$ = 8.5 Hz, 1H, H-C3), 5.15 (ddddd, $^{2}J_{FH}$ = 47.0 Hz, $^{3}J_{HH}$ = 4.1, 3.8, 3.4, 1.5 Hz, 1H, H-C6), 4.47 (dddd, $^{2}J_{HH}$ = 12.4 Hz, $^{3}J_{FH}$ = 9.4 Hz, $^{3}J_{HH}$ = 3.8 Hz, $^{4}J_{HH}$ = 3.3 Hz, 1H, H-C5), 4.16 (ddq, $^{3}J_{FH}$ = 33.5 Hz, $^{2}J_{HH}$ = 12.4 Hz, $^{3}J_{HH}$ = $^{4}J_{HH}$ = 1.4 Hz, 1H, H-C5), 3.11 (ddddd, $^{3}J_{FH}$ = 34.3 Hz, $^{2}J_{HH}$ = 17.1 Hz, $^{3}J_{HH}$ = 4.1 Hz, $^{4}J_{HH}$ = 1.4, 0.9 Hz, 1H, H-C7), 3.07 (ttdd, $^{3}J_{FH}$ = $^{2}J_{HH}$ = 17.1 Hz, $^{3}J_{HH}$ = $^{4}J_{HH}$ = 3.3 Hz, $^{4}J_{HH}$ = 1.4, 0.9 Hz, 1H, H-C7).

^{13}C{^{1}H} NMR (151 MHz, $CDCl_3$): δ 157.3 (1C, C4), 134.6 (1C, C9), 132.1 (1C, C2), 119.4 (d, $^{3}J_{FC}$ = 1.3 Hz, 1C, C8), 119.1 (1C, C10), 117.9 (1C, C3), 104.6 (1C, C1), 83.1 (d, $^{1}J_{FC}$ = 176.1 Hz, 1C, C6), 67.5 (d, $^{2}J_{FC}$ = 21.6 Hz, 1C, C5), 30.2 (d, $^{2}J_{FC}$ = 23.1 Hz, 1C, C7).

^{19}F NMR (564 MHz, $CDCl_3$): δ -187.34 (d, $^{2}J_{FH}$ = 47.1 Hz, $^{3}J_{FH}$ = 34.3, 33.5, 17.1, 9.6 Hz, 1F, F-C6).

ESI-EM-MS: (*m/z*) required: $[(C_{10}H_8NOF)Na]^+$ = 200.0482; (*m/z)* found: $[(C_{10}H_8NOF)Na]^+$ = 200.0484.

FT-IR ($\tilde{\nu}$ = cm^{-1}): 2990 (w), 2943 (w), 2225 (m), 1607 (m), 1576 (m), 1495 (m), 1454 (w), 1418 (w), 1395 (w), 1349 (w), 1308 (m), 1269 (m), 1253 (s), 1224 (m), 1204 (w), 1175 (w), 1130 (m), 1091 (m), 1057 (m), 1002 (m), 940 (m), 921 (w), 910 (m), 875 (w) 857 (m), 840 (m), 817 (s), 743 (w), 723 (w), 676 (w).

The *e.r.* of the product was determined by HPLC analysis using a DAICEL Chiral OJ-H column (5 µm, 250 x 4.6 mm) as the stationary phase and hexanes:*i*-PrOH (9:1) as eluent system at a flow rate of 1.0 mL·min^{-1}. Detection took place at λ = 210 nm. t_R(minor) = 33.88 min; t_R(major) = 38.31 min.

3-Fluorochromane-6-carbaldehyde (**19**)

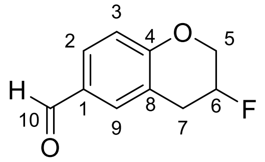

This compound was synthesised from **S27** (81 mg, 0.50 mmol) with amine:HF 1:7.5 according to General Procedure **B**. Purification by column chromatography on silica gel (*n*-pentane:EtOAc 1:1) afforded the desired product as a white solid (34 mg, 38%, *e.r.* 20:80).

R*f* = 0.57 (*n*-pentane:EtOAc 1:1).

$[\alpha]_D^{27}$: +58.2° (*c* = 0.10, $CHCl_3$ for sample with *e.r.* 20:80).

M.p.: 49 – 51 °C.

^{1}H NMR (400 MHz, $CDCl_3$): δ 9.86 (s, 1H, H-C10), 7.68 (dd, $^3J_{HH}$ = 8.4 Hz, $^4J_{HH}$ = 2.1 Hz, 1H, H-C2), 7.64 (dt, $^4J_{HH}$ = 2.1, 0.9 Hz, 1H, H-C9), 6.98 (d, $^3J_{HH}$ = 8.4 Hz, 1H, H-C3), 5.18 (dqd, $^2J_{FH}$ = 47.1 Hz, $^3J_{HH}$ = 3.8, 1.6 Hz, 1H, H-C6), 4.49 (dddt, $^2J_{HH}$ = 12.3 Hz, $^3J_{FH}$ = 9.7 Hz, $^3J_{HH}$ = 3.8 Hz, $^4J_{HH}$ = 1.3 Hz, 1H, H-C5), 4.20 (ddq, $^3J_{FH}$ = 33.1 Hz, $^2J_{HH}$ = 12.3 Hz, $^3J_{HH}$ = $^4J_{HH}$ = 1.3 Hz, 1H, H-C5), 3.18 (dddt, $^3J_{FH}$ = 34.3 Hz, $^2J_{HH}$ = 17.7 Hz, $^4J_{HH}$ = 4.2 Hz, 1.3 Hz, 0.9 Hz, 1H, H-C7), 3.12 (tdddd, $^2J_{HH}$ = $^3J_{FH}$ = 17.7 Hz, $^3J_{HH}$ = 3.8 Hz, $^4J_{HH}$ = 2.2, 1.3, 0.9 Hz, 1H, H-C7).

^{13}C{^{1}H} NMR (126 MHz, $CDCl_3$): δ 190.9 (d, *J* = 7.8 Hz, 1C, C10), 159.0 (1C, C4), 132.5 (1C, C9), 130.5 (d, *J* = 1.1 Hz, 1C, C1), 130.3 (1C, C2), 118.8 (d, $^3J_{FC}$ = 1.5 Hz, 1C, C8), 117.5 (1C, C3), 83.4 (d, $^1J_{FC}$ = 175.8 Hz, 1C, C6), 67.6 (d, $^2J_{FC}$ = 21.7 Hz, 1C, C5), 30.4 (d, $^2J_{FC}$ = 23.0 Hz, 1C, C5).

^{19}F NMR (470 MHz, $CDCl_3$): δ -186.61 – -188.38 (m, 1F, F-C6).

ESI-EM-MS: (*m/z*) required: $[(C_{10}H_9O_2F)Na]^+$ = 203.0479; (*m/z)* found: $[(C_{10}H_9O_2F)Na]^+$ = 203.0476.

FT-IR (ATR) ($\tilde{\nu}$ = cm^{-1}): 2927 (w), 2742 (w), 1727 (w), 1682 (m), 1605 (m), 1578 (m), 1497 (m), 1451 (w), 1437 (w), 1422 (w), 1386 (w), 1345 (w), 1326 (w), 1311 (m), 1245 (s), 1200 (m), 1153 (m), 1113 (m), 1088 (m), 1062 (m), 1001 (m), 940 (m), 925 (m), 910 (m), 872 (m), 859 (m), 839 (m), 814 (m), 769 (m), 754 (w), 739 (w), 724 (w), 680 (w).

The *e.r.* of the product was determined by HPLC analysis using a Reprosil Chiral AM column (10 µm, 250 x 4.6 mm) as the stationary phase and hexanes:*i*-PrOH (97:3) as eluent system at a flow rate of 1.0 mL·min^{-1}. Detection took place at λ = 254 nm. t_R(minor) = 13.38 min; t_R(major) = 15.85 min.

3-Fluoro-6-(methylsulfonyl)chromane (18)

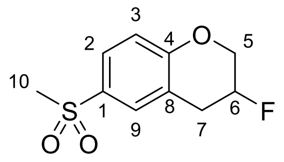

This compound was synthesised from **S30** (106 mg, 0.50 mmol) with amine:HF 1:7.5 according to General Procedure **B**. Purification by column chromatography on silica gel (*n*-pentane:EtOAc 1:1) afforded the desired product as a colorless solid (67 mg, 56%, *e.r.* 16:84).

R$_f$ = 0.38 (*n*-pentane:EtOAc 1:1).

$[\alpha]_D^{27}$: +44.0° (*c* = 0.10, $CHCl_3$ for sample with *e.r.* 16:84).

M.p.: 103 – 104 °C.

^{1}H NMR (599 MHz, $CDCl_3$): δ 7.70 (dd, $^3J_{HH}$ = 8.5 Hz, $^4J_{HH}$ = 2.3 Hz, 1H, H-C2), 7.68 (dt, $^4J_{HH}$ = 2.3 Hz, 0.7 Hz, 1H, H-C9), 7.00 (d, $^3J_{HH}$ = 8.5 Hz, 1H, H-C3), 5.18 (dqd, $^2J_{FH}$ = 47.0 Hz, $^3J_{HH}$ = 3.6, 1.4 Hz, 1H, H-C6), 4.49 (ddddd, $^2J_{HH}$ = 12.1 Hz, $^3J_{FH}$ = 9.3 Hz, $^3J_{HH}$ = 3.6 Hz, $^4J_{HH}$ = 2.3, 0.8 Hz, 1H, H-C5), 4.18 (dddt, $^3J_{FH}$ = 33.8 Hz, $^2J_{HH}$ = 12.1 Hz, $^3J_{HH}$ = $^4J_{HH}$ = 1.5 Hz, 1.1 Hz, 1H, H-C5), 3.16 (ddddt, $^3J_{FH}$ = 32.9 Hz, $^2J_{HH}$ = 17.2 Hz, $^3J_{HH}$ = 3.4 Hz, $^4J_{HH}$ = 1.1, 0.8 Hz, 1H, H-C7), 3.13 (tdddd, $^2J_{HH}$ = $^3J_{FH}$ = 17.5 Hz, $^3J_{HH}$ = 3.4 Hz, $^4J_{HH}$ = 2.3, 1.1, 0.7 Hz, 1H, H-C7), 3.02 (s, 3H, H-C10).

^{13}C{^{1}H} NMR (151 MHz, $CDCl_3$): δ 158.1 (1C, C4), 133.0 (1C, C1), 130.2 (1C, C9), 127.7 (1C, C2), 119.1 (1C, C8), 117.8 (1C, C3), 83.1 (d, $^1J_{FC}$ = 176.3 Hz, 1C, C6), 67.6 (d, $^2J_{FC}$ = 21.7 Hz, 1C, C5), 45.0 (1C, C10), 30.4 (d, $^2J_{FC}$ = 23.4 Hz, 1C, C7).

^{19}F NMR (564 MHz, $CDCl_3$): δ -186.2 – -188.4 (m, 1F, F-C6).

FT-IR ($\tilde{\nu}$ = cm^{-1}): 2960 (w), 2933 (w), 1604 (w), 1577 (m), 1487 (m), 1422 (w), 1339 (w), 1315 (m), 1293 (m), 1262 (m), 1239 (m), 1193 (w), 1145 (m), 1122 (s), 1089 (s), 1059 (m), 1000 (m), 960 (m), 913 (w), 894 (m), 874 (w), 845 (m), 829 (m), 812 (m), 762 (s), 739 (m).

ESI-EM-MS: (*m/z*) required: $[(C_{10}H_{11}O_3SF)Na]^+$ = 253.0305; (*m/z*) found: $[(C_{10}H_{11}O_3SF)Na]^+$ = 253.0325.

The *e.r.* of the product was determined by HPLC analysis using a DAICEL Chiral OJ-H column (5 µm, 250 x 4.6 mm) as the stationary phase and hexanes:*i*-PrOH (7:3) as eluent system at a flow rate of 0.7 mL·min^{-1}. Detection took place at λ = 210 nm. t_R(minor) = 42.73 min; t_R(major) = 56.03 min.

6-Nitro-3-fluorochromane (20)

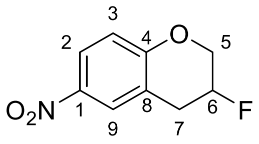

This compound was synthesised from **S31** (90 mg, 0.50 mmol) with amine:HF 1:7.5 according to General Procedure **B**. Purification by column chromatography on silica gel (*n*-pentane:EtOAc 1:1) afforded the desired product as a colorless solid (43 mg, 44%, *e.r.* 17:83).

$\mathbf{R}_f$ = 0.18 (CyH:EtOAc 4:1).

$[\alpha]_D^{25}$: +62.6° (c = 0.50, $CHCl_3$ for sample with *e.r.* 17:83).

M.p.: 103 – 104 °C.

^{1}H NMR (500 MHz, $CDCl_3$): δ 8.06 – 8.00 (m, 2H, H-C2/9), 6.96 – 6.90 (m, 1H, H-C3), 5.18 (dqd, J = 46.9, 3.5, 1.5 Hz, 1H, H-C6), 4.58 – 4.46 (m, 1H, H-C5), 4.20 (ddq, J = 34.2, 12.4, 1.3 Hz, 1H, H-C5), 3.26 – 3.07 (m, 2H, H-C7).

^{13}C{^{1}H} NMR (126 MHz, $CDCl_3$): δ 159.1 (1C, C4), 141.8 (1C, C1), 126.4 (1C, C9), 124.1 (1C, C2), 118.7 (d, $^3J_{FC}$ = 1.2 Hz, 1C, C8), 117.4 (1C, C3), 82.9 (d, $^1J_{FC}$ = 176.2 Hz, 1C, C6), 67.8 (d, $^2J_{FC}$ = 21.4 Hz, 1C, C5), 30.4 (d, $^2J_{FC}$ = 23.2 Hz, 1C, C7).

^{19}F NMR (470 MHz, $CDCl_3$): δ -187.2 – 187.5 (m, 1F, F-C6).

ESI-EM-MS: (*m/z*) required: $[(C_9H_8NO_3F)Na]^+$ = 220.0380; (*m/z)* found: $[(C_9H_8NO_3F)Na]^+$ = 220.0382;

FT-IR (ATR) ($\tilde{\nu}$ = cm^{-1}): 1651 (w), 1618 (w), 1497 (s), 1469 (s), 1425 (w), 1412 (w), 1386 (w), 1365 (w), 1302 (m), 1286 (m), 1252 (w), 1235 (m), 1217 (m), 1113 (w), 1073 (s), 1055 (m), 994 (w), 930 (m), 878 (s), 789 (m), 746 (m), 704 (w).

The *e.r.* of the product was determined by HPLC analysis using a chiral Reprosil Chiral-AM column (10 µm, 250 x 4.6 mm) as the stationary phase and *n*-hexane/*i*-PrOH (95:5) as eluent system at a flow rate of 1.0 mL·min^{-1}. Detection took place at λ = 290 nm. t_R(minor) = 11.62 min; t_R(major) = 13.21 min.

6-Bromo-3-fluoro-3-methylchromane (21)

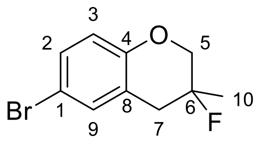

This compound was synthesised from **S34** (114 mg, 0.50 mmol) with amine:HF 1:5 according to General Procedure **B**. Purification by column chromatography on silica gel (*n*-pentane:CH_2Cl_2 1:0 → 10:1, dry load) afforded the desired product as a white solid (62 mg, 51%, *e.r.* 45:55).

R*f* = 0.39 (CyH:EtOAc 10:1).

M.p.: 49 – 50 °C.

^{1}H NMR (599 MHz, $CDCl_3$): δ 7.22 (ddt, $^3J_{HH}$ = 8.7 Hz, $^4J_{HH}$ = 2.5 Hz, J =0.8 Hz, 1H, H-C2), 7.17 (dt, $^4J_{HH}$ = 2.3 Hz, 1.0 Hz, 1H, H-C9), 6.76 (d, $^3J_{HH}$ = 8.7 Hz, 1H, H-C3), 4.18 (ddd, $^2J_{HH}$ = 11.7 Hz, $^3J_{FH}$ = 7.4 Hz, $^4J_{HH}$ = 2.6 Hz, 1H, H-C5), 3.86 (dddd, $^3J_{FH}$ = 27.8 Hz, $^2J_{HH}$ = 11.7 Hz, $^4J_{HH}$ = 1.5 Hz, 0.9 Hz, 1H, H-C5), 3.01 (dddt, $^2J_{HH}$ = 17.1 Hz, $^3J_{FH}$ = 15.2 Hz, $^4J_{HH}$ = 2.6, 0.7 Hz, 1H, H-C7), 2.89 (ddq, $^3J_{FH}$ = 32.1 Hz, $^2J_{HH}$ = 17.2 Hz, $^4J_{HH}$ = 1.2 Hz, 1H, H-C7), 1.50 (d, $^3J_{FH}$ = 20.7 Hz, 3H, H-C10).

^{13}C{^{1}H} NMR (151 MHz, $CDCl_3$): δ 152.4 (C4), 132.4 (C9), 130.8 (C2), 121.3 (d, $^3J_{FC}$ = 1.7 Hz, C8), 118.5 (C3), 113.2 (C1), 88.7 (d, $^1J_{FC}$ = 173.7 Hz, C6), 71.1 (d, $^2J_{FC}$ = 24.0 Hz, C5), 36.4 (d, $^2J_{FC}$ = 24.6 Hz, C7), 23.0 (d, $^2J_{FC}$ = 23.9 Hz, C10).

^{19}F NMR (564 MHz, $CDCl_3$): δ -153.7 (ddqdd, $^3J_{FH}$ = 32.1, 27.8, 20.7, 15.2, 7.4 Hz, 1F, F-C6).

ESI-EM-MS: (*m/z*) required: $[(C_{10}H_{10}BrOF)Na]^+$ = 266.9791; (*m/z)* found: $[(C_{10}H_{10}BrOF)Na]^+$ = 266.9775.

FT-IR (ATR) ($\tilde{\nu}$ = cm^{-1}): 2982 (w), 2937 (w), 2877 (w), 1579 (w), 1481 (s), 1455 (w), 1413 (w), 1382 (w), 1323 (w), 1298 (m), 1278 (m), 1258 (w), 1237 (s), 1180 (s), 1155 (w), 1116 (m), 1106 (m), 1073 (w), 1044 (m), 994 (w), 956 (w), 909 (m), 896 (w), 874 (w), 856 (m), 805 (s), 750 (w), 673 (m).

The *e.r.* of the product was determined by HPLC analysis using a Reprosil Chiral-AM column (10 µm, 250 x 4.6 mm) as the stationary phase and *n*-hexane:*i*-PrOH (99.5:0.5) as eluent system at a flow rate of 0.5 mL·min^{-1}. Detection took place at λ = 290 nm. t_R(minor) = 13.79 min; t_R(major) = 16.00 min.

3. Mechanistic Study

a. Electronic Modification of Aryl Substituent

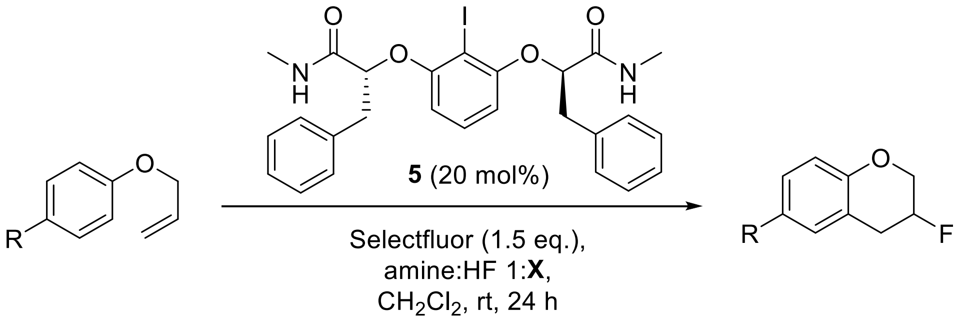

Following General procedure **B**, the reactivity of a group of electronically different allyl aryl ethers (0.2 mmol) were explored modifying the amine:HF ratio. Results were obtained by ^{19}F NMR using ethyl fluoroacetate as internal standard and are summarised hereafter.

entry	R	σ_p^+	amine:HF	^{19}F yield (%)	log(e.r.)
**1**	F	-0.07	1:4.5	37	0.91
			1:5	50	0.87
			1:7.5	20	0.67
**2**	Cl	0.11	1:4.5	50	0.91
			1:5	69	0.87
			1:7.5	24	0.66
**3**	Br	0.15	1:4.5	45	0.91
			1:5	68	0.91
			1:7.5	29	0.76
			1:9.2	9	0.45
**4**	CO_2Me	0.49	1:5	75	0.92
			1:7.5	73	0.66
			1:9.2	52	0.33
**5**	CN	0.66	1:5	33	-
**6**	CHO	0.73	1:5	22	-
**7**	NO_2	0.79	1:5	9	-
			1:7.5	43	0.72
			1:9.2	48	0.47

b. Substrate Modification Experiments

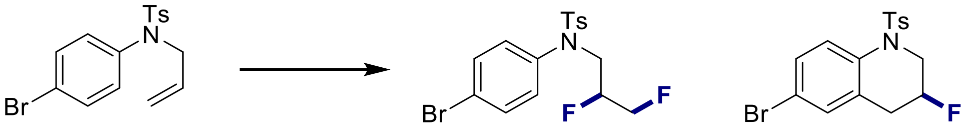

26 **27** 60% **28** <5%

N-(4-Bromophenyl)-N-(2,3-difluoropropyl)-4-methylbenzenesulfonamide (27)

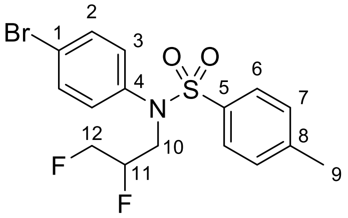

Based on procedure **B**, a Teflon® vessel was charged with **26** (73.0 mg, 0.20 mmol, 1.0 equiv.), **7** (22.0 mg, 0.04 mmol, 0.2 equiv.) and CH_2Cl_2 (0.5 mL). Then, amine:HF 1:5 (0.5 mL) and Selectfluor (106 mg, 0.30 mmol, 1.5 equiv.) were added. The reaction vessel was sealed with a Teflon® screw cap. After stirring at 350 rpm at room temperature for 24 h, the mixture was poured into an aqueous saturated solution of $NaHCO_3$ (25 mL). Once the gas evolution has ceased the aqueous layer was extracted with CH_2Cl_2 (3 x 25 mL). The combined organic layers were dried over $MgSO_4$, filtered, concentrated under reduced pressure and analysed by ^{19}F NMR using ethyl fluoroacetate as internal standard (^{19}F NMR yield: 60%). Purification by column chromatography on silica gel (*n*-pentane:CH_2Cl_2 1:1) afforded a colorless oil (25 mg, 31%).

R_f = 0.36 (CyH/EtOAc 4:1).

^{1}H NMR (599 MHz, $CDCl_3$): δ 7.46 (m, 2H, H-C6), 7.45 (m, 2H, H-C2), 7.26 (m, 2H, H-C7), 6.94 (m, 2H, H-C3), 4.78 (ddtdd, $^2J_{FH}$ = 47.1 Hz, $^3J_{FH}$ = 23.8 Hz, $^3J_{HH}$ = 6.2, 4.1, 2.3 Hz, 1H, H-C11), 4.67 (dddd, $^2J_{FH}$ = 47.4 Hz, $^3J_{FH}$ = 21.3 Hz, $^2J_{HH}$ = 11.1 Hz, $^3J_{HH}$ = 2.2 Hz, 1H, H-C12), 4.57 (dddd, $^2J_{FH}$ = 47.1 Hz, $^3J_{FH}$ = 28.0 Hz, $^2J_{HH}$ = 11.2 Hz, $^3J_{HH}$ = 4.3 Hz, 1H, H-C12), 3.81 (dd, $^3J_{FH}$ = 16.7 Hz, $^3J_{HH}$ = 6.0 Hz, 2H, H-C10), 2.43 (s, 3H, H-C9).

^{13}C{^{1}H} NMR (151 MHz, $CDCl_3$): δ 144.4 (1C, C8), 139.0 (1C, C4), 134.5 (1C, C5), 132.7 (2C, C2), 130.5 (2C, C3), 129.8 (2C, C7), 127.9 (2C, C6), 122.5 (1C, C1), 90.0 (dd, $^1J_{FC}$ = 178.4 Hz, $^2J_{FC}$ = 19.3 Hz, C1, C11), 82.1 (dd, $^1J_{FC}$ = 174.5 Hz, $^2J_{FC}$ = 21.8 Hz, C1, C12), 50.7 (dd, $^2J_{FC}$ = 26.8 Hz, $^3J_{FC}$ = 8.1 Hz, C1, C10), 21.7 (C1, C9).

^{19}F NMR (564 MHz, $CDCl_3$): δ -192.9 (dddtd, $^2J_{FH}$ = 47.1 Hz, $^3J_{FH}$ = 27.9, 21.4, 17.0 Hz, $^3J_{FF}$ = 12.5 Hz, 1F, F-C11), -34.9 (tdd, $^2J_{FH}$ = 47.4 Hz, $^3J_{FH}$ = 23.8 Hz, $^3J_{FF}$ = 12.5 Hz, 1F, F-C12).

ESI-EM-MS: (*m/z*) required: $[(C_{16}H_{16}BrNO_2SF_2)Na]^+$ = 427.9925; (*m/z)* found: $[(C_{16}H_{16}BrNO_2SF_2)Na]^+$ = 427.9922.

FT-IR (ATR) ($\tilde{\nu}$ = cm^{-1}): 2962 (w), 1598 (w), 1486 (m), 1451 (w), 1399 (w), 1349 (m), 1306 (w), 1269 (w), 1223 (w), 1186 (w), 1162 (s), 1072 (m), 1031 (m), 1011 (m), 984 (w), 923 (w), 872 (m), 836 (m), 814 (m), 743 (m), 712 (s), 683 (w), 661 (s).

c. Deuteration Experiments

i. NMR Spectra of Crude Reaction Mixtures

1. From Catalyst {*R*,*R*}-**5**

In order to interrogate the stereoselectivity of the reaction, two experiments were independently performed using the *Z*-isomer **30** and *E*-isomer **31** using **{*R*,*R*}-7** as catalyst.

- Reaction **A** using *Z*-isomer:

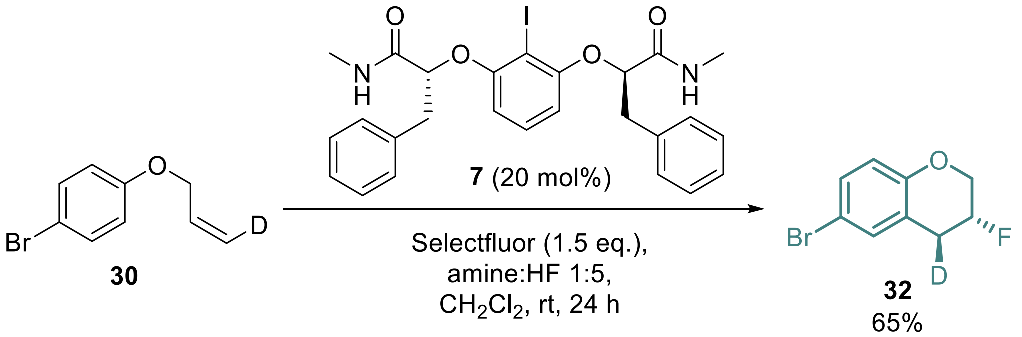

(3*R*,4*S*)-6-Bromo-3-fluorochromane-4-*d* (32)

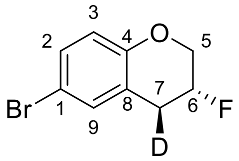

This compound was synthesised from **30** (43 mg, 0.20 mmol) with amine:HF 1:5 according to General Procedure **B.** The crude was analysed by ^{19}F NMR using ethyl fluoroacetate as internal standard (^{19}F yield: 65%). Purification by column chromatography on silica gel (*n*-pentane:CH_2Cl_2 4:1) afforded the desired product as a white solid (21 mg, 45%, *e.r.* 13:87).

R*f* = 0.19 (*n*-pentane:CH_2Cl_2 4:1). $[\alpha]_D^{25}$: +34.5° (*c* = 1.00, $CHCl_3$ for sample with *e.r.* 13:87). **M.p.**: 73 – 74 C. **^{1}H NMR** (599 MHz, $CDCl_3$): δ 7.22 (ddd, $^3J_{HH}$ = 8.7 Hz, $^4J_{HH}$ = 2.4 Hz, J = 0.6 Hz, 1H, H-C2), 7.19 (dd, $^4J_{HH}$ = 2.4 Hz, J = 0.9 Hz, 1H, H-C9), 6.75 (d, $^3J_{HH}$ = 8.7 Hz, 1H, H-C3), 5.09 (dtd, $^2J_{FH}$ = 47.4 Hz, $^3J_{HH}$ = 3.8, 1.6 Hz, 1H, H-C6), 4 .36 (dddd, $^2J_{HH}$ = 12.1 Hz, $^3J_{FH}$ = 9.1 Hz, $^3J_{HH}$ = 4.1 Hz, $^4J_{HH}$ = 2.4 Hz, 1H, H-C5), 4.08 (dddd, $^3J_{FH}$ = 31.7 Hz, $^2J_{HH}$ = 12.1 Hz, $^3J_{HH}$ = 1.7 Hz, J = 0.9 Hz, 1H, H-C5), 3.01 (dp, $^3J_{FH}$ = 16.7 Hz, $^2J_{HD}$ = $^4J_{HH}$ = 2.9 Hz, 1H). **^{13}C{^{1}H} NMR** (151 MHz, $CDCl_3$): δ 152.9 (1C, C4), 132.6 (1C, C9), 130.9 (1C, C2), 120.3 (1C, C8), 118.6 (1C, C3), 113.3 (1C, C1), 83.6 (d, $^1J_{FC}$ = 175.6 Hz, 1C, C6), 67.1 (d, $^2J_{FC}$ = 21.9 Hz, 1C, C5), 30.2 (dt, $^2J_{FC}$ = 23.1 Hz, $^1J_{CD}$ = 19.9 Hz, 1C, C7). **^{19}F{^{1}H} NMR** (564 MHz, $CDCl_3$): δ -187.4 (t, $^3J_{FD}$ = 5.3 Hz, 1F, F-C6). **^{19}F NMR** (564 MHz, $CDCl_3$): δ -187.4 (ddddt, $^2J_{FH}$ = 47.4 Hz, $^3J_{FH}$ = 31.7, 16.7, 9.1 Hz, $^3J_{FD}$ = 5.3 Hz, 1F, F-C6). **EI-EM-MS**: (*m/z*) required: $[C_9H_7OBrDF]^{+\bullet}$ = 230.9800; (*m/z*) found: $[C_9H_8OFBr]^{+\bullet}$ = 230.9798.

The *e.r.* of the product was determined by HPLC analysis using a DAICEL Chiral OJ-H column (5 µm, 250 x 4.6 mm) as the stationary phase and hexanes:*i*-PrOH (99.5:0.5) as eluent system at a flow rate of 0.5 mL·min^{-1}. Detection took place at λ = 230 nm. t_R(minor) = 59.17 min; t_R(major) = 74.84 min.

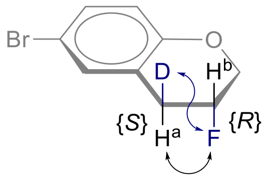

32
$^3J_{FD}$=5.3 Hz
(calculated $^3J_{FH}$=34.5 Hz)
$^3J_{FH^a}$=16.7 Hz

- Reaction **B** using *E*-isomer:

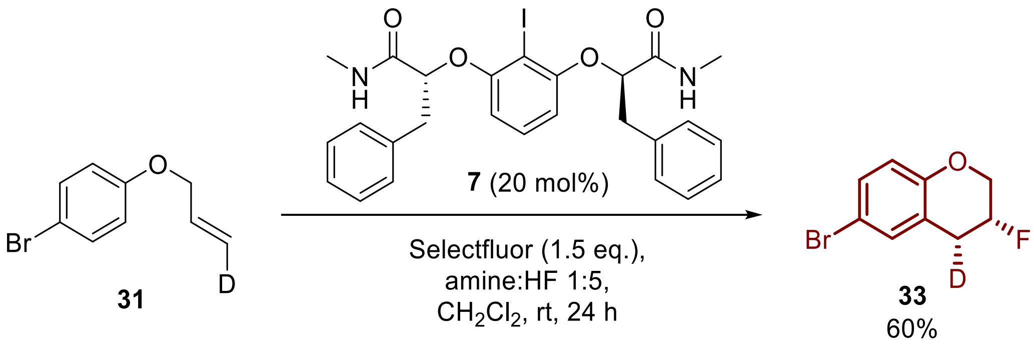

(3*R*,4*R*)-6-Bromo-3-fluorochromane-4-*d* (33)

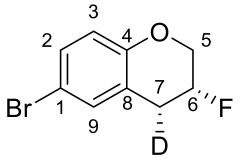

This compound was synthesised from **31** (43 mg, 0.20 mmol, 90% deuterated by ^{1}H NMR) with amine:HF 1:5 according to General Procedure **B**. The crude was analysed by ^{19}F NMR using ethyl fluoroacetate as internal standard (^{19}F yield: 60%). Purification by column chromatography on silica gel (*n*-pentane:CH_2Cl_2 4:1) afforded the desired product as a white solid (22 mg, 47%, *e.r.* 13:87, 89% deuterated by ^{19}F NMR).

R$_f$ = 0.19 (*n*-pentane:CH_2Cl_2 4:1). $[\alpha]_D^{25}$: +29.8° (*c* = 1.00, $CHCl_3$ for sample with *e.r.* 13:87). **M.p.**: 70 – 72 C. **^{1}H NMR** (500 MHz, $CDCl_3$): δ 7.22 (ddd, $^{3}J_{HH}$ = 8.7 Hz, $^{4}J_{HH}$ = 2.4 Hz, J = 0.8 Hz, 1H, H-C2), 7.19 (dd, $^{4}J_{HH}$ = 2.4 Hz, J = 1.1 Hz, 1H, H-C9), 6.75 (d, $^{3}J_{HH}$ = 8.6 Hz, 1H, H-C3), 5.10 (dtd, $^{2}J_{FH}$ = 47.4 Hz, $^{3}J_{HH}$ = 4.2, 1.6 Hz, 1H, H-C6), 4.37 (ddd, $^{2}J_{HH}$ = 12.2 Hz, $^{3}J_{FH}$ = 9.2 Hz, $^{3}J_{HH}$ = 4.0 Hz, 1H), 4.09 (ddt, $^{3}J_{FH}$ = 32.0 Hz, $^{2}J_{HH}$ = 12.1 Hz, $^{3}J_{HH}$ = J =1.6 Hz, 1H), 3.08 (br d, $^{3}J_{FH}$ = 35.4 Hz, 1H).**^{13}C{^{1}H} NMR** (126 MHz, $CDCl_3$): δ 152.9 (1C, C4), 132.6 (1C, C9), 130.9 (1C, C2), 120.3 (1C, C8), 118.6 (C3), 113.3 (C1), 83.6 (d, $^{1}J_{FC}$ = 175.7 Hz, 1C, C6), 67.1 (d, $^{2}J_{FC}$ = 21.8 Hz, 1C, C5), 30.2 (dt, $^{2}J_{FC}$ = 22.8 Hz, $^{1}J_{CD}$ = 20.7 Hz, 1C, C7). **^{19}F{^{1}H} NMR** (470 MHz, $CDCl_3$): δ -187.4 (t, $^{3}J_{FD}$ = 2.3 Hz, 1F, F-C6). **^{19}F NMR** (470 MHz, $CDCl_3$): δ -187.4 (ddddt, $^{2}J_{FH}$ = 47.4 Hz, $^{3}J_{FH}$ = 35.4, 32.0, 9.2 Hz, $^{3}J_{FD}$ = 2.3 Hz, 1F, F-C6). **EI-EM-MS**: (*m/z*) required: $[C_9H_7OBrDF]^{+\bullet}$ = 230.9800; (*m/z*) found: $[C_9H_8OFBr]^{+\bullet}$ = 230.9798. **FT-IR** (ATR) ($\tilde{\nu}$ = cm^{-1}): 2983 (w), 1576 (w), 1480 (s), 1455 (w), 1411 (w), 1383 (w), 1345 (w), 1288 (w), 1257 (s), 1245 (s), 1236 (s), 1188 (m), 1129 (m), 1095 (m), 1080 (m), 1065 (m), 1056 (w), 997 (m), 961 (m), 913 (m), 888 (w), 852 (w), 827 (s), 813 (s), 797 (s), 731 (w).

The *e.r.* of the product was determined by HPLC analysis using a DAICEL Chiral OJ-H column (5 µm, 250 x 4.6 mm) as the stationary phase and hexanes:*i*-PrOH (99.5:0.5) as eluent system at a flow rate of 0.5 mL·min^{-1}. Detection took place at λ = 230 nm. t_R(minor) = 59.07 min; t_R(major) = 73.53 min.

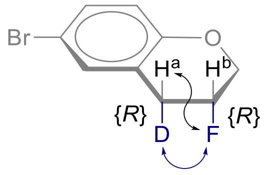

33
$^{3}J_{FH^a}$=35.4 Hz
$^{3}J_{FD}$=2.3 Hz
(calculated $^{3}J_{FH}$=15.0 Hz)

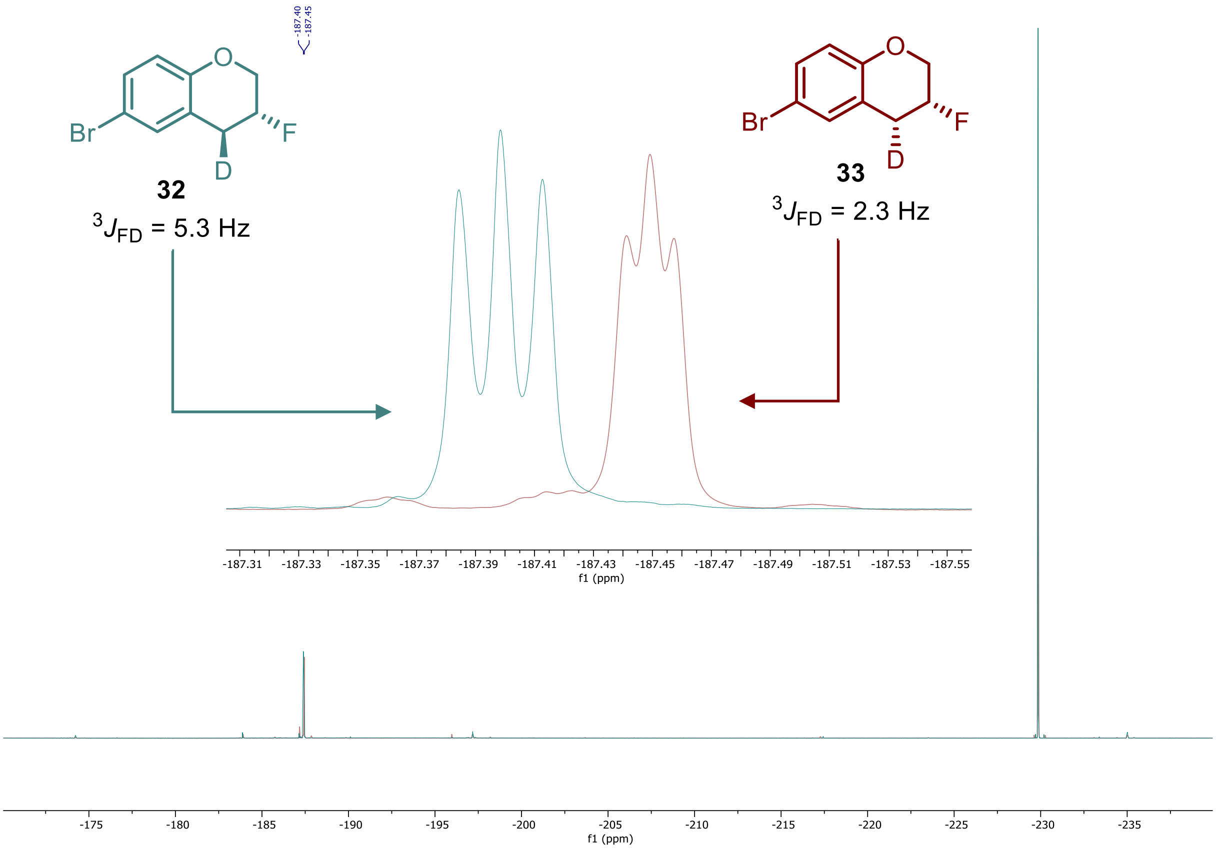

Figure S1: Stacked ^{19}F NMR spectra of crude reaction mixture using ethyl fluoroacetate as internal standard. Teal spectrum (376 MHz, $CDCl_3$): reaction mixture **A** using *Z*-isomer **30**. Dark red spectra (282 MHz, $CDCl_3$): reaction mixture **B** using *E*-isomer **31**.

2. From Catalyst {*S*,*S*}-**5**

Two similar experiments were independently performed using the *Z*-isomer **30** and *E*-isomer **31** using **{*S*,*S*}-7** as catalyst.

- Reaction **C** using Z-isomer:

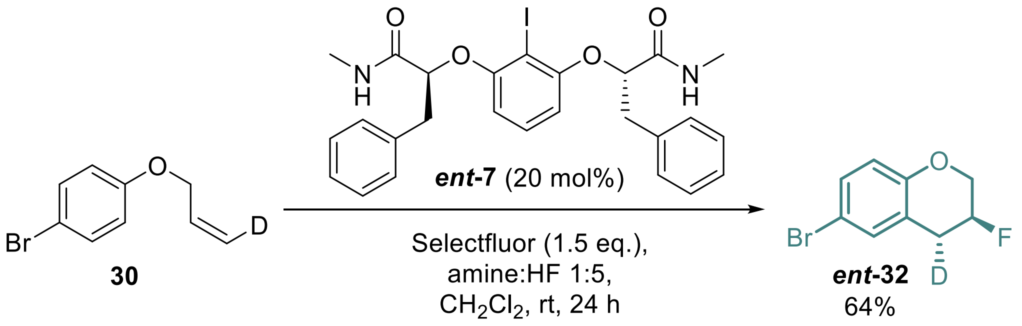

(3*S*,4*R*)-6-Bromo-3-fluorochromane-4-*d* (*ent*-32)

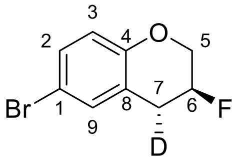

This compound was synthesised from **29** (43 mg, 0.20 mmol) with amine:HF 1:5 according to General Procedure **B**. The crude was analysed by ^{19}F NMR using ethyl fluoroacetate as internal standard (^{19}F yield: 64%). Purification by column chromatography on silica gel (*n*-pentane:CH_2Cl_2 4:1) afforded the desired product as a white solid (27 mg, 58%, *e.r.* 12:88).

M.p.: 73 – 74 C. $[\alpha]_D^{24}$: -37.3° (*c* = 1.00, $CHCl_3$ for sample with *e.r.* 12:88). **^{1}H NMR** (599 MHz, $CDCl_3$): δ 7.22 (ddd, $^3J_{HH}$ = 8.7 Hz, $^4J_{HH}$ = 2.5 Hz, J = 0.4 Hz, 1H, H-C2), 7.19 (dd, $^4J_{HH}$ = 2.4 Hz, J = 0.8 Hz, 1H, H-C9), 6.75 (d, $^3J_{HH}$ = 8.6 Hz, 1H, H-C3), 5.09 (dtd, $^2J_{FH}$ = 47.4 Hz, $^3J_{HH}$ = 3.9, 1.6 Hz, 1H, H-C6), 4.36 (dddd, $^2J_{HH}$ = 11.8 Hz, $^3J_{FH}$ = 9.1 Hz, $^3J_{HH}$ = 4.1 Hz, $^4J_{HH}$ = 2.4 Hz, 1H, H-C5), 4.09 (dddd, $^3J_{FH}$ = 31.7 Hz, $^2J_{HH}$ = 12.2 Hz, $^3J_{HH}$ = 1.7 Hz, J = 0.9 Hz, 1H, H-C5), 3.01 (dp, $^3J_{FH}$ = 16.7 Hz, $^2J_{HD}$ = $^4J_{HH}$ = 2.9 Hz, 1H). **^{13}C{^{1}H} NMR** (151 MHz, $CDCl_3$): δ 152.9 (1C, C4), 132.6 (1C, C9), 130.9 (1C, C2), 120.3 (1C, C8), 118.6 (1C, C3), 113.3 (1C, C1), 83.6 (d, $^1J_{FC}$ = 175.4 Hz, 1C, C6), 67.1 (d, $^2J_{FC}$ = 22.0 Hz, 1C, C5), 30.2 (dt, $^2J_{FC}$ = 23.3 Hz, $^1J_{CD}$ = 20.2 Hz, 1C, C7). **^{19}F{^{1}H} NMR** (564 MHz, $CDCl_3$): δ -187.4 (t, $^3J_{FD}$ = 5.3 Hz, 1F, F-C6). **^{19}F NMR** (564 MHz, $CDCl_3$): δ -187.4 (ddddt, $^2J_{FH}$ = 47.4 Hz, $^3J_{FH}$ = 31.7, 16.7, 9.1 Hz, $^3J_{FD}$ = 5.3 Hz, 1F, F-C6). **EI-EM-MS**: (*m/z*) required: $[C_9H_7OBrDF]^{+\bullet}$ = 230.9800; (*m/z*) found: $[C_9H_8OFBr]^{+\bullet}$ = 230.9799. **FT-IR** (ATR) ($\tilde{\nu}$ = cm^{-1}): 2940 (w), 1577 (w), 1482 (m), 1455 (w), 1414 (w), 1386 (w), 1341 (w), 1292 (w), 1255 (m), 1235 (m), 1181 (m), 1126 (w), 1110 (w), 1078 (w), 1056 (m), 1027 (m), 949 (m), 919 (w), 881 (w), 861 (w), 839 (m), 829 (m), 810 (s), 756 (m), 735 (w).

The *e.r.* of the product was determined by HPLC analysis using a DAICEL Chiral OJ-H column (5 µm, 250 x 4.6 mm) as the stationary phase and hexanes:*i*-PrOH (99.5:0.5) as eluent system at a flow rate of 0.5 mL·min^{-1}. Detection took place at λ = 230 nm. t_R(minor) = 58.71 min; t_R(major) = 75.11 min.

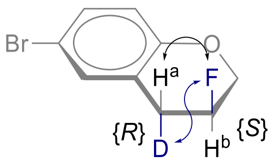

***ent*-32**
$^3J_{FH^a}$=16.7 Hz
$^3J_{FD}$=5.3 Hz
(calculated $^3J_{FH}$=34.5 Hz)

- Reaction **D** using *E*-isomer:

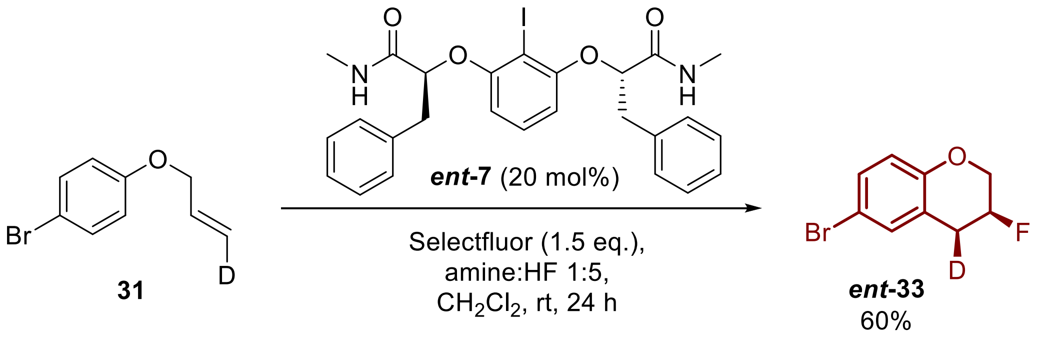

(3*S*,4*S*)-6-Bromo-3-fluorochromane-4-*d* (*ent*-33)

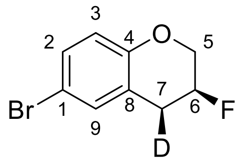

This compound was synthesised from **31** (43 mg, 0.20 mmol, 90% deuterated by ^{1}H NMR) with amine:HF 1:5 according to General Procedure **B**. The crude was analysed by ^{19}F NMR using ethyl fluoroacetate as internal standard (^{19}F yield: 60%). Purification by column chromatography on silica gel (*n*-pentane:CH_2Cl_2 4:1) afforded the desired product as a white solid (11 mg, 24%, *e.r.* 16:84, 88% deuterated by ^{19}F NMR).

M.p.: 70 – 71 C. $[\alpha]_{D}^{25}$: -25.2° (*c* = 0.25, $CHCl_3$ for sample with *e.r.* 16:84). **^{1}H NMR** (300 MHz, $CDCl_3$): δ 7.22 (dd, $^{3}J_{HH}$ = 8.6 Hz, $^{4}J_{HH}$ = 2.4 Hz, 1H, H-C2), 7.19 (m, 1H, H-C9), 6.75 (d, $^{3}J_{HH}$ = 8.6 Hz, 1H, H-C3), 5.10 (dtd, $^{2}J_{FH}$ = 47.3 Hz, $^{3}J_{HH}$ = 4.1, 1.4 Hz, 1H, H-C6), 4.37 (ddd, $^{2}J_{HH}$ = 12.1 Hz, $^{3}J_{FH}$ = 9.3 Hz, $^{3}J_{HH}$ = 4.0 Hz, 1H), 4.09 (ddt, $^{3}J_{FH}$ = 32.1 Hz, $^{2}J_{HH}$ = 12.1 Hz, $^{3}J_{HH}$ = *J* =1.5 Hz, 1H), 3.08 (br d, $^{3}J_{FH}$ = 35.7 Hz, 1H). **^{19}F{^{1}H} NMR** (282 MHz, $CDCl_3$): δ -187.5 (t, $^{3}J_{FD}$ = 2.3 Hz, 1F, F-C6). **^{19}F NMR** (282 MHz, $CDCl_3$): δ -187.5 (ddddt, $^{2}J_{FH}$ = 47.3 Hz, $^{3}J_{FH}$ = 35.7, 32.1, 9.3 Hz, $^{3}J_{FD}$ = 2.3 Hz, 1F, F-C6). **EI-EM-MS**: (*m/z*) required: $[C_9H_7OBrDF]^{+\bullet}$ = 230.9800; (*m/z*) found: $[C_9H_8OFBr]^{+\bullet}$ = 230.9800.

The *e.r.* of the product was determined by HPLC analysis using a DAICEL Chiral OJ-H column (5 µm, 250 x 4.6 mm) as the stationary phase and hexanes:*i*-PrOH (99.5:0.5) as eluent system at a flow rate of 0.5 mL·min^{-1}. Detection took place at λ = 230 nm. t_R(minor) = 58.84 min; t_R(major) = 74.69 min.

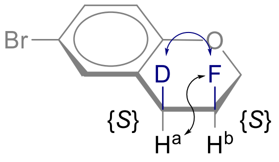

***ent*-33**

$^{3}J_{FD}$=2.3 Hz

(calculated $^{3}J_{FH}$=15.0 Hz)

$^{3}J_{FH^a}$=35.7 Hz

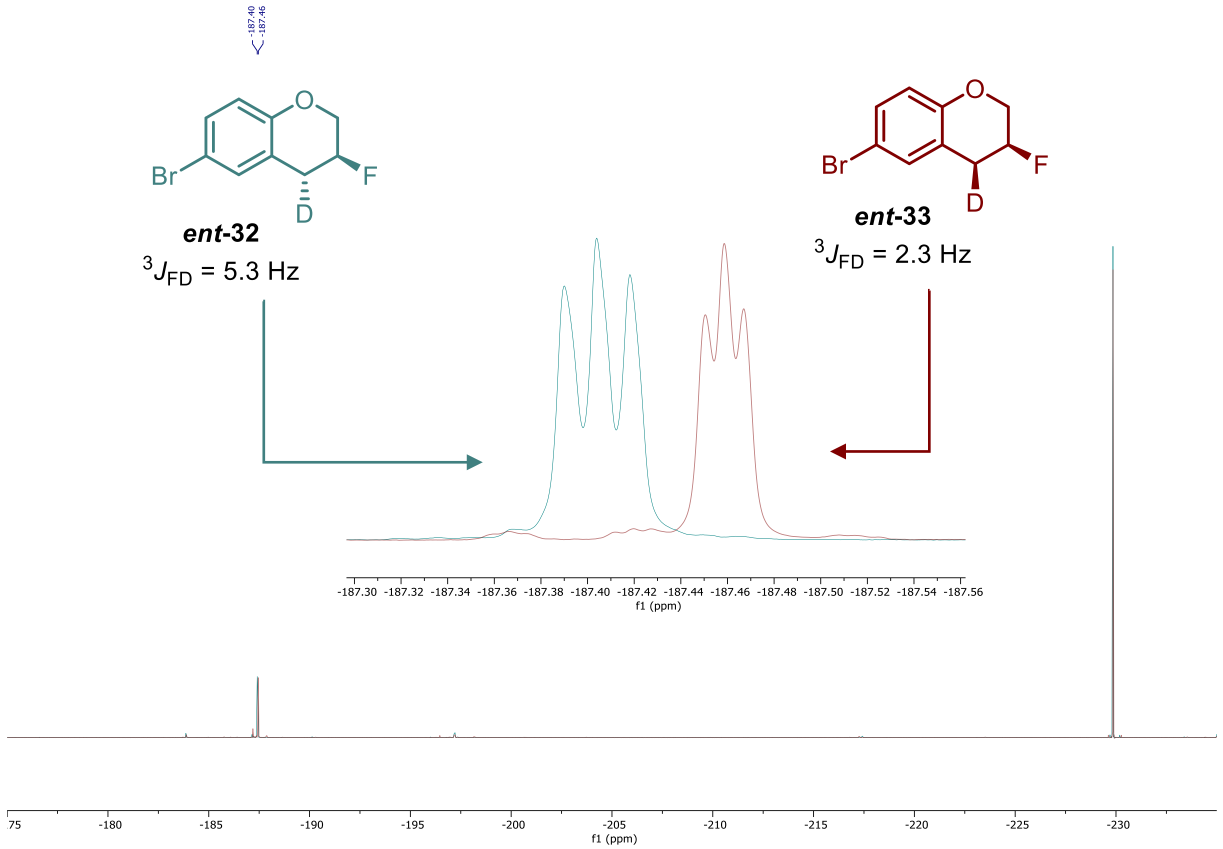

<u>Figure S2:</u> Stacked ^{19}F NMR spectra of crude reaction mixture using ethyl fluoroacetate as internal standard. Teal spectrum (376 MHz, $CDCl_3$): reaction mixture **C** using *Z*-isomer **30**. Dark red spectra (282 MHz, $CDCl_3$): reaction mixture **D** using *E*-isomer **310**.

.

4. HPLC traces.

6-Bromo-3-fluorochromane (6)

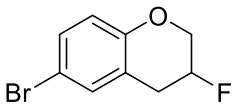

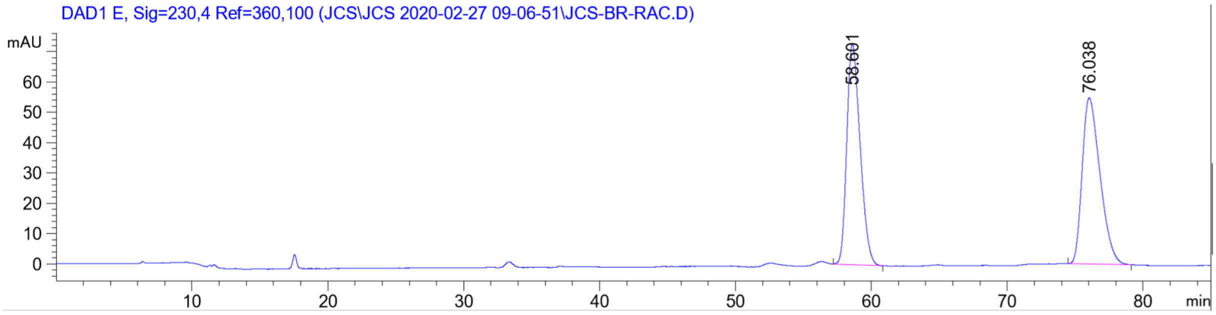

Signal 4: DAD1 E, Sig=230,4 Ref=360,100

Peak #	RetTime [min]	Type	Width [min]	Area [mAU*s]	Height [mAU]	Area %
1	58.601	BB	0.9876	4964.65527	72.79835	49.7469
2	76.038	BB	1.3308	5015.18164	54.84668	50.2531

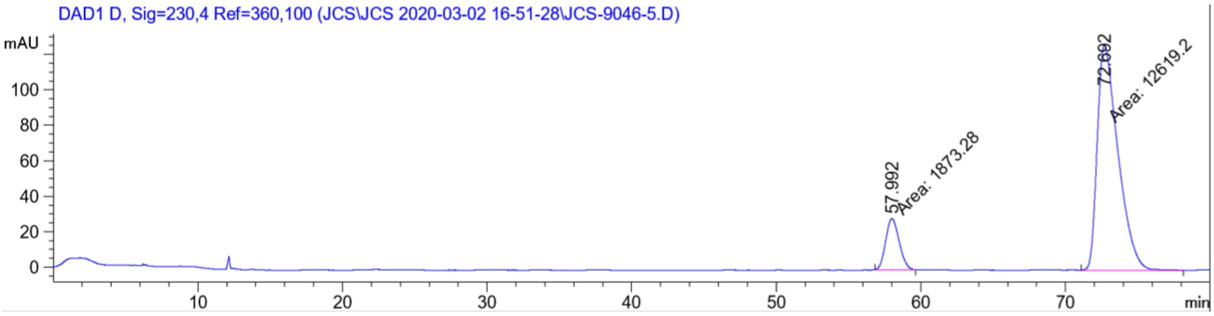

Signal 2: DAD1 D, Sig=230,4 Ref=360,100

Peak #	RetTime [min]	Type	Width [min]	Area [mAU*s]	Height [mAU]	Area %
1	57.992	MM	1.0837	1873.27551	28.80894	12.9258
2	72.692	MM	1.6542	1.26192e4	127.14421	87.0742

6-Chloro-3-fluorochromane (8)

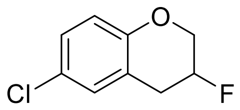

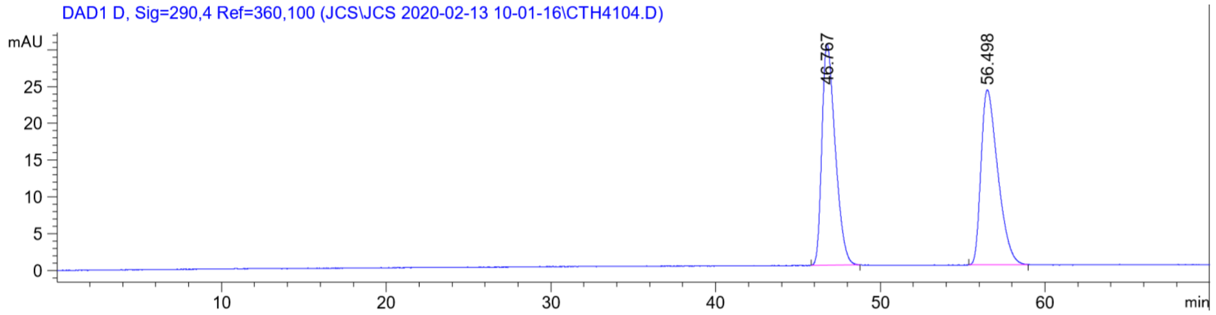

Signal 2: DAD1 D, Sig=290,4 Ref=360,100

Peak #	RetTime [min]	Type	Width [min]	Area [mAU*s]	Height [mAU]	Area %
1	46.767	BB	0.8206	1689.41724	30.10874	49.9690
2	56.498	BB	0.9133	1691.51099	23.79759	50.0310

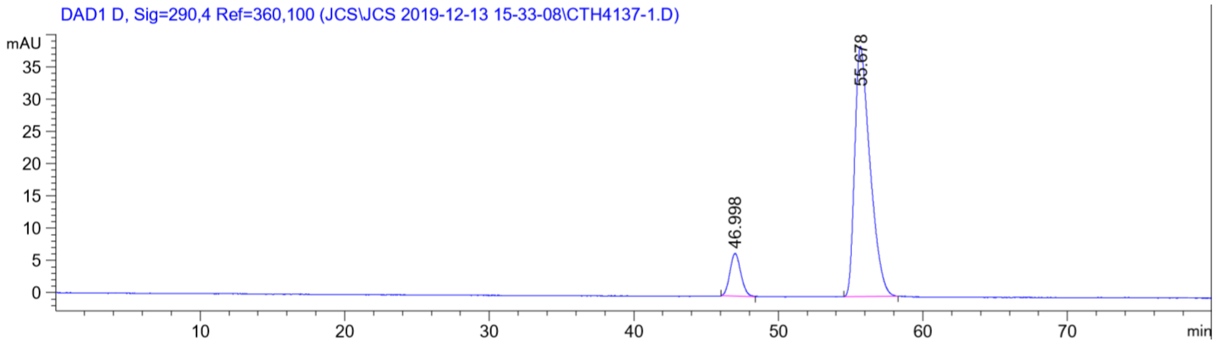

Signal 1: DAD1 D, Sig=290,4 Ref=360,100

Peak #	RetTime [min]	Type	Width [min]	Area [mAU*s]	Height [mAU]	Area %
1	46.998	BB	0.6533	346.46777	6.61275	10.7773
2	55.678	BB	1.0087	2868.31177	38.78782	89.2227

3,6-Difluorochromane (9)

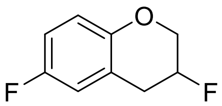

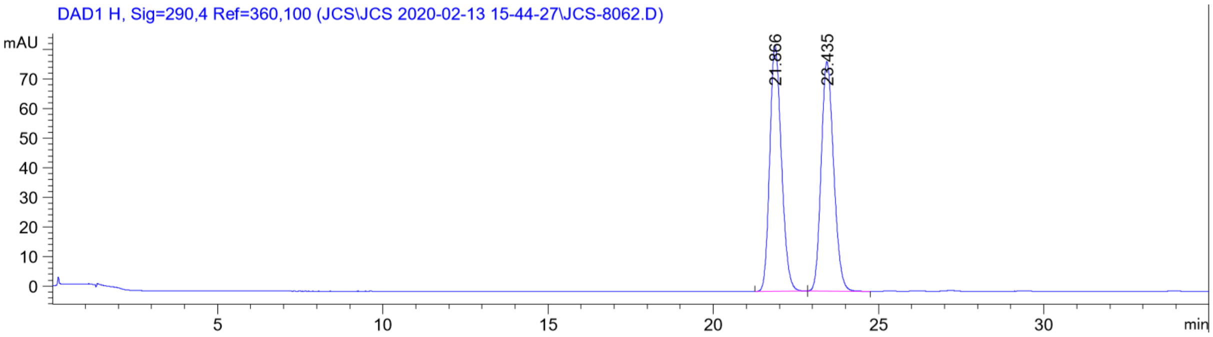

Signal 5: DAD1 H, Sig=290,4 Ref=360,100

Peak #	RetTime [min]	Type	Width [min]	Area [mAU*s]	Height [mAU]	Area %
1	21.866	BB	0.3808	2042.82300	83.10168	50.0413
2	23.435	BB	0.4104	2039.44934	77.64754	49.9587

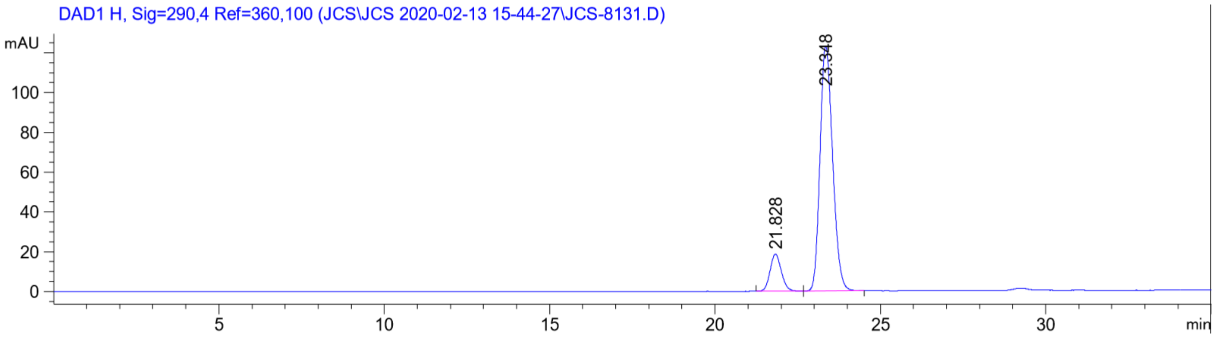

Signal 5: DAD1 H, Sig=290,4 Ref=360,100

Peak #	RetTime [min]	Type	Width [min]	Area [mAU*s]	Height [mAU]	Area %
1	21.828	BB	0.3711	442.52133	18.63452	11.9448
2	23.348	BB	0.4131	3262.18652	123.14571	88.0552

Methyl 3-fluorochromane-6-carboxylate (14)

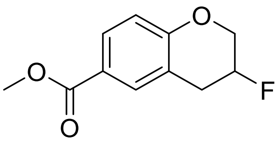

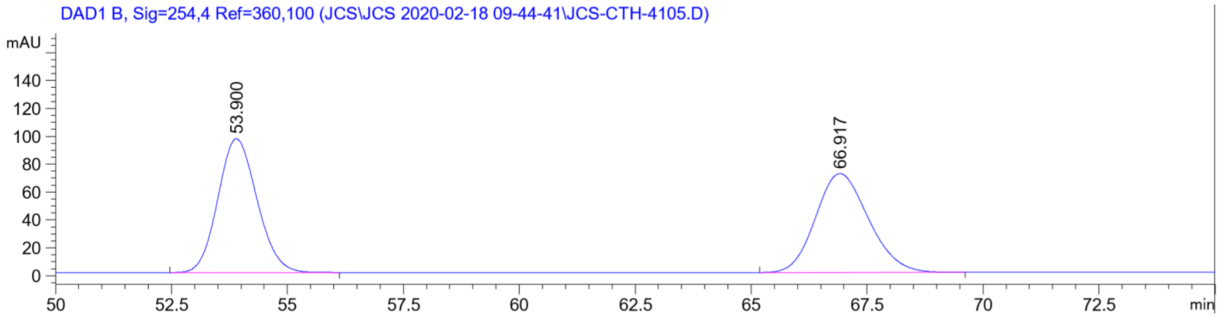

Signal 1: DAD1 B, Sig=254,4 Ref=360,100

Peak #	RetTime [min]	Type	Width [min]	Area [mAU*s]	Height [mAU]	Area %
1	53.900	BB	0.9027	5634.00391	95.99297	49.9941
2	66.917	BB	1.1611	5635.33252	70.91358	50.0059

Using amine:HF 1:5

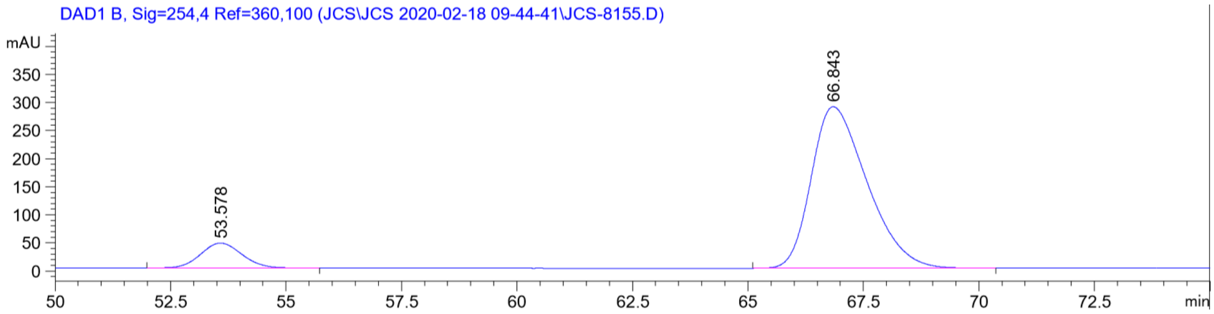

Signal 1: DAD1 B, Sig=254,4 Ref=360,100

Peak #	RetTime [min]	Type	Width [min]	Area [mAU*s]	Height [mAU]	Area %
1	53.578	BB	0.9514	2754.47632	44.29829	10.2305
2	66.843	BB	1.2578	2.41696e4	287.79660	89.7695

Methyl 3-fluorochromane-6-carboxylate (14)

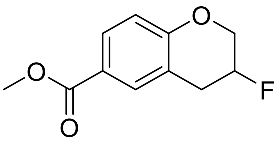

Using amine:HF 1:7.5

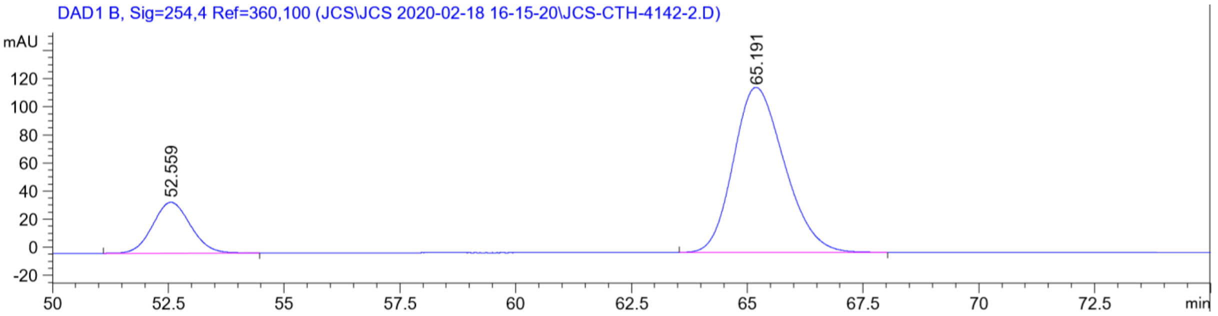

Signal 1: DAD1 B, Sig=254,4 Ref=360,100

Peak #	RetTime [min]	Type	Width [min]	Area [mAU*s]	Height [mAU]	Area %
1	52.559	BB	0.8457	2050.15918	36.14720	18.5487
2	65.191	BB	1.1678	9002.68359	117.50782	81.4513

(3-Fluorochroman-6-yl)(phenyl)methanone (15)

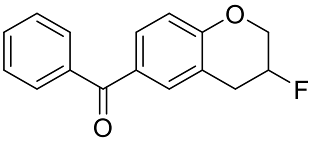

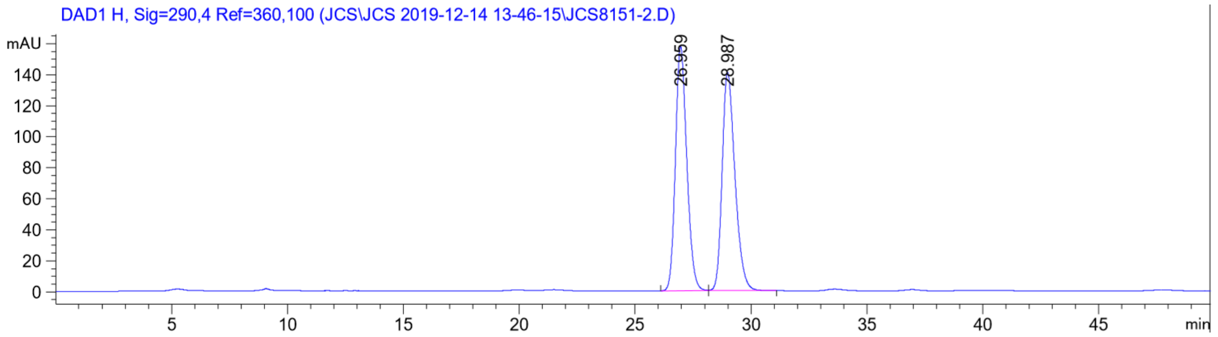

Signal 5: DAD1 H, Sig=290,4 Ref=360,100

Peak #	RetTime [min]	Type	Width [min]	Area [mAU*s]	Height [mAU]	Area %
1	26.959	BB	0.5203	5349.92676	157.49814	49.7931
2	28.987	BB	0.5855	5394.37695	140.64752	50.2069

(3-Fluorochroman-6-yl)(phenyl)methanone (15)

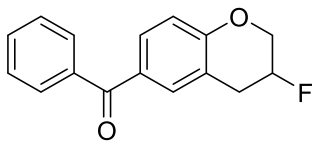

Using amine:HF 1:5

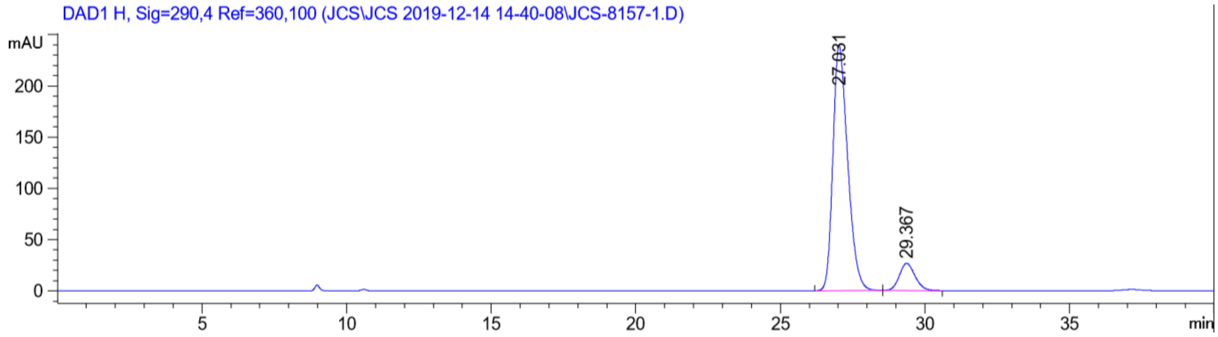

Signal 5: DAD1 H, Sig=290,4 Ref=360,100

Peak #	RetTime [min]	Type	Width [min]	Area [mAU*s]	Height [mAU]	Area %
1	27.031	BB	0.5444	8498.22168	239.25381	89.4656
2	29.367	BB	0.5825	1000.65375	26.50324	10.5344

Using amine:HF 1:7.5

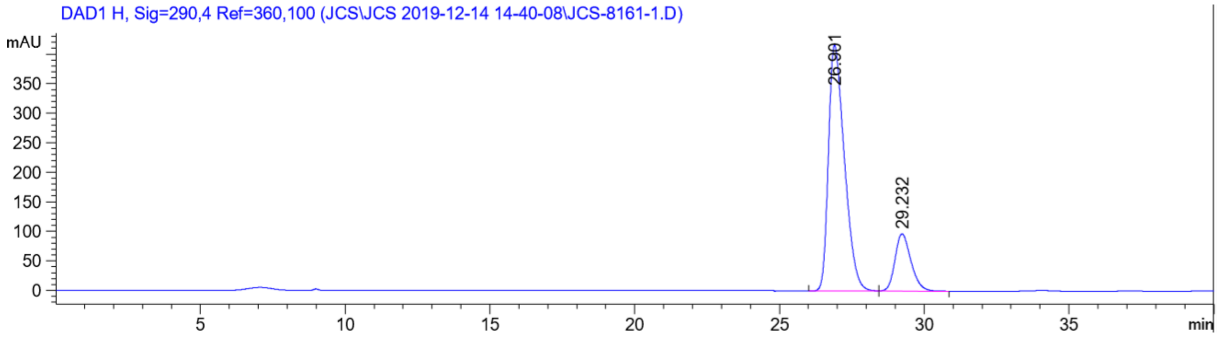

Signal 5: DAD1 H, Sig=290,4 Ref=360,100

Peak #	RetTime [min]	Type	Width [min]	Area [mAU*s]	Height [mAU]	Area %
1	26.901	BB	0.5794	1.59585e4	416.10547	81.2026
2	29.232	BB	0.5892	3694.19775	96.81865	18.7974

5,7-Dibromo-3-fluorochromane (10)

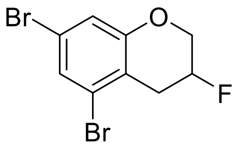

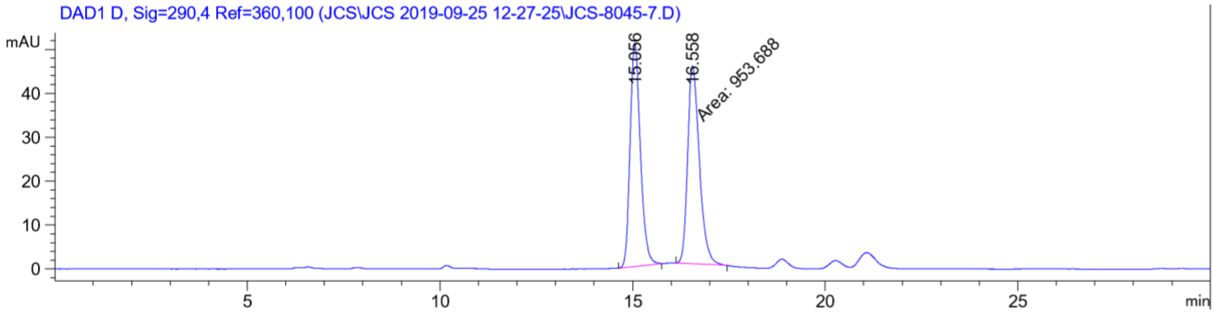

Signal 3: DAD1 D, Sig=290,4 Ref=360,100

Peak #	RetTime [min]	Type	Width [min]	Area [mAU*s]	Height [mAU]	Area %
1	15.056	BB	0.2891	942.82050	50.65966	49.7135
2	16.558	MM	0.3533	953.68799	44.98980	50.2865

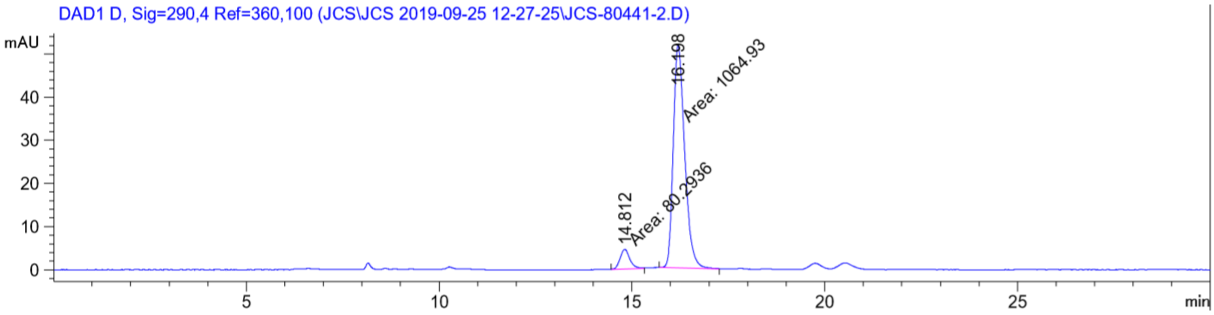

Signal 3: DAD1 D, Sig=290,4 Ref=360,100

Peak #	RetTime [min]	Type	Width [min]	Area [mAU*s]	Height [mAU]	Area %
1	14.812	MM	0.2936	80.29357	4.55814	7.0112
2	16.198	MM	0.3426	1064.93237	51.80264	92.9888

5,7-Dichloro-3-fluorochromane (11)

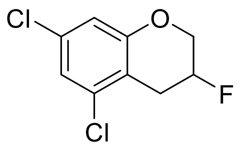

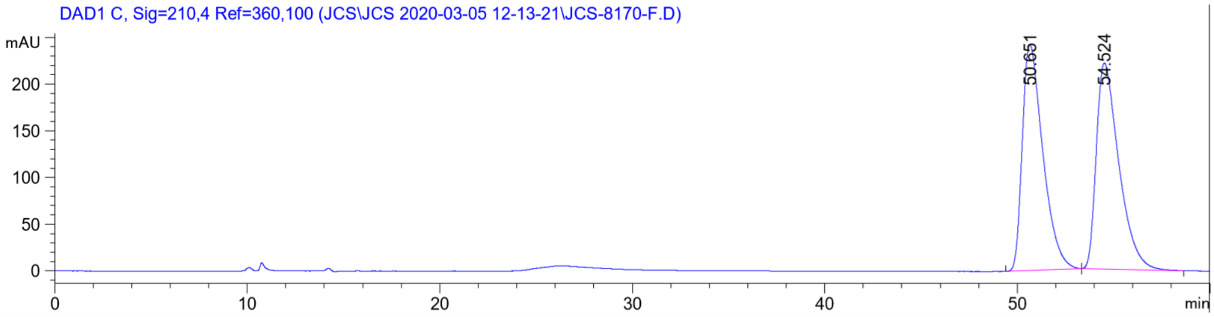

Signal 2: DAD1 C, Sig=210,4 Ref=360,100

Peak #	RetTime [min]	Type	Width [min]	Area [mAU*s]	Height [mAU]	Area %
1	50.651	BB	1.0976	1.79586e4	242.04683	49.9865
2	54.524	BB	1.2006	1.79683e4	220.52313	50.0135

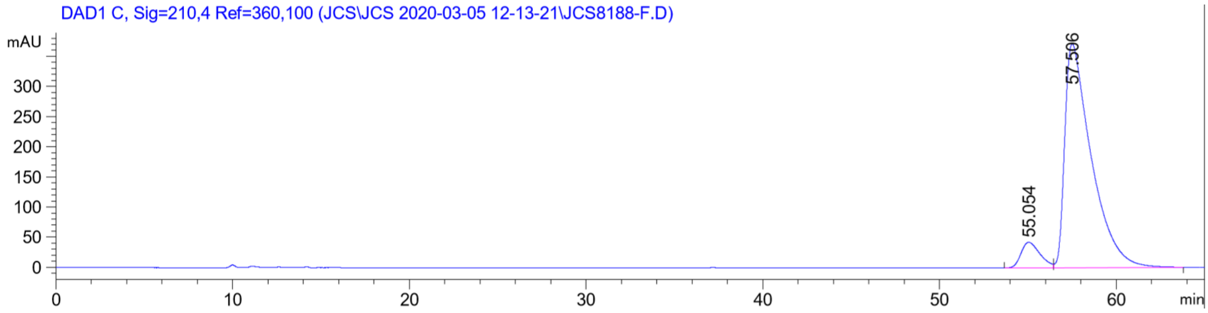

Signal 2: DAD1 C, Sig=210,4 Ref=360,100

Peak #	RetTime [min]	Type	Width [min]	Area [mAU*s]	Height [mAU]	Area %
1	55.054	BV	1.0645	3166.45630	42.43650	7.5617
2	57.506	VB	1.4929	3.87083e4	371.70303	92.4383

5,8-Dichloro-3-fluorochromane (12)

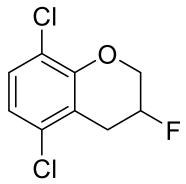

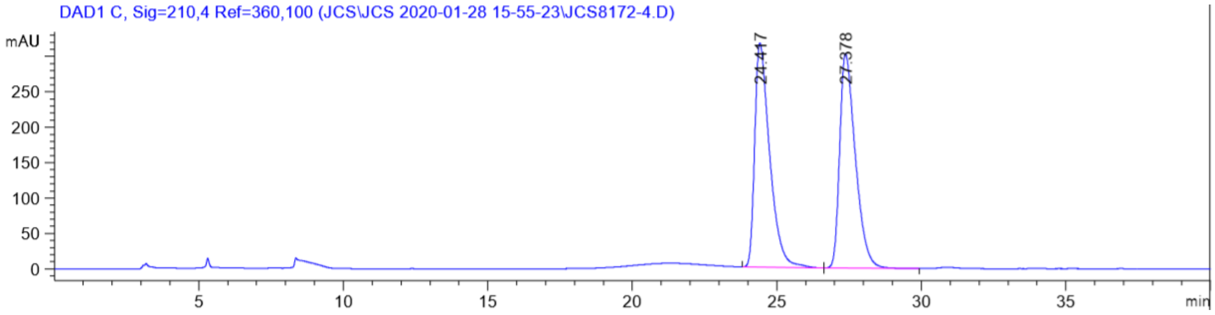

Signal 2: DAD1 C, Sig=210,4 Ref=360,100

Peak #	RetTime [min]	Type	Width [min]	Area [mAU*s]	Height [mAU]	Area %
1	24.417	BB	0.5384	1.14149e4	316.80066	49.9707
2	27.378	BB	0.5701	1.14283e4	302.94904	50.0293

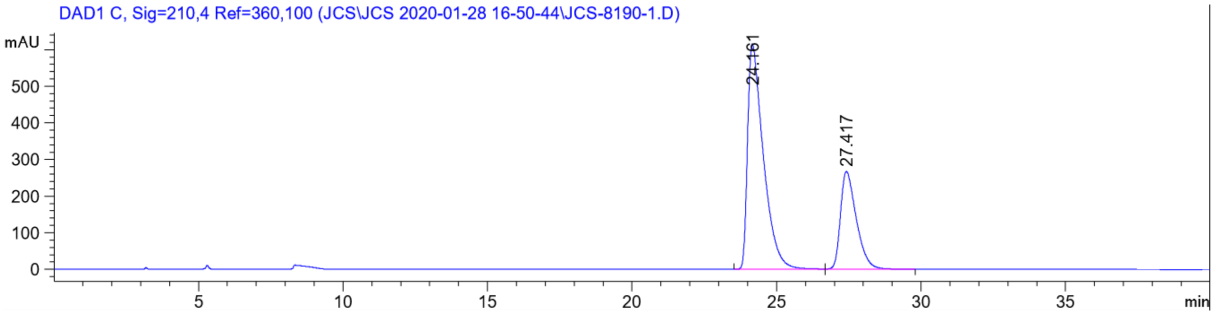

Signal 2: DAD1 C, Sig=210,4 Ref=360,100

Peak #	RetTime [min]	Type	Width [min]	Area [mAU*s]	Height [mAU]	Area %
1	24.161	BB	0.5574	2.32899e4	615.53326	69.6751
2	27.417	BB	0.5713	1.01365e4	266.76651	30.3249

3-Fluorochroman-6-yl methanesulfonate (13)

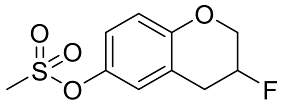

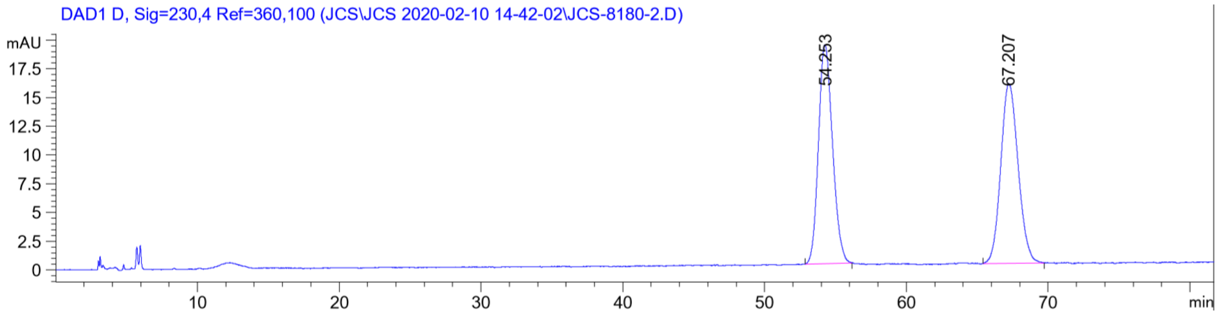

Signal 3: DAD1 D, Sig=230,4 Ref=360,100

Peak #	RetTime [min]	Type	Width [min]	Area [mAU*s]	Height [mAU]	Area %
1	54.253	BB	0.9678	1311.18665	19.03117	49.6843
2	67.207	BB	1.0046	1327.85132	15.63842	50.3157

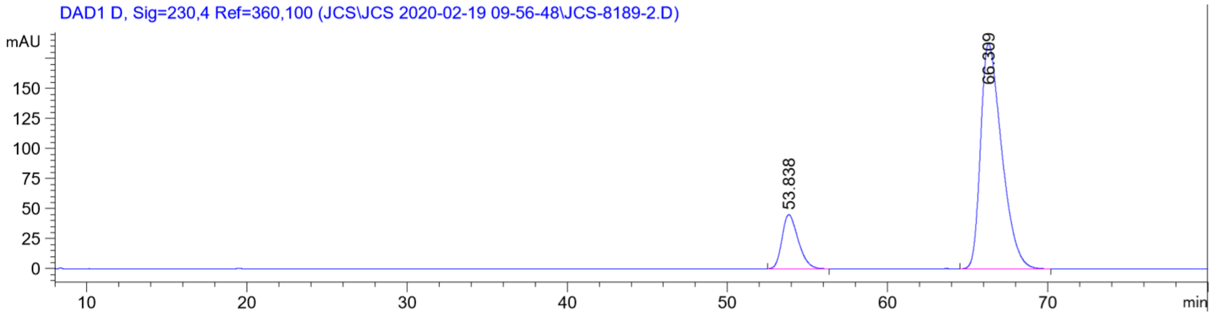

Signal 3: DAD1 D, Sig=230,4 Ref=360,100

Peak #	RetTime [min]	Type	Width [min]	Area [mAU*s]	Height [mAU]	Area %
1	53.838	BB	1.0190	3178.64453	45.05960	15.6120
2	66.309	BB	1.3488	1.71817e4	187.54198	84.3880

3-Fluoro-6-(trifluoromethyl)chromane (16)

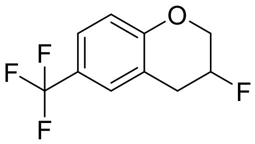

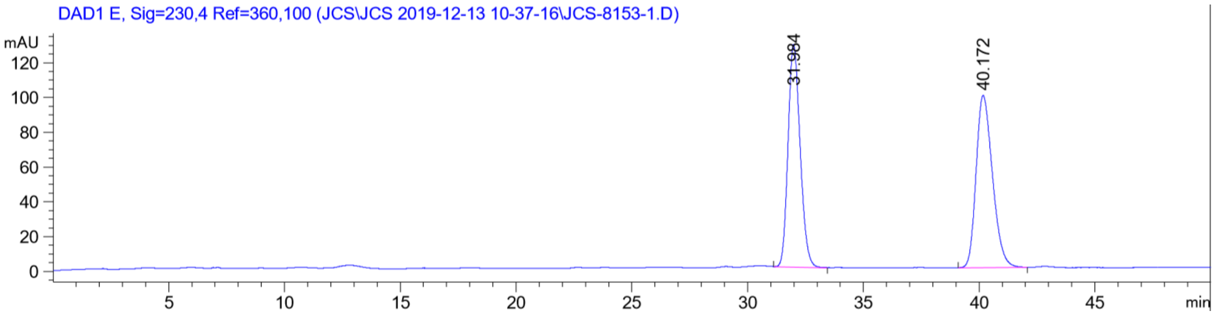

Signal 4: DAD1 E, Sig=230,4 Ref=360,100

Peak #	RetTime [min]	Type	Width [min]	Area [mAU*s]	Height [mAU]	Area %
1	31.984	BB	0.5580	4629.34766	127.98323	48.7752
2	40.172	BB	0.7425	4861.85254	99.06895	51.2248

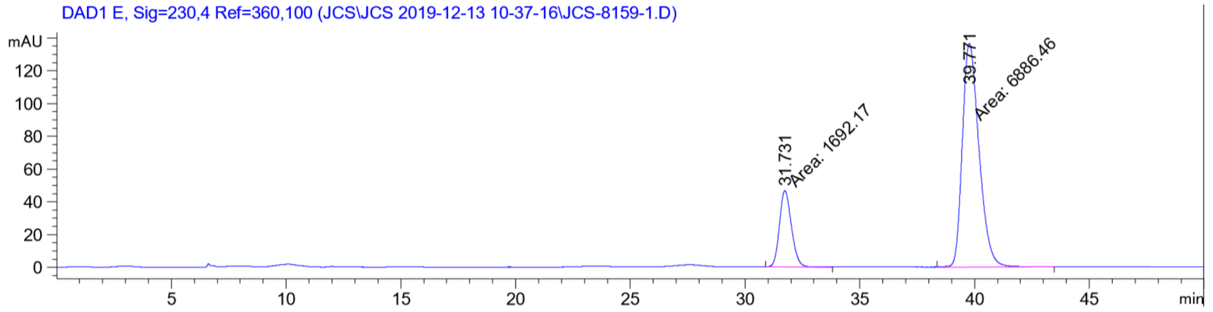

Signal 4: DAD1 E, Sig=230,4 Ref=360,100

Peak #	RetTime [min]	Type	Width [min]	Area [mAU*s]	Height [mAU]	Area %
1	31.731	MM	0.6045	1692.16882	46.65633	19.7254
2	39.771	MM	0.8404	6886.46045	136.56662	80.2746

3-Fluorochromane-6-carbonitrile (17)

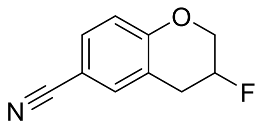

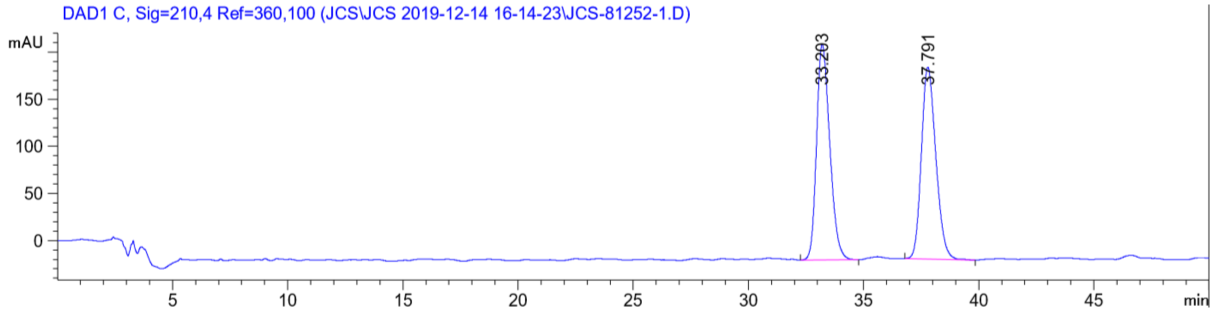

Signal 2: DAD1 C, Sig=210,4 Ref=360,100

Peak #	RetTime [min]	Type	Width [min]	Area [mAU*s]	Height [mAU]	Area %
1	33.203	BB	0.6150	9136.87793	229.22330	50.7360
2	37.791	BB	0.6665	8871.79785	203.56197	49.2640

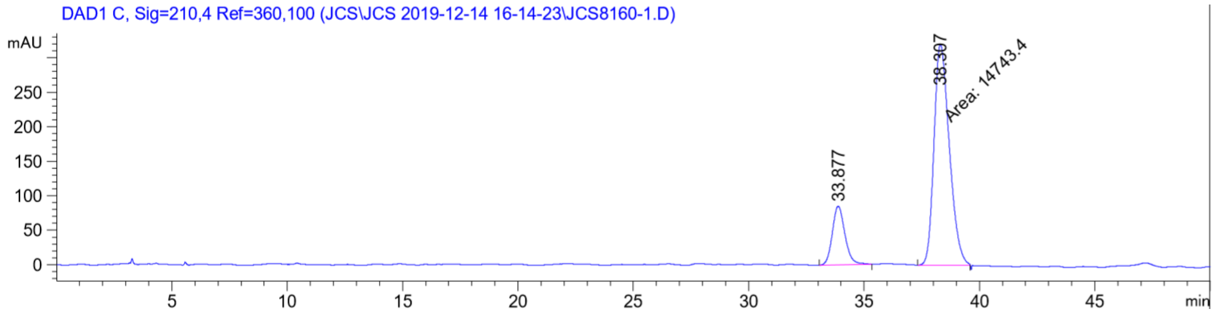

Signal 2: DAD1 C, Sig=210,4 Ref=360,100

Peak #	RetTime [min]	Type	Width [min]	Area [mAU*s]	Height [mAU]	Area %
1	33.877	BB	0.5785	3228.38574	85.11553	17.9637
2	38.307	MM	0.7679	1.47434e4	319.98148	82.0363

3-Fluorochromane-6-carbaldehyde (19)

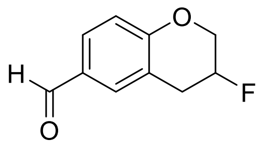

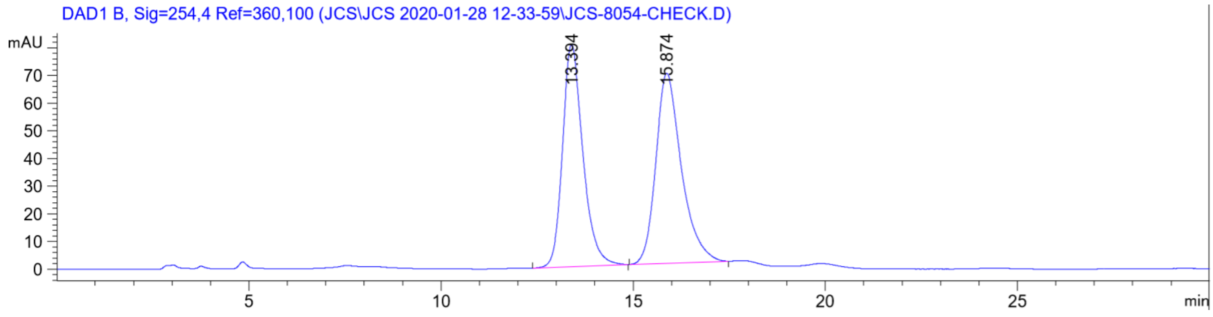

Signal 1: DAD1 B, Sig=254,4 Ref=360,100

Peak #	RetTime [min]	Type	Width [min]	Area [mAU*s]	Height [mAU]	Area %
1	13.394	BB	0.5548	2956.87305	80.44737	48.1701
2	15.874	BB	0.6863	3181.52490	68.70279	51.8299

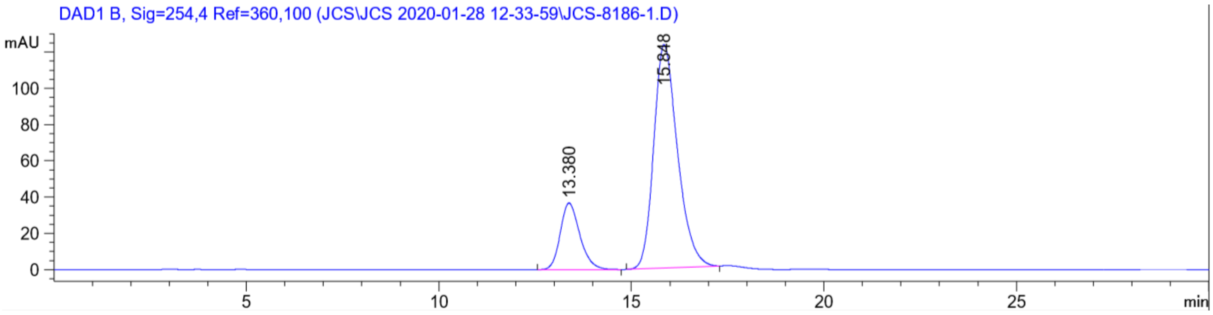

Signal 1: DAD1 B, Sig=254,4 Ref=360,100

Peak #	RetTime [min]	Type	Width [min]	Area [mAU*s]	Height [mAU]	Area %
1	13.380	BB	0.5430	1303.35339	36.81694	19.7494
2	15.848	BB	0.6531	5296.10986	123.31291	80.2506

3-Fluoro-6-(methylsulfonyl)chromane (18)

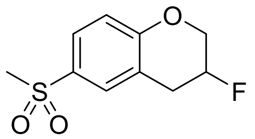

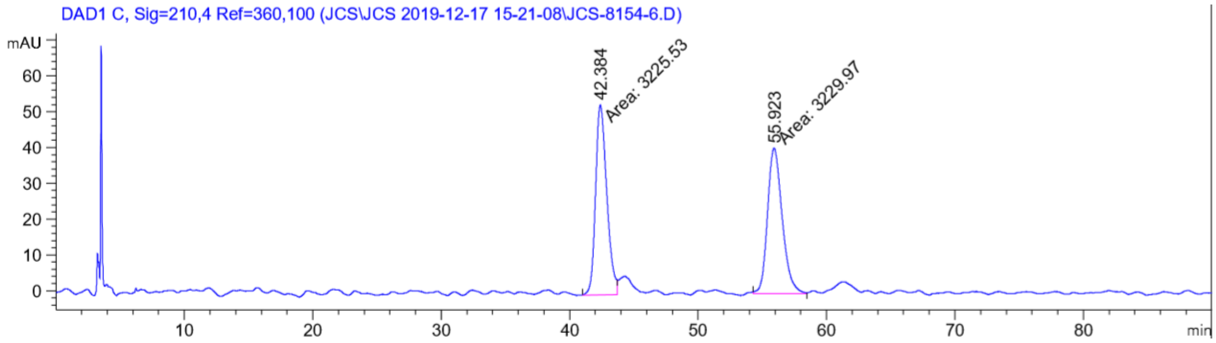

Signal 1: DAD1 C, Sig=210,4 Ref=360,100

Peak #	RetTime [min]	Type	Width [min]	Area [mAU*s]	Height [mAU]	Area %
1	42.384	MF	1.0136	3225.52905	53.03773	49.9656
2	55.923	MM	1.3233	3229.97070	40.68118	50.0344

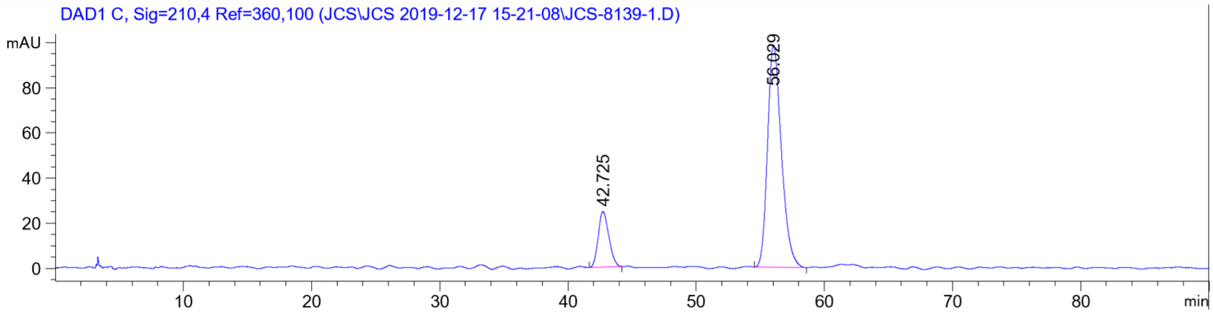

Signal 2: DAD1 C, Sig=210,4 Ref=360,100

Peak #	RetTime [min]	Type	Width [min]	Area [mAU*s]	Height [mAU]	Area %
1	42.725	BB	0.7986	1424.04761	24.70261	16.1150
2	56.029	BB	1.0770	7412.73389	98.60779	83.8850

6-Nitro-3-fluorochromane (20)

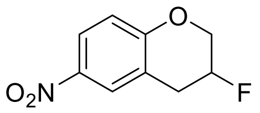

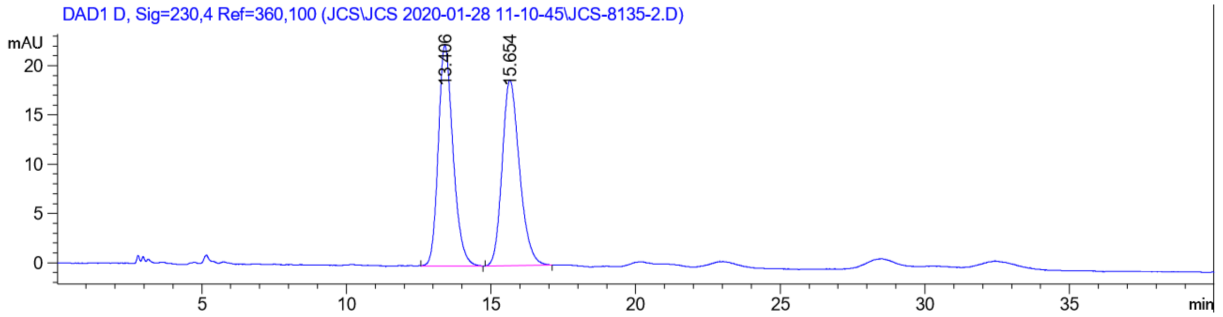

Signal 1: DAD1 D, Sig=230,4 Ref=360,100

Peak #	RetTime [min]	Type	Width [min]	Area [mAU*s]	Height [mAU]	Area %
1	13.406	BB	0.5399	794.98340	22.40703	50.2984
2	15.654	BB	0.6246	785.55029	18.83563	49.7016

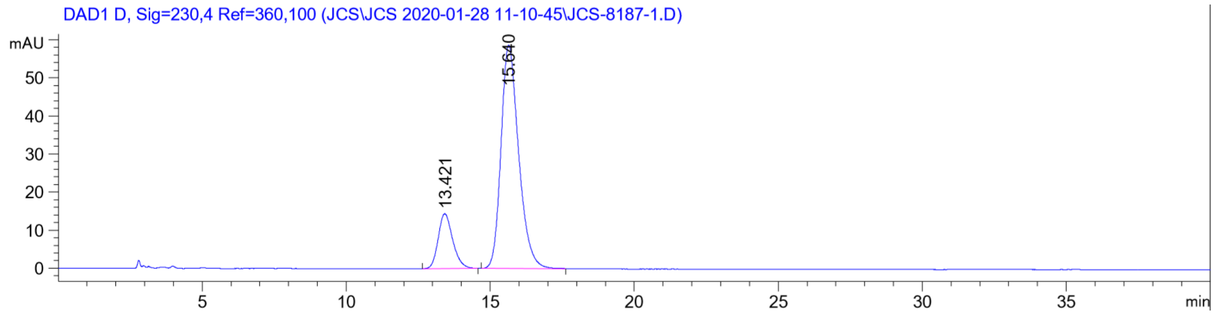

Signal 3: DAD1 D, Sig=230,4 Ref=360,100

Peak #	RetTime [min]	Type	Width [min]	Area [mAU*s]	Height [mAU]	Area %
1	13.421	BB	0.5244	507.23315	14.42044	16.8884
2	15.640	BB	0.6382	2496.20190	58.69547	83.1116

6-Bromo-3-fluoro-3-methylchromane (21)

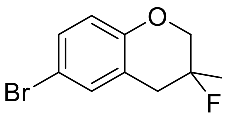

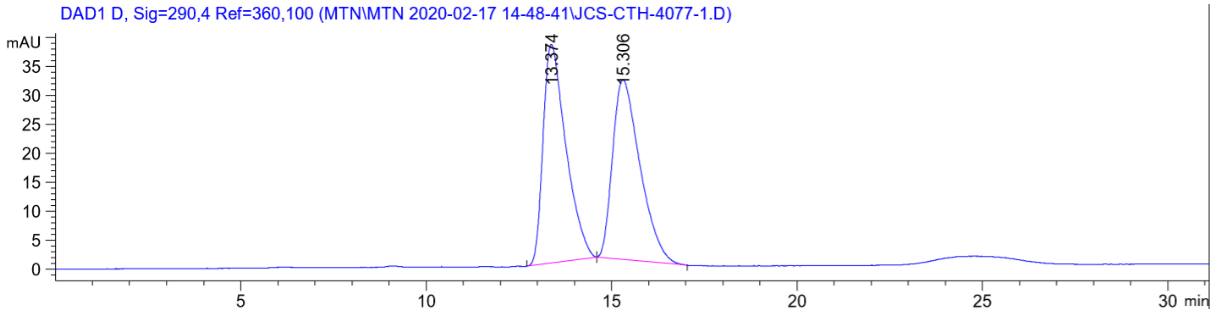

Signal 3: DAD1 D, Sig=290,4 Ref=360,100

Peak #	RetTime [min]	Type	Width [min]	Area [mAU*s]	Height [mAU]	Area %
1	13.374	BB	0.6354	1615.05005	37.73263	50.2851
2	15.306	BB	0.7644	1596.73486	31.02749	49.7149

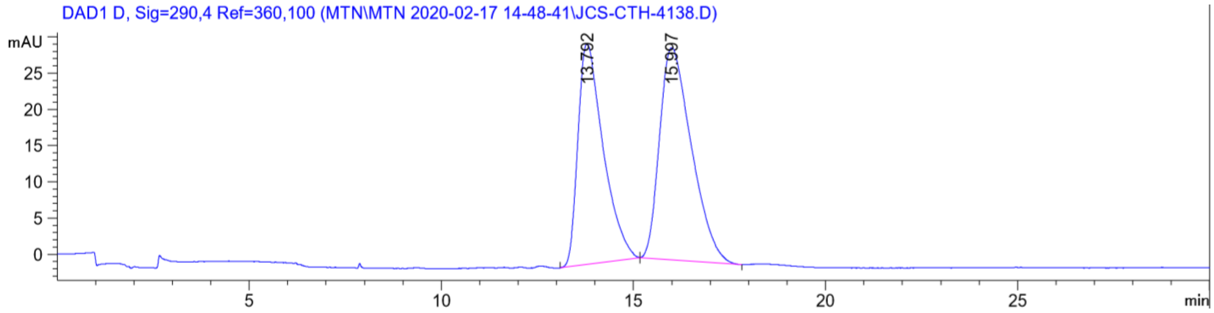

Signal 3: DAD1 D, Sig=290,4 Ref=360,100

Peak #	RetTime [min]	Type	Width [min]	Area [mAU*s]	Height [mAU]	Area %
1	13.792	BB	0.6702	1394.72461	30.46194	45.2079
2	15.997	BB	0.7940	1690.41309	29.26051	54.7921

Mixture of 32 and *ent*-32

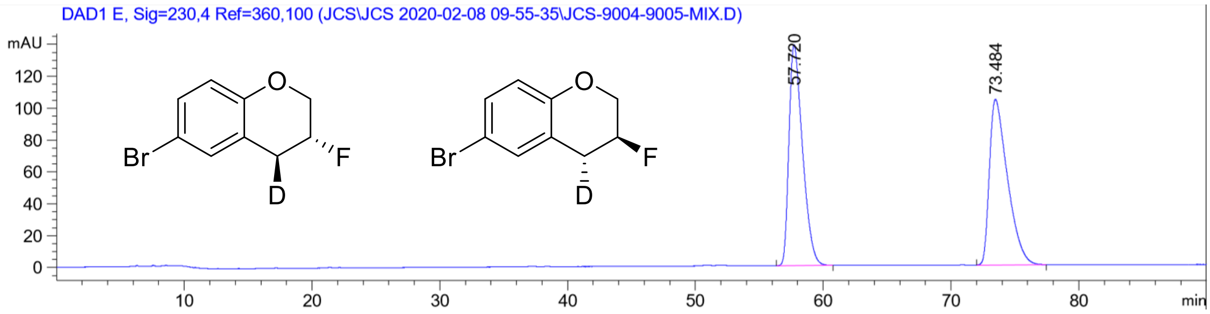

(3*R*,4*S*)-6-Bromo-3-fluorochromane-4-*d* (32)

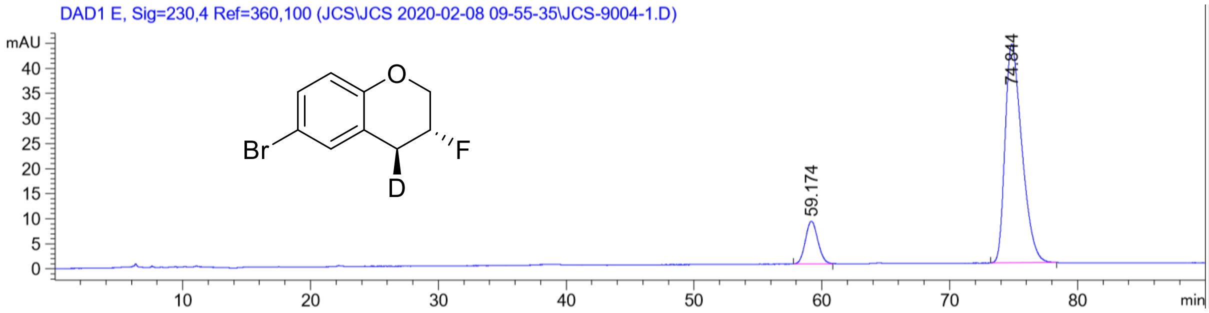

Signal 4: DAD1 E, Sig=230,4 Ref=360,100

Peak #	RetTime [min]	Type	Width [min]	Area [mAU*s]	Height [mAU]	Area %
1	59.174	BB	0.8156	574.35455	8.50839	12.5611
2	74.844	BB	1.2715	3998.11816	43.61361	87.4389

(3*S*,4*R*)-6-Bromo-3-fluorochromane-4-*d* (*ent*-32)

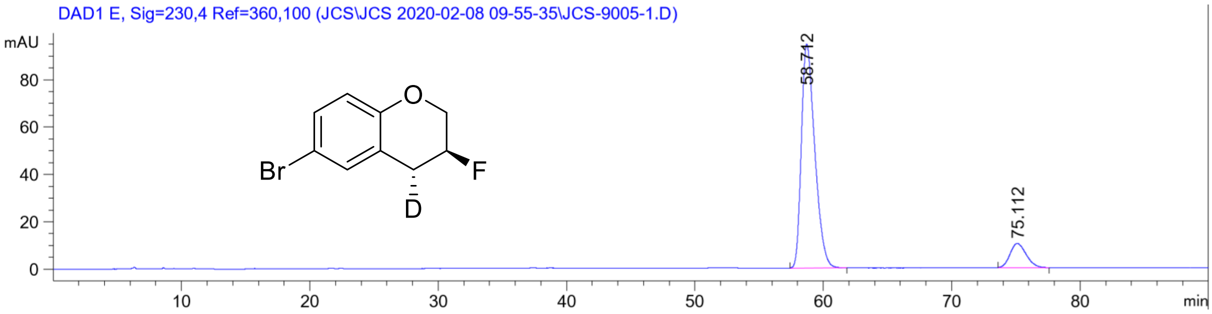

Signal 4: DAD1 E, Sig=230,4 Ref=360,100

Peak #	RetTime [min]	Type	Width [min]	Area [mAU*s]	Height [mAU]	Area %
1	58.712	BB	1.0793	6834.13525	94.54738	88.4706
2	75.112	BB	1.0392	890.61615	10.21480	11.5294

Mixture of 33 and *ent*-33

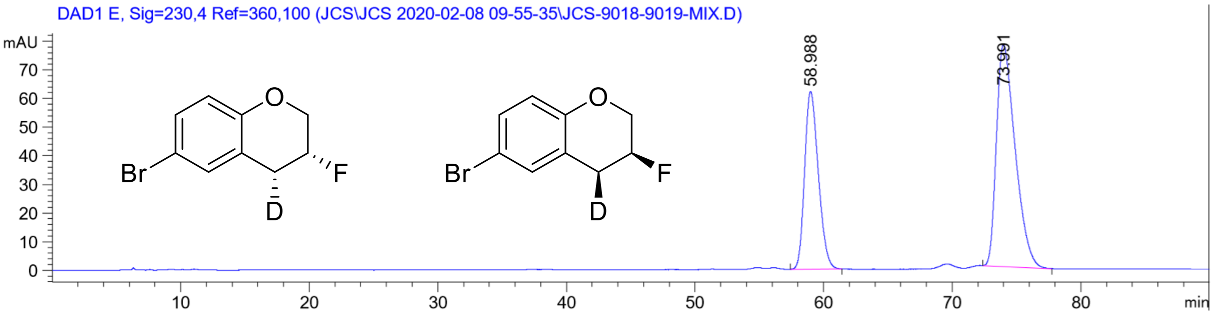

(3*R*,4*R*)-6-Bromo-3-fluorochromane-4-*d* (33)

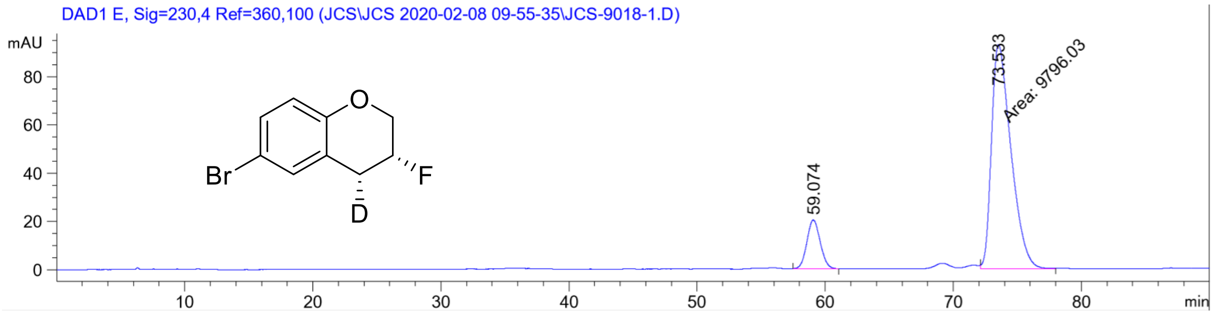

Signal 4: DAD1 E, Sig=230,4 Ref=360,100

Peak #	RetTime [min]	Type	Width [min]	Area [mAU*s]	Height [mAU]	Area %
1	59.074	BB	0.9710	1459.87207	20.25096	12.9698
2	73.533	FM	1.7569	9796.03027	92.92681	87.0302

(3*S*,4*S*)-6-Bromo-3-fluorochromane-4-*d* (*ent*-33)

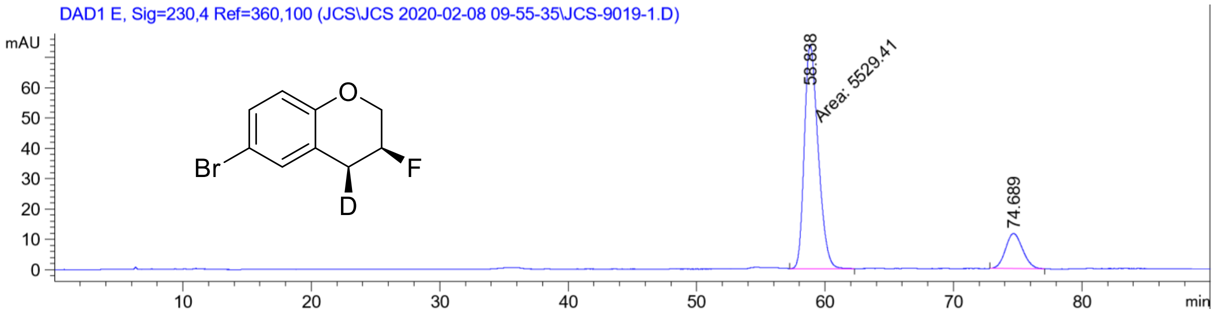

Signal 4: DAD1 E, Sig=230,4 Ref=360,100

Peak #	RetTime [min]	Type	Width [min]	Area [mAU*s]	Height [mAU]	Area %
1	58.838	MM	1.2457	5529.41064	73.97756	83.7340
2	74.689	BB	1.0998	1074.13306	11.58347	16.2660

5. X-ray Analysis

Data sets for compounds **17** and **20** were collected with a Bruker D8 Venture CMOS diffractometer. Programs used: data collection: APEX3 V2016.1-0 (Bruker AXS Inc., **2016**); cell refinement: SAINT V8.37A (Bruker AXS Inc., **2015**); data reduction: SAINT V8.37A (Bruker AXS Inc., **2015**); absorption correction, SADABS V2014/7 (Bruker AXS Inc., **2014**); structure solution *SHELXT-2015* (Sheldrick, G. M. *Acta Cryst.,* **2015**, *A71*, 3-8); structure refinement *SHELXL-2015* (Sheldrick, G. M. *Acta Cryst.,* **2015**, *C71* (1), 3-8) and graphics, *XP* (Version 5.1, Bruker AXS Inc., Madison, Wisconsin, USA, **1998**). For compounds **7** and **21** data sets were collected with a Nonius Kappa CCD diffractometer. Programs used: data collection, COLLECT (R. W. W. Hooft, Bruker AXS, **2008**, Delft, The Netherlands); data reduction Denzo-SMN (Z. Otwinowski, W. Minor, *Methods Enzymol.* **1997**, *276*, 307-326); absorption correction, Denzo (Z. Otwinowski, D. Borek, W. Majewski, W. Minor, *Acta Crystallogr.* **2003**, *A59*, 228-234); structure solution SHELXT-2015; structure refinement SHELXL-2015. *R*-values are given for observed reflections, and wR^2 values are given for all reflections.

Exceptions and special features: In the asymmetric unit of compound **7** six badly disordered methanol molecules were found and could not be satisfactorily refined. The program SQUEEZE (A. L. Spek (**2015)** Acta Cryst., C71, 9-18) was therefore used to remove mathematically the effect of the solvents.

X-ray crystal structure analysis of 7 (gil8880): A colorless prism-like specimen of $C_{27}H_{29}IN_2O_4$, approximate dimensions 0.080 mm x 0.120 mm x 0.120 mm, was used for the X-ray crystallographic analysis. The X-ray intensity data were measured. The integration of the data using a trigonal unit cell yielded a total of 15870 reflections to a maximum θ angle of 25.00° (0.84 Å resolution), of which 15870 were independent (average redundancy 1.000, completeness = 99.5%, R_{int} = 5.10%, R_{sig} = 2.72%) and 15358 (96.77%) were greater than $2\sigma(F^2)$. The final cell constants of a = 22.9741(3) Å, b = 22.9741(3) Å, c = 15.1323(2) Å, volume = 6916.9(2) Å^3, are based upon the refinement of the XYZ-centroids of reflections above 20 σ(I). Data were corrected for absorption effects using the multi-scan method (SADABS). The calculated minimum and maximum transmission coefficients (based on crystal size) are 0.8820 and 0.9190. The structure was solved and refined using the Bruker SHELXTL Software Package, using the space group $P3_2$, with Z = 9 for the formula unit, $C_{27}H_{29}IN_2O_4$. The final anisotropic full-matrix least-squares refinement on F^2 with 1003 variables converged at R1 = 5.20%, for the observed data and wR2 = 13.71% for all data. The goodness-of-fit was 1.029. The largest peak in the final difference electron density synthesis was 0.687 $e^-/Å^3$ and the largest hole was -0.688 $e^-/Å^3$ with an RMS deviation of 0.058 $e^-/Å^3$. On the basis of the final model, the calculated density was 1.237 g/cm^3 and F(000), 2610 e^-. The hydrogens at N1 and N2 atoms were refined freely, but with N-H distance restraints (DFIX and U-fixed value). Flack parameter was refined to 0.01(2). CCDC number: 1995718.

X-ray crystal structure analysis of 17 (gil9775): A colorless plate-like specimen of $C_{10}H_8FNO$, approximate dimensions 0.038 mm x 0.208 mm x 0.278 mm, was used for the X-ray crystallographic analysis. The X-ray intensity data were measured on a Bruker D8 Venture PHOTON III Diffractometer system equipped with a micro focus tube Cu Ims (CuKα, λ = 1.54178 Å) and a MX mirror monochromator. A total of 890 frames were collected. The total exposure time was 16.00 hours. The frames were integrated with the Bruker SAINT software package using a wide-frame algorithm. The integration of the data using an orthorhombic unit cell yielded a total of 5976 reflections to a maximum θ angle of 68.43° (0.83 Å resolution), of which 1451 were independent (average redundancy 4.119, completeness = 98.7%, R_{int} = 8.22%, R_{sig} = 7.61%) and 1185 (81.67%) were greater than $2\sigma(F^2)$. The final cell constants of a = 5.9104(3) Å, b = 7.2446(5) Å, c = 19.3965(13) Å, volume = 830.53(9) Å^3, are based upon the

refinement of the XYZ-centroids of 2605 reflections above 20 σ(I) with 9.116° < 2θ < 136.1°. Data were corrected for absorption effects using the Multi-Scan method (SADABS). The ratio of minimum to maximum apparent transmission was 0.651. The calculated minimum and maximum transmission coefficients (based on crystal size) are 0.7870 and 0.9660. The structure was solved and refined using the Bruker SHELXTL Software Package, using the space group $P2_12_12_1$, with Z = 4 for the formula unit, $C_{10}H_8FNO$. The final anisotropic full-matrix least-squares refinement on F^2 with 118 variables converged at R1 = 4.67%, for the observed data and wR2 = 11.69% for all data. The goodness-of-fit was 1.076. The largest peak in the final difference electron density synthesis was 0.161 $e^-/Å^3$ and the largest hole was -0.166 $e^-/Å^3$ with an RMS deviation of 0.043 $e^-/Å^3$. On the basis of the final model, the calculated density was 1.417 g/cm^3 and F(000), 368 e^-. Flack parameter was refined to 0.1(3). CCDC number: 1995719.

X-ray crystal structure analysis of 20 (gil9408): A colorless plate-like specimen of $C_9H_8FNO_3$, approximate dimensions 0.046 mm x 0.220 mm x 0.394 mm, was used for the X-ray crystallographic analysis. The X-ray intensity data were measured. A total of 1538 frames were collected. The total exposure time was 29.30 hours. The frames were integrated with the Bruker SAINT software package using a wide-frame algorithm. The integration of the data using an orthorhombic unit cell yielded a total of 5207 reflections to a maximum θ angle of 66.91° (0.84 Å resolution), of which 1451 were independent (average redundancy 3.589, completeness = 98.0%, R_{int} = 5.89%, R_{sig} = 5.30%) and 1313 (90.49%) were greater than 2σ(F^2). The final cell constants of a = 6.2125(4) Å, b = 7.0429(4) Å, c = 19.1857(12) Å, volume = 839.45(9) Å^3, are based upon the refinement of the XYZ-centroids of 354 reflections above 20 σ(I) with 0.930° < 2θ < 64.53°. Data were corrected for absorption effects using the multi-scan method (SADABS). The ratio of minimum to maximum apparent transmission was 0.695. The calculated minimum and maximum transmission coefficients (based on crystal size) are 0.6620 and 0.9490. The structure was solved and refined using the Bruker SHELXTL Software Package, using the space group $P2_12_12_1$, with Z = 4 for the formula unit, $C_9H_8FNO_3$. The final anisotropic full-matrix least-squares refinement on F^2 with 127 variables converged at R1 = 4.43%, for the observed data and wR2 = 11.61% for all data. The goodness-of-fit was 1.060. The largest peak in the final difference electron density synthesis was 0.208 $e^-/Å^3$ and the largest hole was -0.195 $e^-/Å^3$ with an RMS deviation of 0.055 $e^-/Å^3$. On the basis of the final model, the calculated density was 1.560 g/cm^3 and F(000), 408 e^-. Flack parameter was refined to 0.1(2). CCDC number: 1995720.

X-ray crystal structure analysis of 21 (gil9428): A colorless plate-like specimen of $C_{10}H_{10}BrFO$, approximate dimensions 0.040 mm x 0.100 mm x 0.130 mm, was used for the X-ray crystallographic analysis. The X-ray intensity data were measured. The integration of the data using a monoclinic unit cell yielded a total of 3073 reflections to a maximum θ angle of 26.37° (0.80 Å resolution), of which 1890 were independent (average redundancy 1.626, completeness = 97.1%, R_{int} = 3.79%, R_{sig} = 3.68%) and 1752 (92.70%) were greater than 2σ(F^2). The final cell constants of a = 14.5930(4) Å, b = 5.32930(10) Å, c = 12.1952(3) Å, β = 91.6280(10)°, volume = 948.04(4) Å^3, are based upon the refinement of the XYZ-centroids of reflections above 20 σ(I). Data were corrected for absorption effects using the multi-scan method (SADABS). The calculated minimum and maximum transmission coefficients (based on crystal size) are 0.6040 and 0.8470. The structure was solved and refined using the Bruker SHELXTL Software Package, using the space group $P2_1/c$, with Z = 4 for the formula unit, $C_{10}H_{10}BrFO$. The final anisotropic full-matrix least-squares refinement on F^2 with 119 variables converged at R1 = 3.16%, for the observed data and wR2 = 8.35% for all data. The goodness-of-fit was 1.087. The largest peak in the final difference electron density synthesis was 0.356 $e^-/Å^3$ and the largest hole was -0.418 $e^-/Å^3$ with an RMS deviation of 0.087 $e^-/Å^3$. On the basis

of the final model, the calculated density was 1.717 g/cm^3 and F(000), 488 e$^-$. CCDC number: 1995721.

1. *APEX3* (**2016**), *SAINT* (**2015**) and *SADABS* (**2015**), Bruker AXS Inc., Madison, Wisconsin, USA.
2. Sheldrick, G. M., *SHELXT – Integrated space-group and crystal-structure determination*, *Acta Cryst.,* **2015**, *A71*, 3-8.
3. Sheldrick, G.M., *Crystal structure refinement with SHELXL*, *Acta Cryst.*, **2015**, *C71 (1)*, 3-8.
4. *XP – Interactive molecular graphics, Version 5.1,* Bruker AXS Inc., Madison, Wisconsin, USA, **1998**.
5. Hooft, R. W. W., Nonius B. V., *COLLECT, Program for Collecting Data on CCD Area Detectors*, **1998**, Delft, The Netherlands.
6. Z. Otwinowski, W. Minor, *Methods Enzymol.* **1997**, 276, 307 – 326.
7. Z. Otwinowski, D. Borek, W. Majewski, W. Minor, *Acta Crystallogr. Sect. A* **2003**, 59, 228 – 234.

6. NMR Spectra

a. NMR Spectra of Catalysts and Phenyl Allyl Ethers

Methyl (*R*)-2-(2-iodophenoxy)-3-phenylpropanoate (S6)

^{1}H NMR (400 MHz, $CDCl_3$):

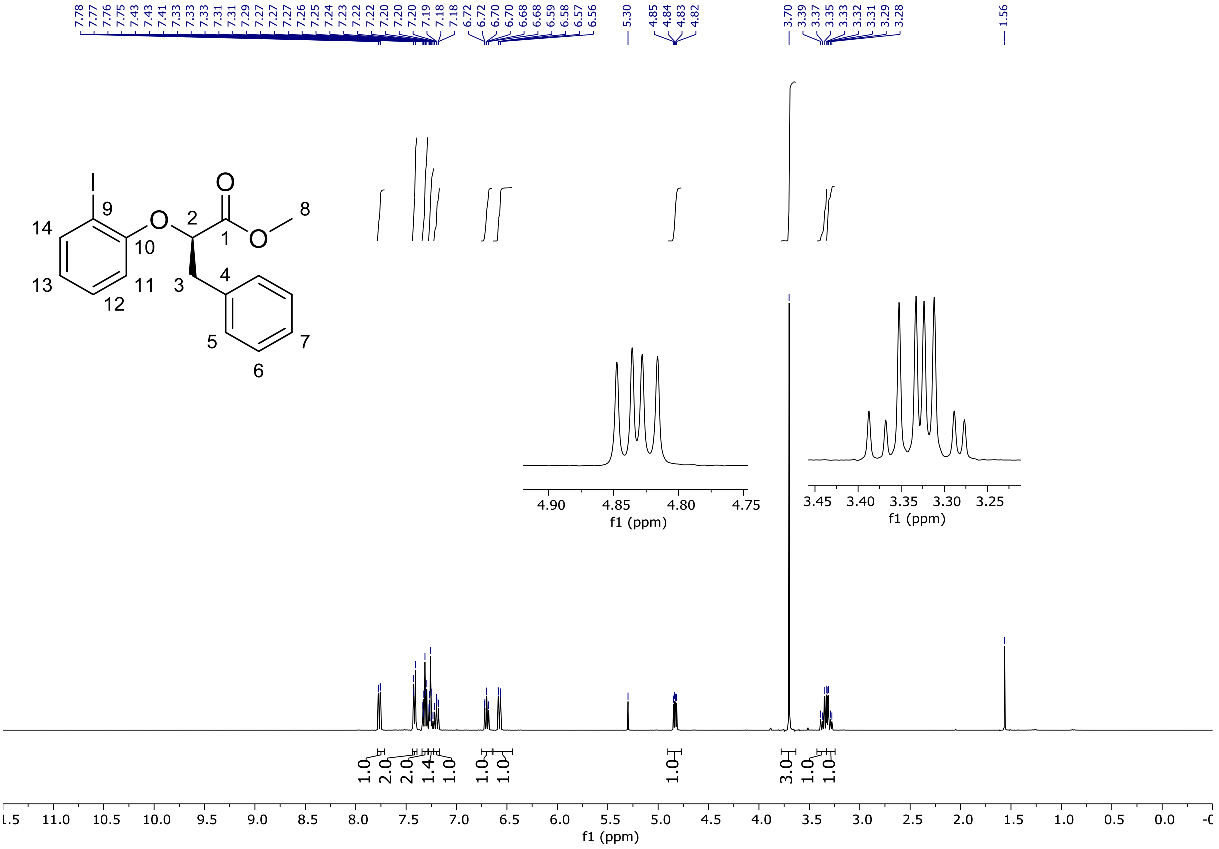

$^{13}C\{^{1}H\}$ NMR (101 MHz, $CDCl_3$):

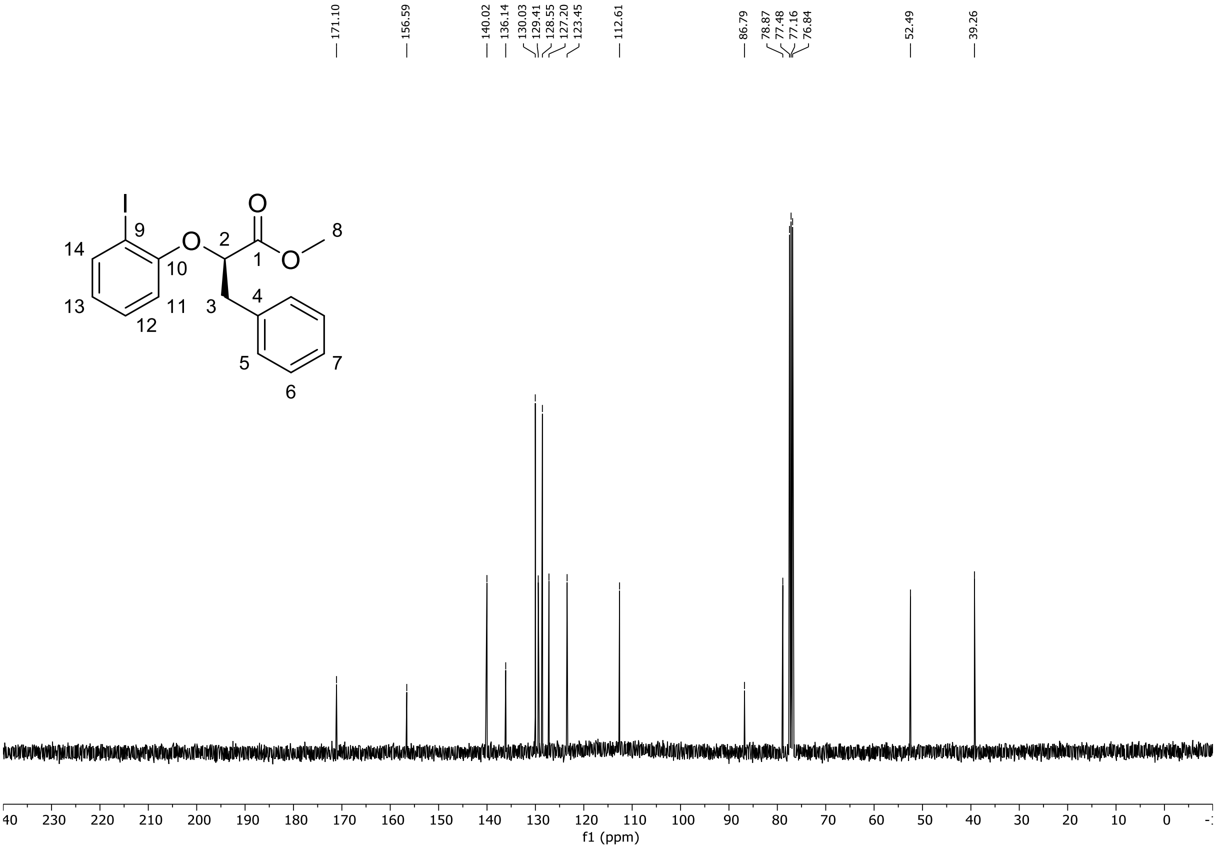

(*R*)-2-(2-Iodophenoxy)-*N*-methyl-3-phenylpropanamide (*S7*)

^{1}H NMR (500 MHz, CDCl$_3$):

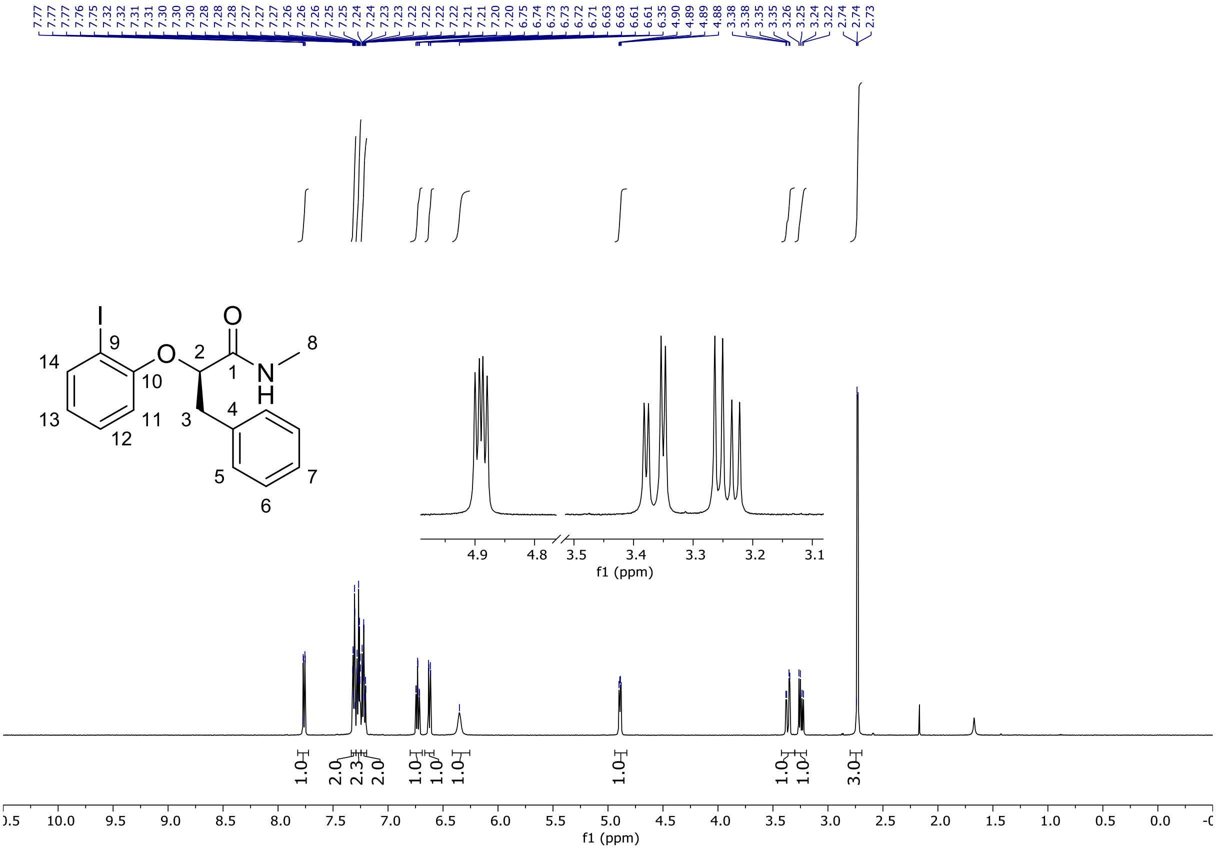

^{13}C{^{1}H} NMR (126 MHz, CDCl$_3$):

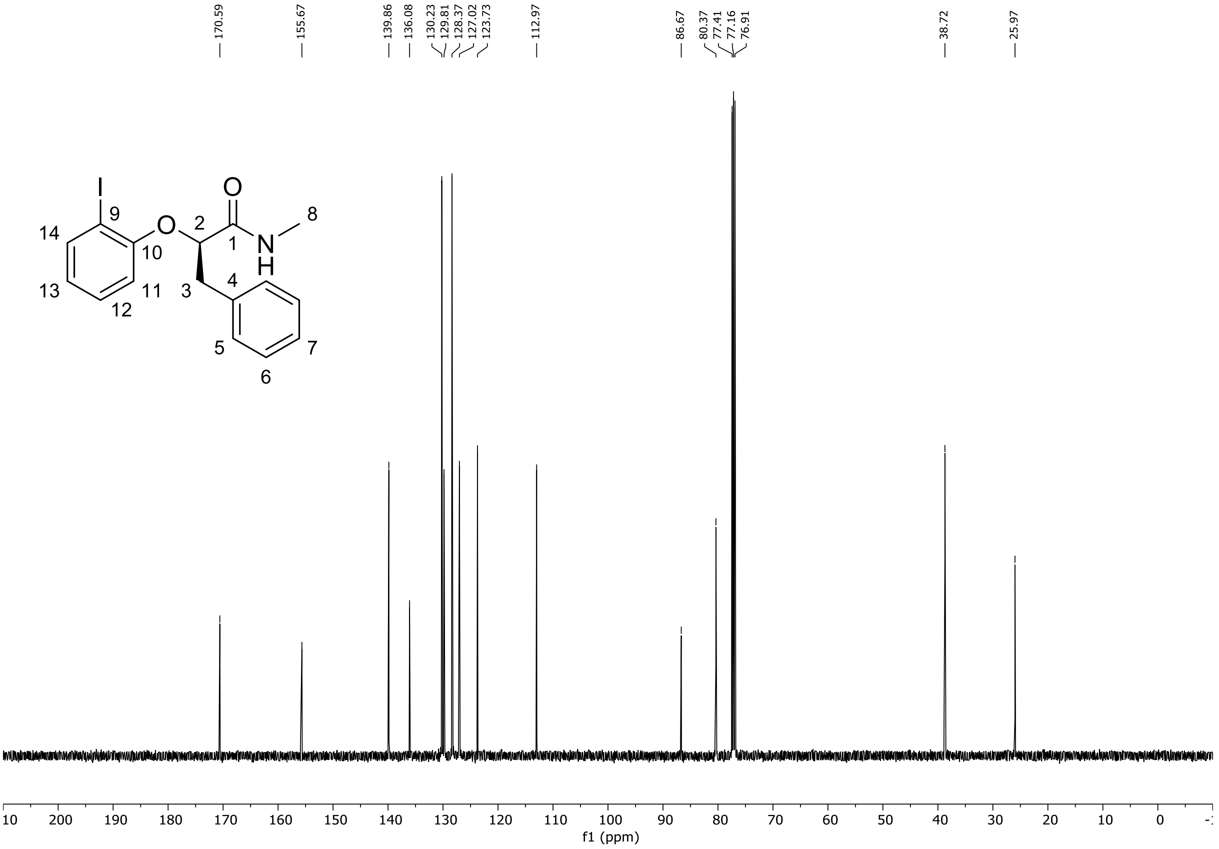

Dimethyl 2,2'-((2-iodo-1,3-phenylene)bis(oxy))(2*R*,2'*R*)-bis(3-phenylpropanoate) (S12)

^{1}H NMR (400 MHz, $CDCl_3$):

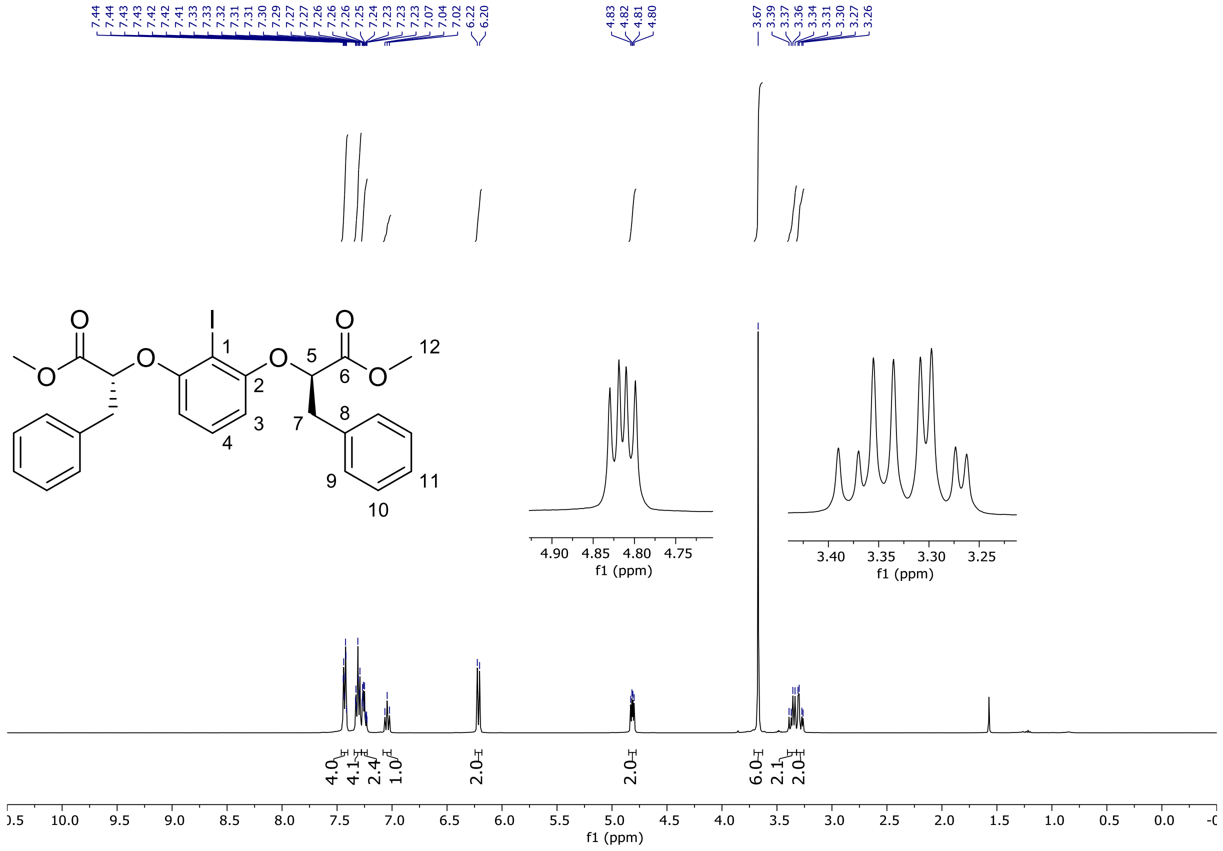

$^{13}C\{^1H\}$ NMR (101 MHz, $CDCl_3$):

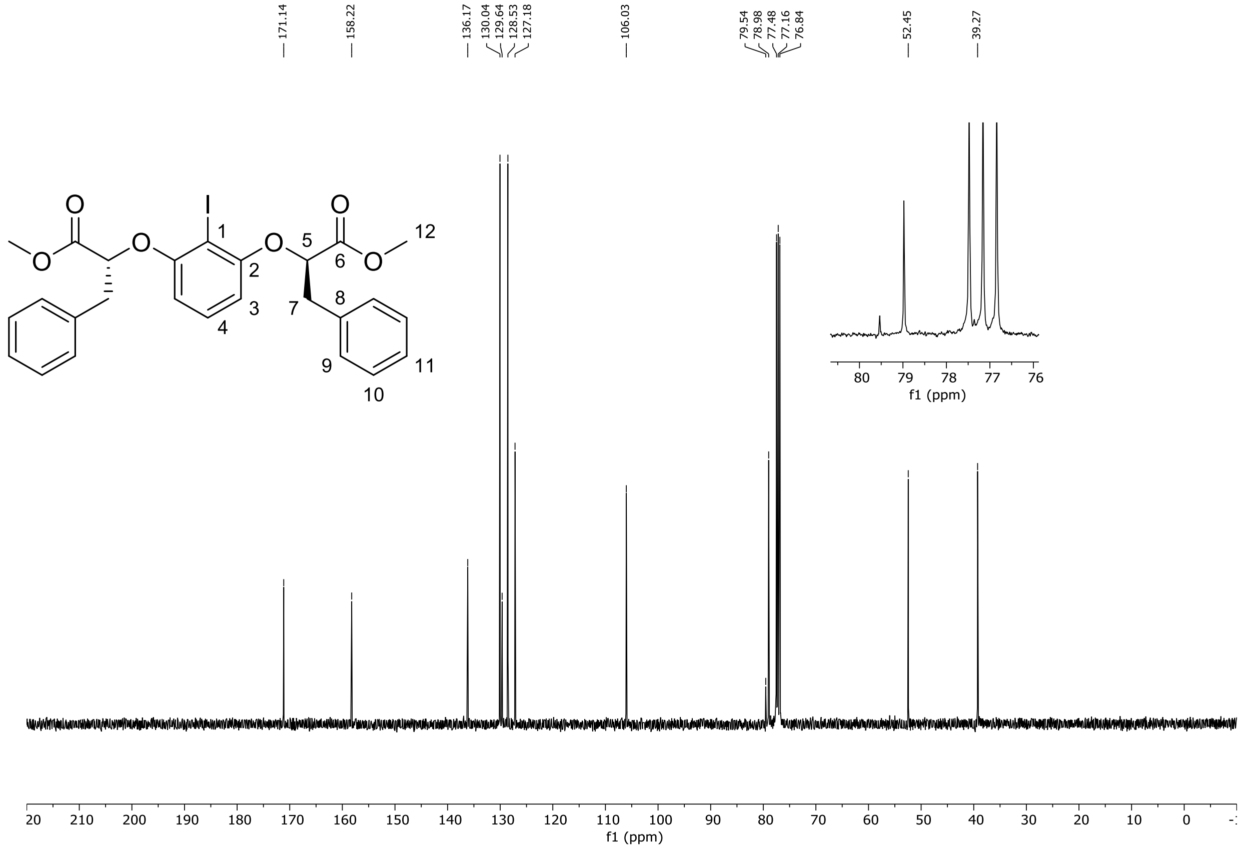

(2*R*,2'*R*)-2,2'-((2-Iodo-1,3-phenylene)bis(oxy))bis(3-phenylpropanoic acid) (**S13**)

^{1}H NMR (599 MHz, $CDCl_3$):

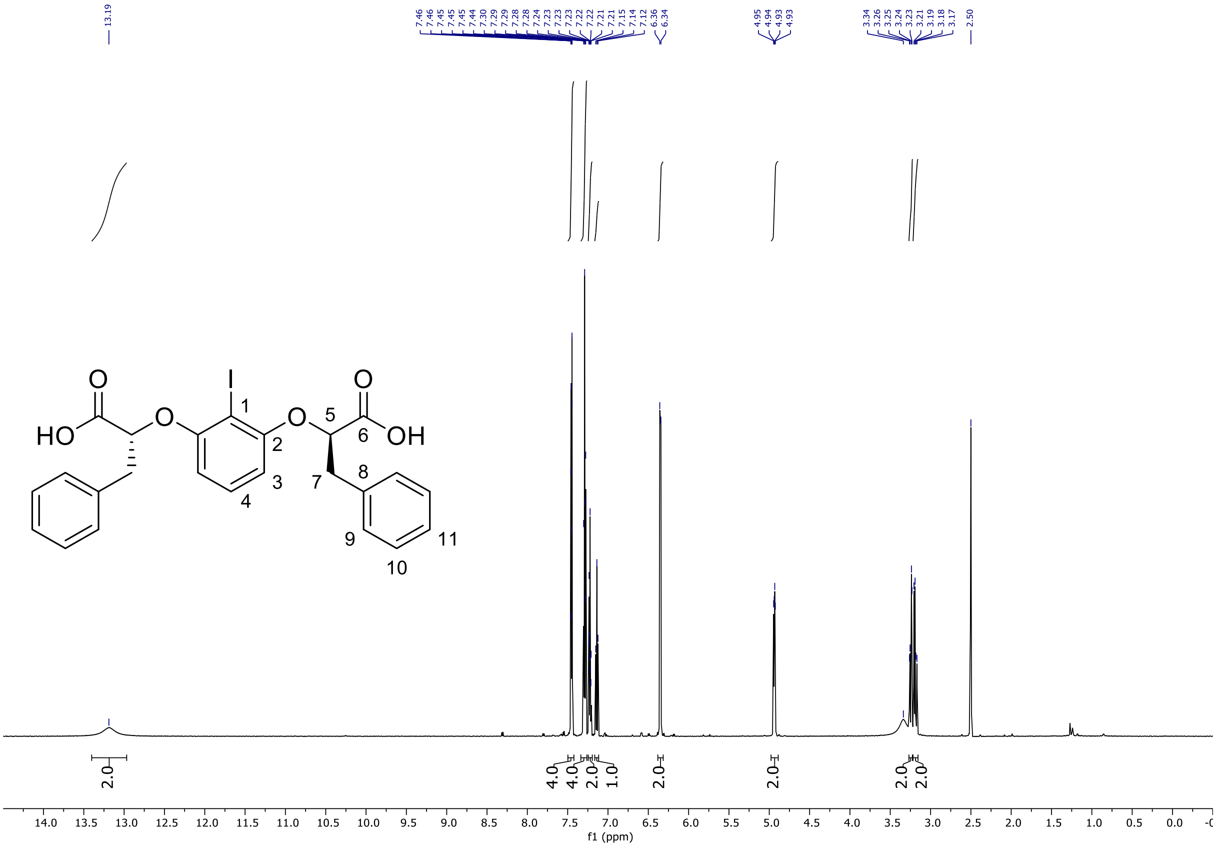

$^{13}C\{^{1}H\}$ NMR (151 MHz, $CDCl_3$):

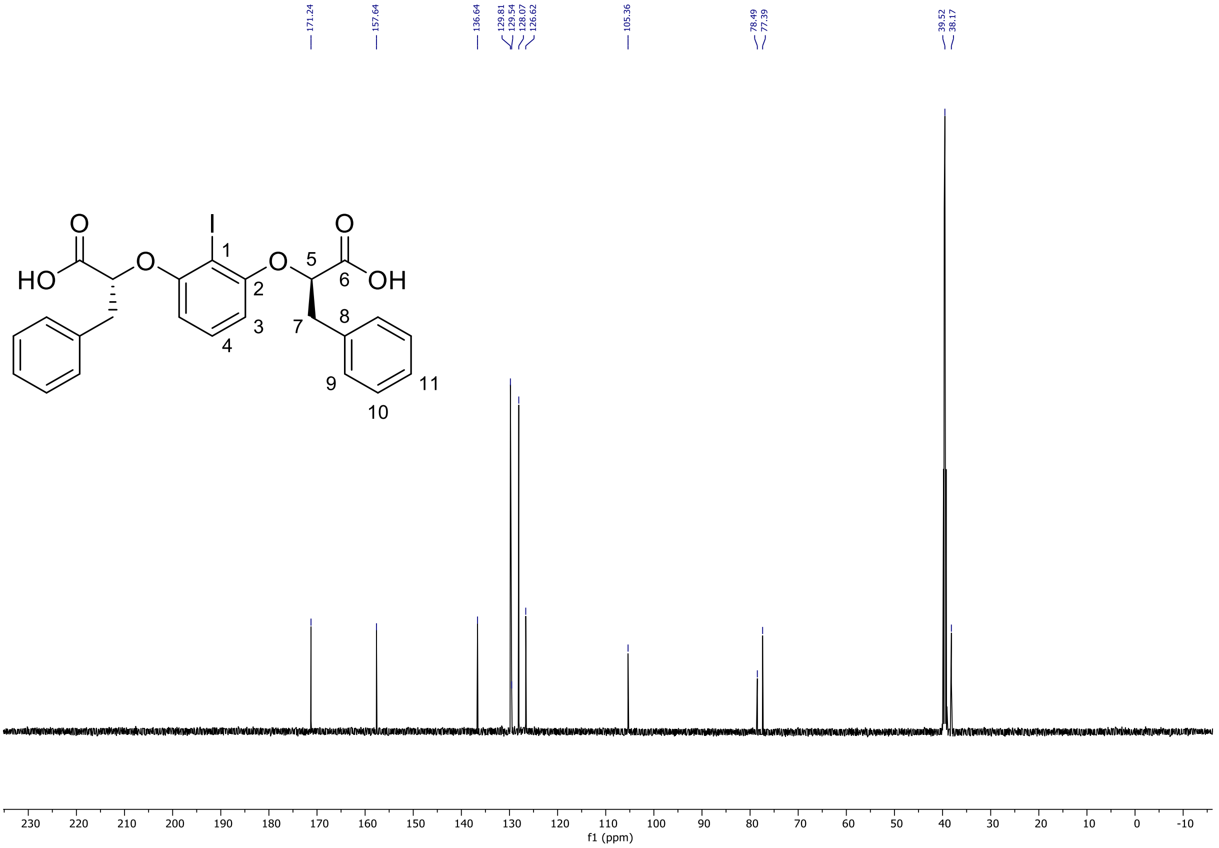

(2*R*,2'*R*)-2,2'-((2-Iodo-1,3-phenylene)bis(oxy))bis(*N*-methyl-3-phenylpropanamide) (**7**)

^{1}H NMR (500 MHz, $CDCl_3$):

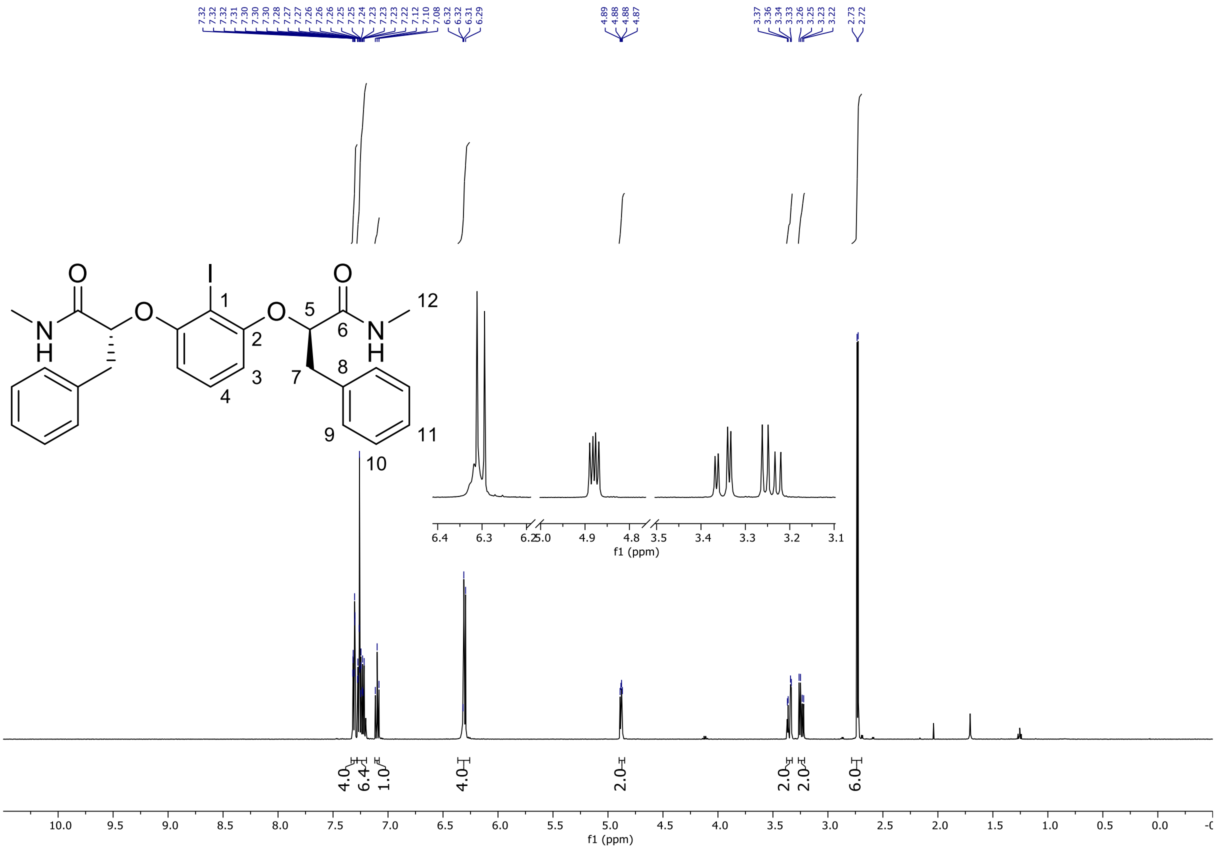

^{13}C{^{1}H} NMR (126 MHz, $CDCl_3$):

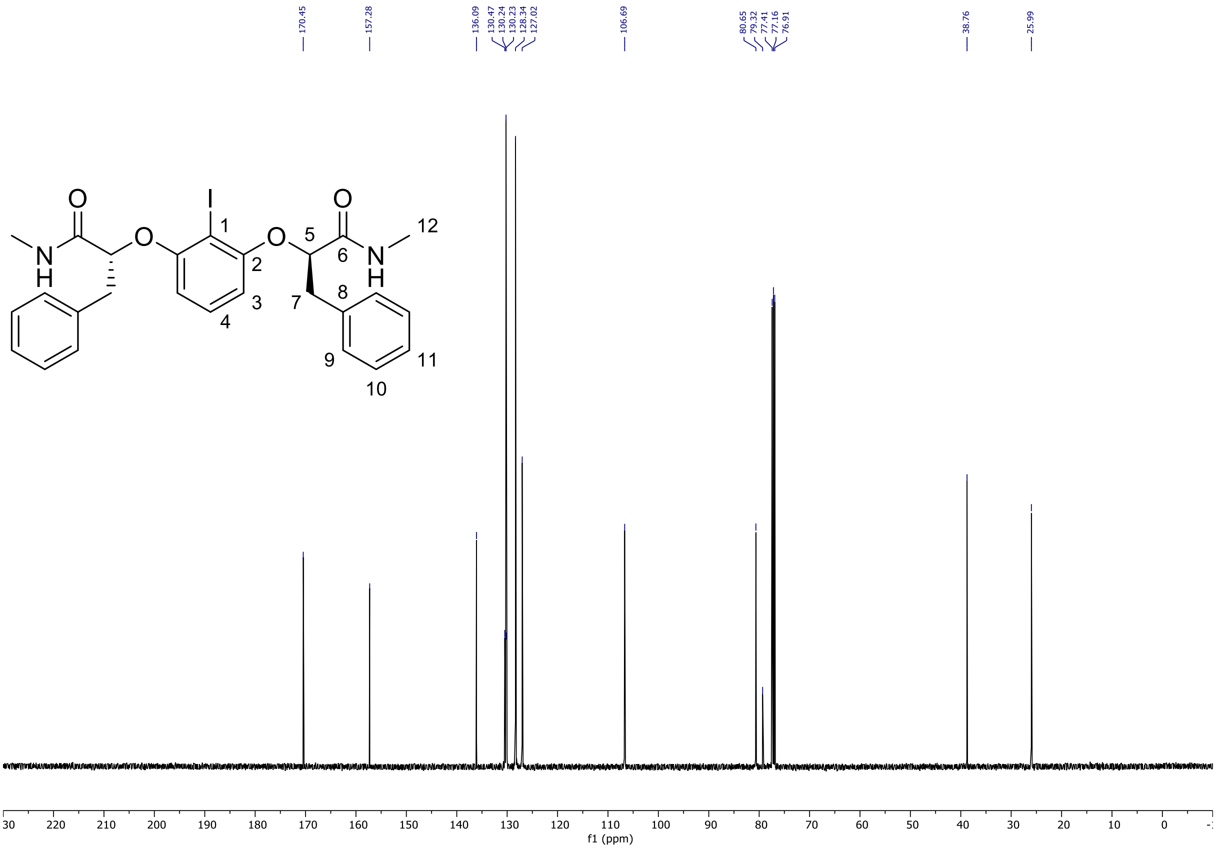

(2*R*,2'*R*)-2,2'-((2-Iodo-1,3-phenylene)bis(oxy))bis(*N*,*N*-dimethyl-3-phenylpropanamide) (**S14**)

^{1}H NMR (400 MHz, $CDCl_3$):

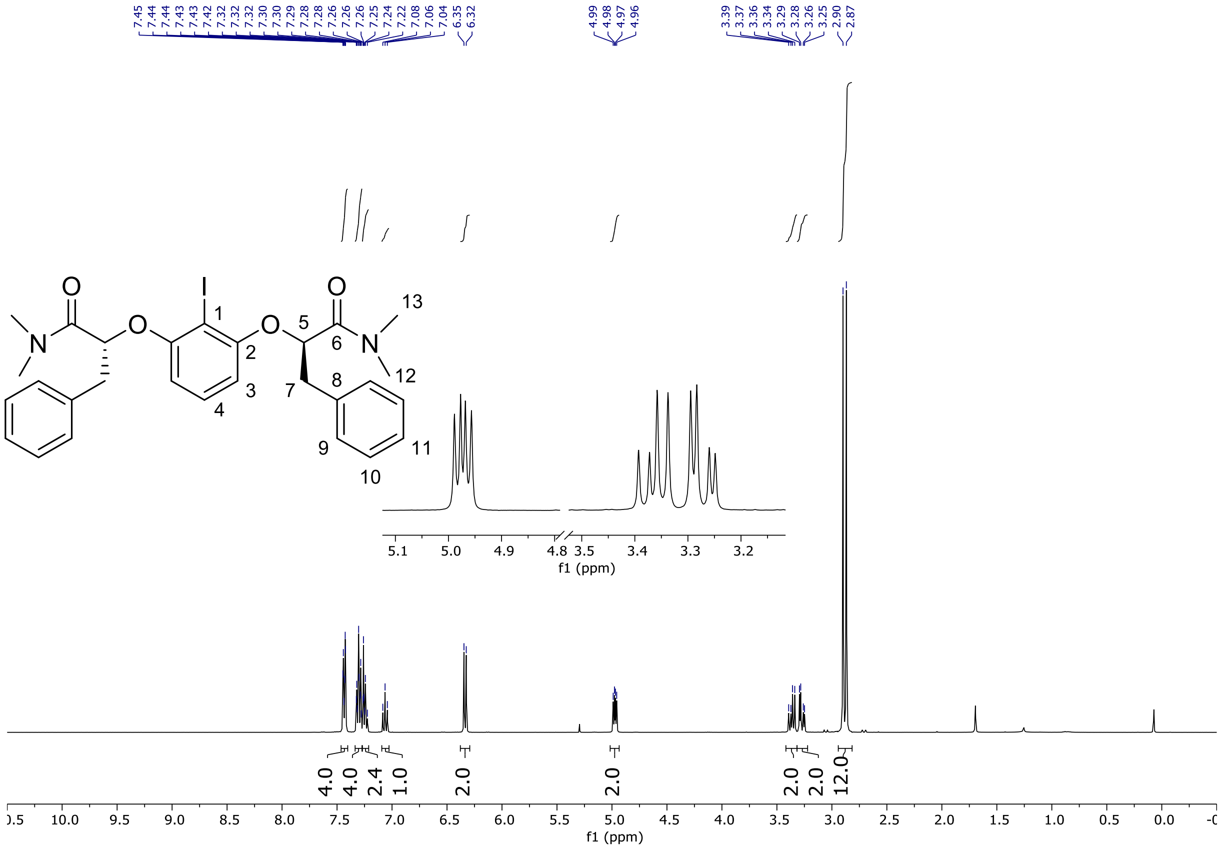

^{13}C{^{1}H} NMR (101 MHz, $CDCl_3$):

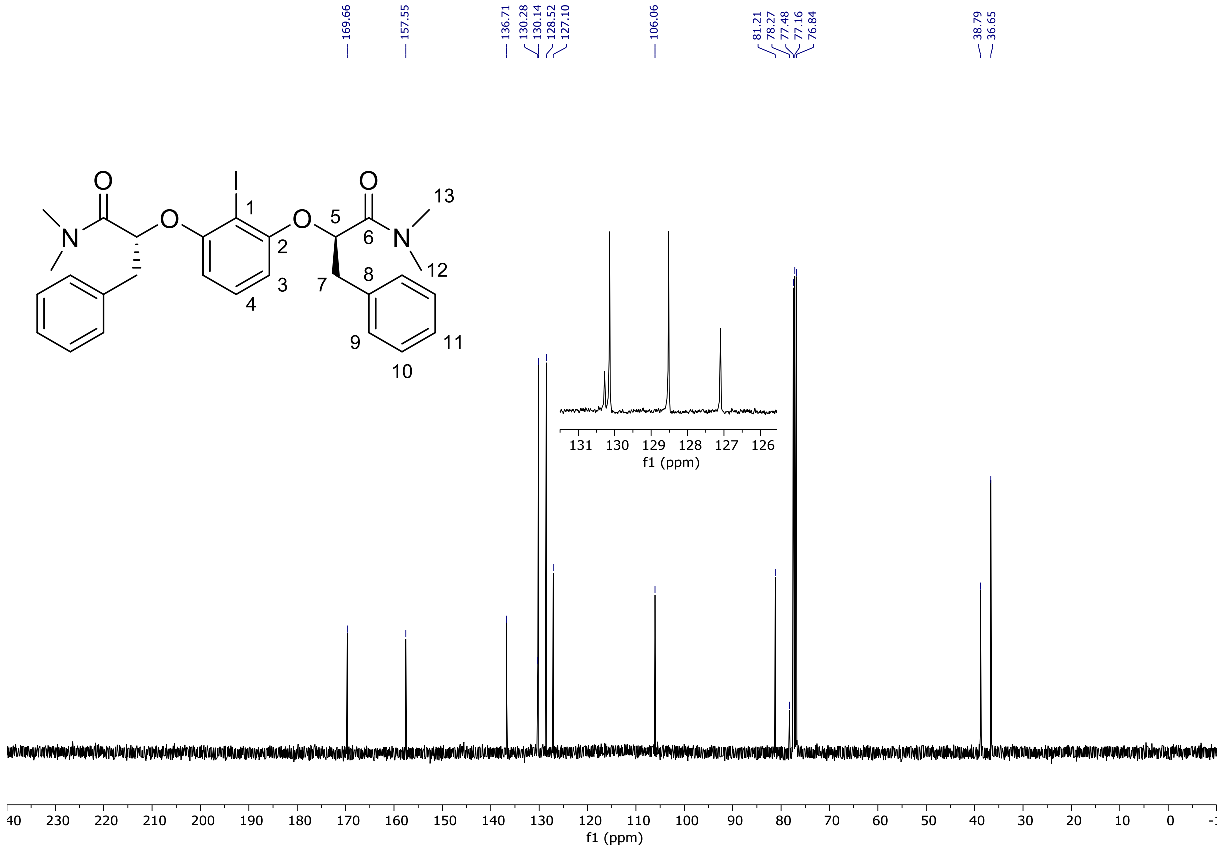

Methyl 4-iodo-3,5-bis(((*R*)-1-(methylamino)-1-oxo-3-phenylpropan-2-yl)oxy)benzoate (S15)

^{1}H NMR (599 MHz, $CDCl_3$):

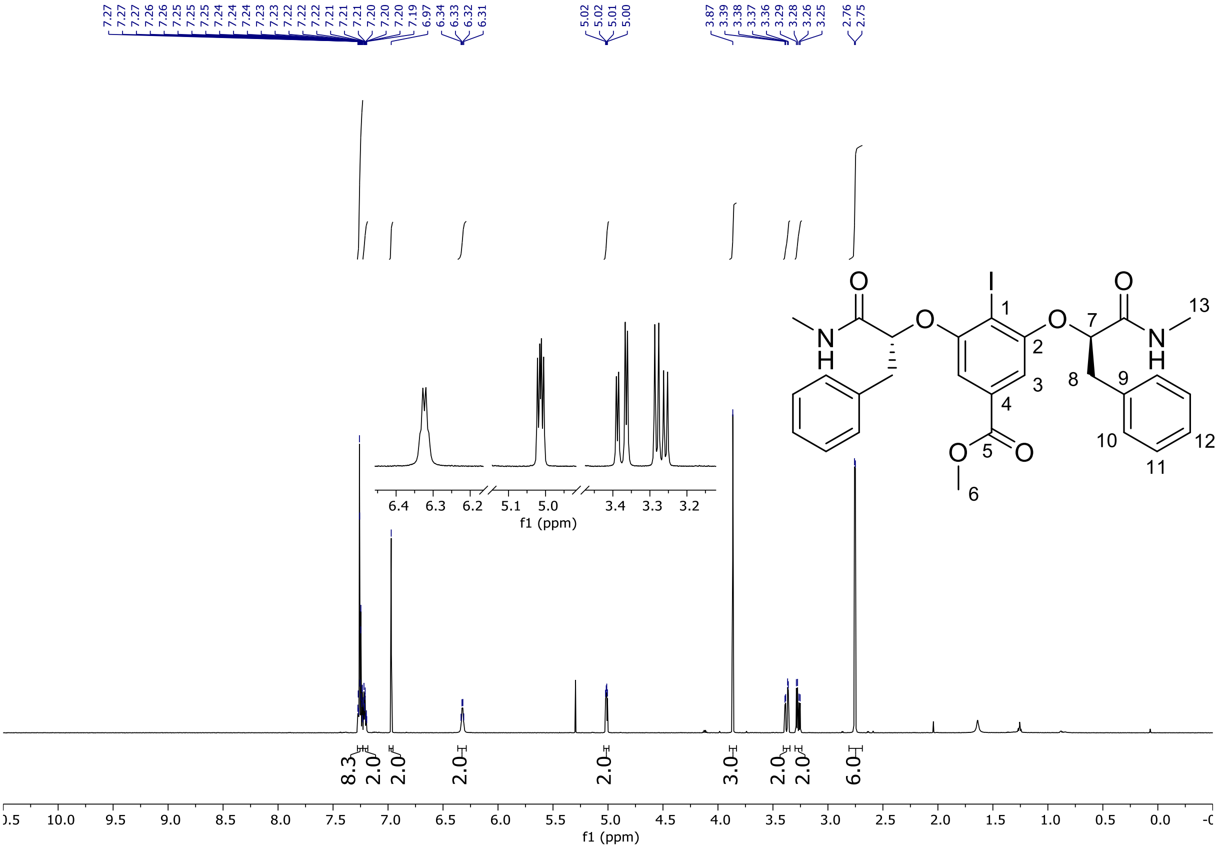

^{13}C{^{1}H} NMR (151 MHz, $CDCl_3$):

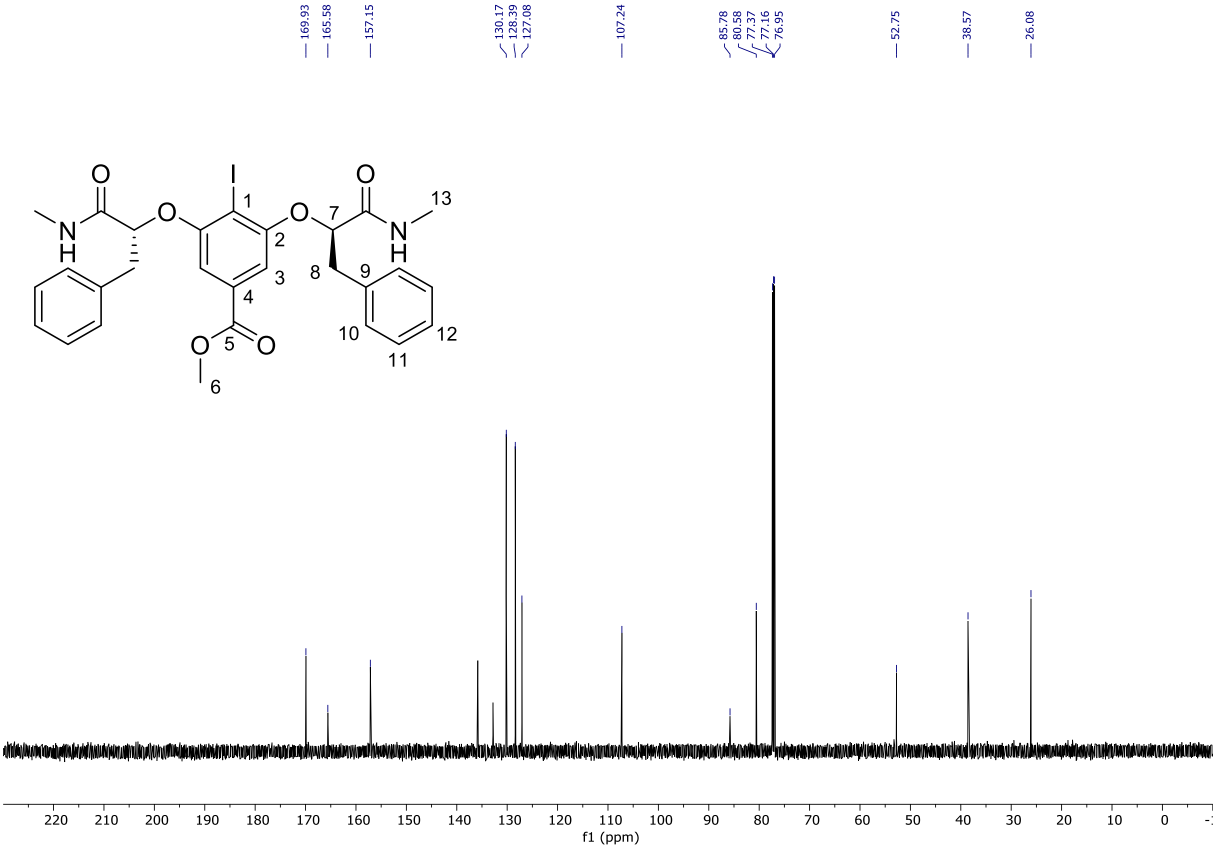

(2*R*,2'*R*)-2,2'-((2-Iodo-5-methyl-1,3-phenylene)bis(oxy))bis(*N*-methyl-3-phenyl-propanamide) (S16)

^{1}H NMR (599 MHz, CDCl$_3$):

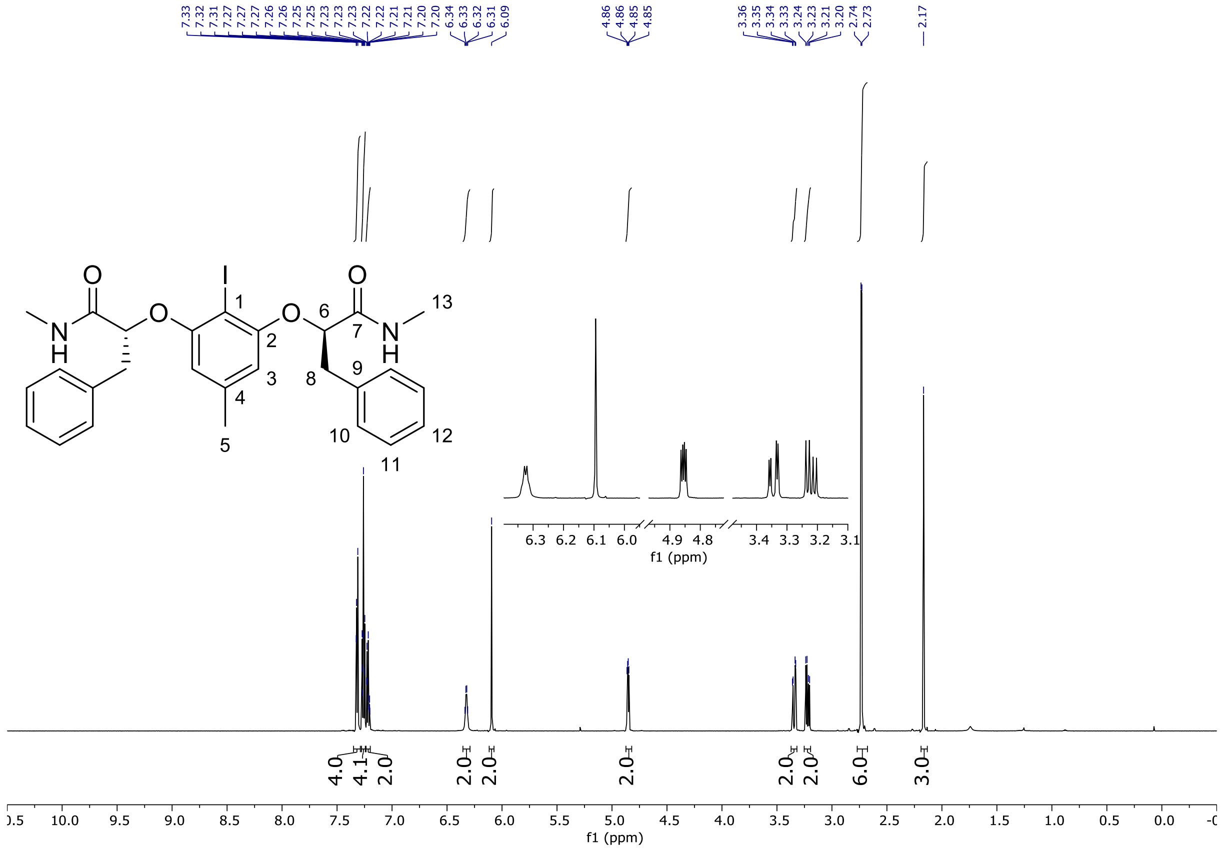

^{13}C{^{1}H} NMR (151 MHz, CDCl$_3$):

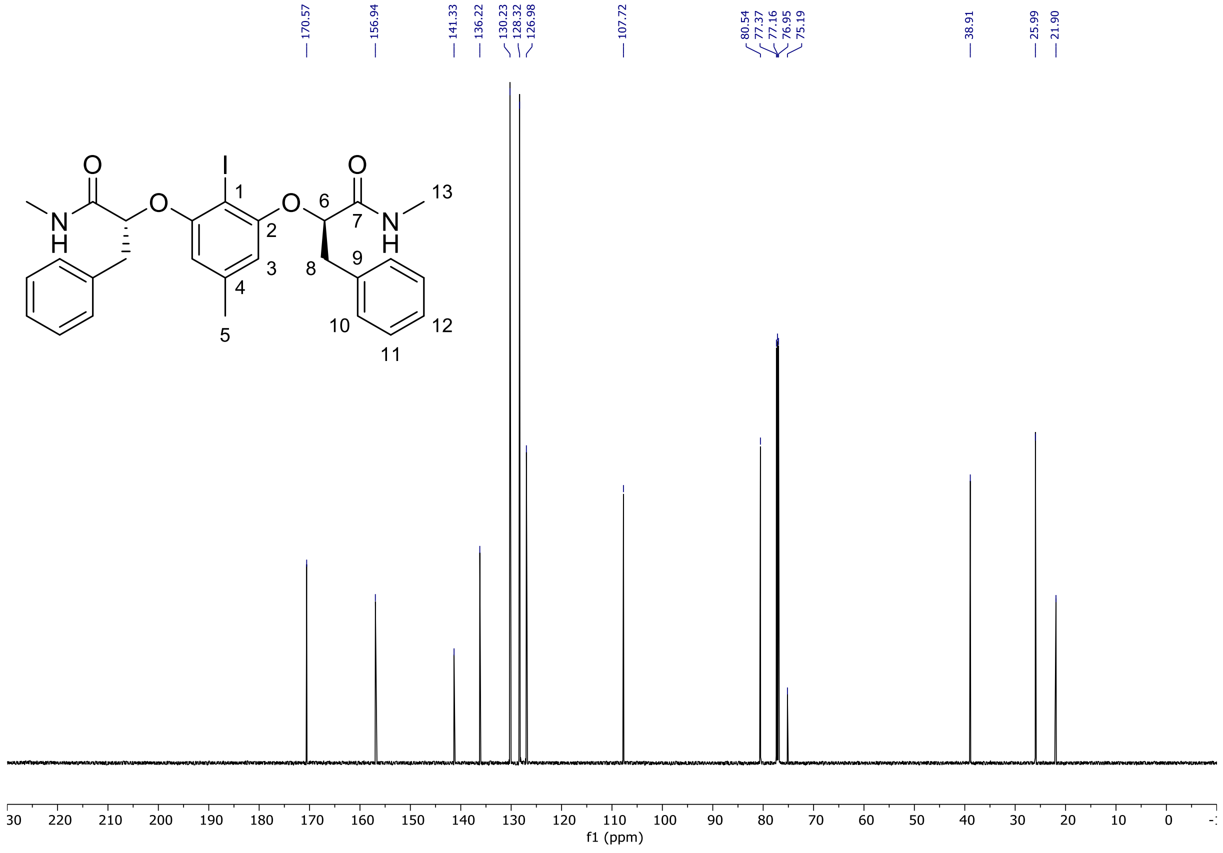

1-(Allyloxy)-3,5-difluorobenzene (**S22**)

^{1}H NMR (400 MHz, $CDCl_3$):

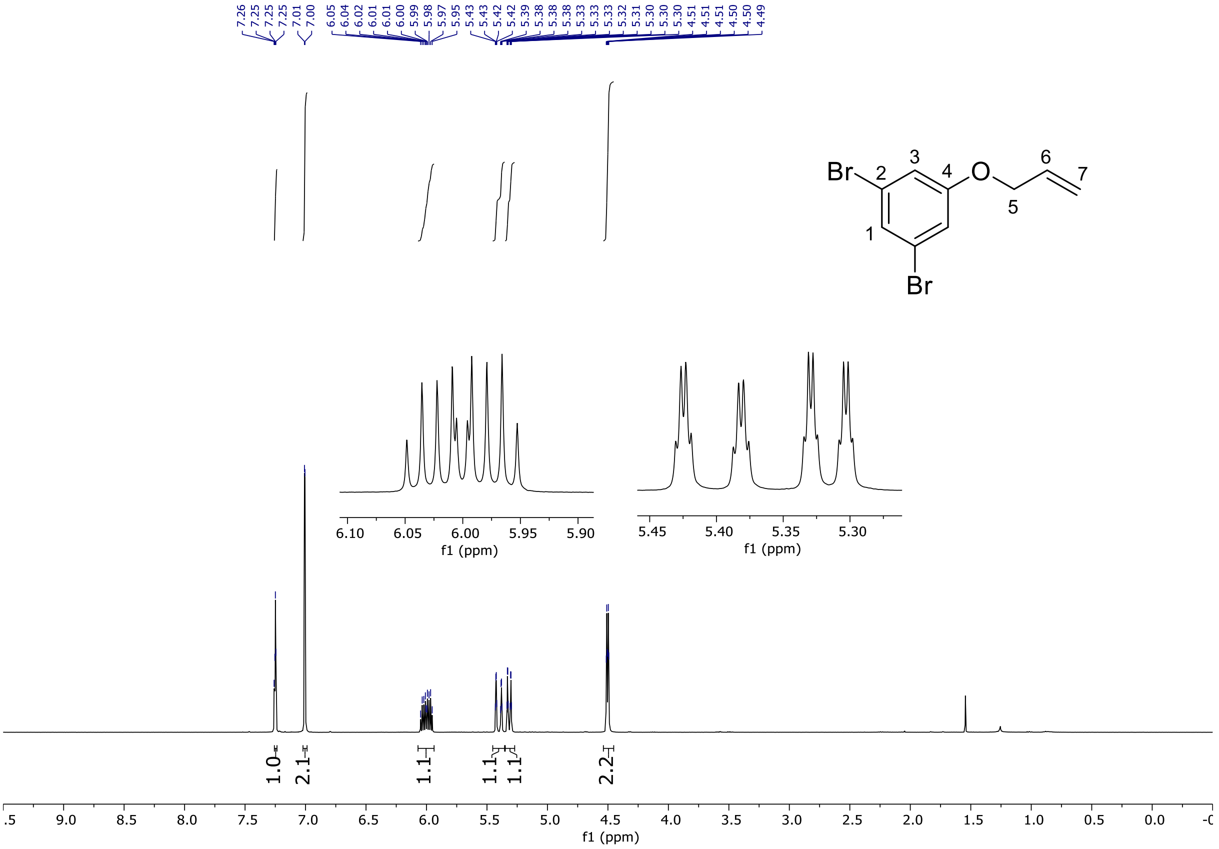

^{13}C{^{1}H} NMR (101 MHz, $CDCl_3$):

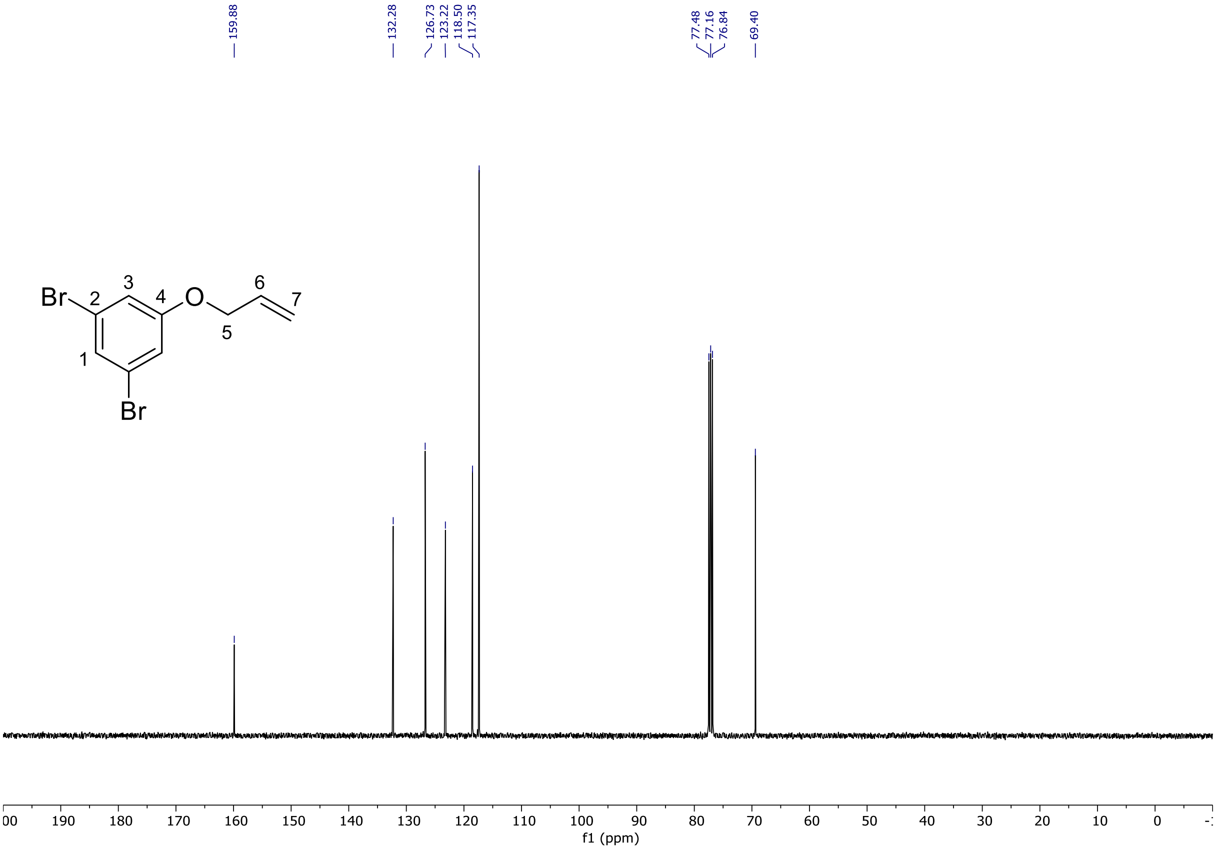

1-(Allyloxy)-3,5-dichlorobenzene (**S23**)

^{1}H NMR (400 MHz, $CDCl_3$):

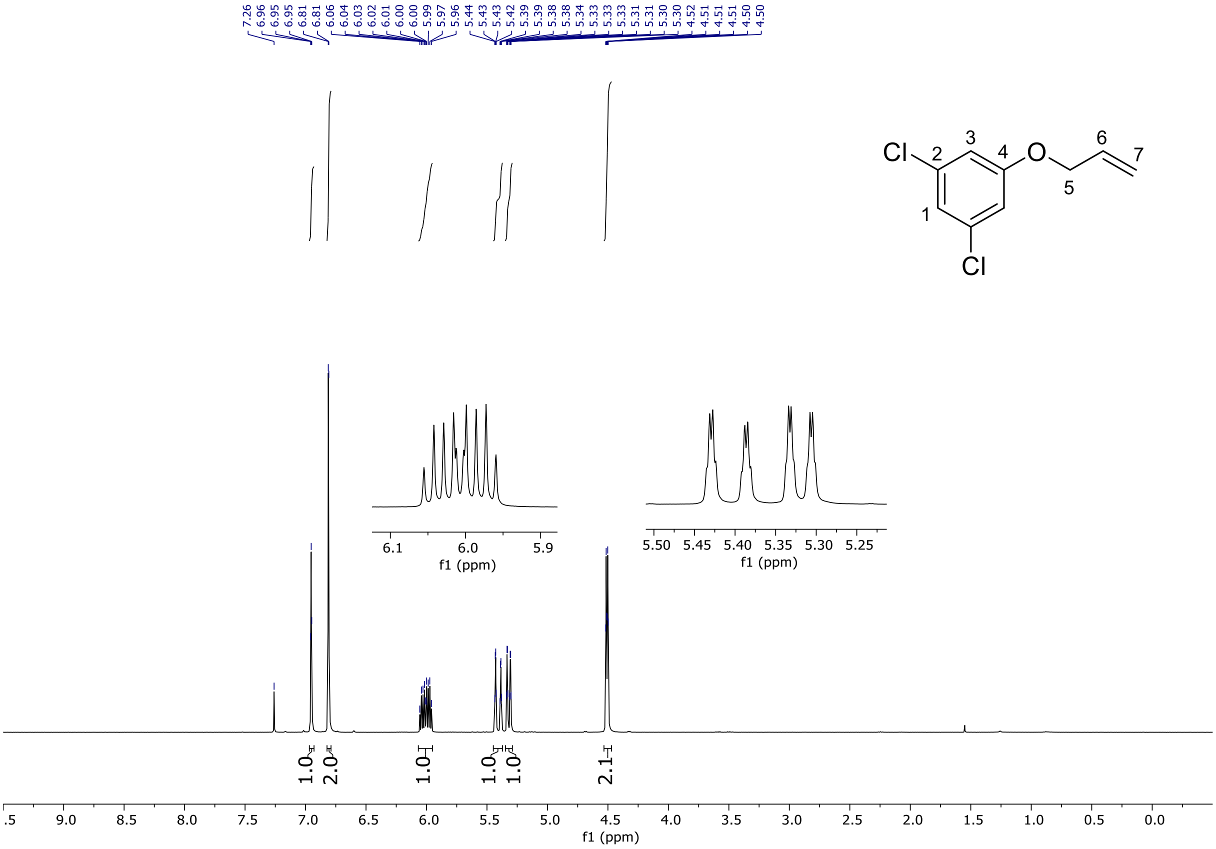

^{13}C{^{1}H} NMR (151 MHz, $CDCl_3$):

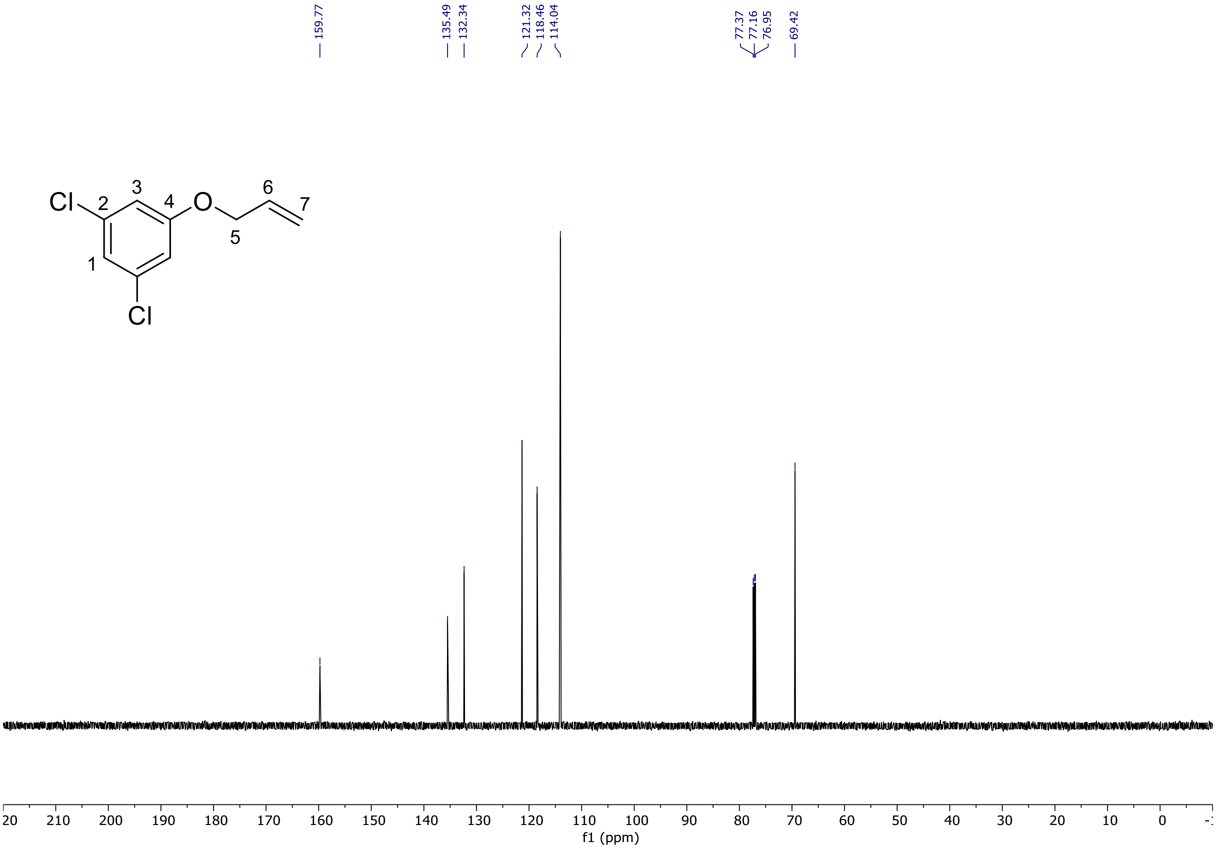

2-(Allyloxy)-1,4-dichlorobenzene (S24)

^{1}H NMR (400 MHz, $CDCl_3$):

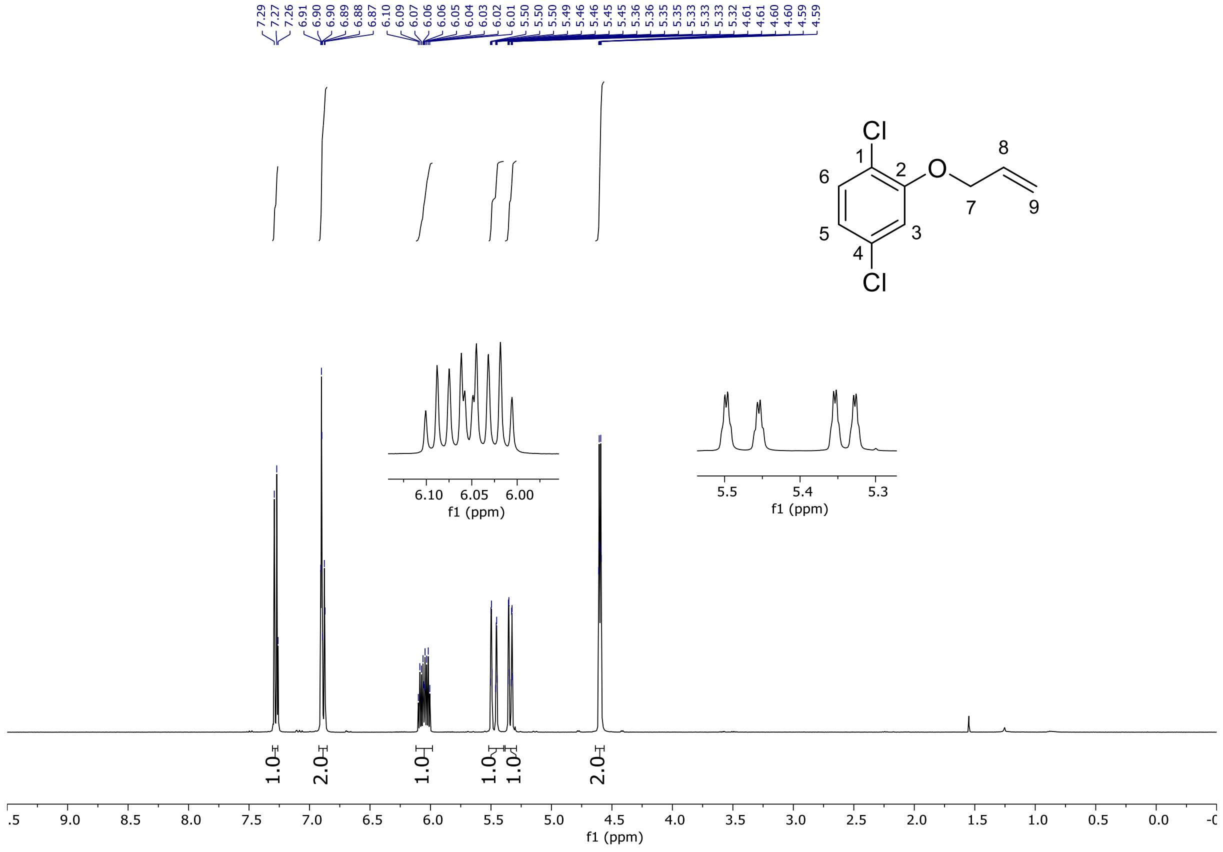

^{13}C{^{1}H} NMR (151 MHz, $CDCl_3$):

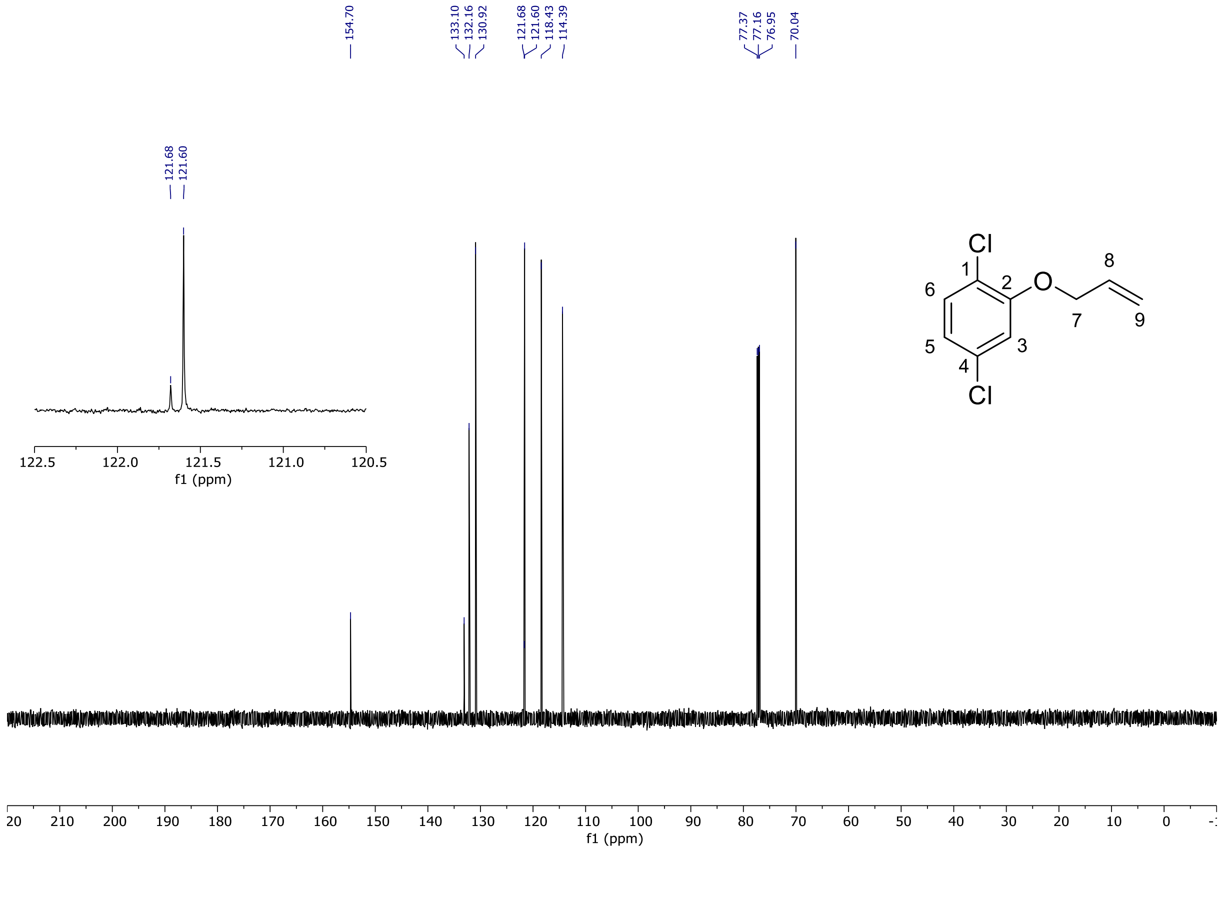

4-(Allyloxy)phenyl methanesulfonate (S33)

^{1}H NMR (599 MHz, $CDCl_3$):

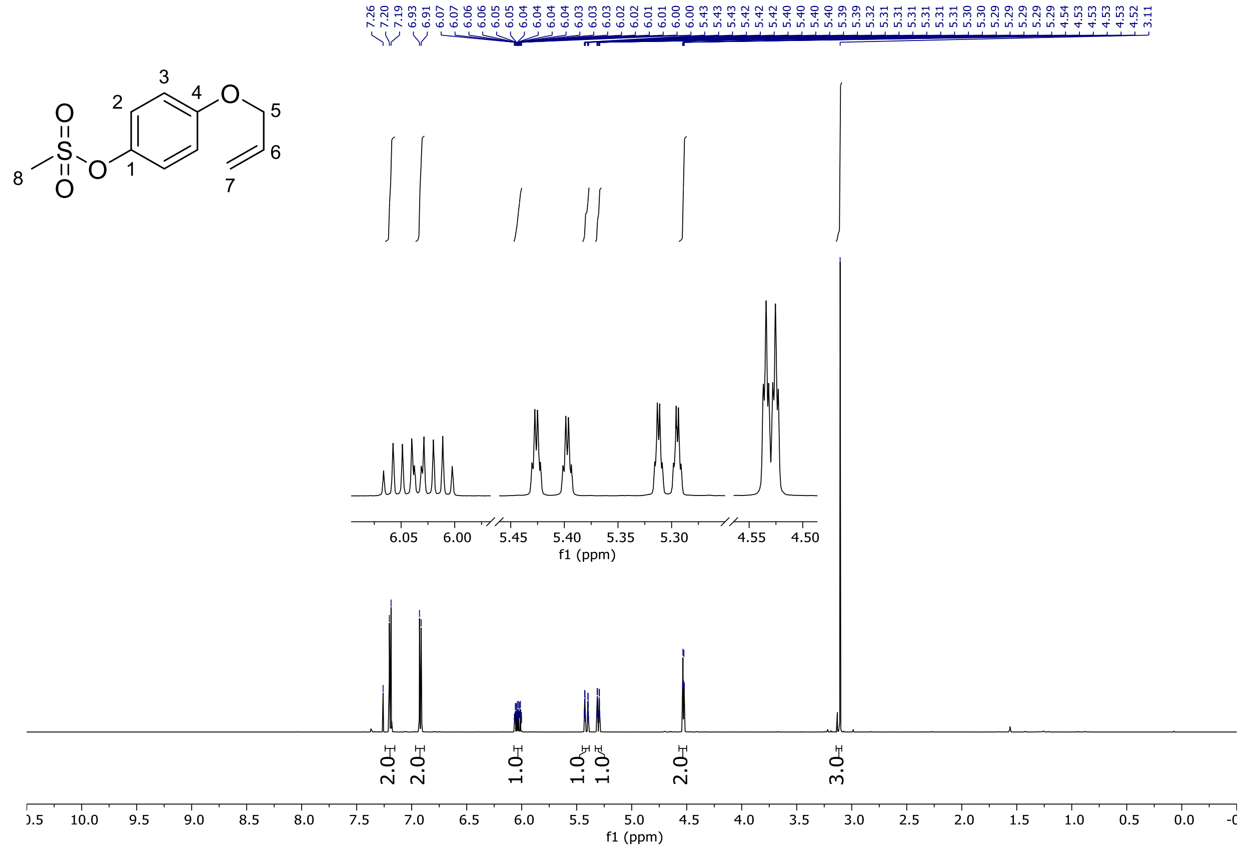

^{13}C{^{1}H} NMR (151 MHz, $CDCl_3$):

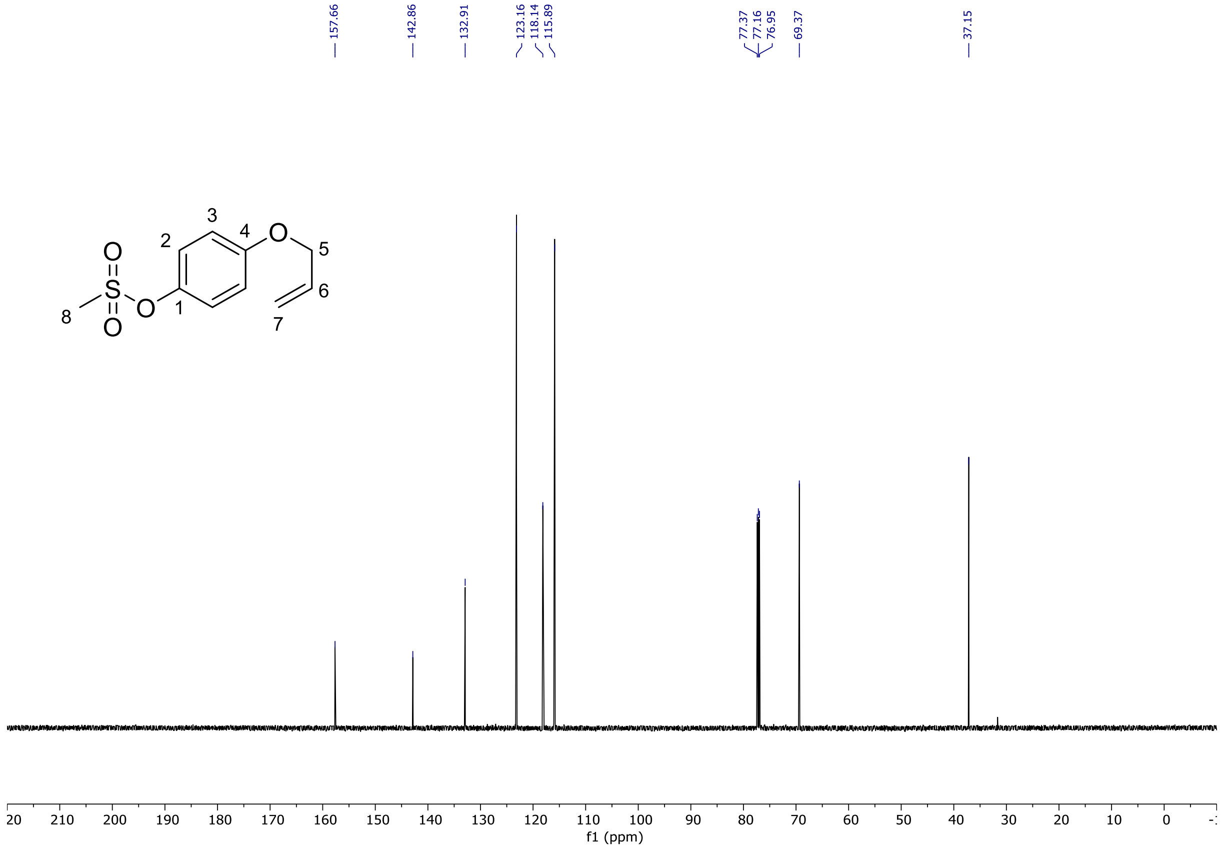

N-Allyl-N-(4-bromophenyl)-4-methylbenzenesulfonamide (24)

^{1}H NMR (500 MHz, $CDCl_3$):

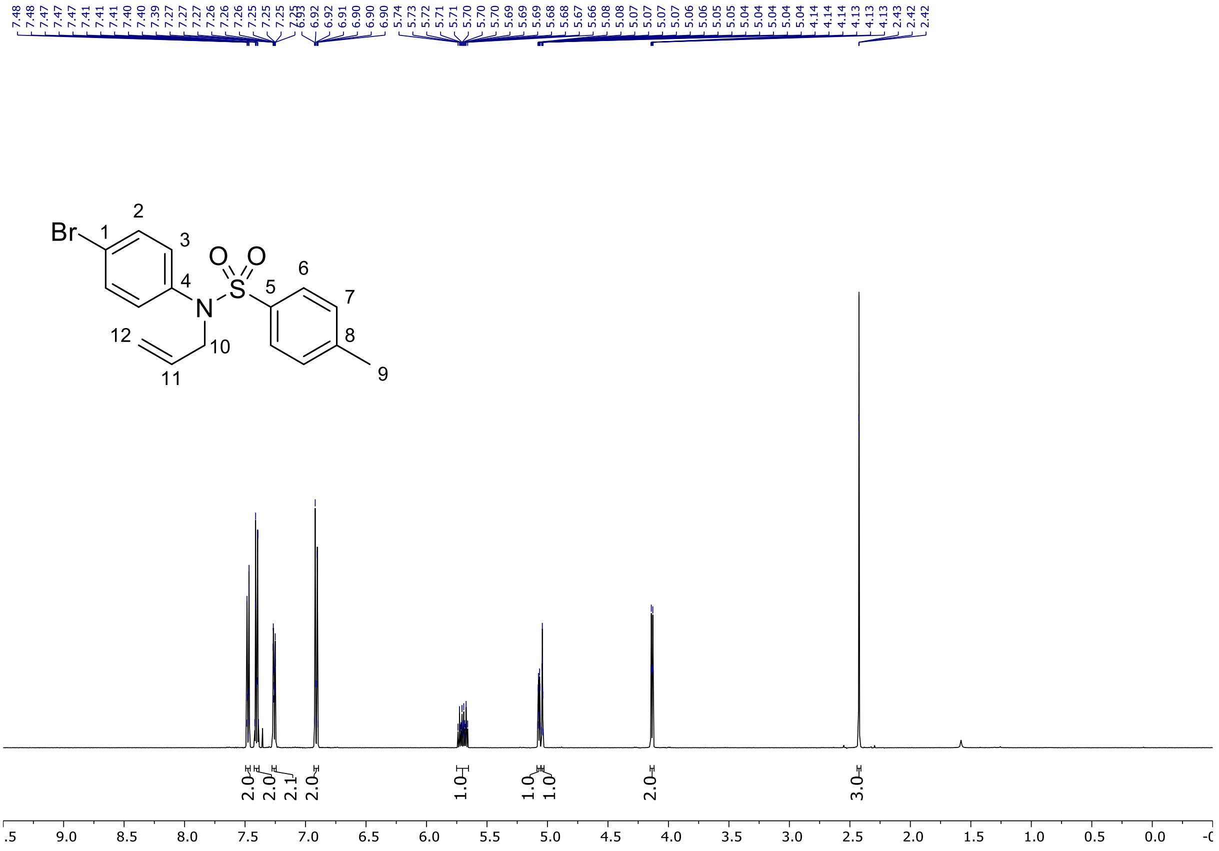

^{13}C{^{1}H} NMR (126 MHz, $CDCl_3$):

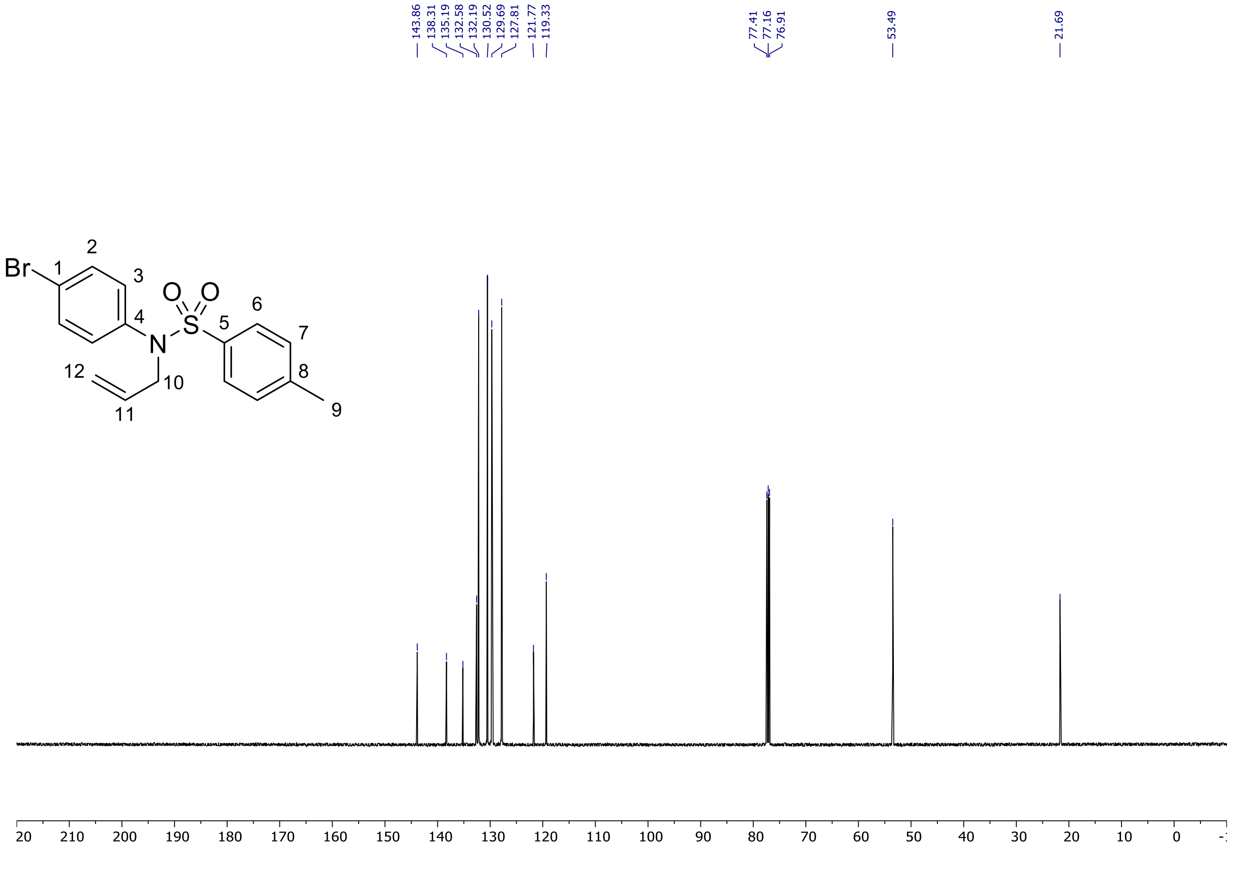

1-Bromo-4-((prop-2-yn-1-yl-3-d)oxy)benzene (S37)

^{1}H NMR (400 MHz, $CDCl_3$):

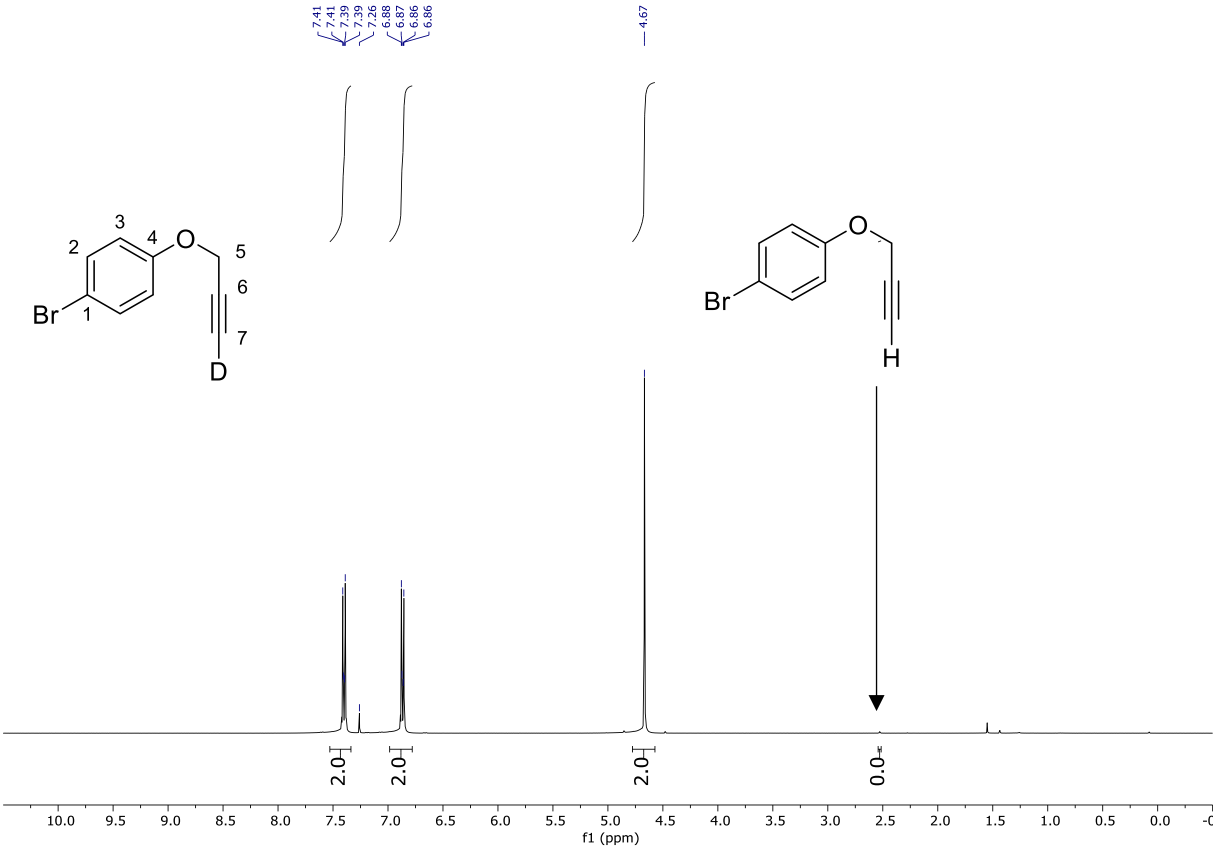

^{13}C{^{1}H} NMR (101 MHz, $CDCl_3$):

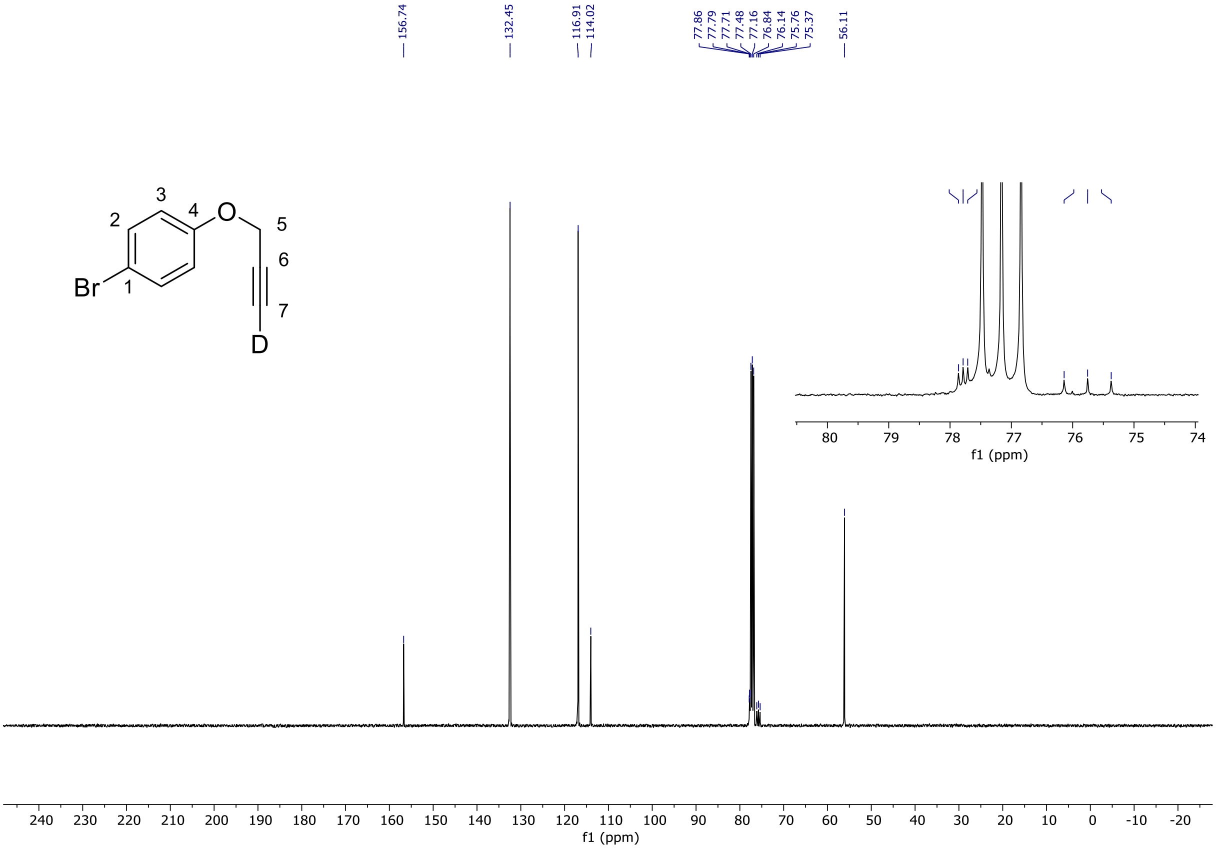

(*Z*)-1-((Allyl-3-d)oxy)-4-bromobenzene (29)

^{1}H NMR (300 MHz, $CDCl_3$):

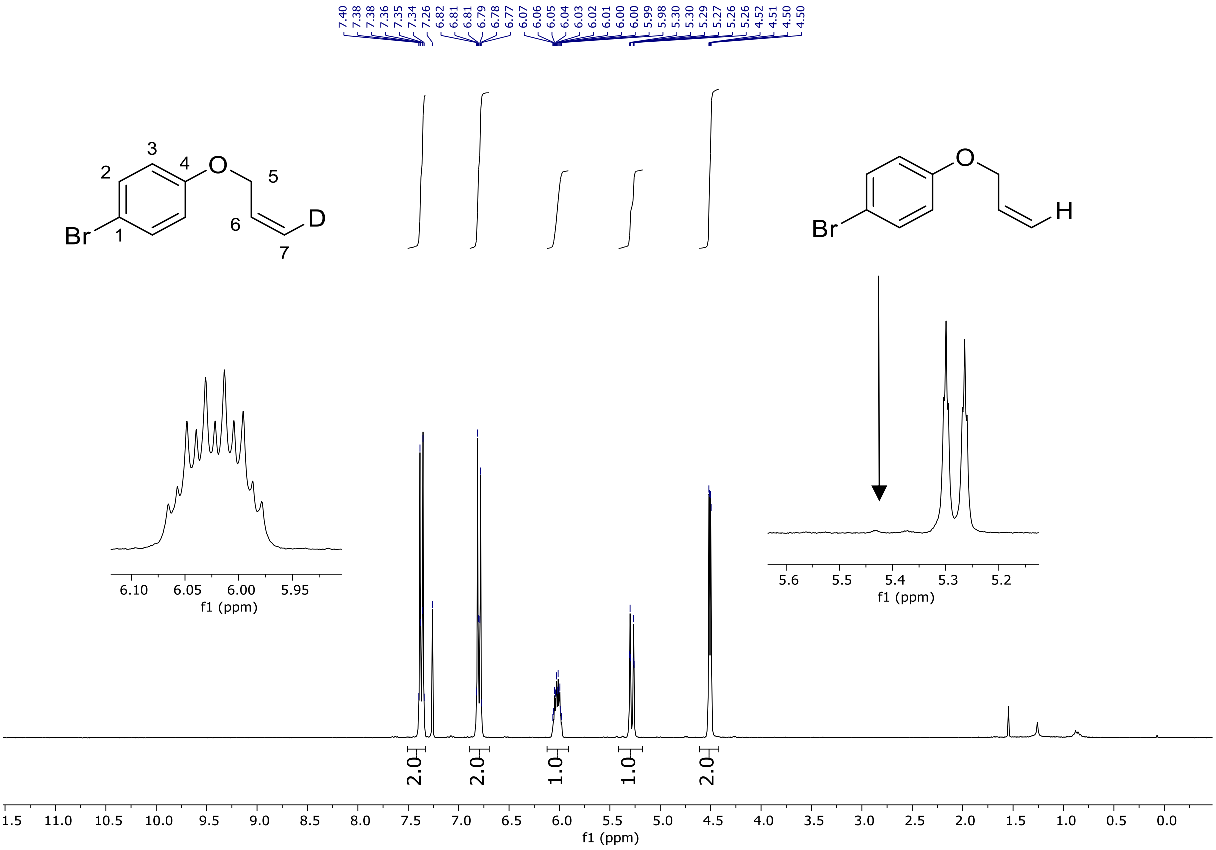

^{13}C{^{1}H} NMR (101 MHz, $CDCl_3$):

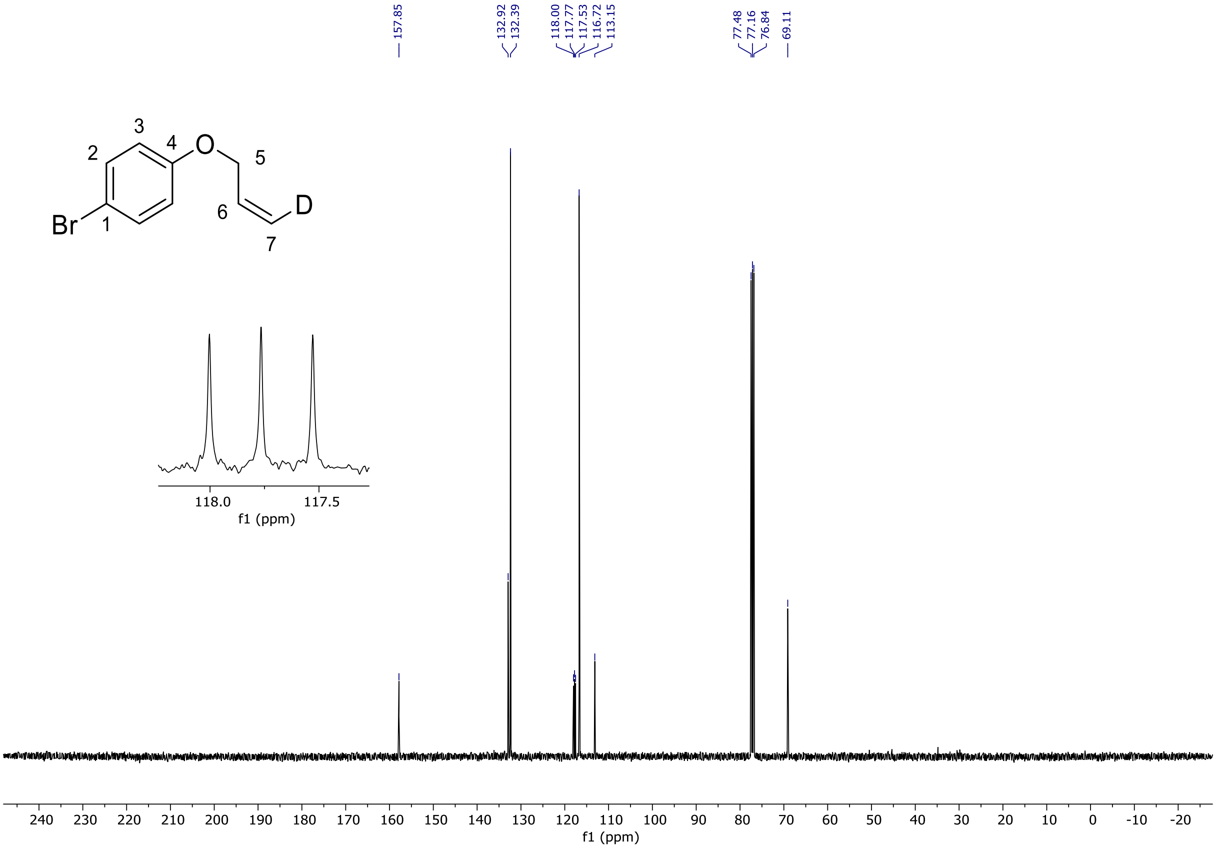

(*E*)-1-((Allyl-3-d)oxy)-4-bromobenzene (30)

^{1}H NMR (500 MHz, $CDCl_3$):

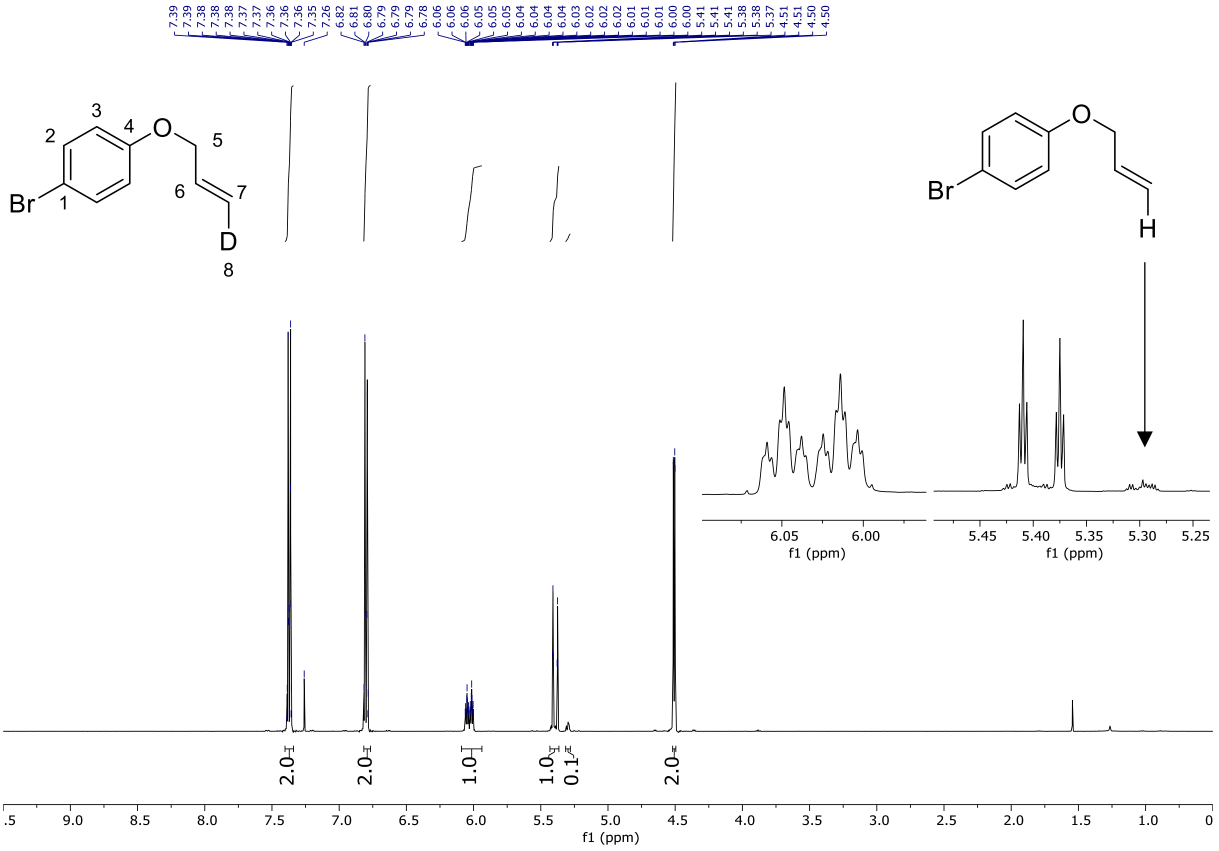

^{13}C{^{1}H} NMR (126 MHz, $CDCl_3$):

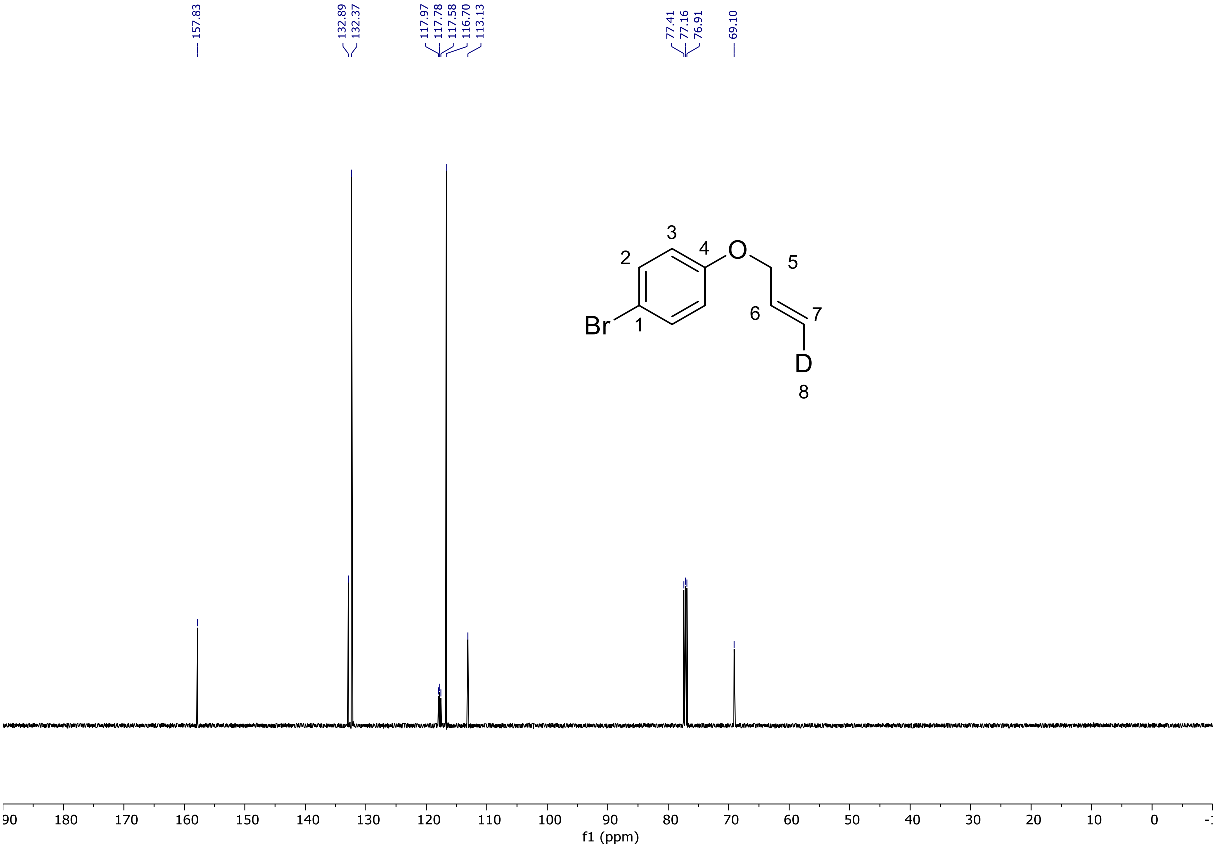

b. NMR Spectra of 3-Fluorochromanes

6-Bromo-3-fluorochromane (6)

^{1}H NMR (599 MHz, $CDCl_3$):

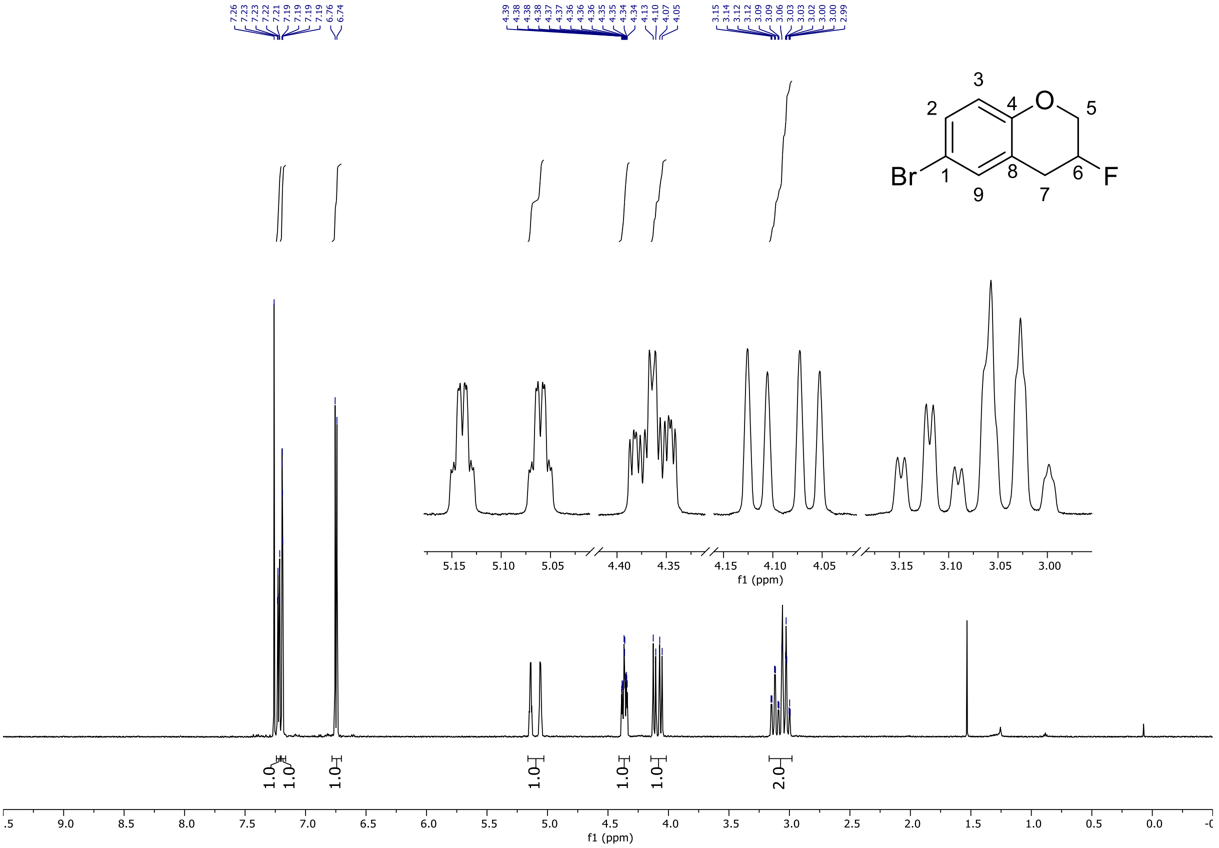

^{13}C{^{1}H} NMR (151 MHz, $CDCl_3$):

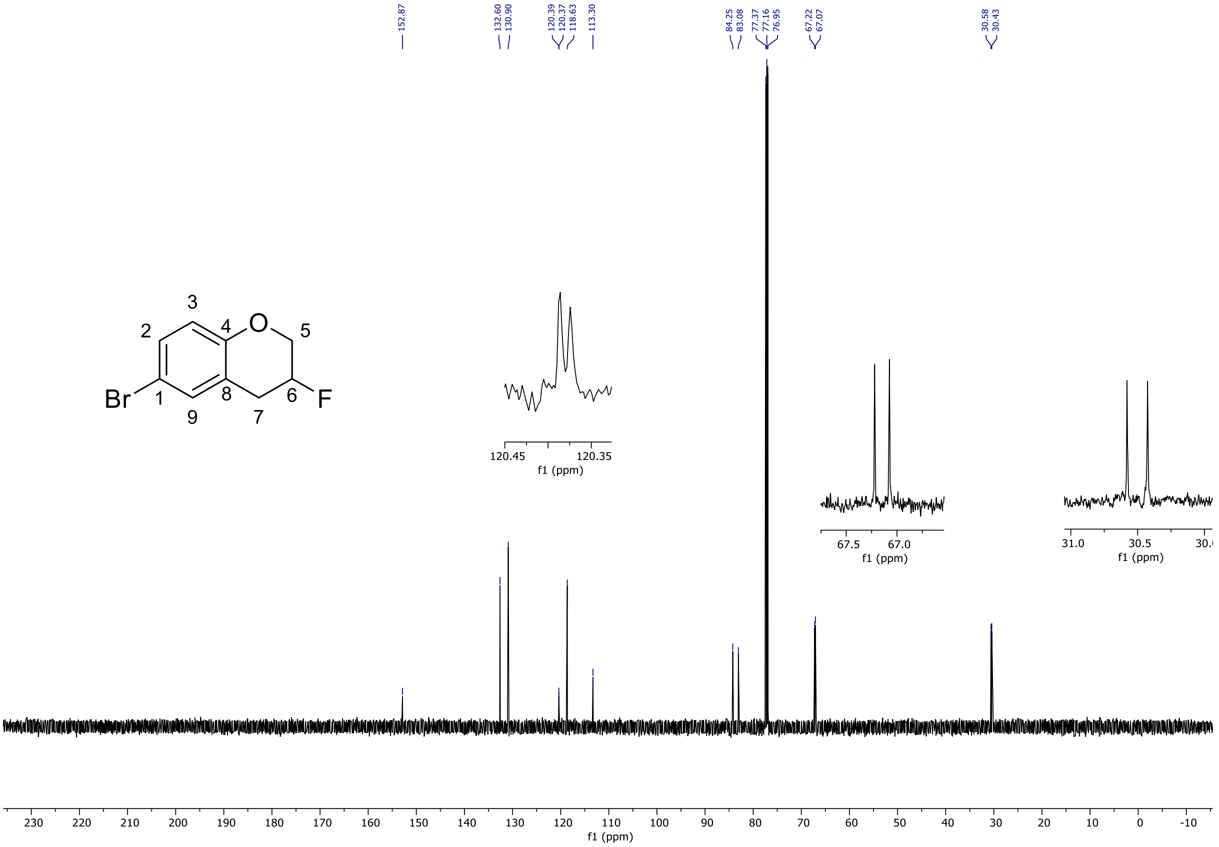

$^{19}F\{^1H\}$ NMR (564 MHz, $CDCl_3$):

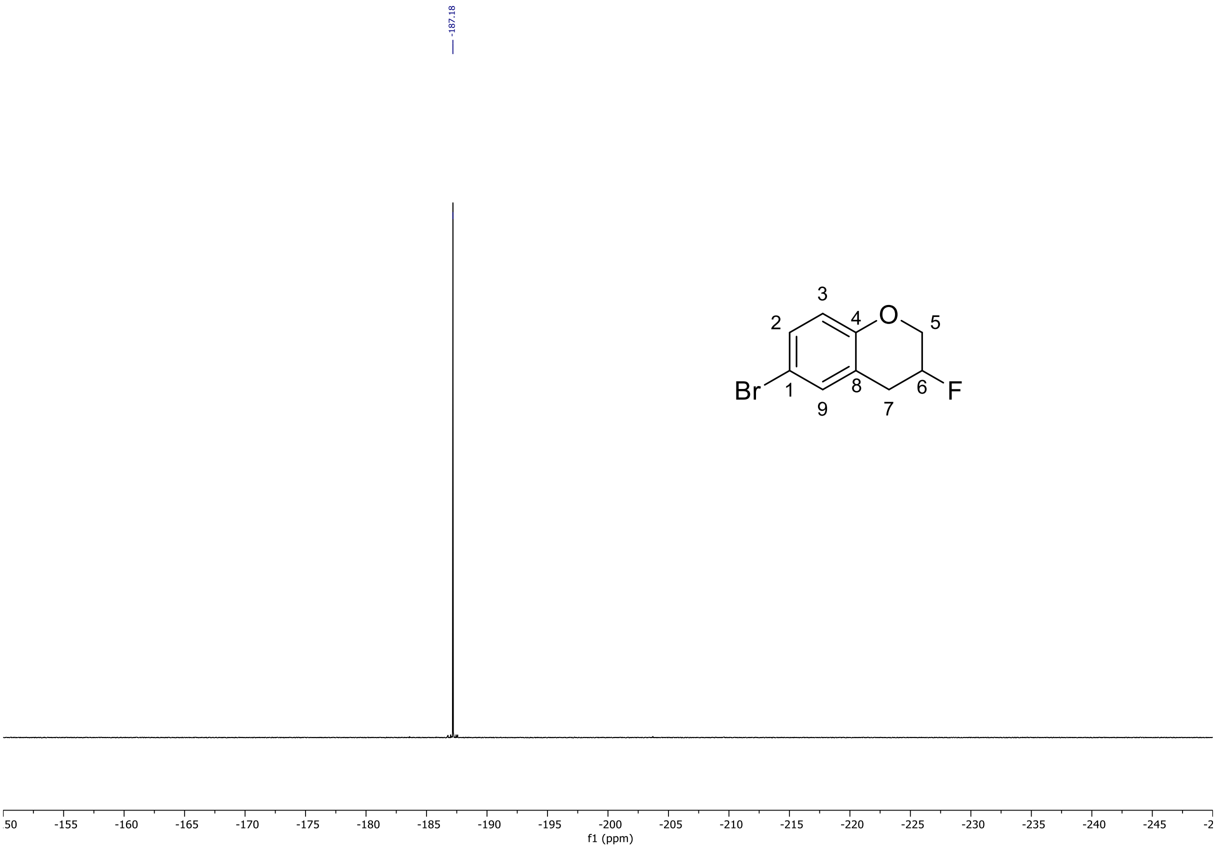

^{19}F NMR (564 MHz, $CDCl_3$):

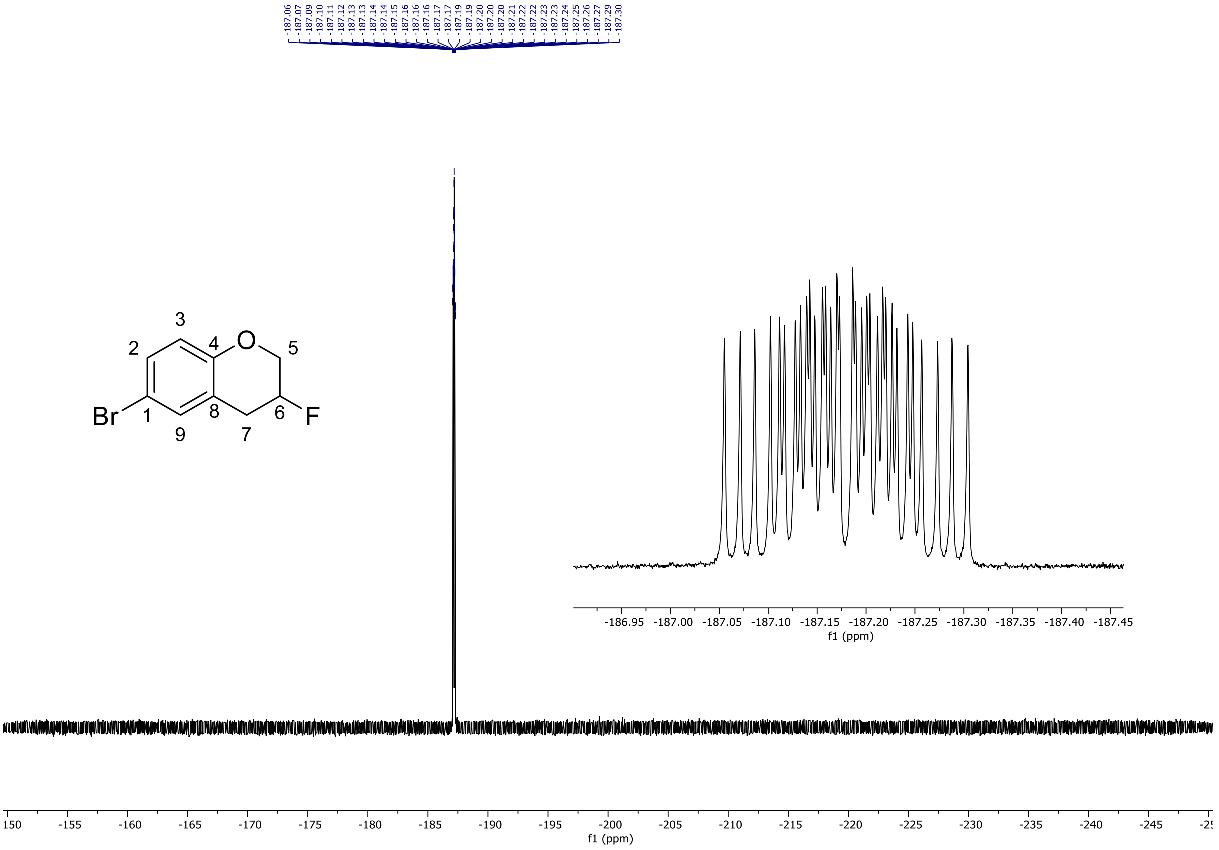

6-Chloro-3-fluorochromane (8)

^{1}H NMR (599 MHz, $CDCl_3$):

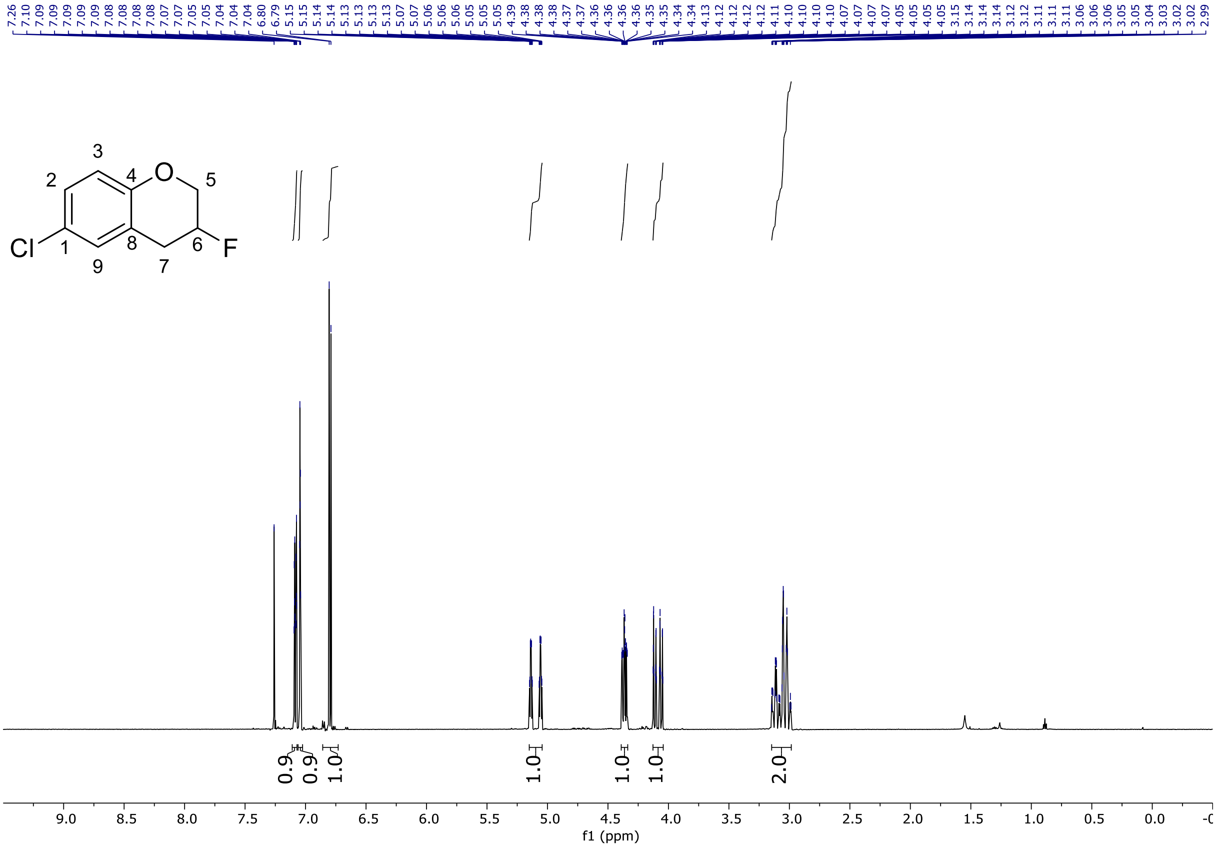

^{13}C{^{1}H} NMR (151 MHz, $CDCl_3$):

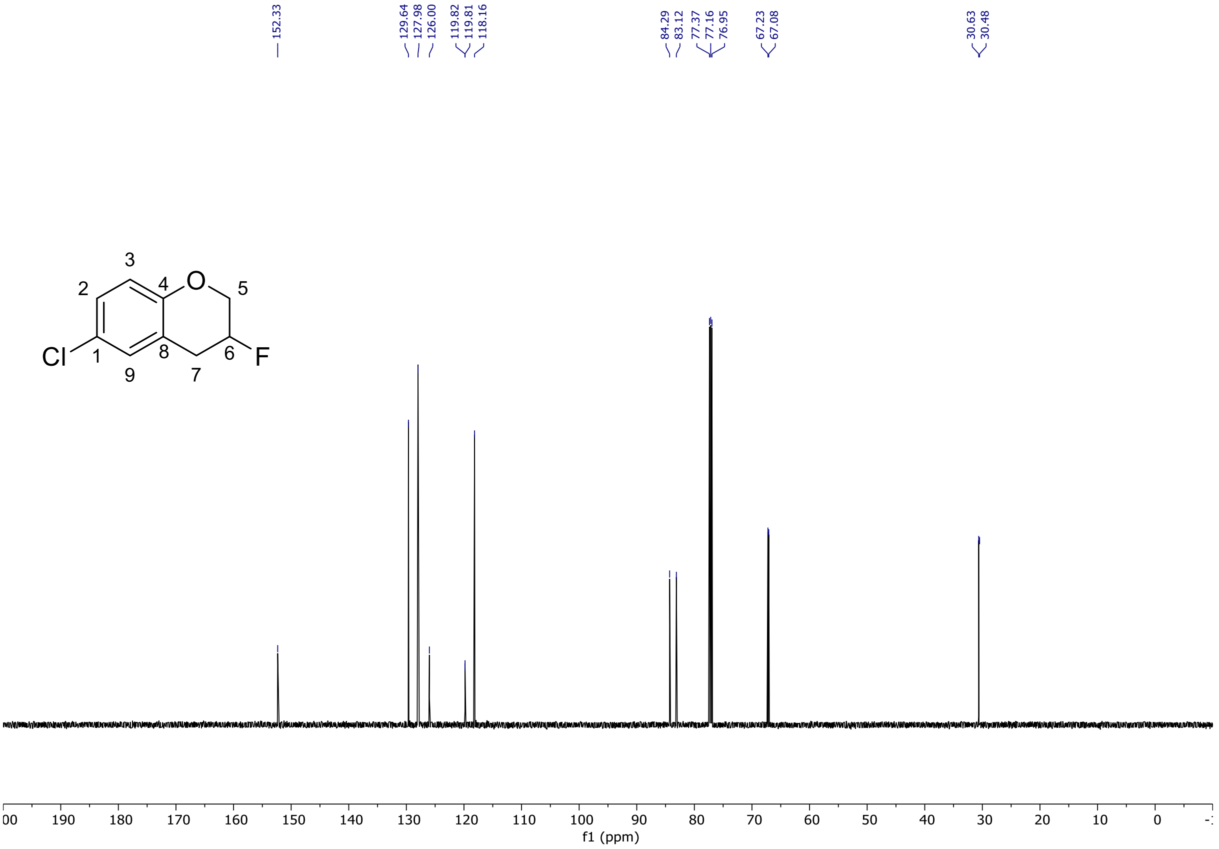

$^{19}F\{^{1}H\}$ NMR (564 MHz, $CDCl_3$):

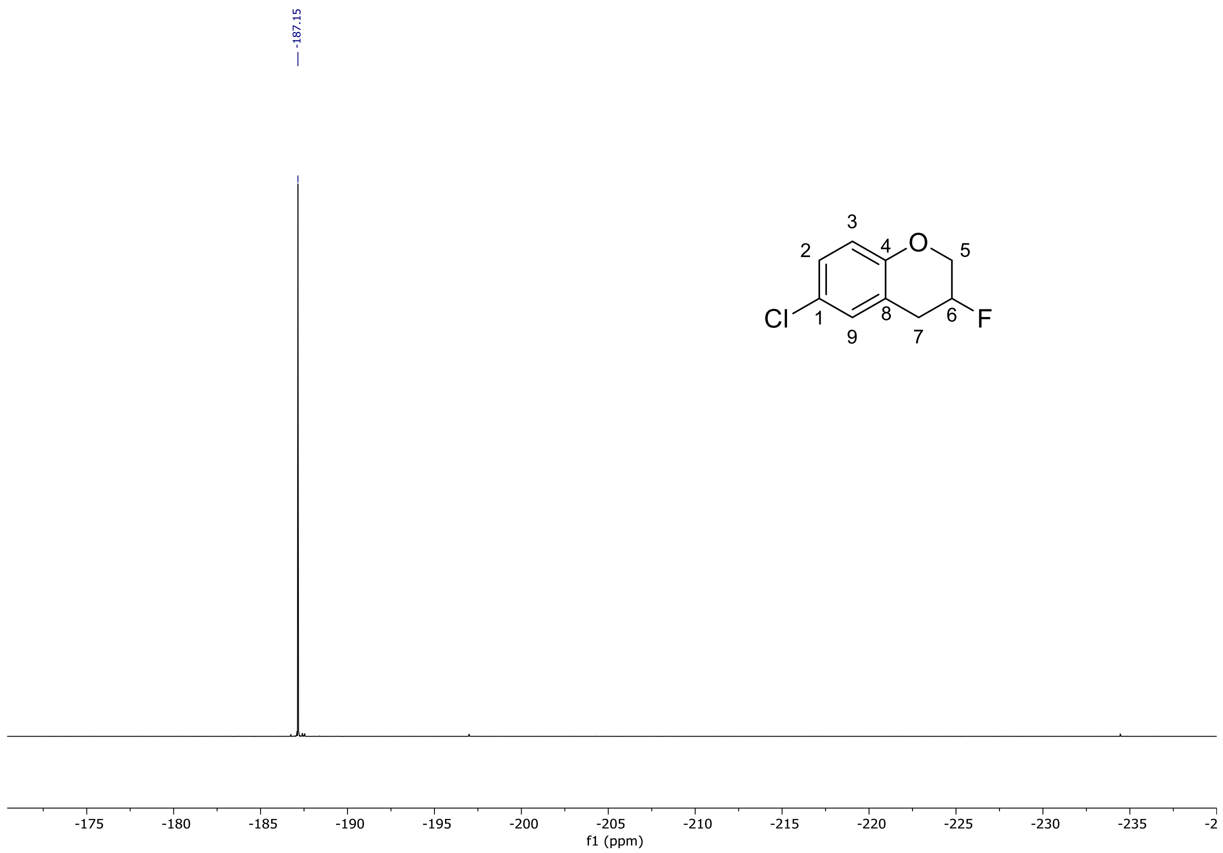

^{19}F NMR (564 MHz, $CDCl_3$):

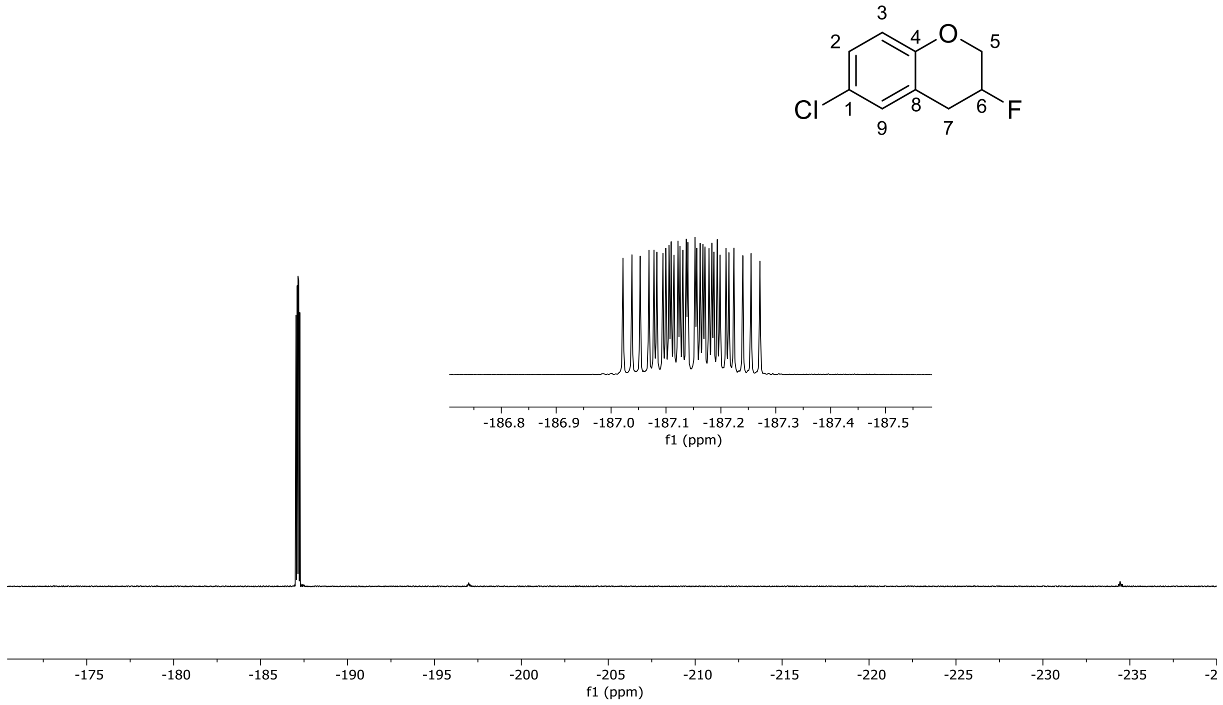

3,6-Difluorochromane (9)

^{1}H NMR (400 MHz, $CDCl_3$):

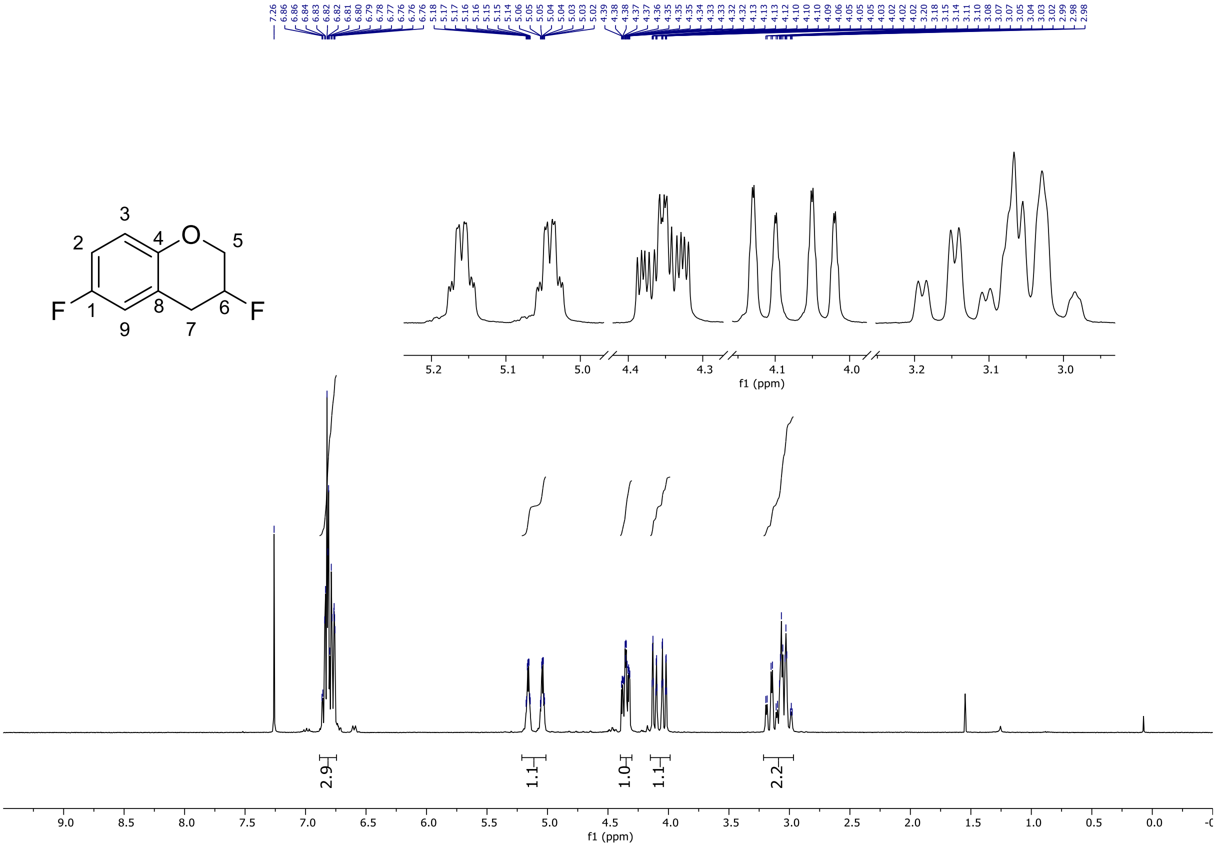

$^{13}C\{^1H\}$ NMR (151 MHz, $CDCl_3$):

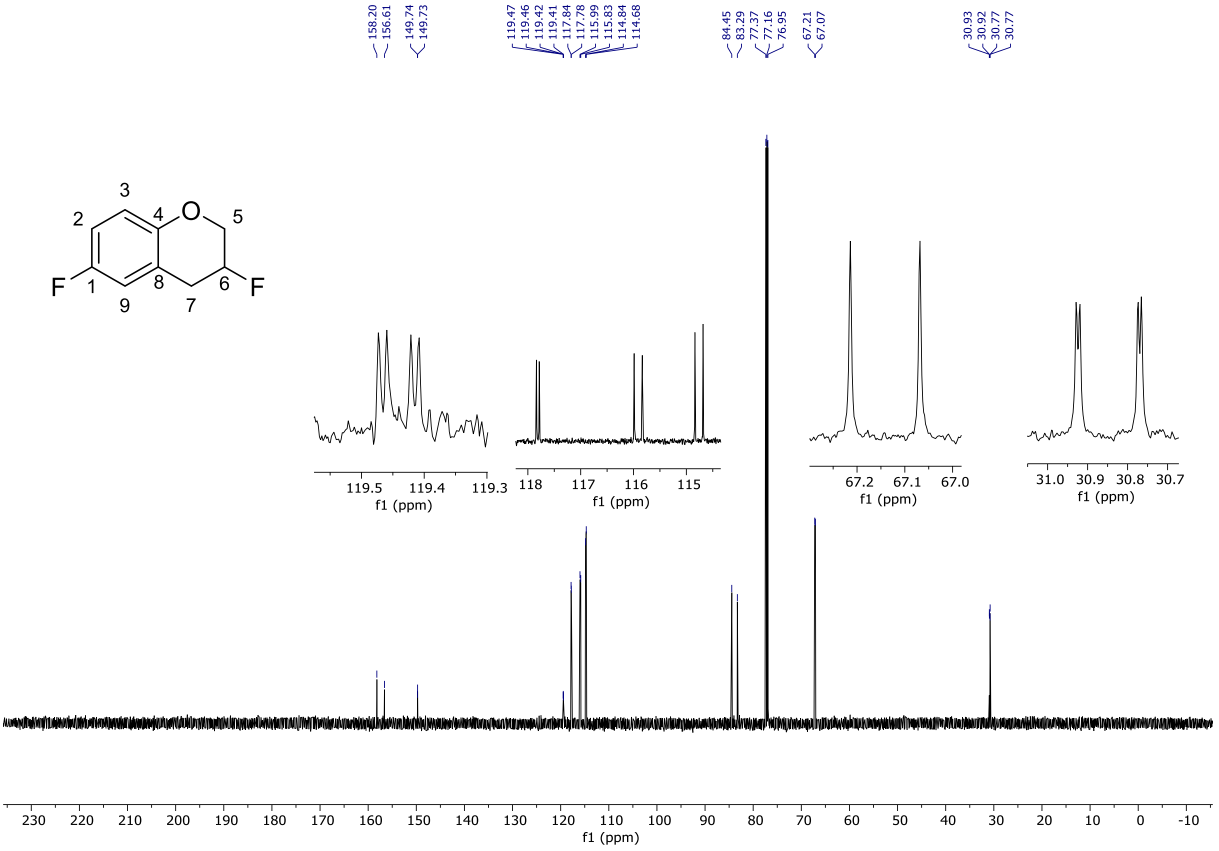

^{19}F{^{1}H} NMR (564 MHz, $CDCl_3$):

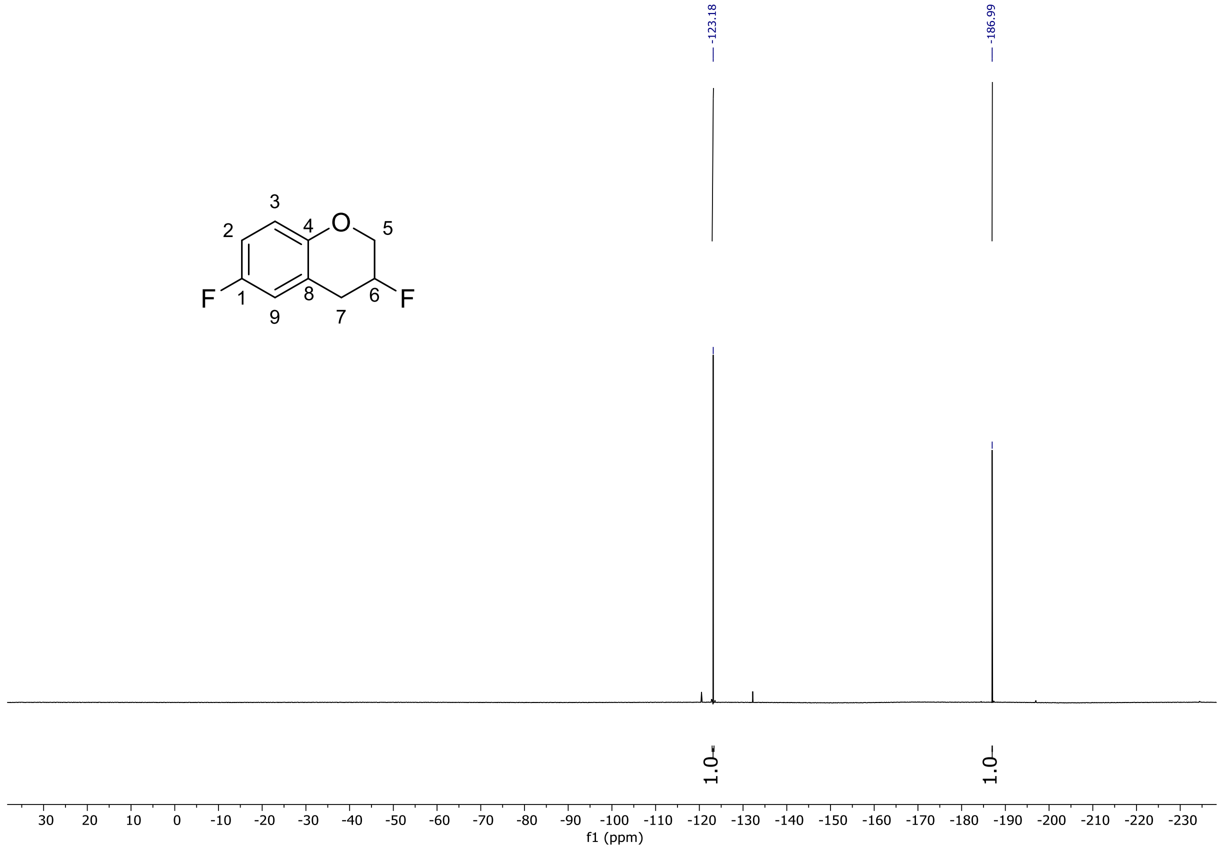

^{19}F NMR (564 MHz, $CDCl_3$):

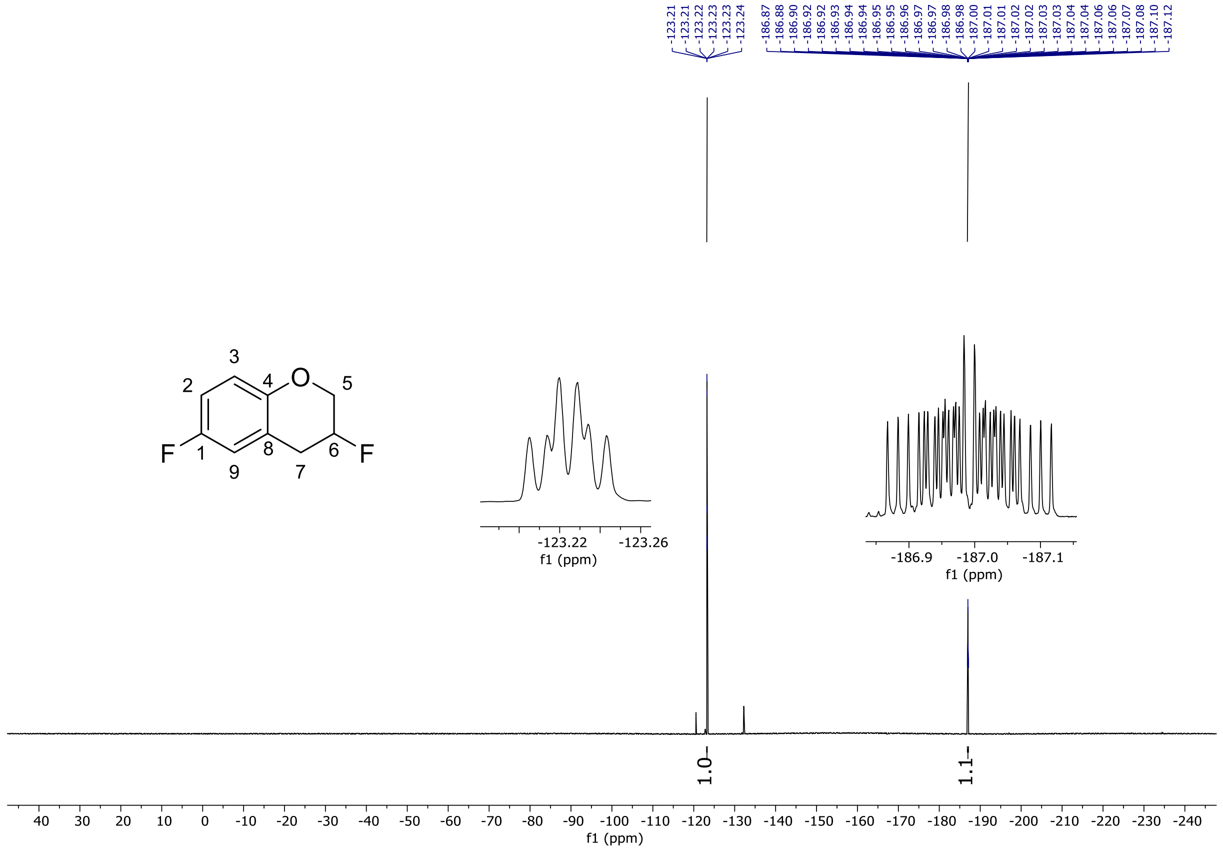

Methyl 3-fluorochromane-6-carboxylate (14)

^{1}H NMR (500 MHz, $CDCl_3$):

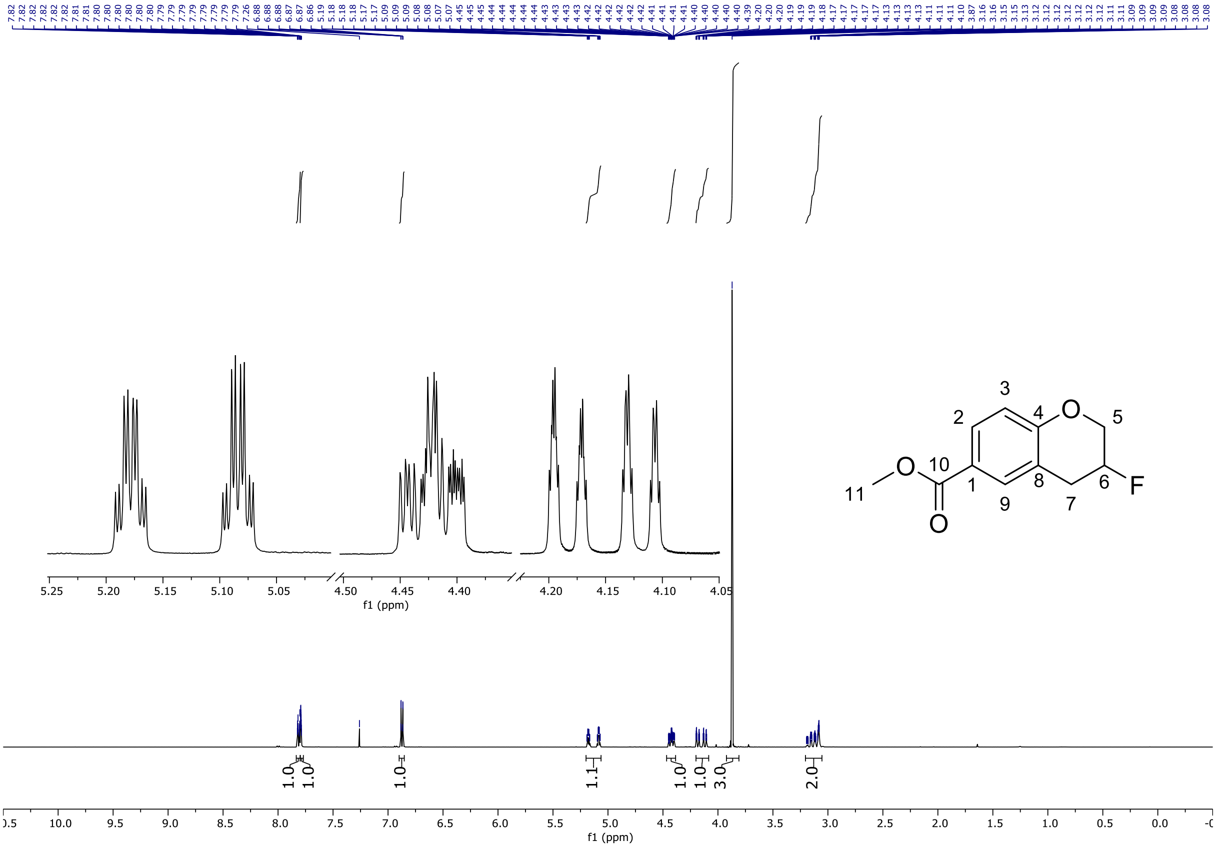

^{13}C{^{1}H} NMR (126 MHz, $CDCl_3$):

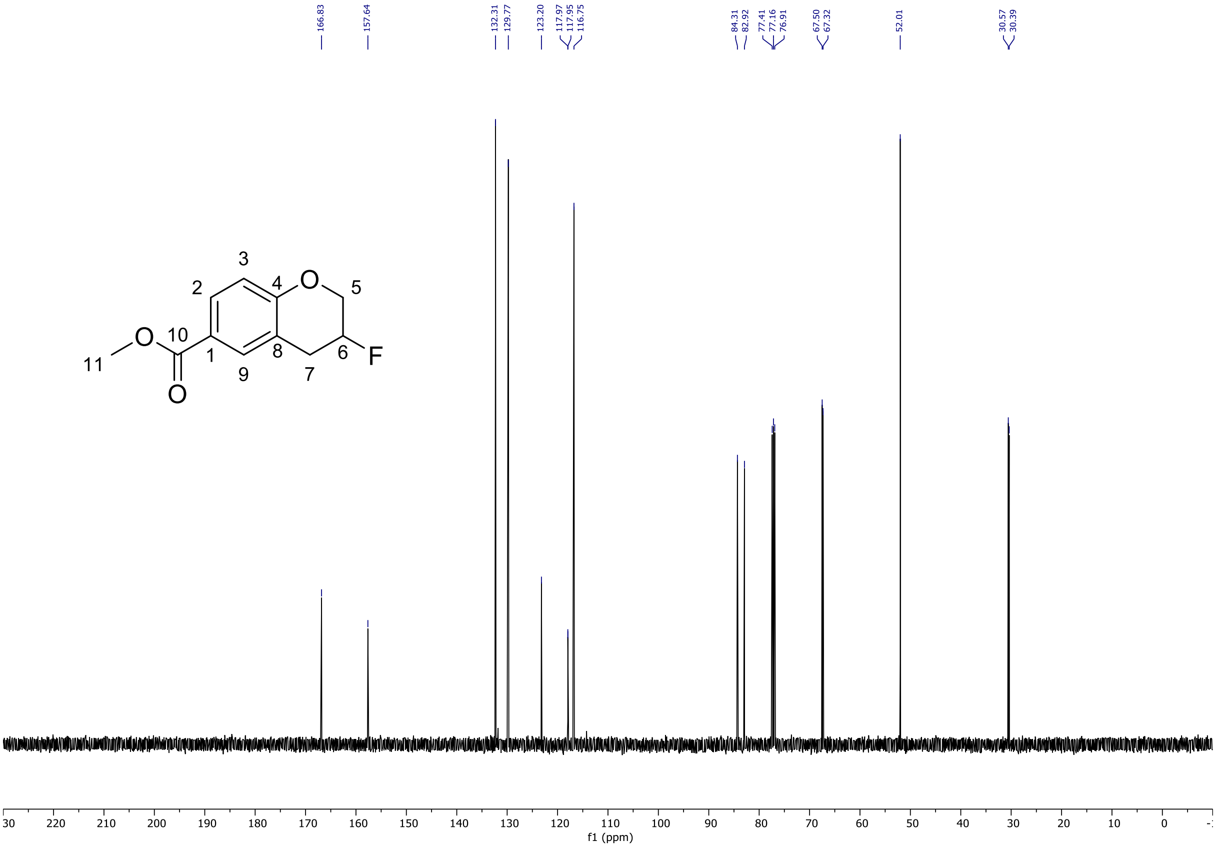

^{19}F{^{1}H} NMR (470 MHz, $CDCl_3$):

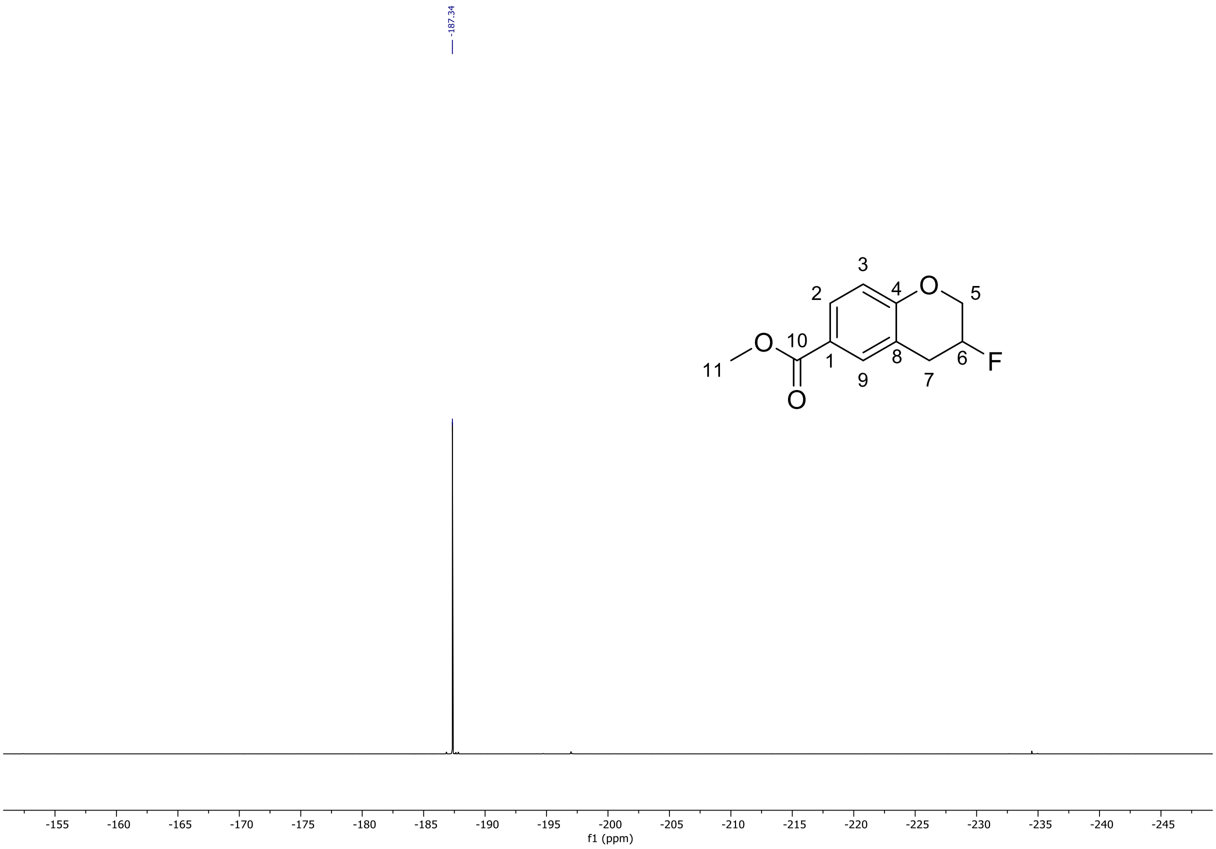

^{19}F NMR (470 MHz, $CDCl_3$):

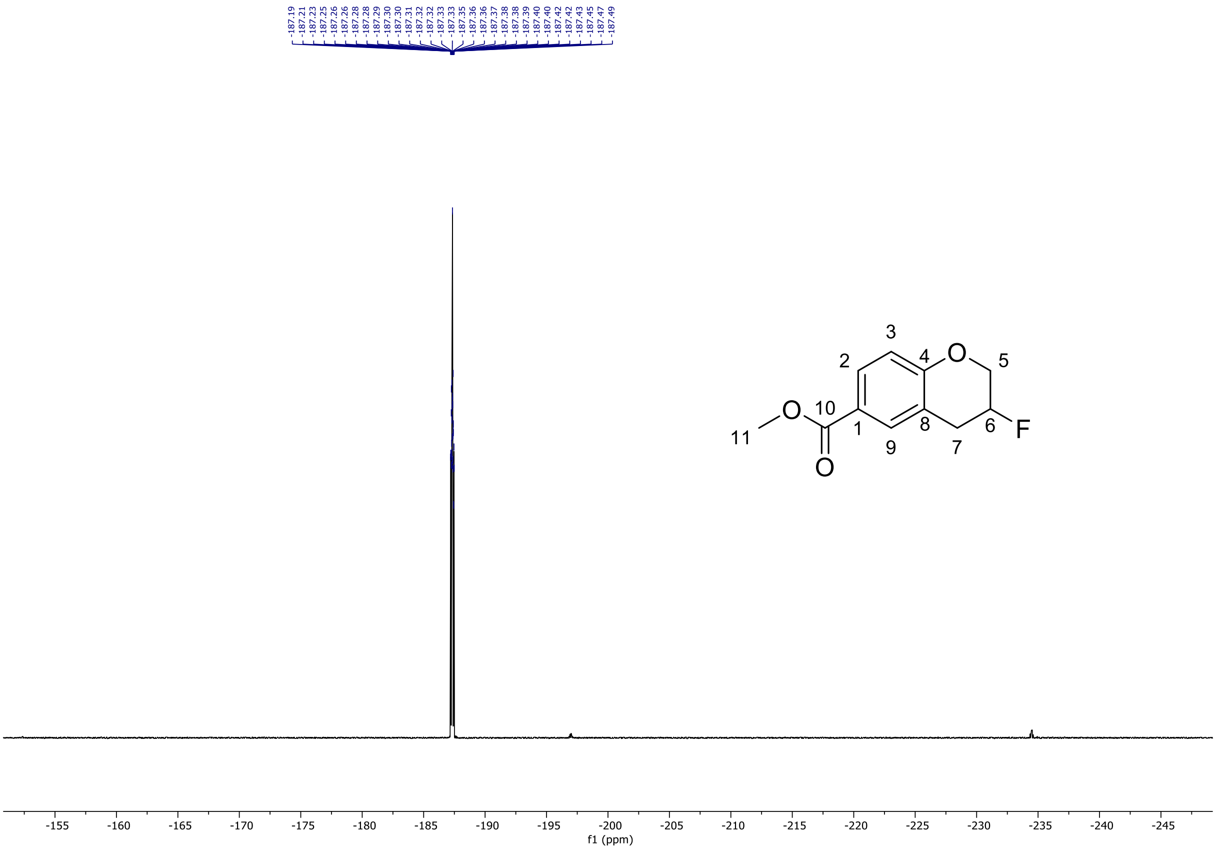

(3-Fluorochroman-6-yl)(phenyl)methanone (15)

^{1}H NMR (400 MHz, $CDCl_3$):

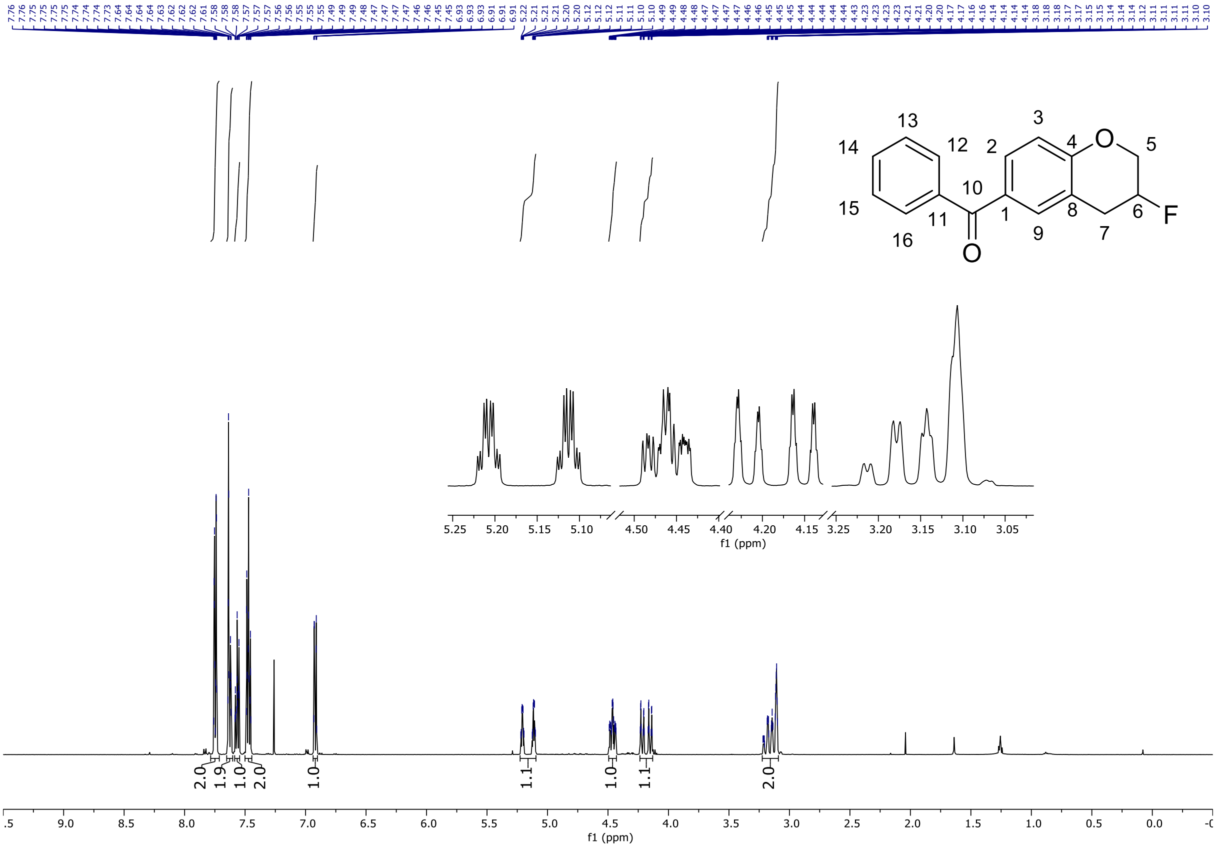

^{13}C{^{1}H} NMR (151 MHz, $CDCl_3$):

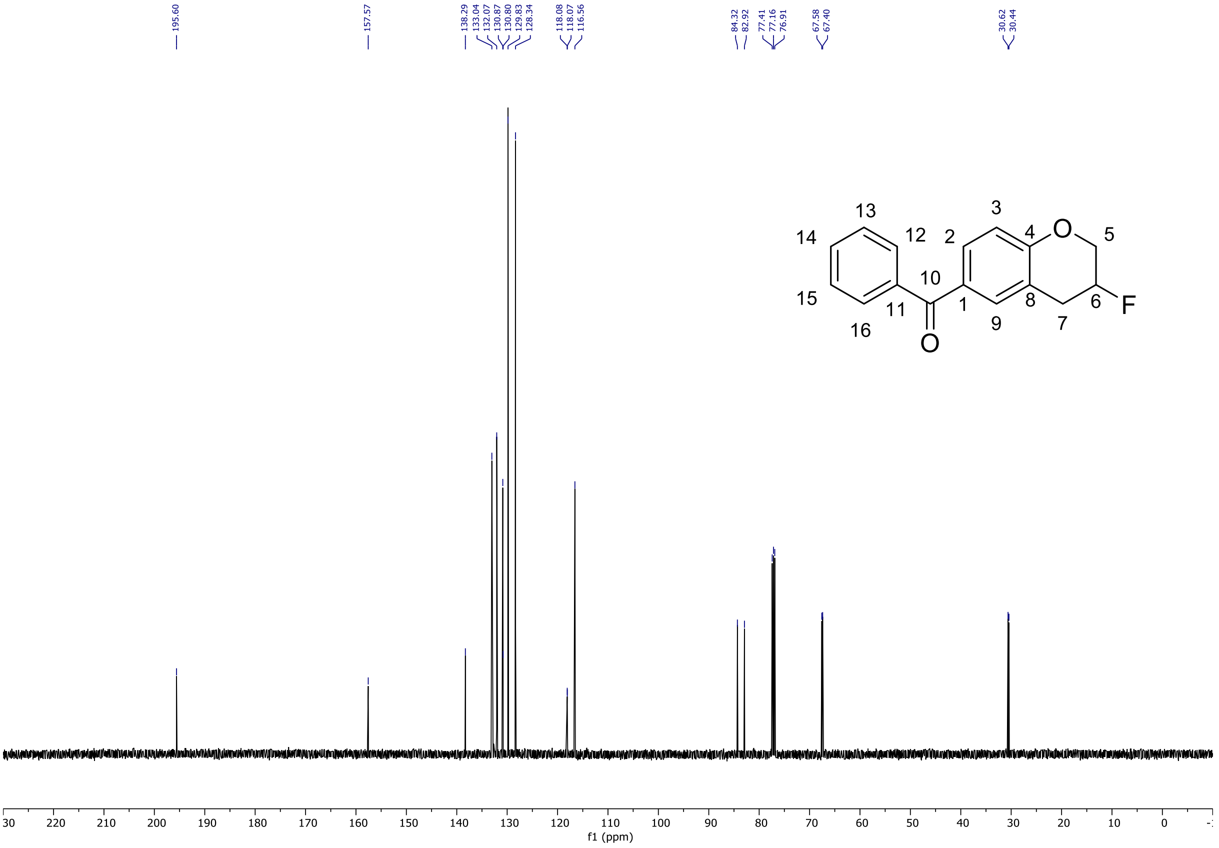

^{19}F{^{1}H} NMR (564 MHz, $CDCl_3$):

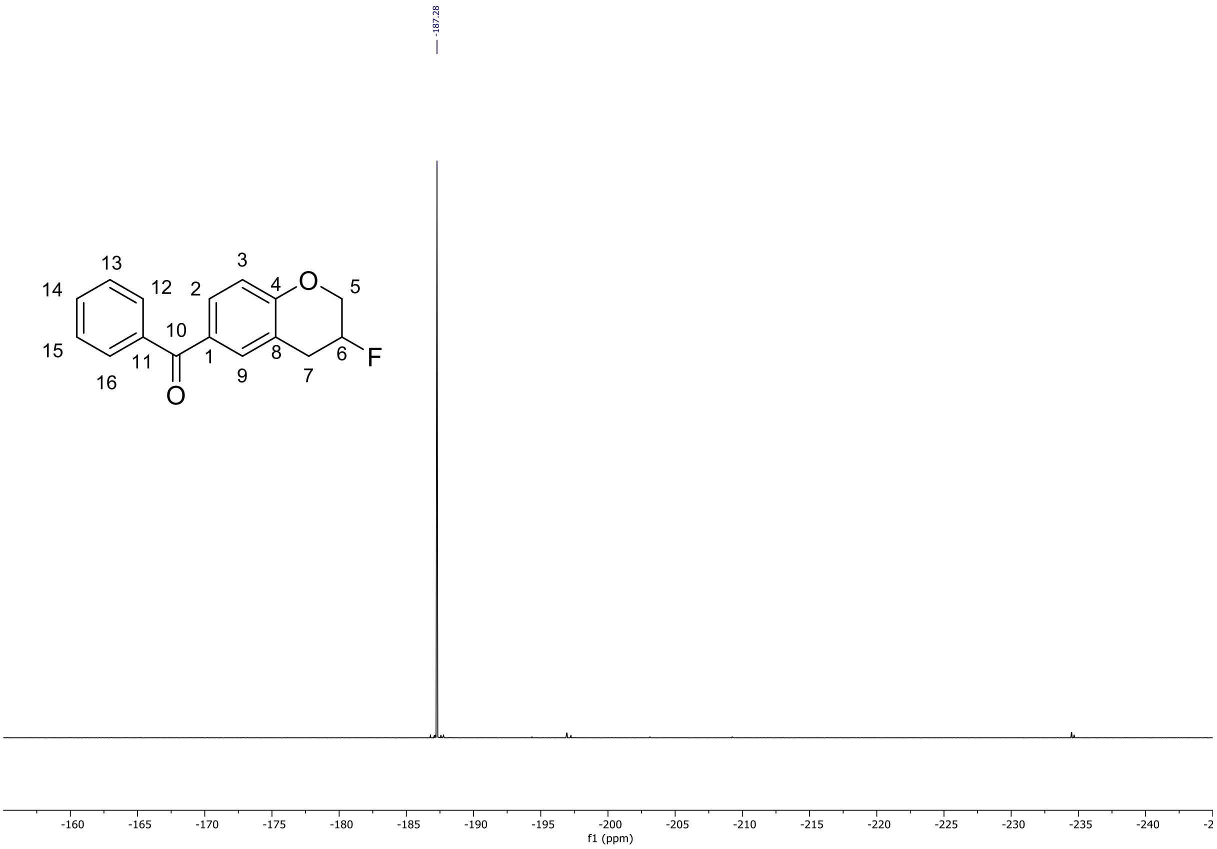

^{19}F NMR (564 MHz, $CDCl_3$):

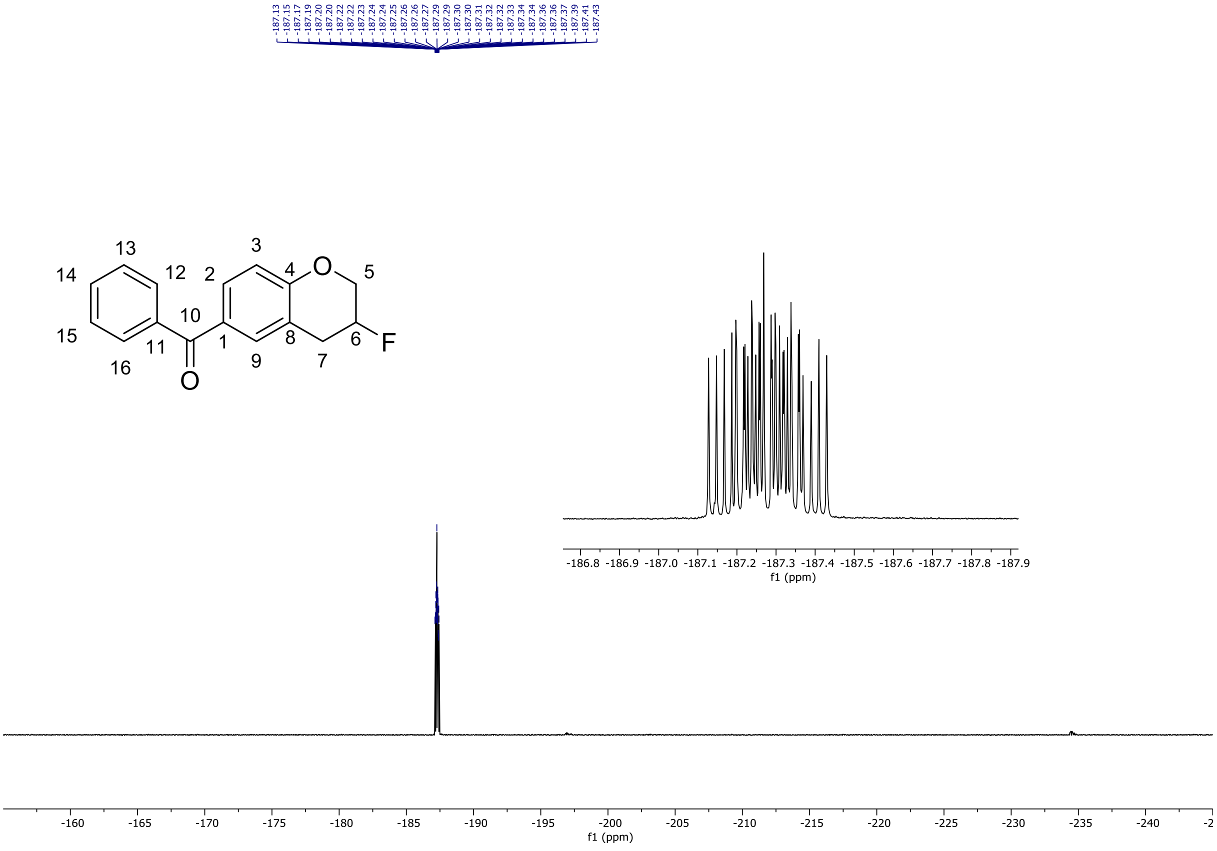

5,7-Dibromo-3-fluorochromane (10)

^{1}H NMR (599 MHz, $CDCl_3$):

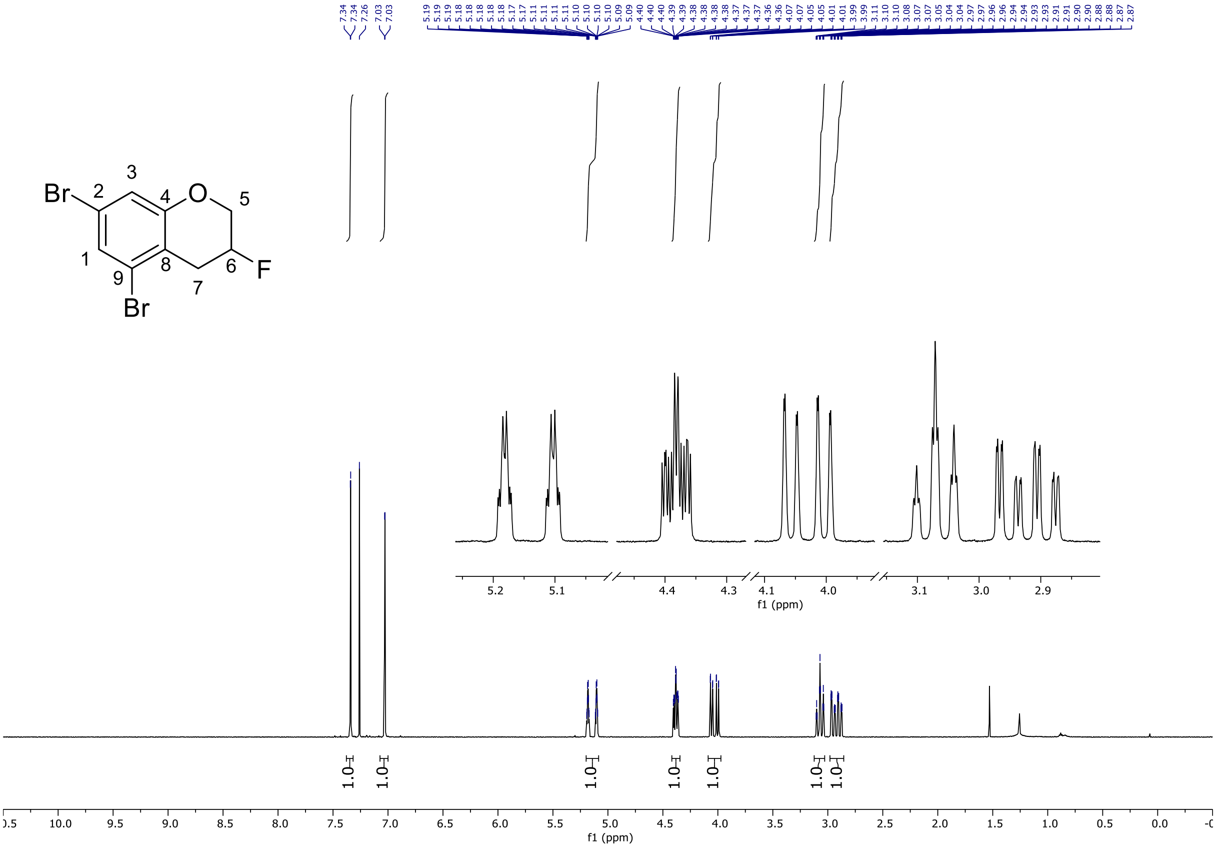

^{13}C{^{1}H} NMR (151 MHz, $CDCl_3$):

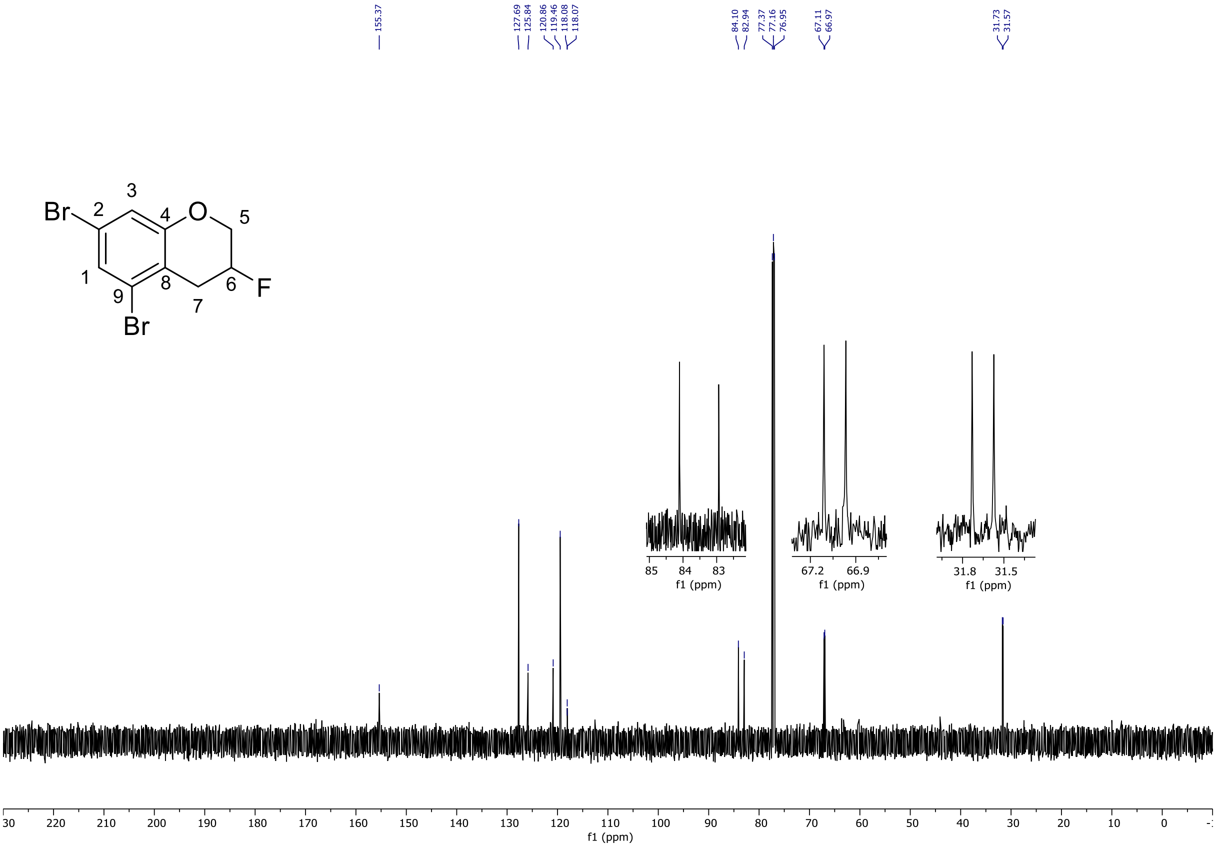

^{19}**F{^{1}H} NMR** (564 MHz, $CDCl_3$):

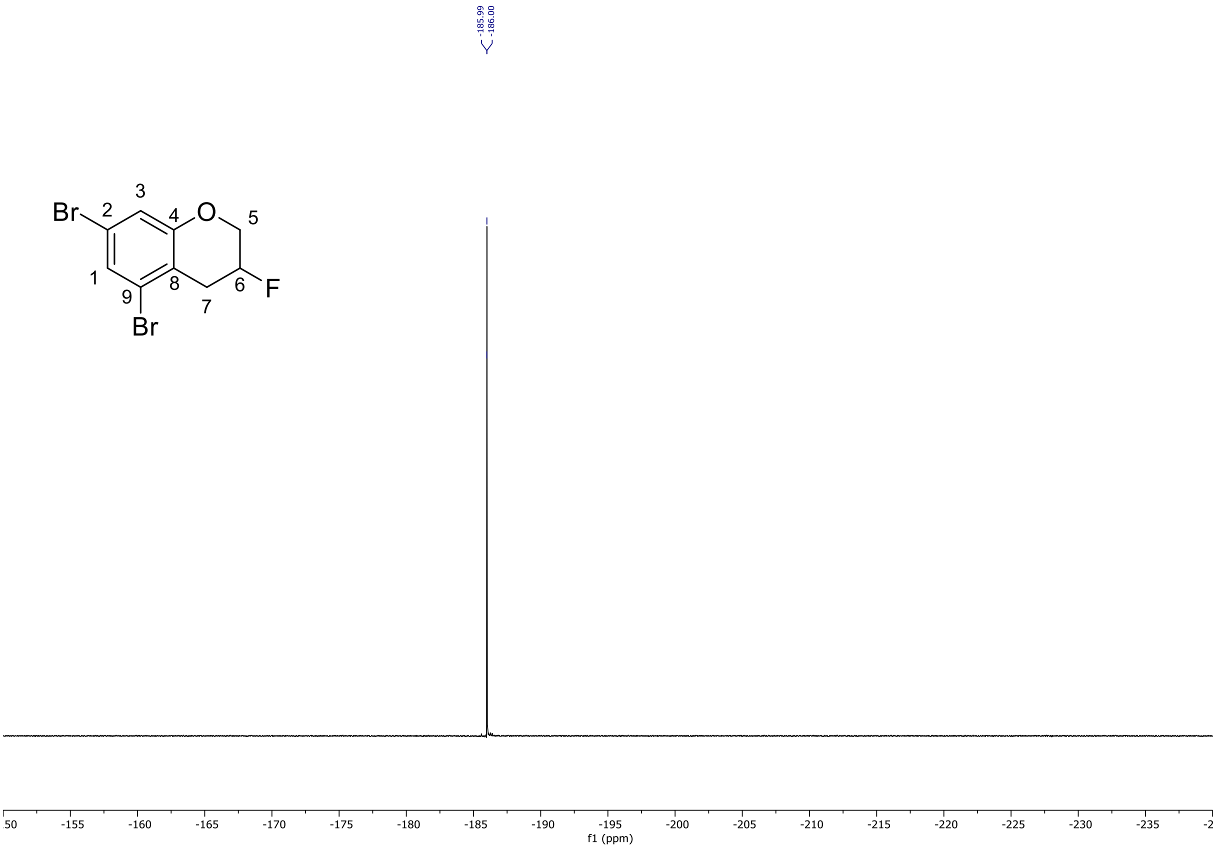

^{19}**F NMR** (564 MHz, $CDCl_3$):

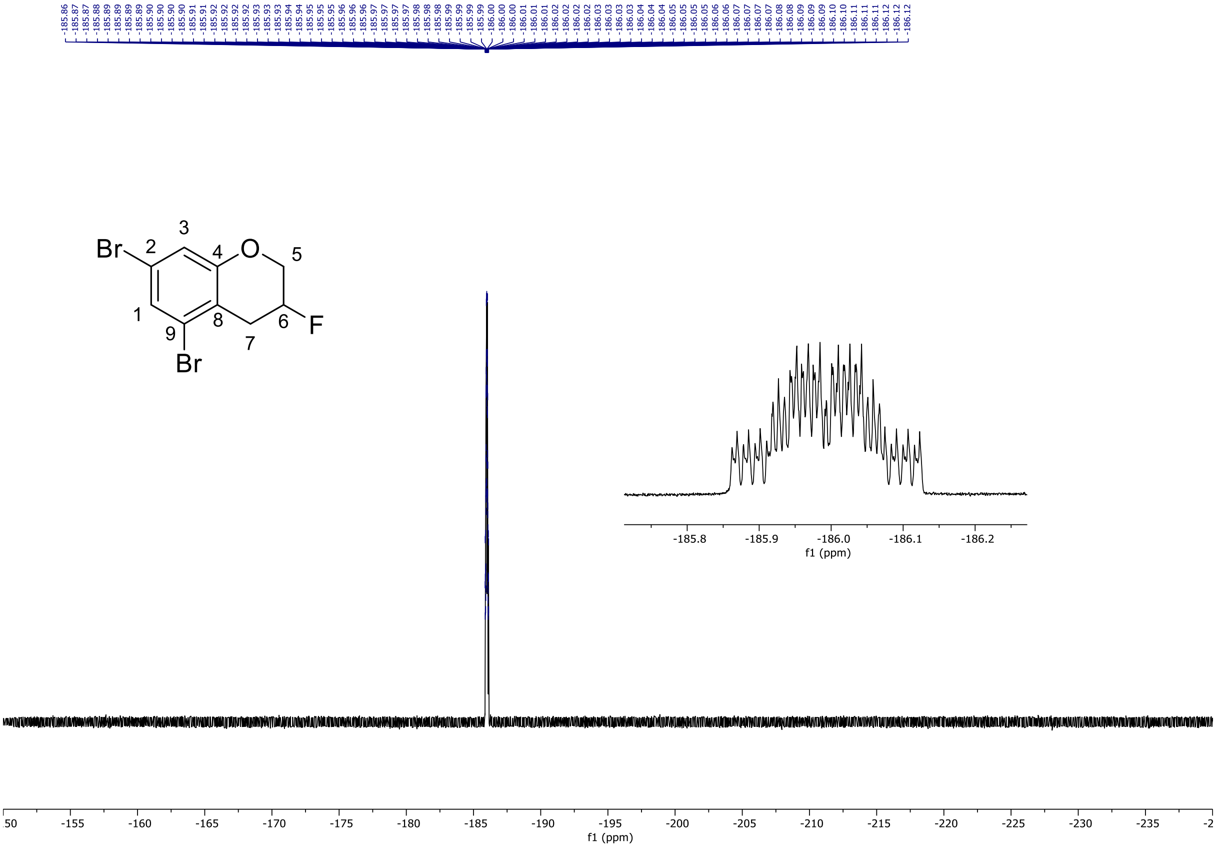

5,7-Dichloro-3-fluorochromane (11)

^{1}H NMR (599 MHz, $CDCl_3$):

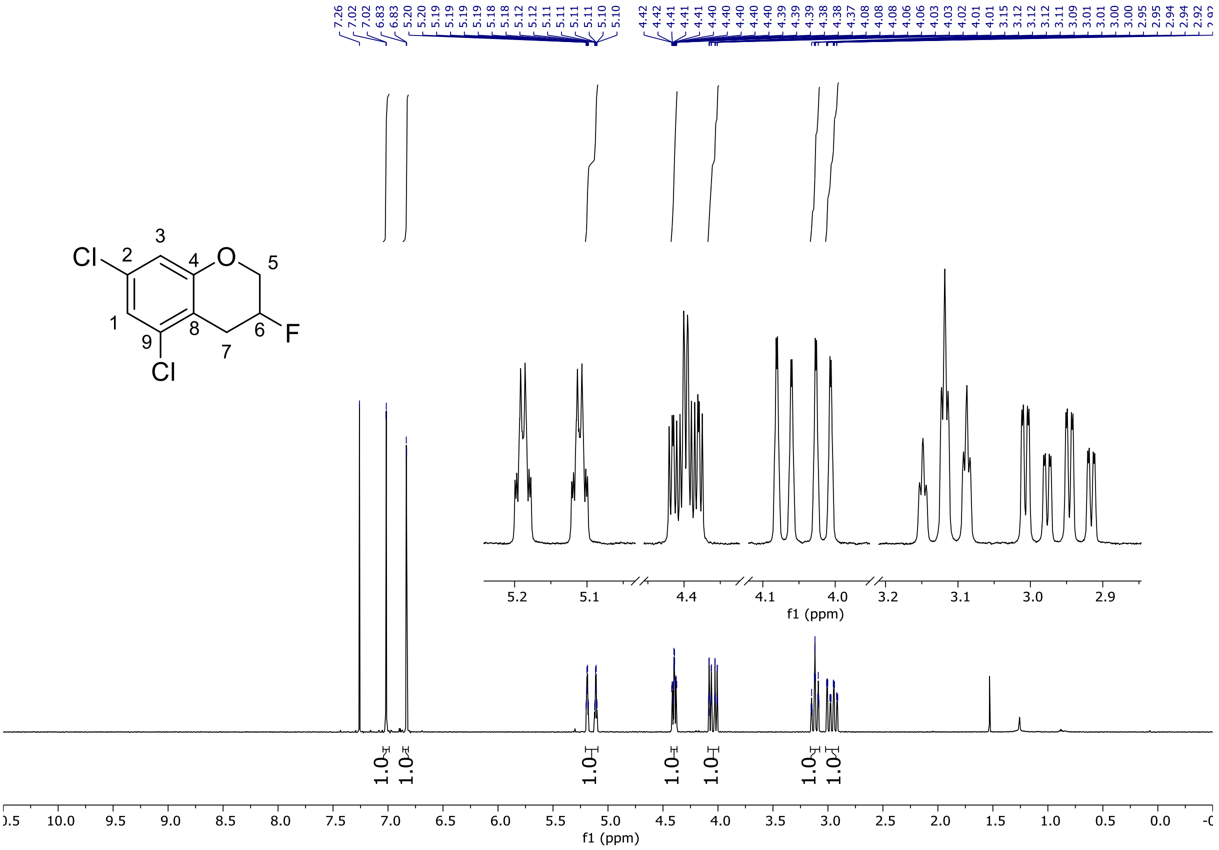

$^{13}C\{^{1}H\}$ NMR (151 MHz, $CDCl_3$):

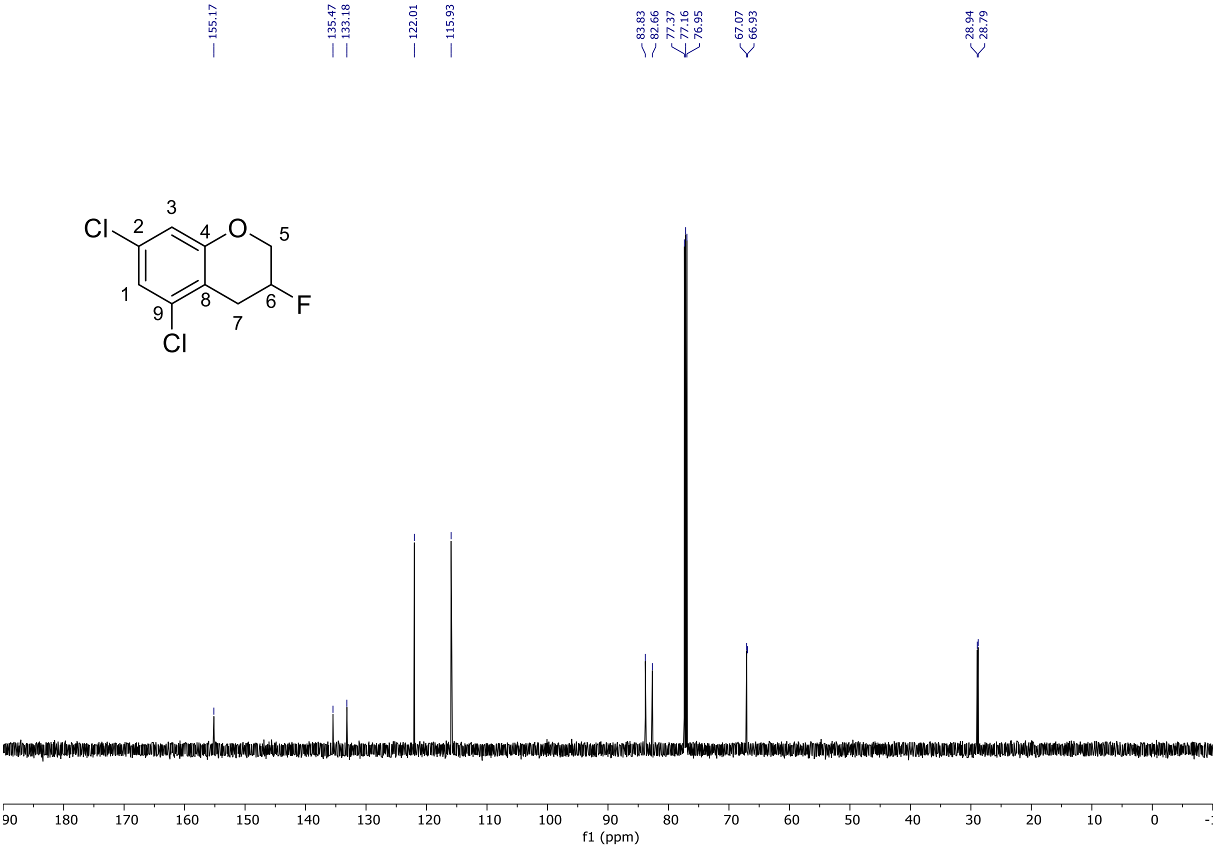

^{19}F{^{1}H} NMR (564 MHz, $CDCl_3$):

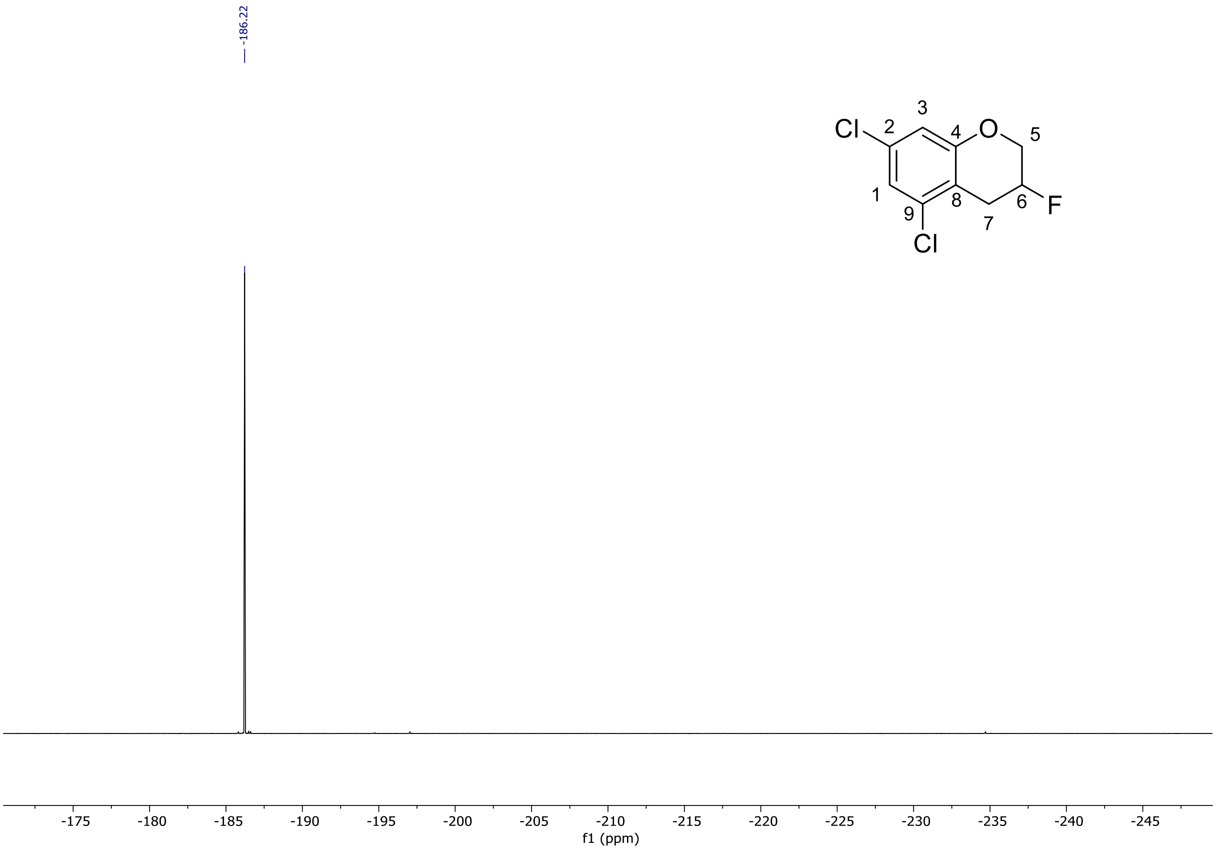

^{19}F NMR (564 MHz, $CDCl_3$):

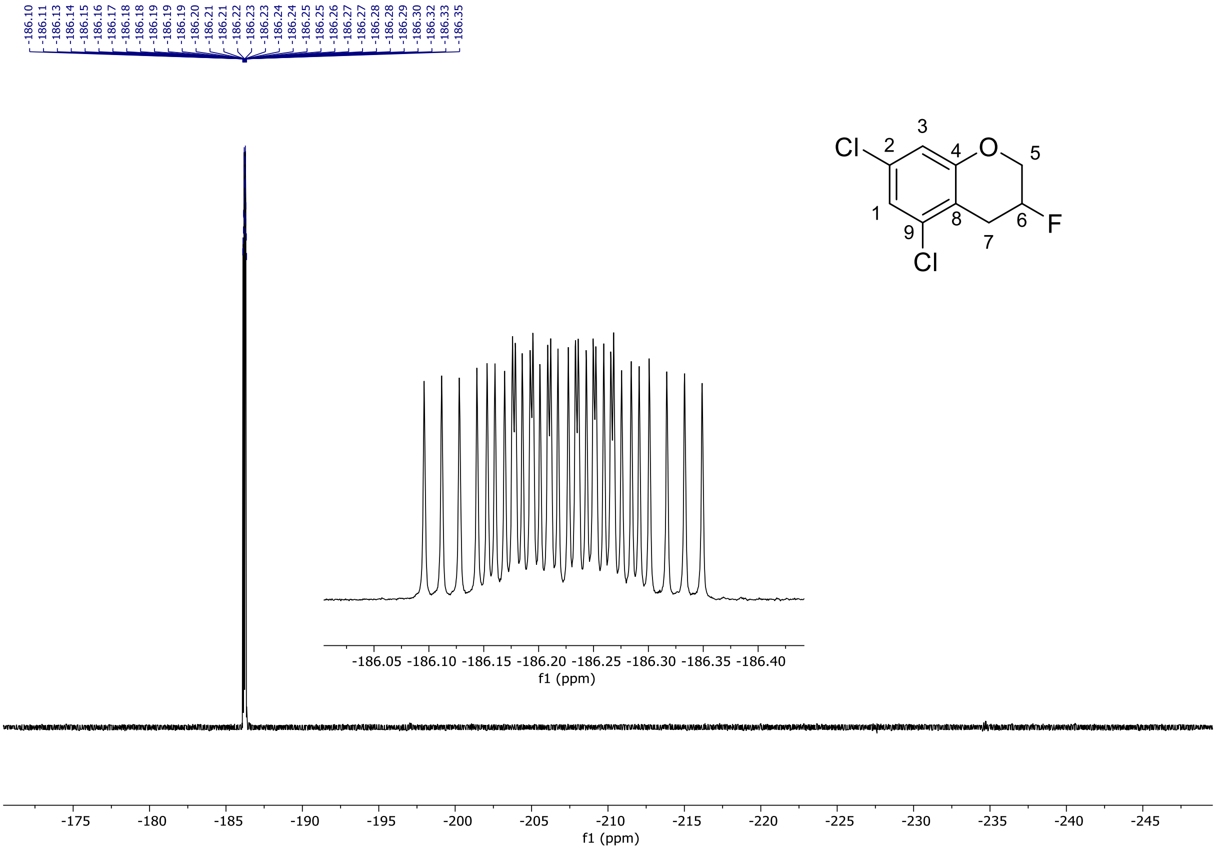

5,8-Dichloro-3-fluorochromane (12)

^{1}H NMR (400 MHz, $CDCl_3$):

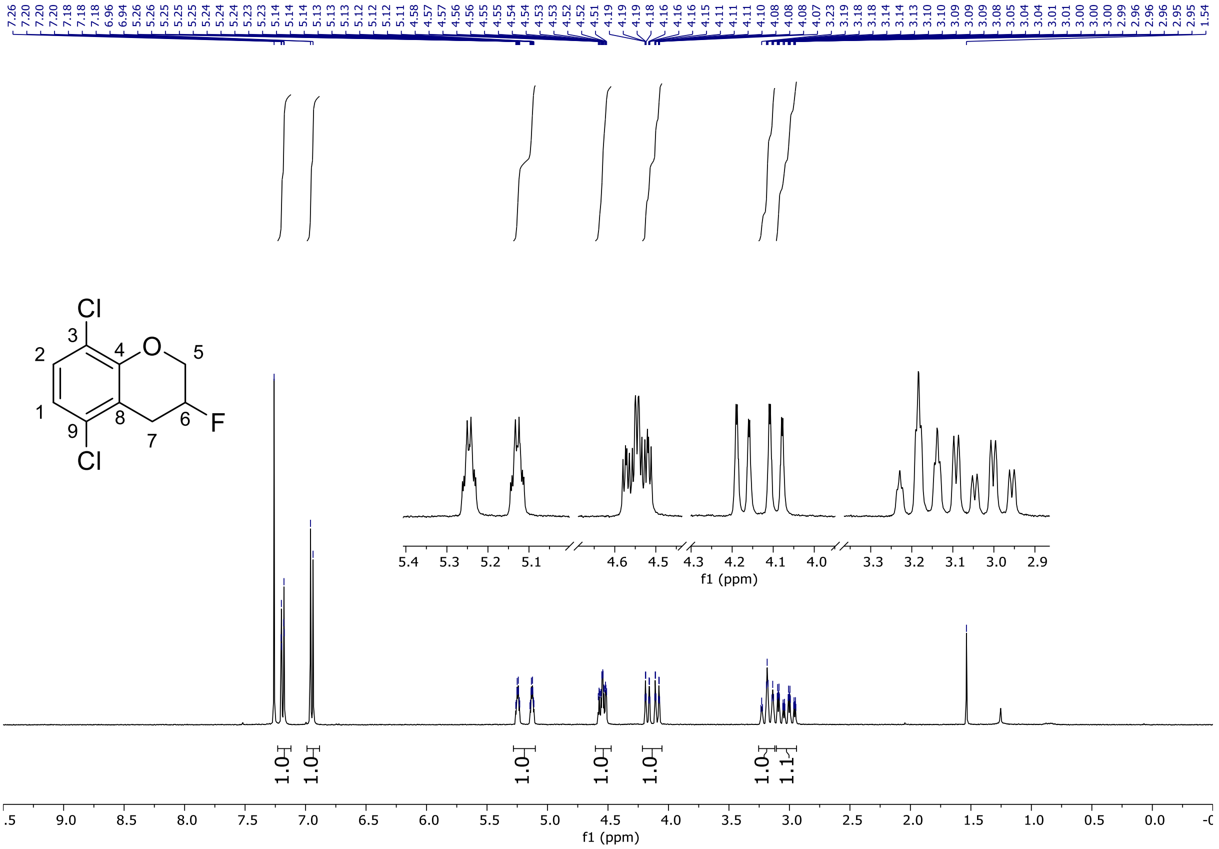

^{13}C{^{1}H} NMR (151 MHz, $CDCl_3$):

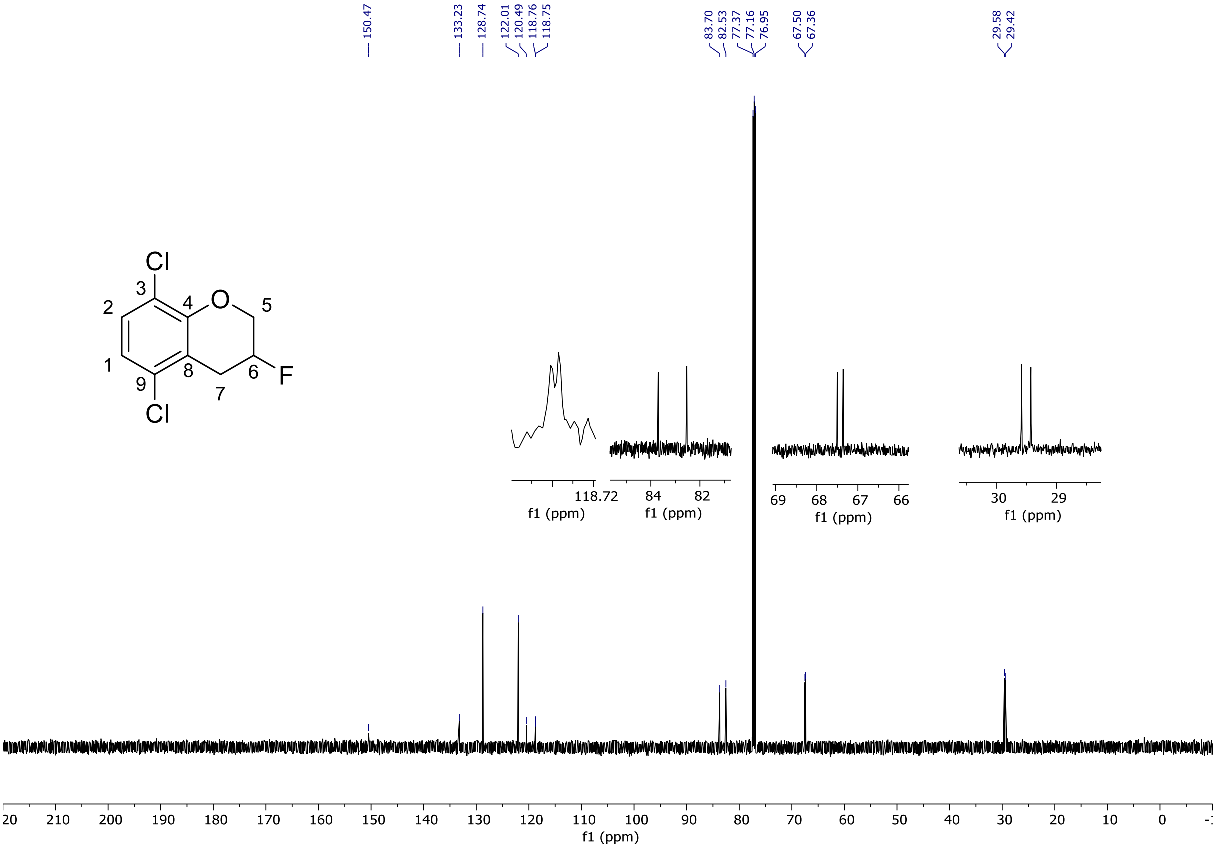

$^{19}F\{^1H\}$ NMR (564 MHz, $CDCl_3$):

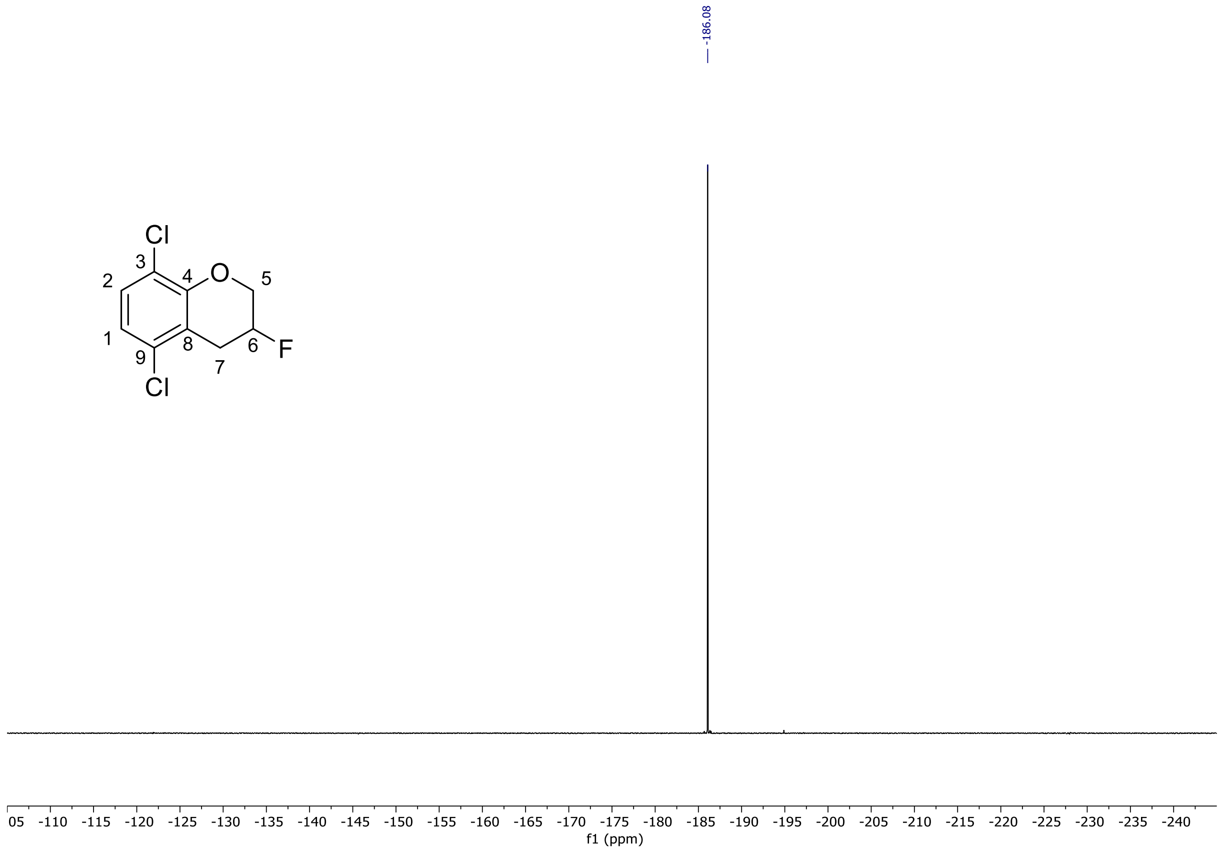

^{19}F NMR (564 MHz, $CDCl_3$):

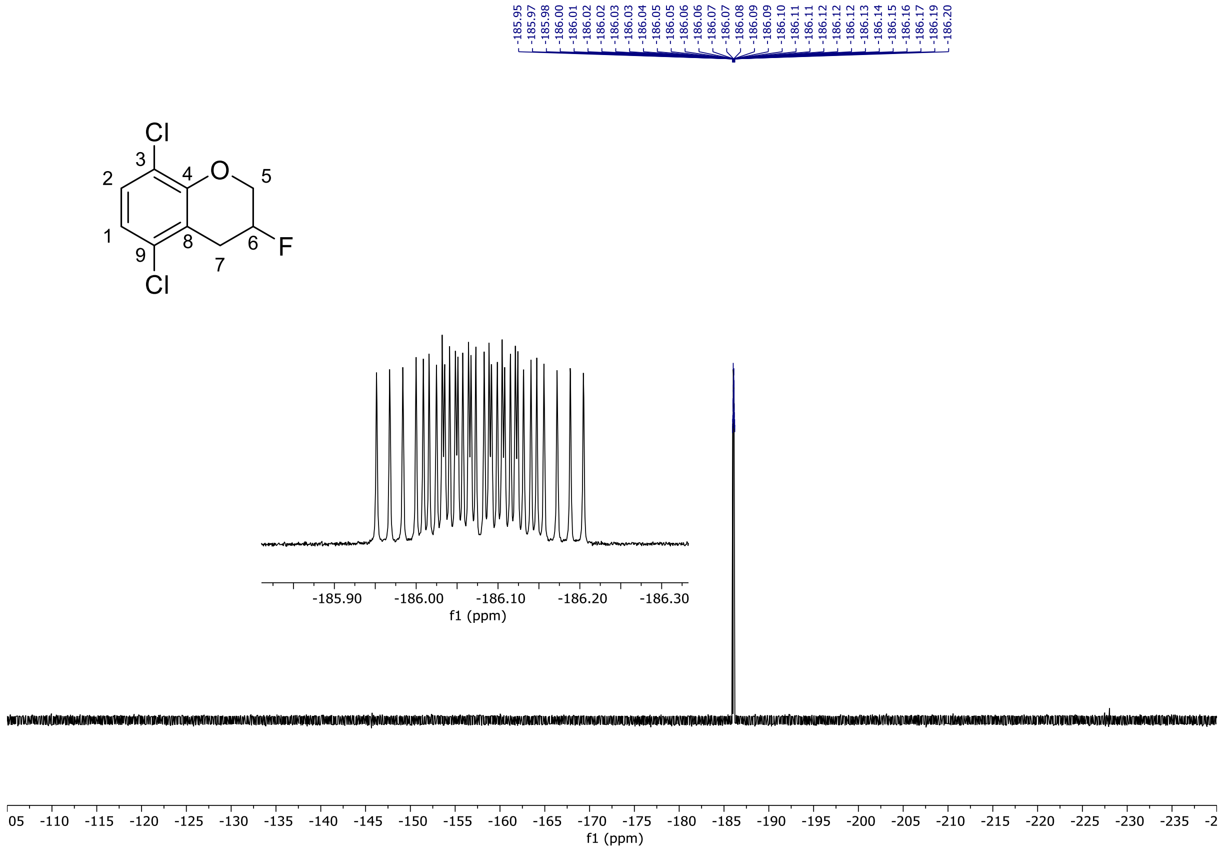

3-Fluoro-6-(trifluoromethyl)chromane (16)

^{1}H NMR (599 MHz, $CDCl_3$):

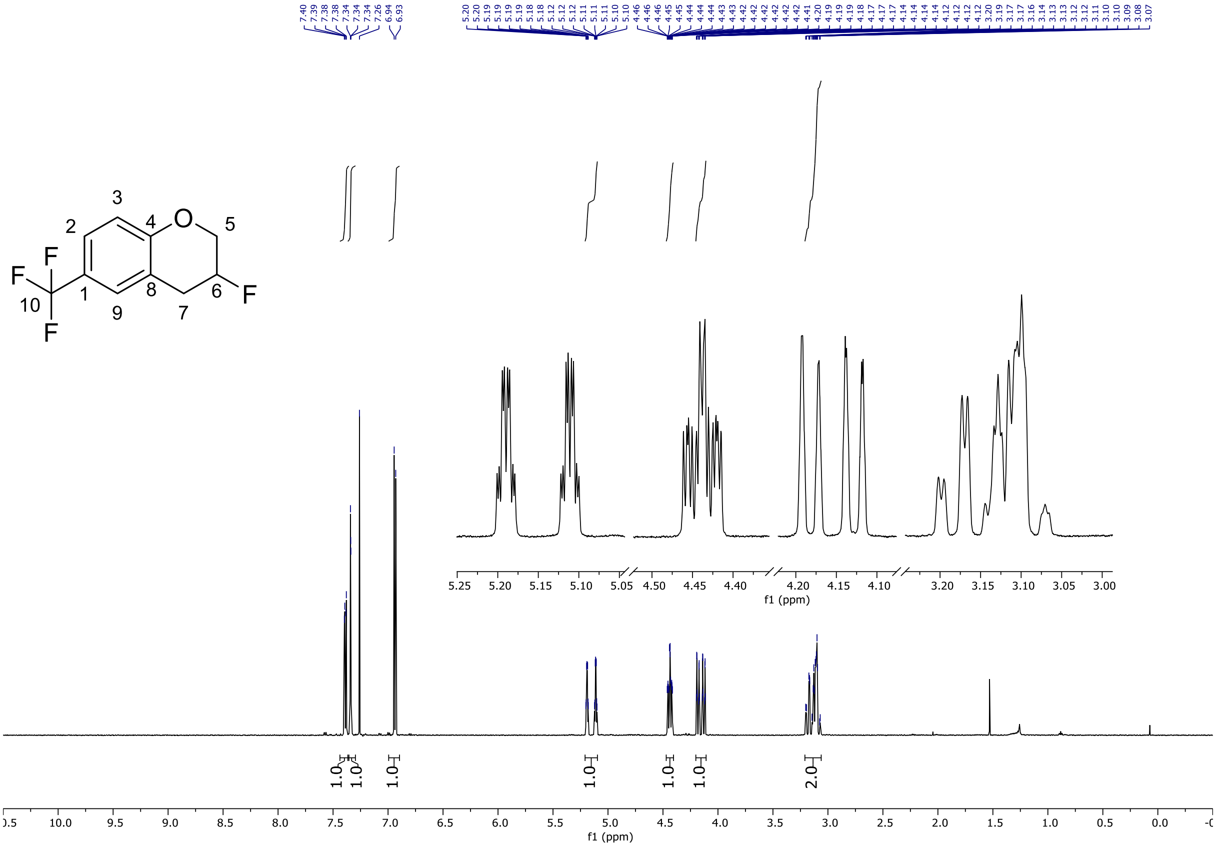

^{13}C{^{1}H} NMR (151 MHz, $CDCl_3$):

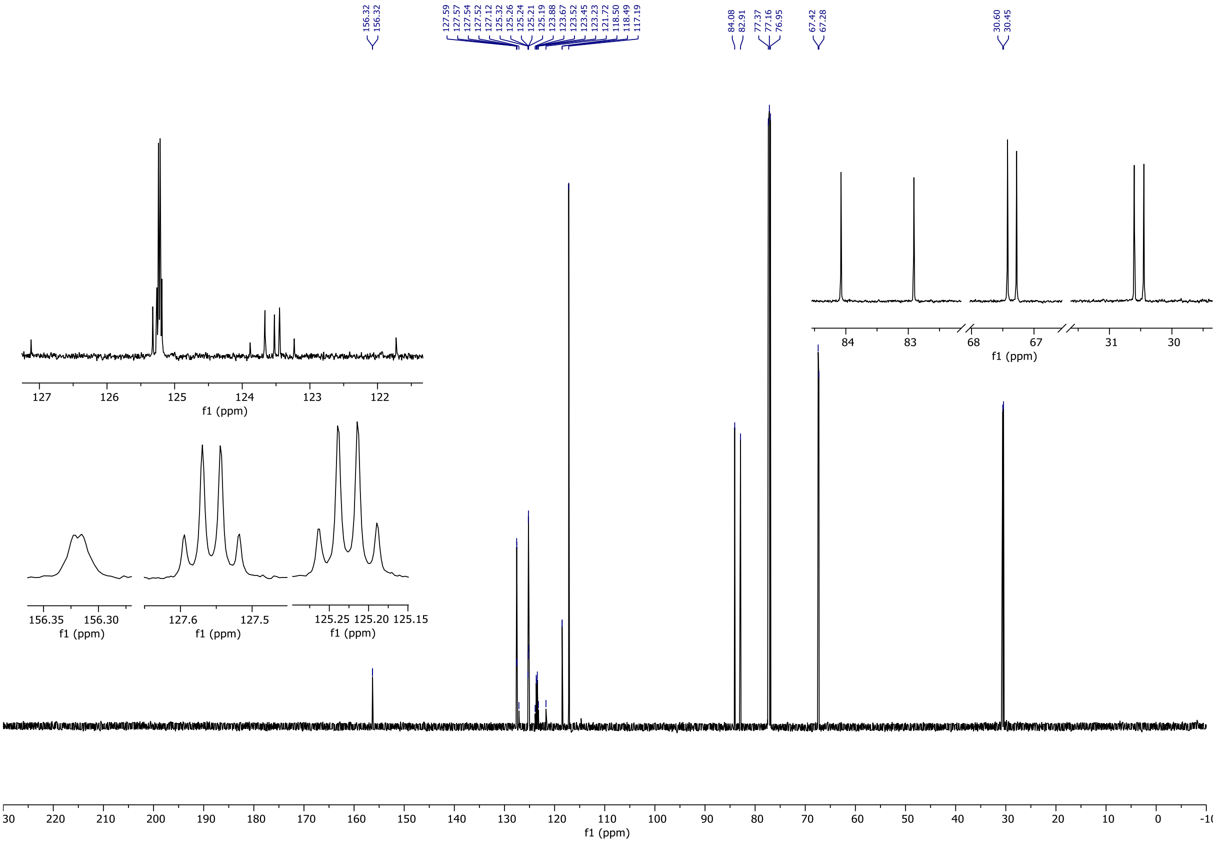

$^{19}F\{^{1}H\}$ NMR (564 MHz, $CDCl_3$):

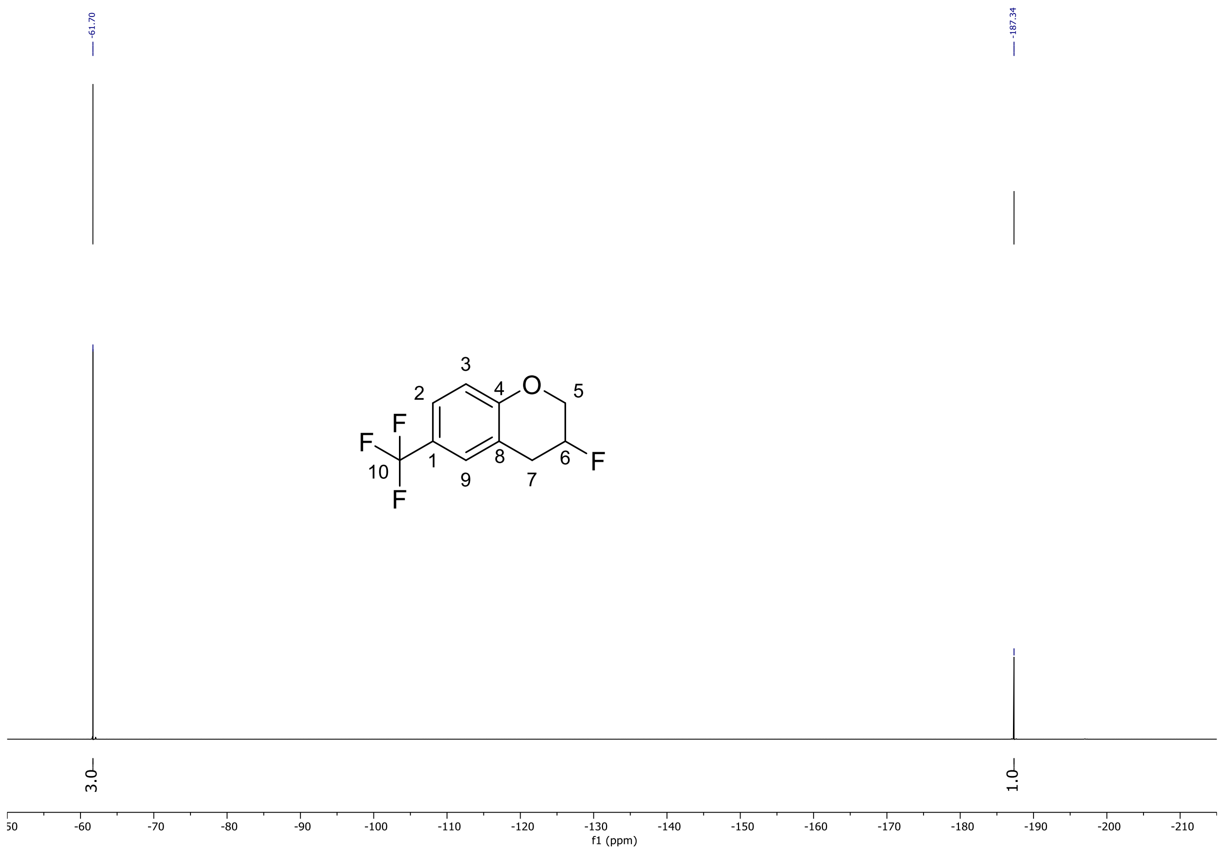

^{19}F NMR (564 MHz, $CDCl_3$):

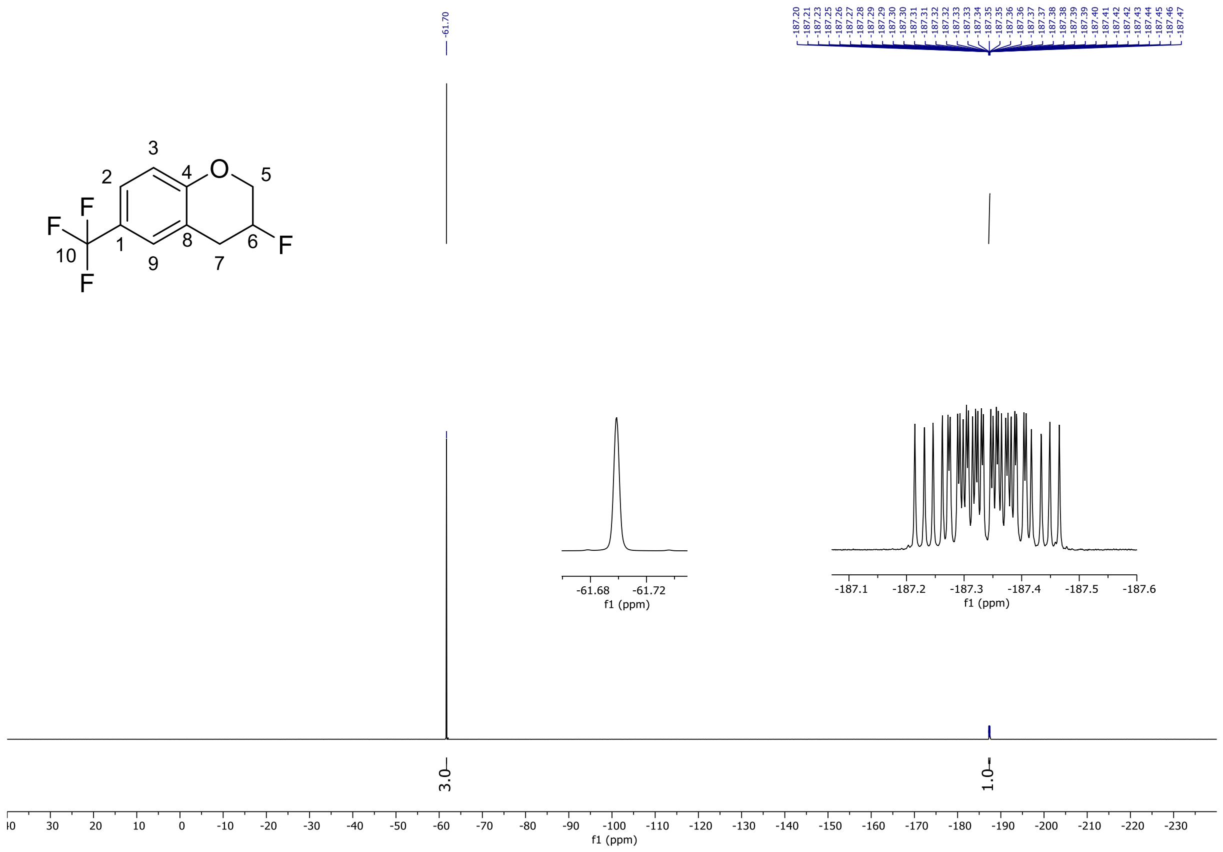

3-Fluorochromane-6-carbonitrile (17)

^{1}H NMR (599 MHz, $CDCl_3$) :

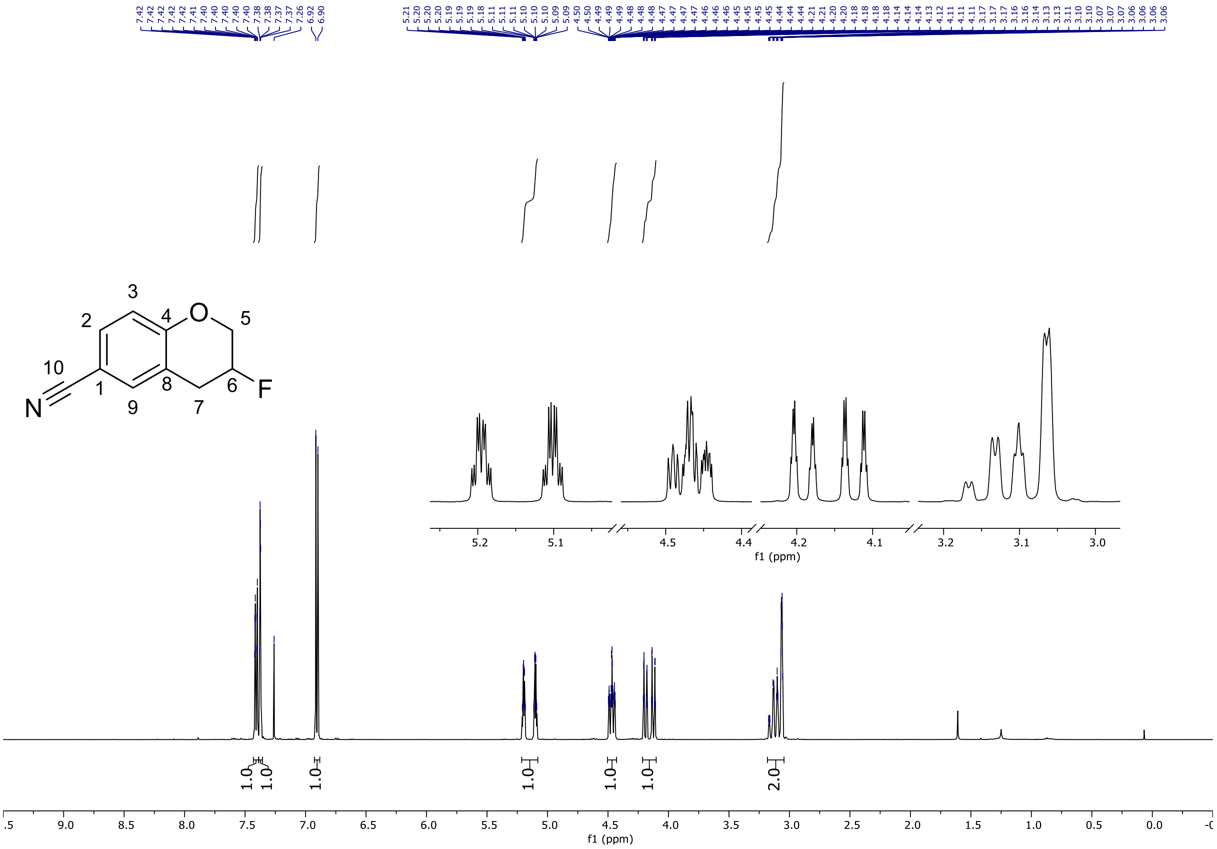

^{13}C{^{1}H} NMR (151 MHz, $CDCl_3$) :

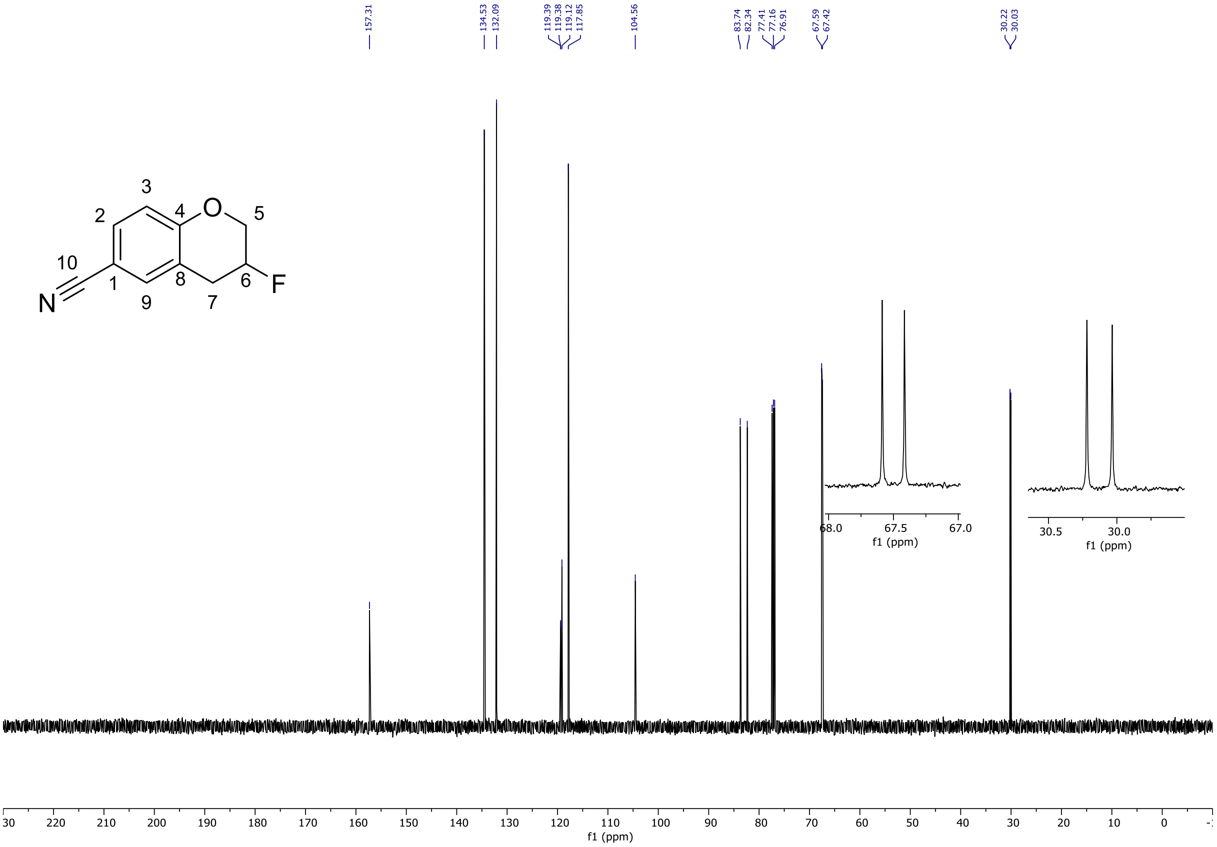

$^{19}F\{^{1}H\}$ NMR (564 MHz, $CDCl_3$):

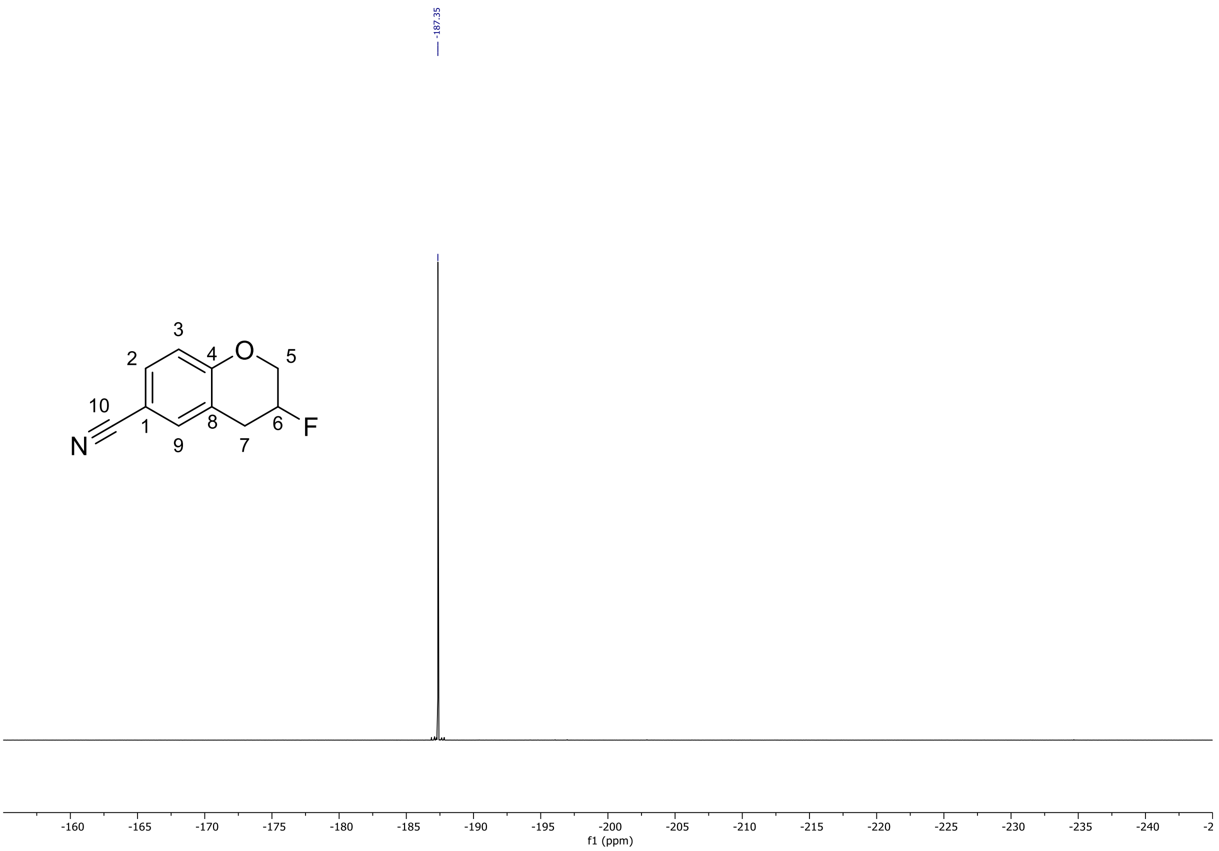

^{19}F NMR (564 MHz, $CDCl_3$):

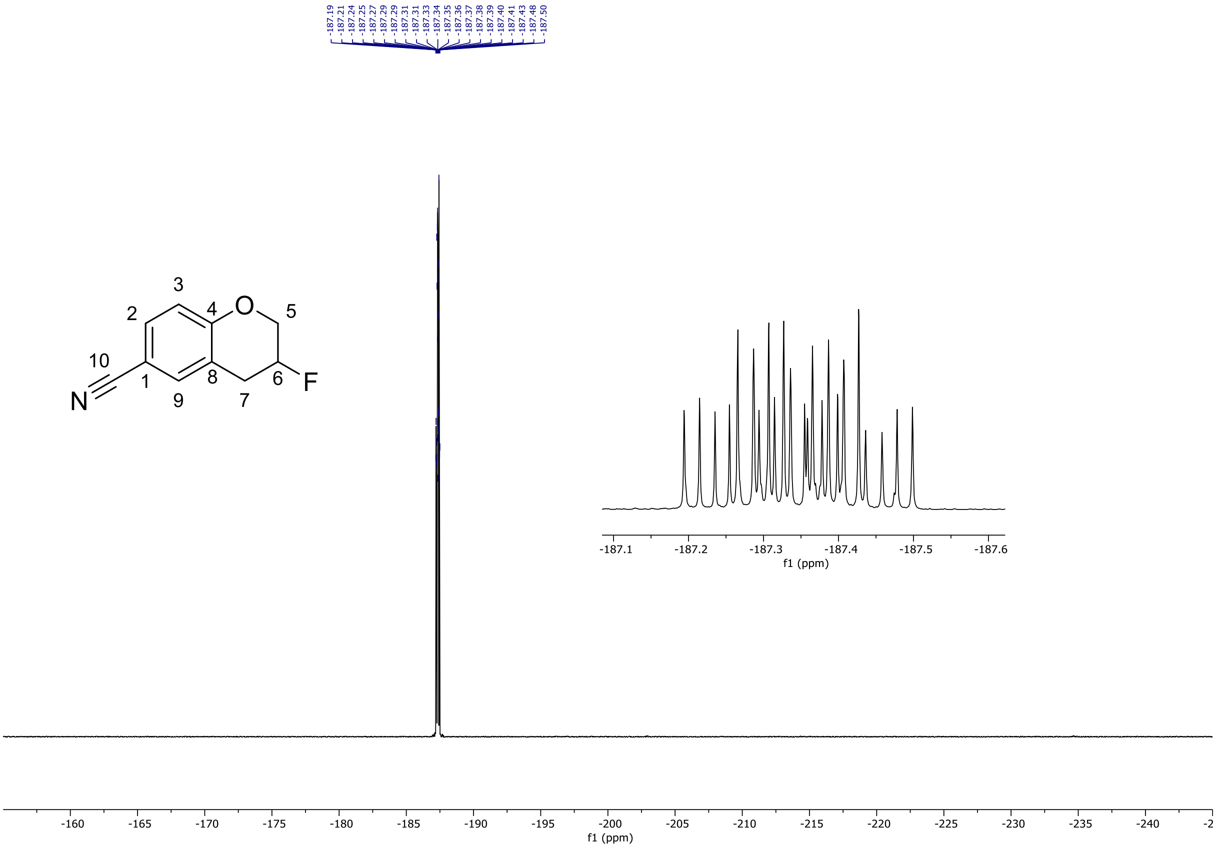

3-Fluorochromane-6-carbaldehyde (19)

^{1}H NMR (400 MHz, $CDCl_3$):

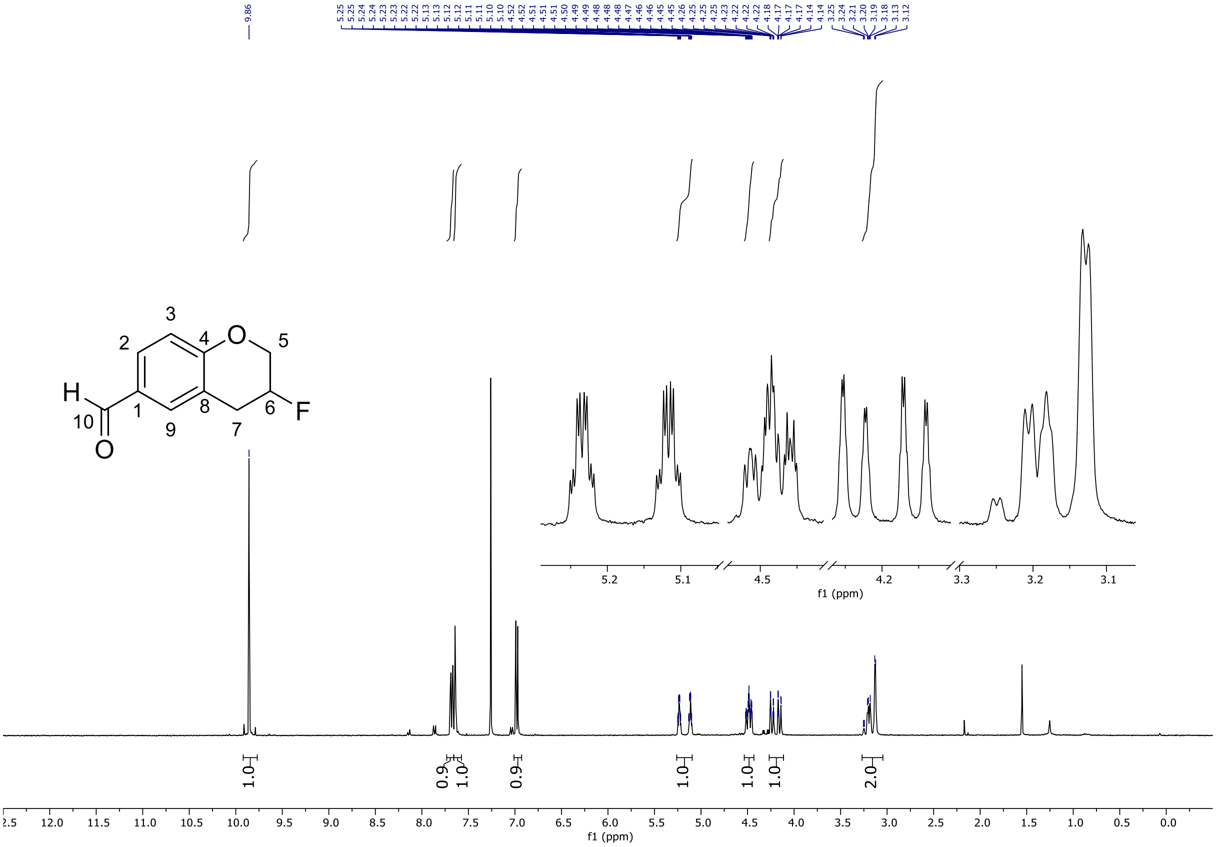

^{13}C{^{1}H} NMR (126 MHz, $CDCl_3$):

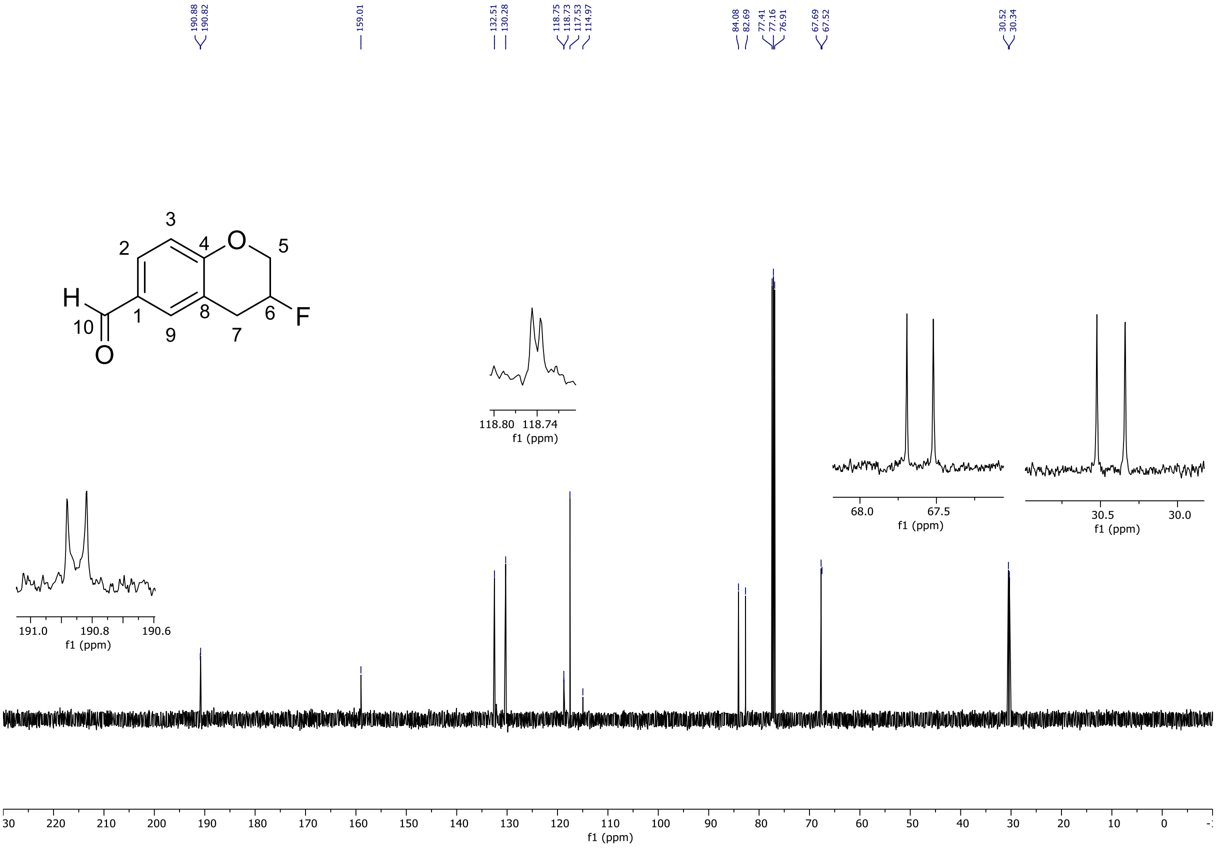

^{19}F NMR{^{1}H} (470 MHz, $CDCl_3$):

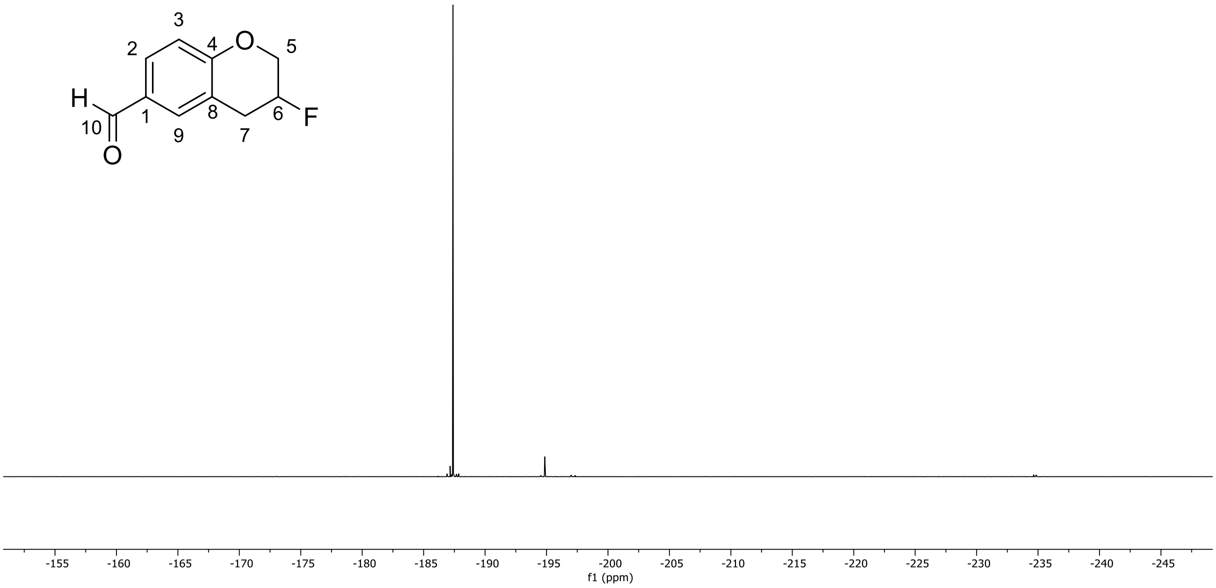

^{19}F NMR (470 MHz, $CDCl_3$):

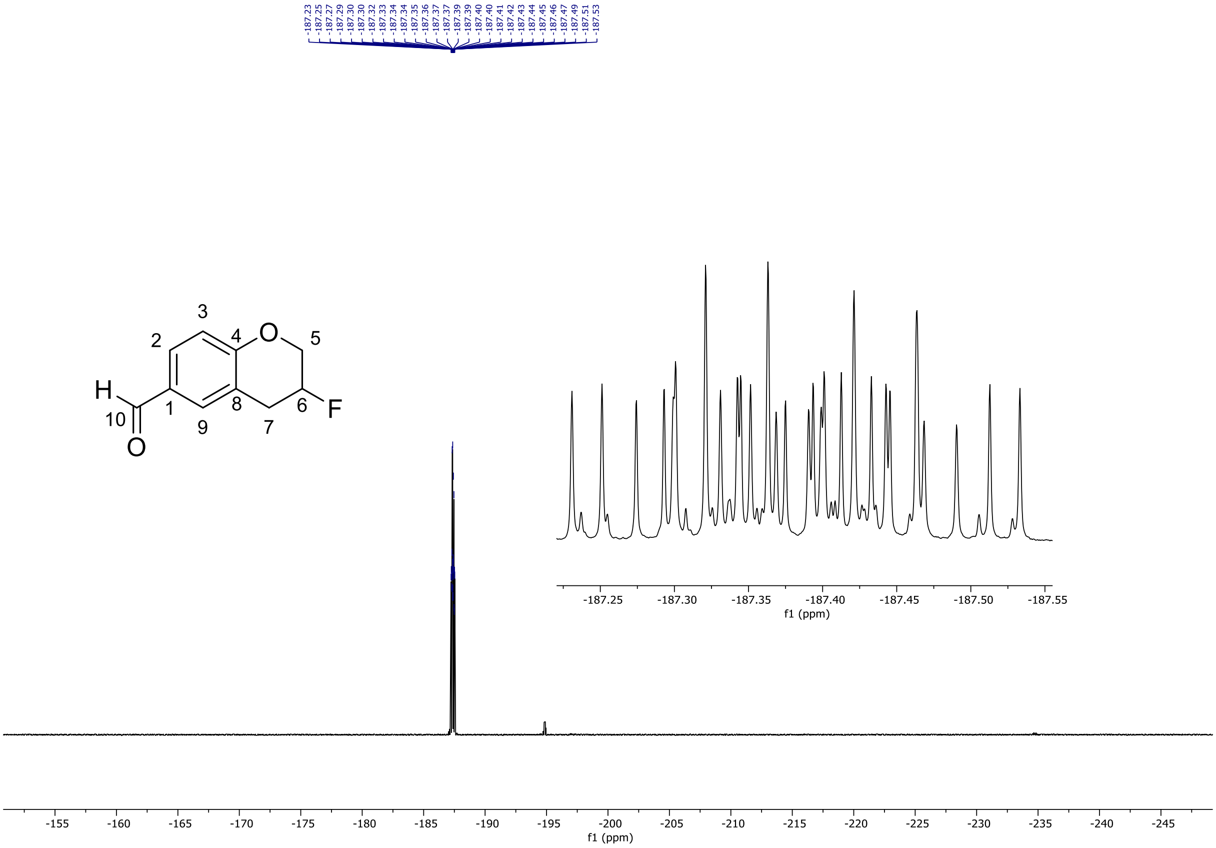

3-Fluoro-6-(methylsulfonyl)chromane (18)

^{1}H NMR (599 MHz, $CDCl_3$) :

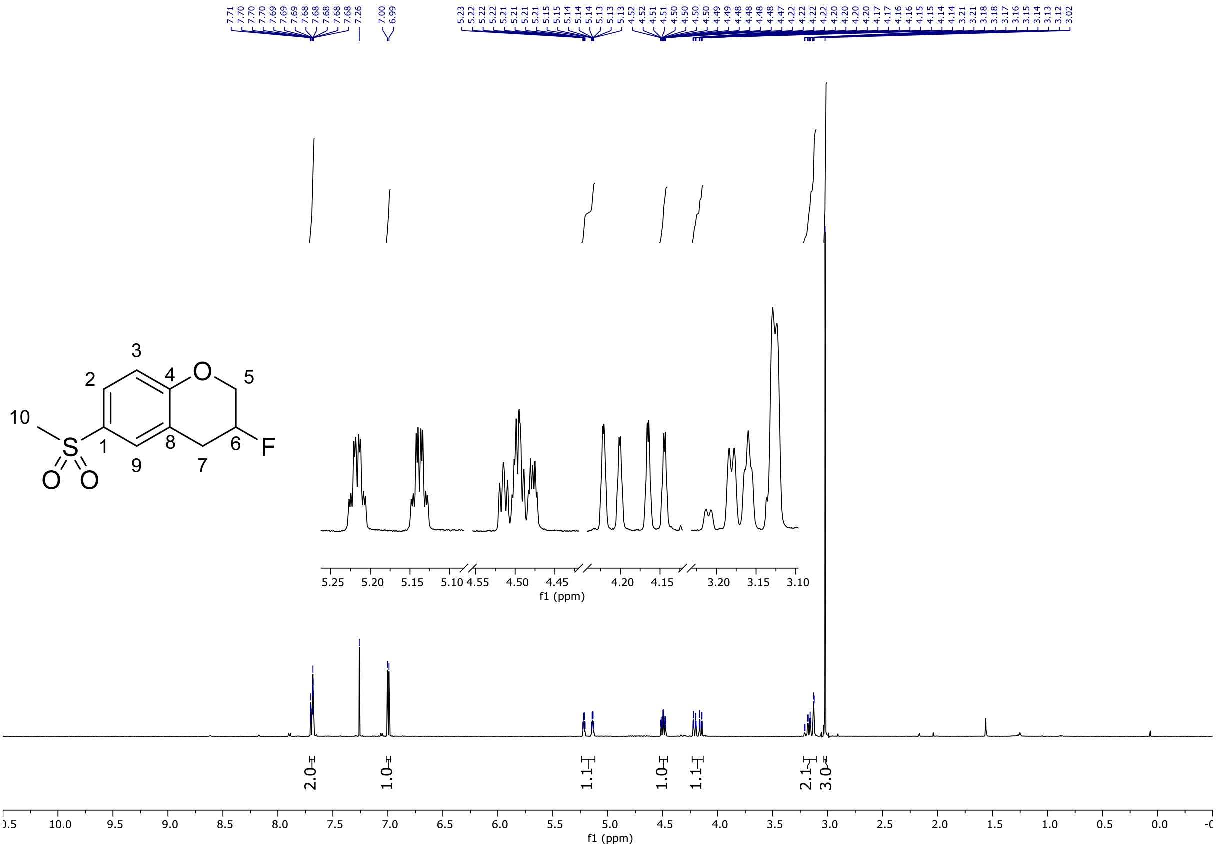

^{13}C{^{1}H} NMR (151 MHz, $CDCl_3$):

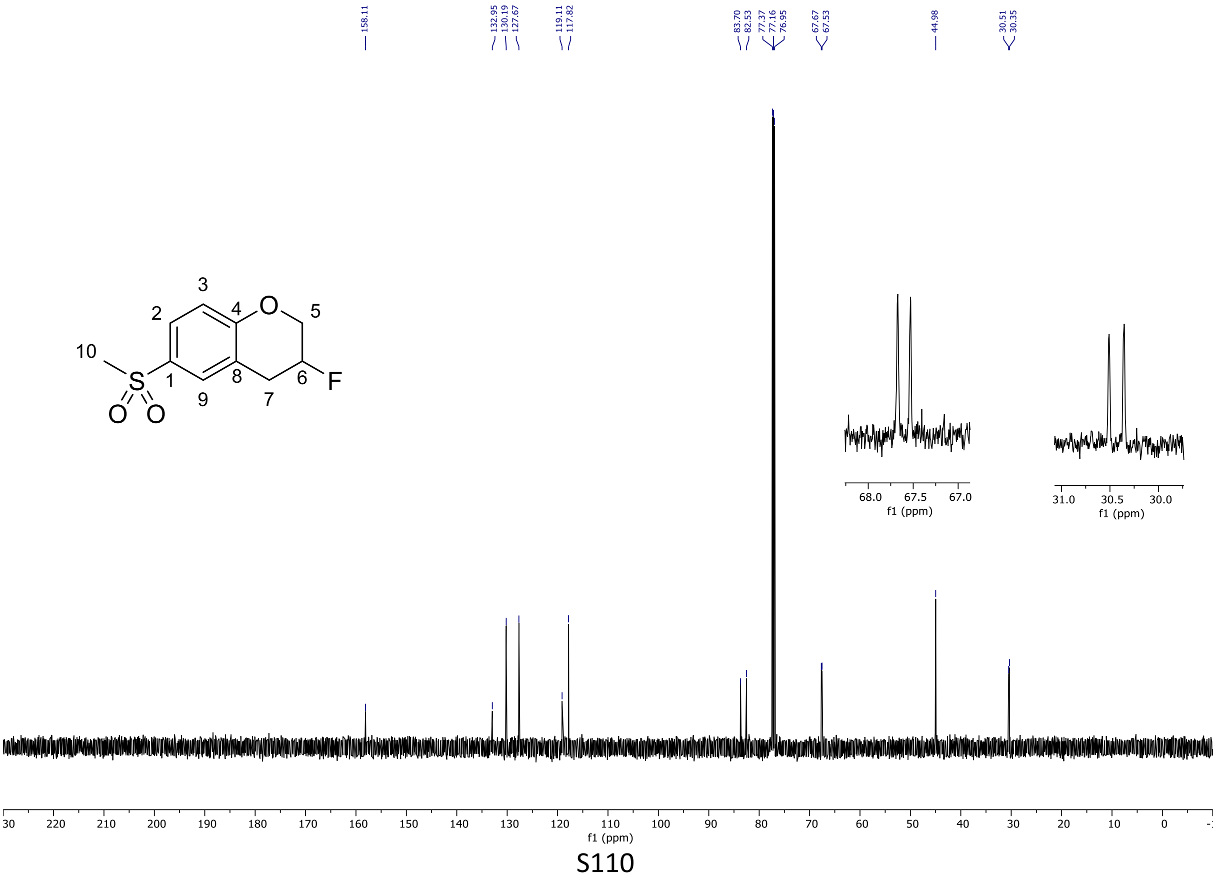

$^{19}F\{^{1}H\}$ NMR (564 MHz, $CDCl_3$):

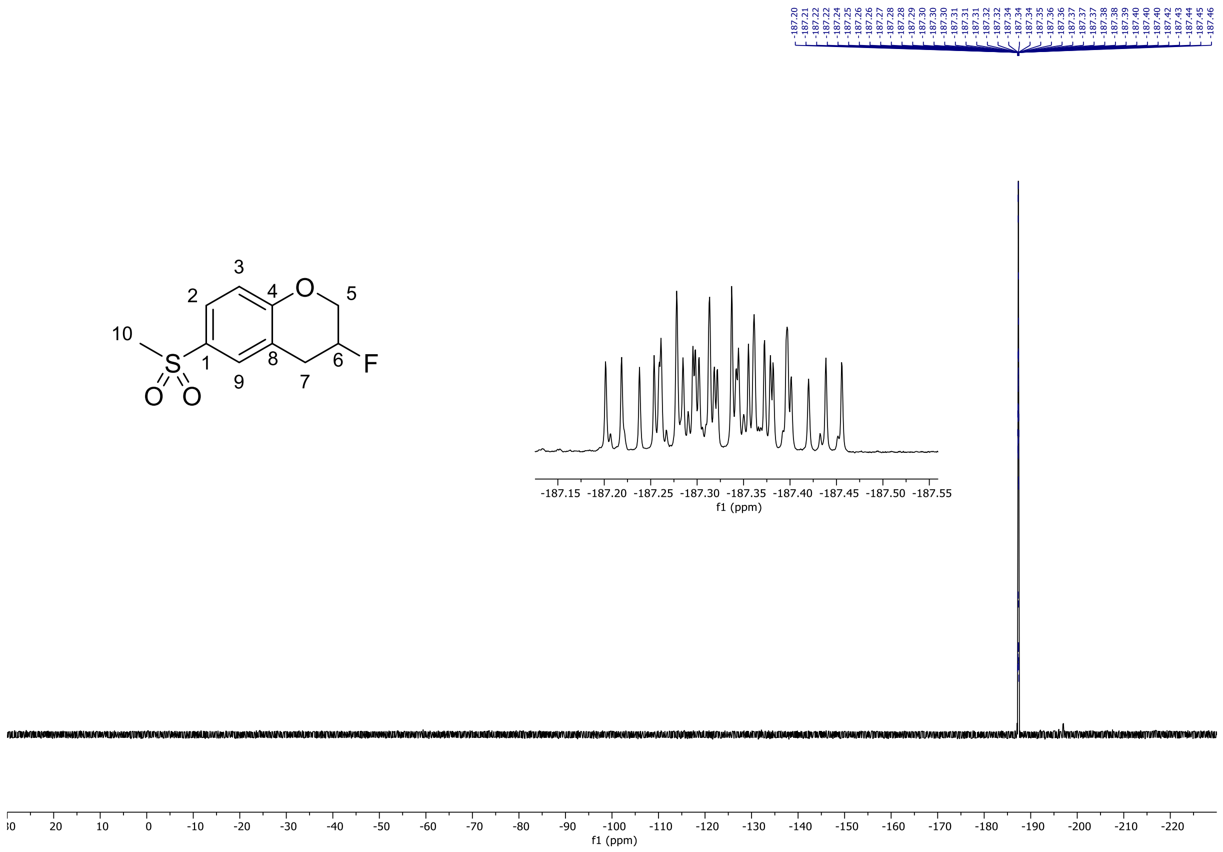

^{19}F NMR (564 MHz, $CDCl_3$):

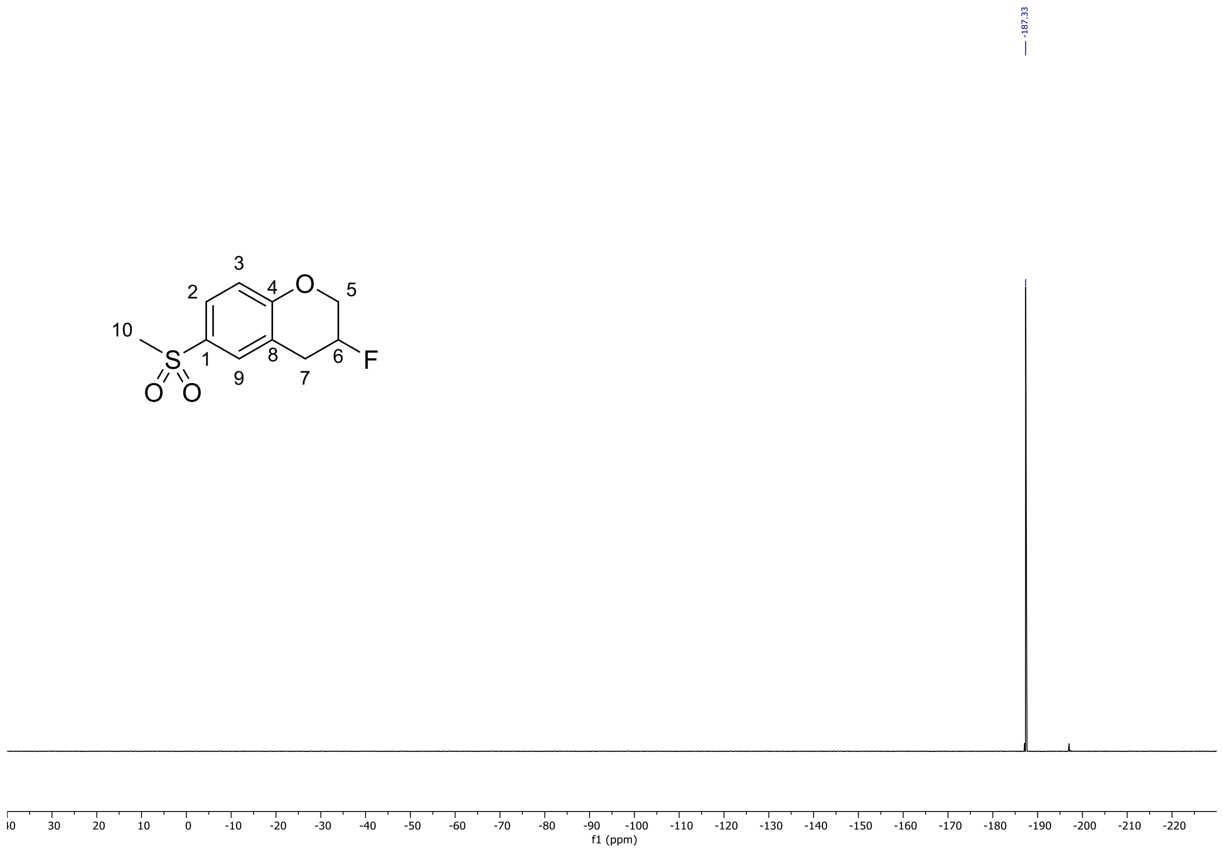

6-Nitro-3-fluorochromane (20)

^{1}H NMR (500 MHz, $CDCl_3$):

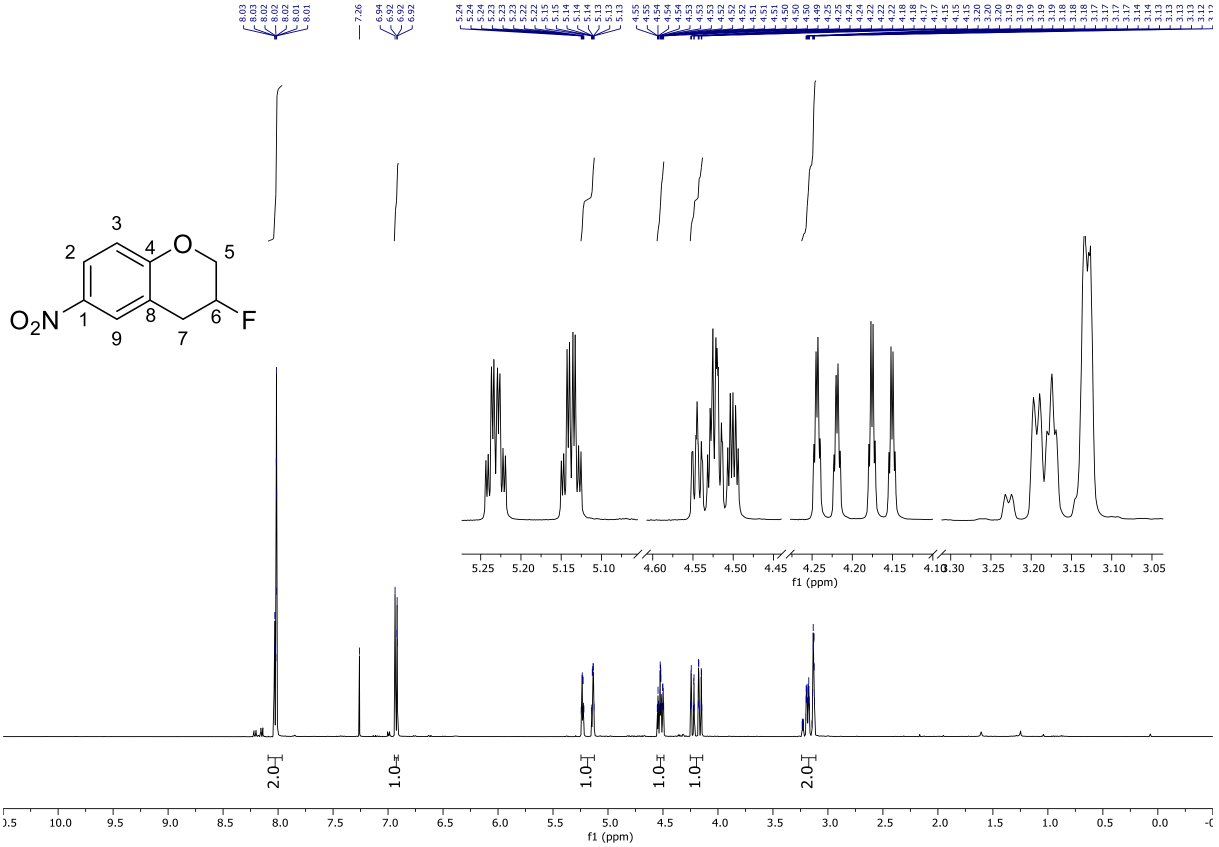

^{13}C{^{1}H} NMR (126 MHz, $CDCl_3$):

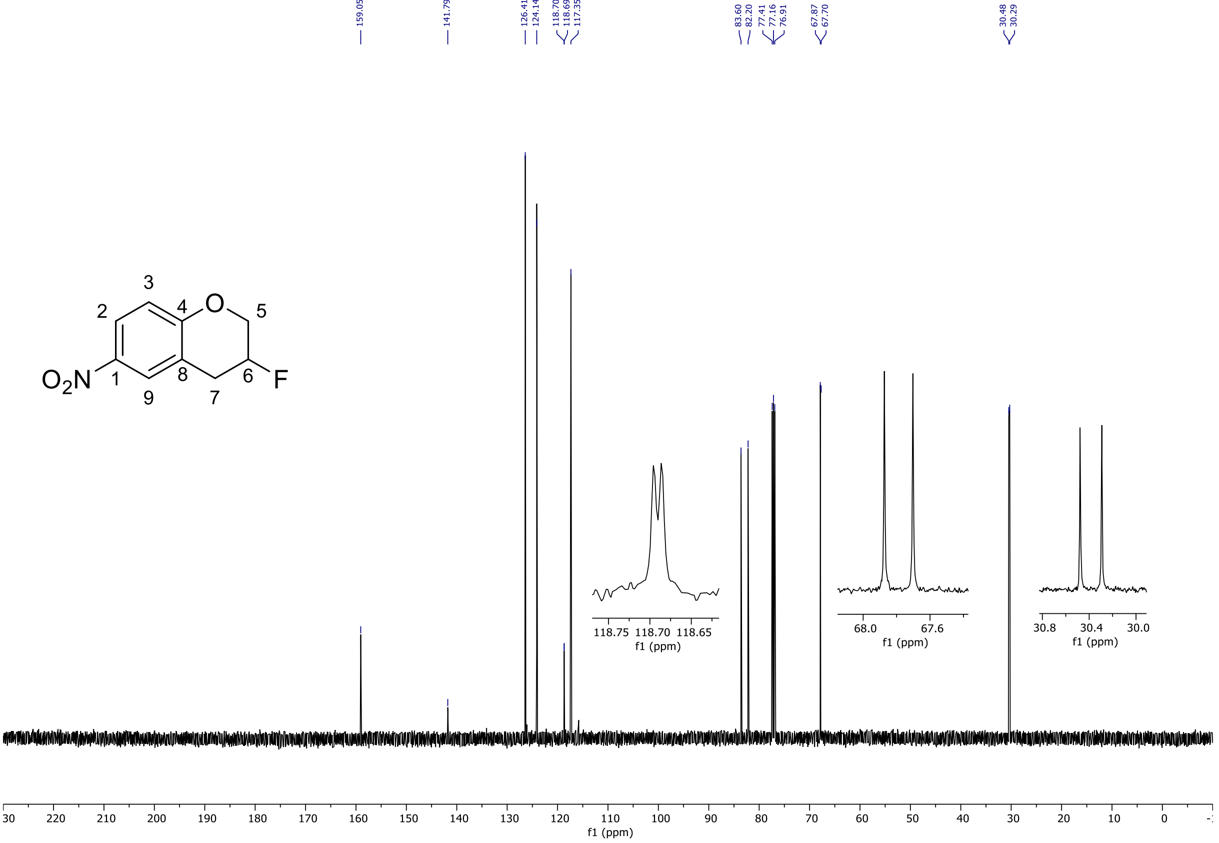

$^{19}F\{^{1}H\}$ NMR (564 MHz, $CDCl_3$):

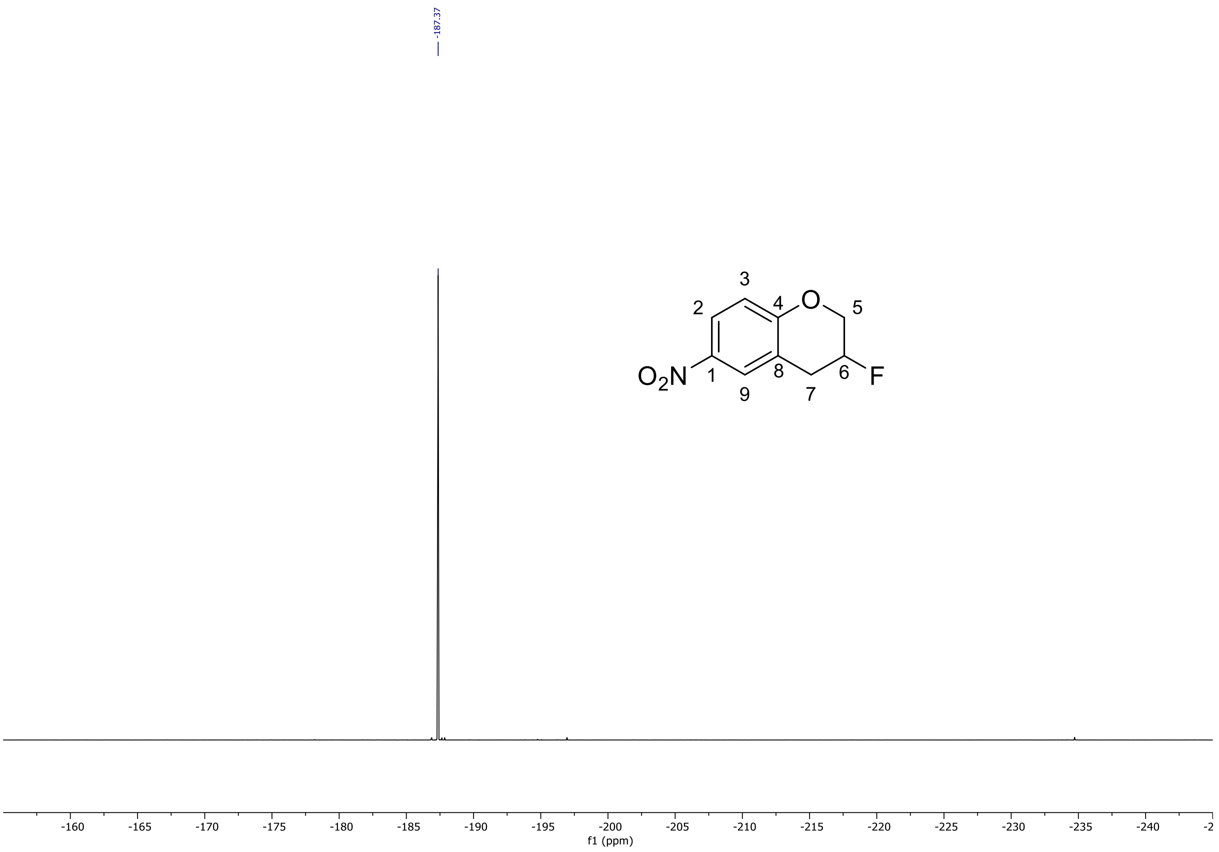

^{19}F NMR (564 MHz, $CDCl_3$):

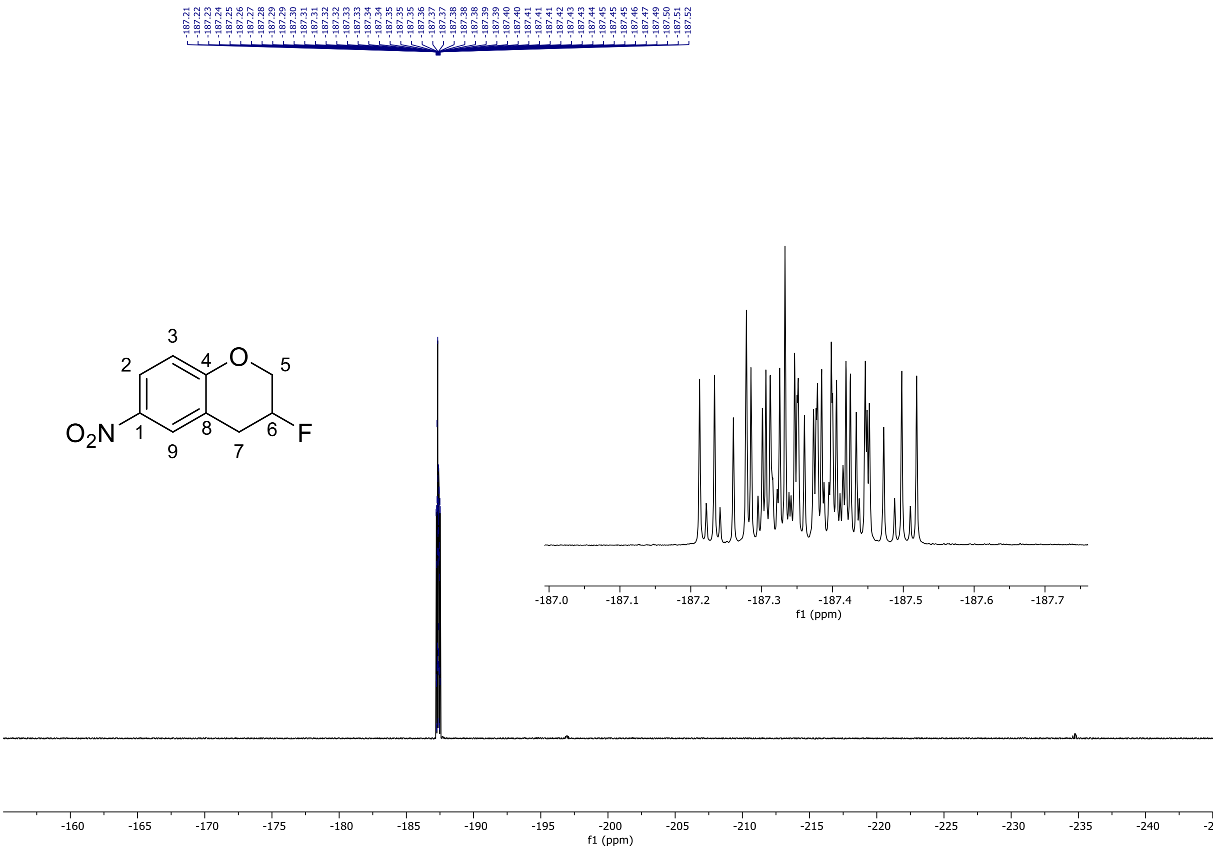

6-Bromo-3-fluoro-3-methylchromane (21)

^{1}H NMR (599 MHz, $CDCl_3$):

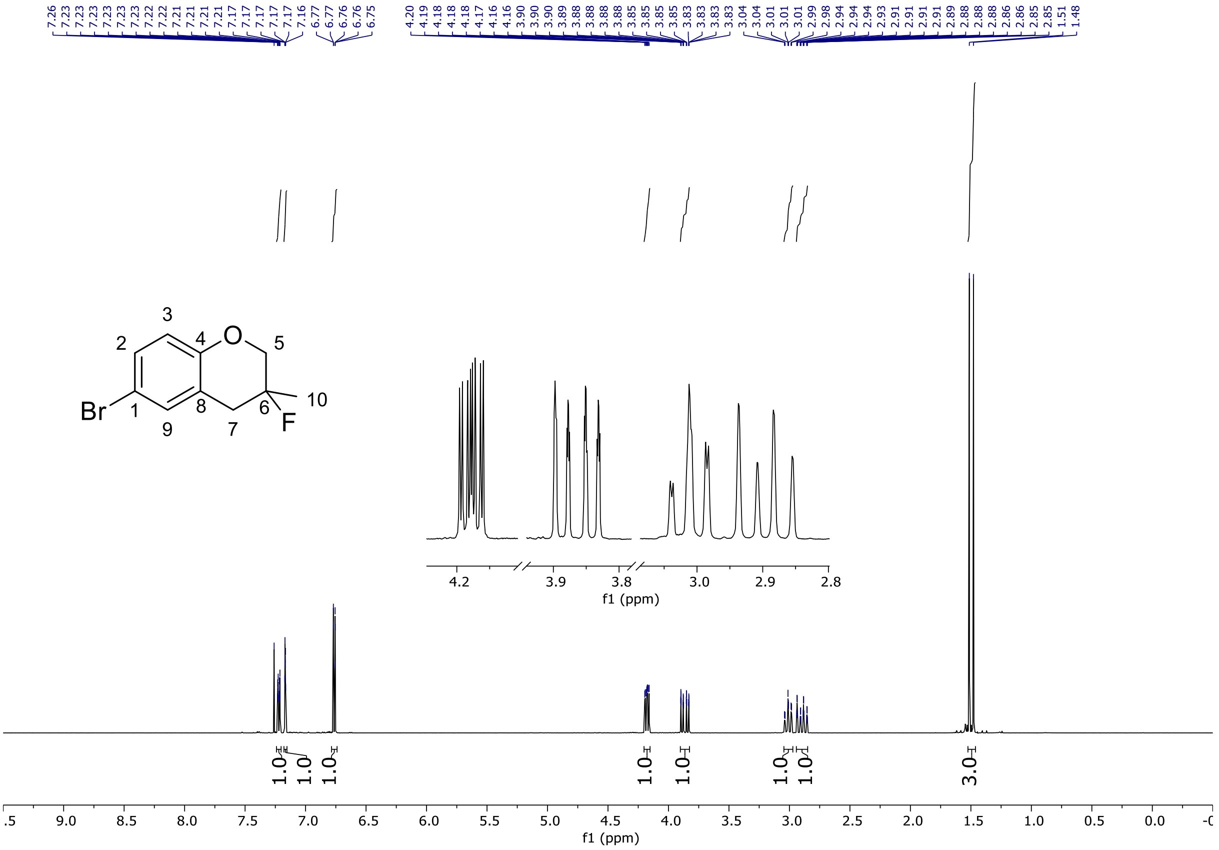

^{13}C{^{1}H} NMR (151 MHz, $CDCl_3$):

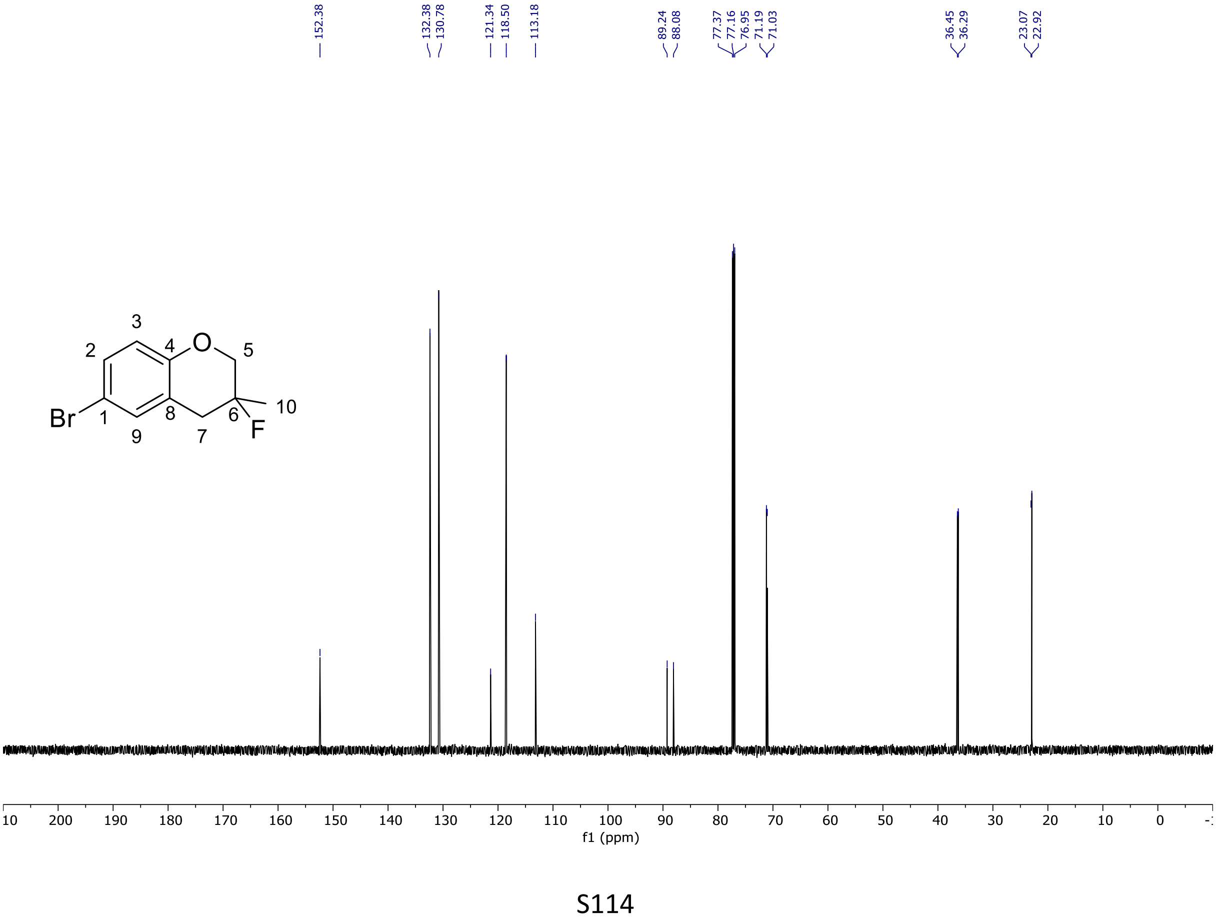

$^{19}F\{^{1}H\}$ NMR (564 MHz, $CDCl_3$):

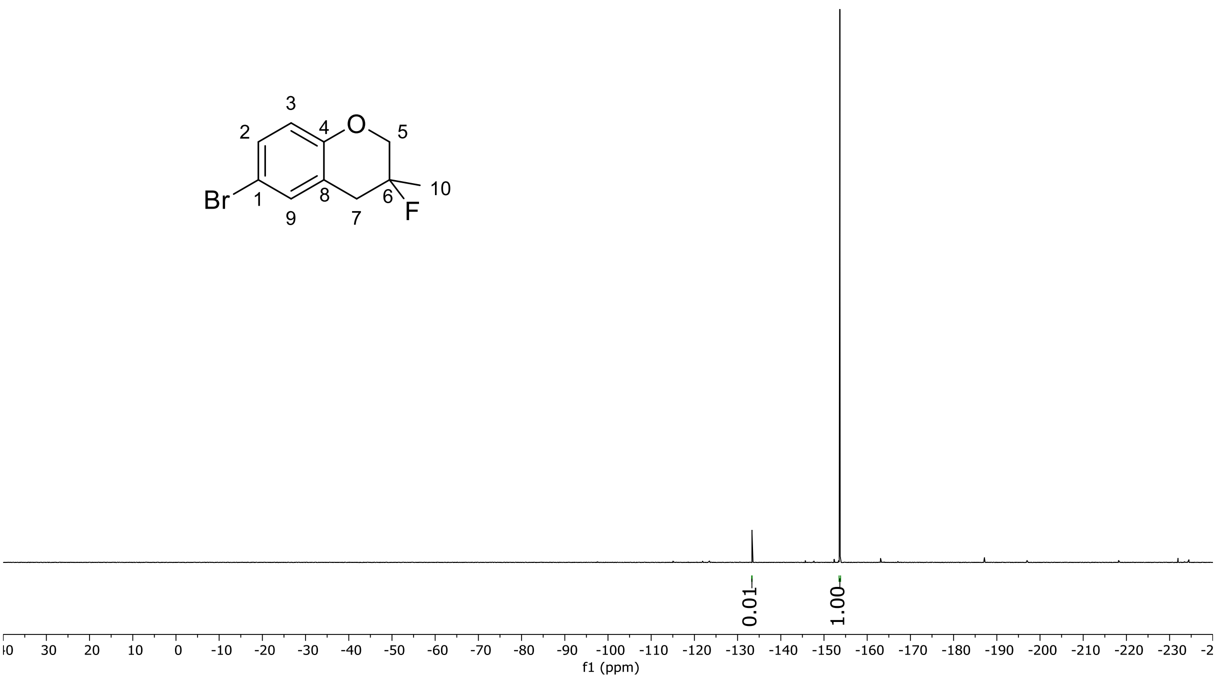

^{19}F NMR (564 MHz, $CDCl_3$):

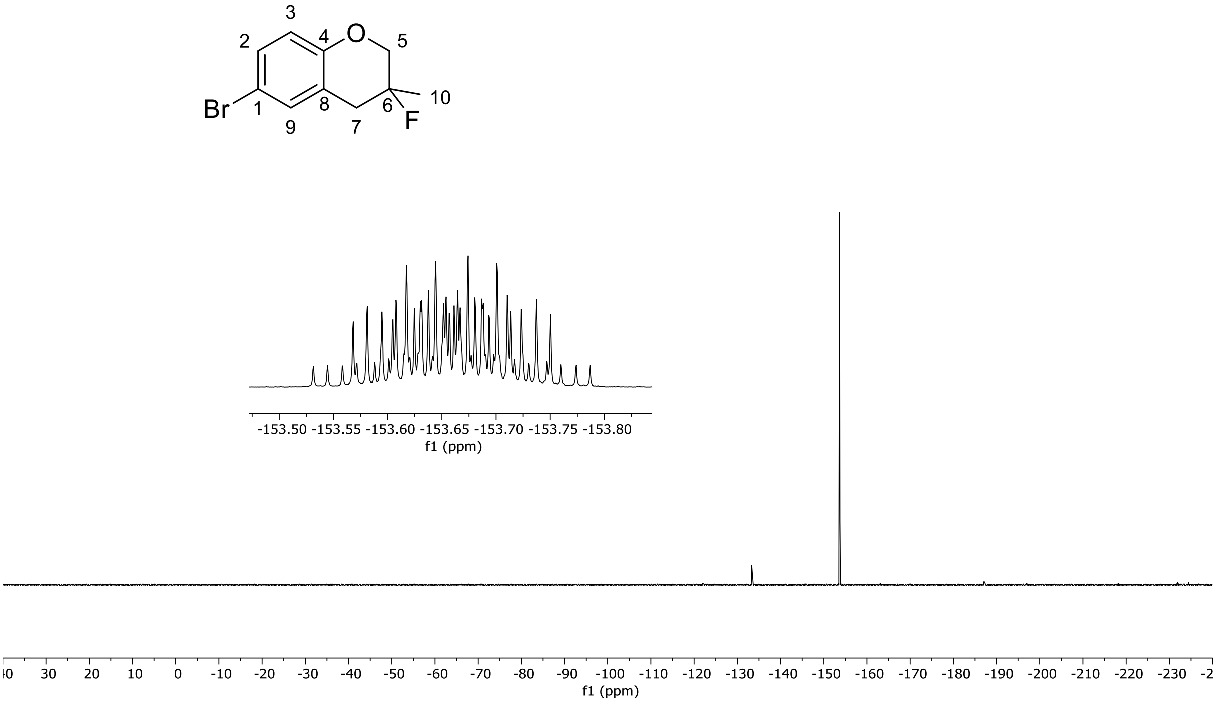

3-Fluorochroman-6-yl methanesulfonate (13)

^{1}H NMR (599 MHz, $CDCl_3$):

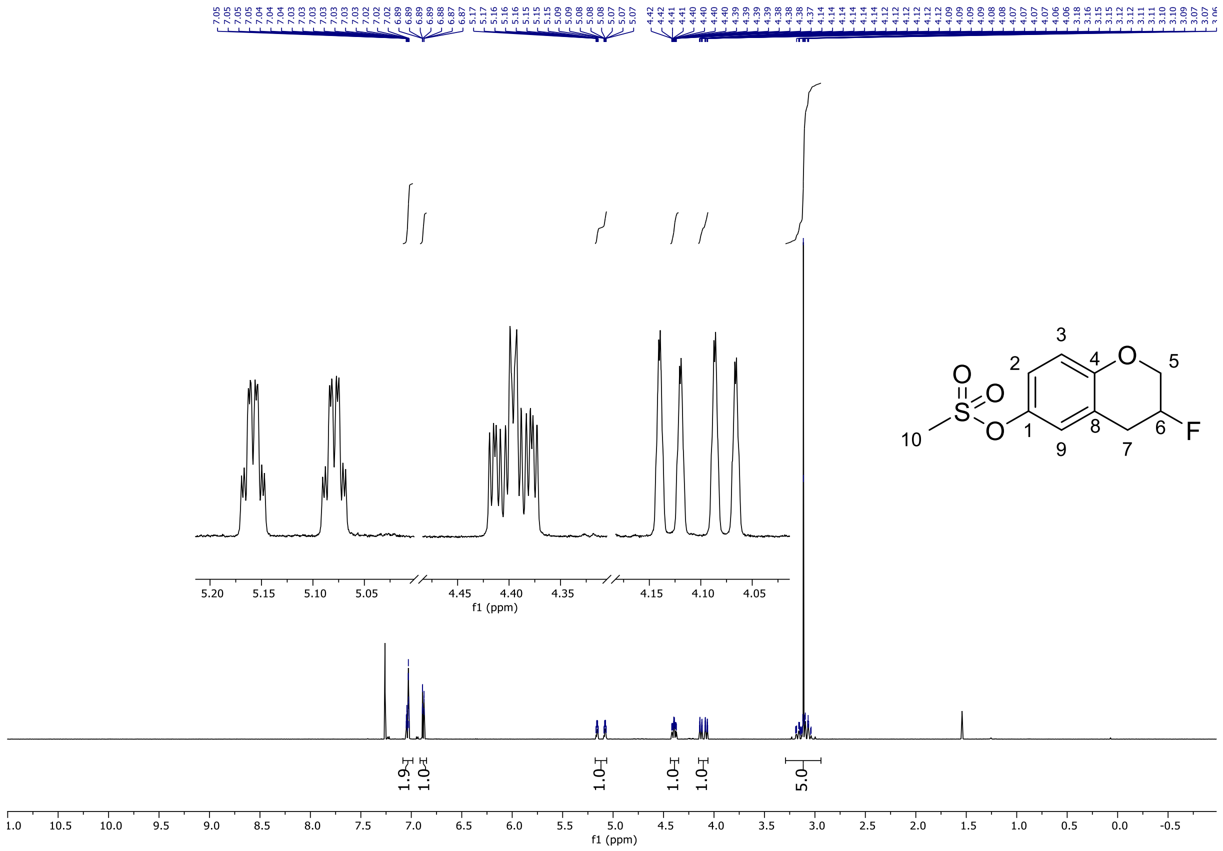

^{13}C{^{1}H} NMR (151 MHz, $CDCl_3$):

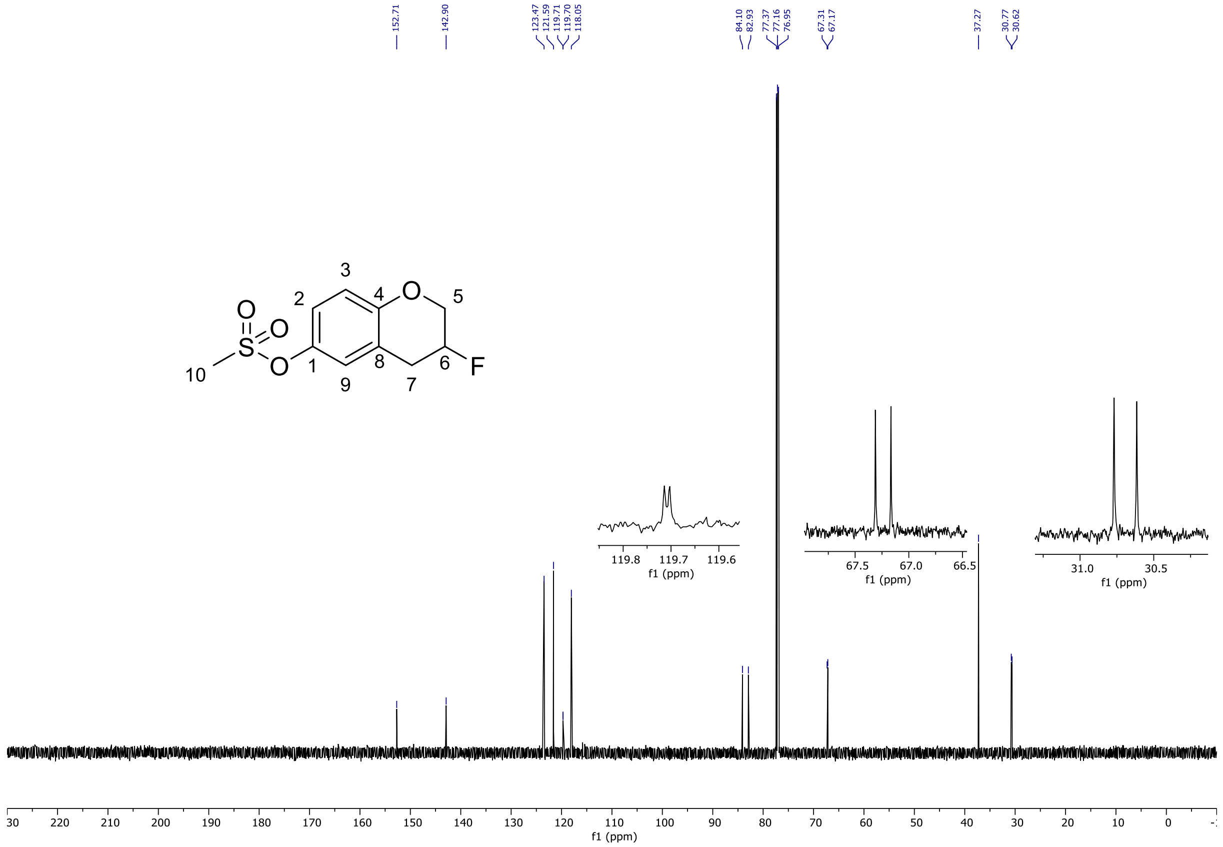

^{19}F{^{1}H} NMR (564 MHz, $CDCl_3$):

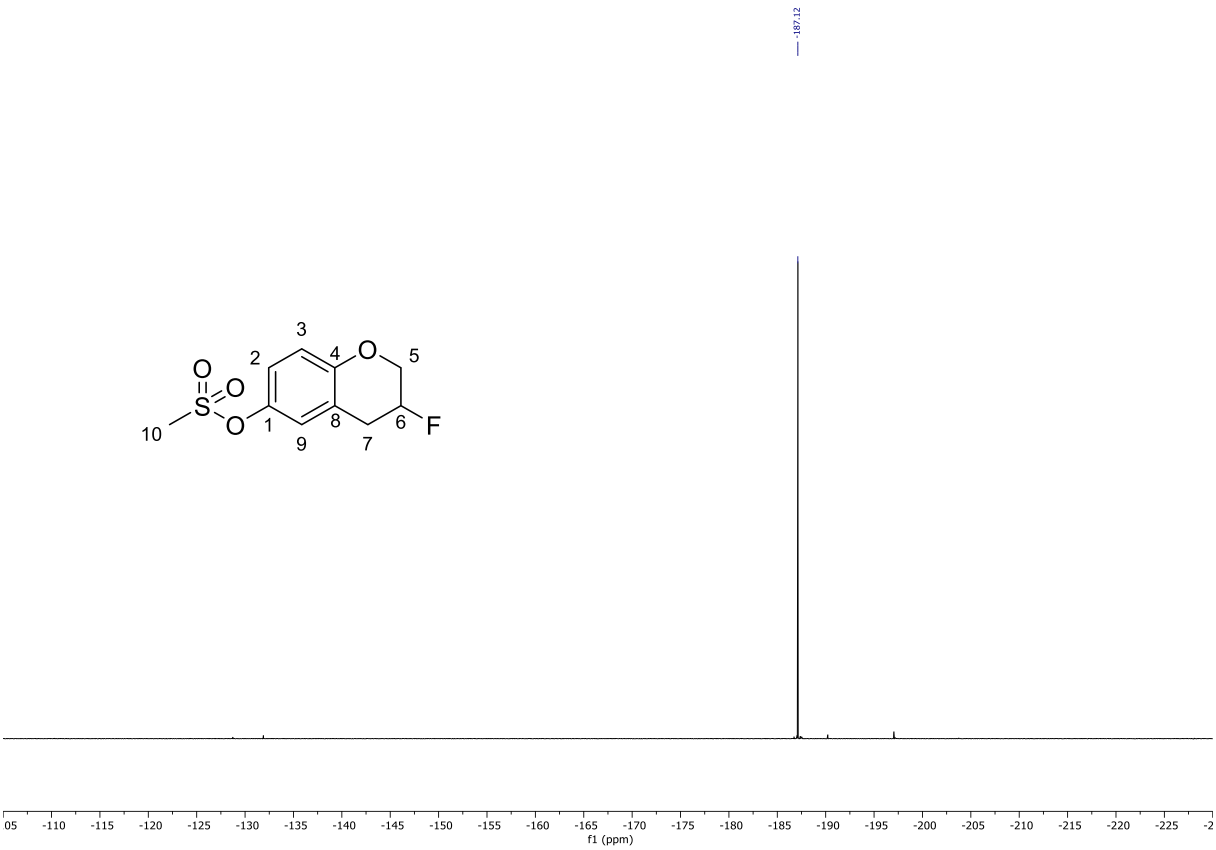

^{19}F NMR (564 MHz, $CDCl_3$):

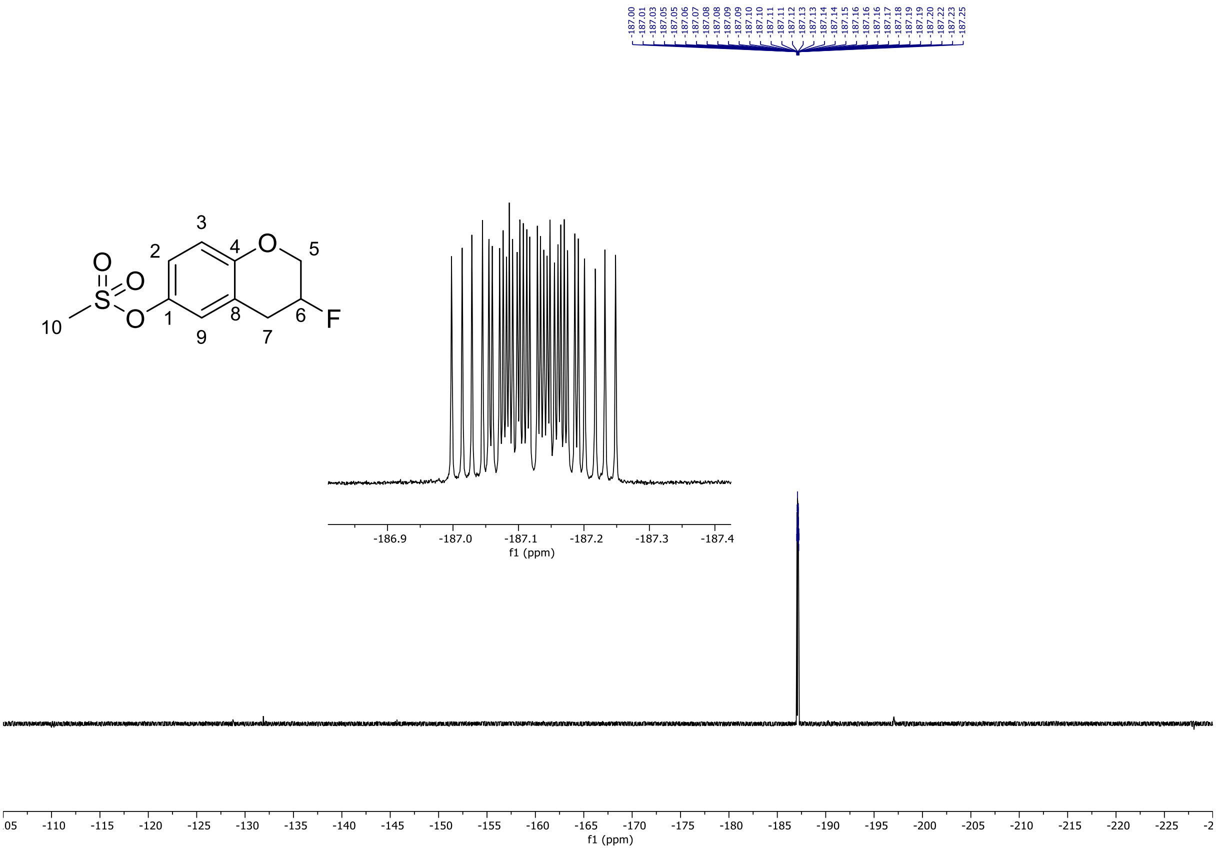

(3*R*,4*S*)-6-Bromo-3-fluorochromane-4-*d* (32)

^{1}H NMR (599 MHz, $CDCl_3$):

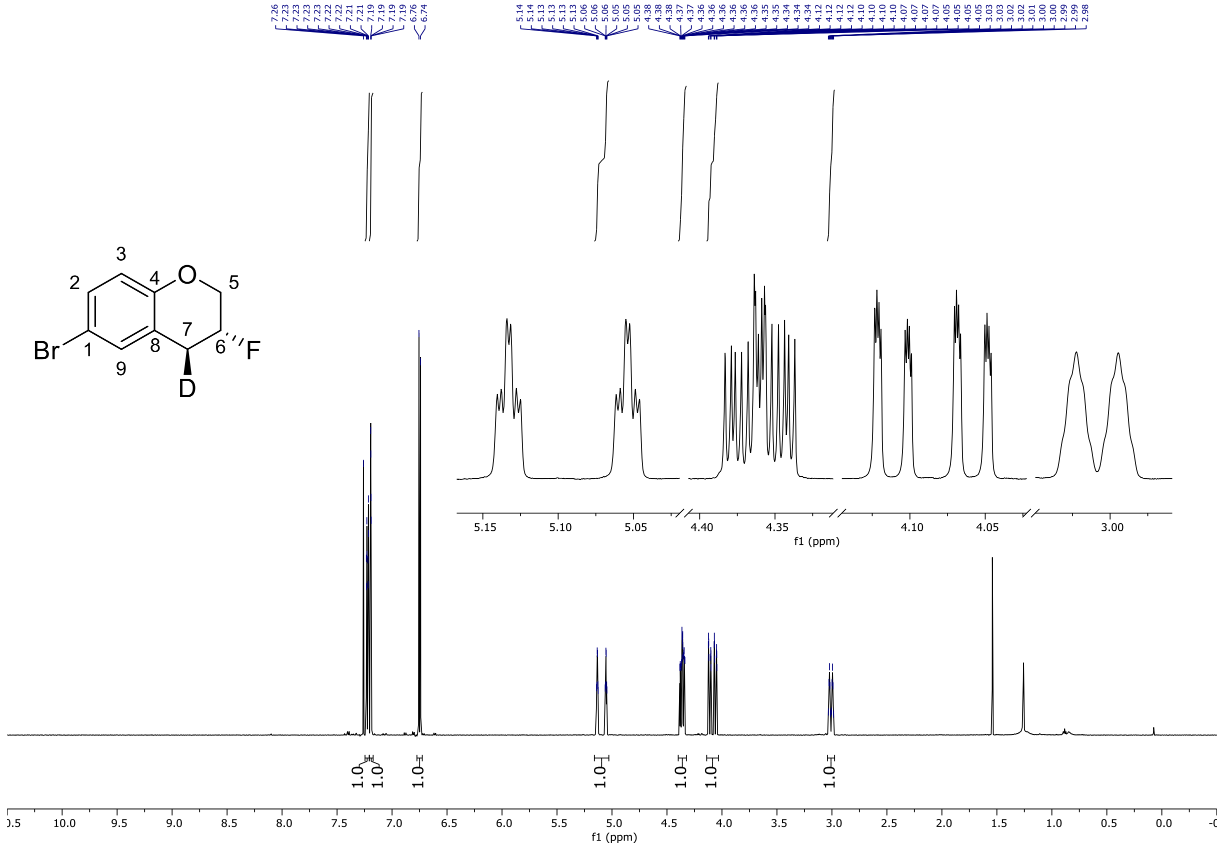

^{13}C{^{1}H} NMR (151 MHz, $CDCl_3$):

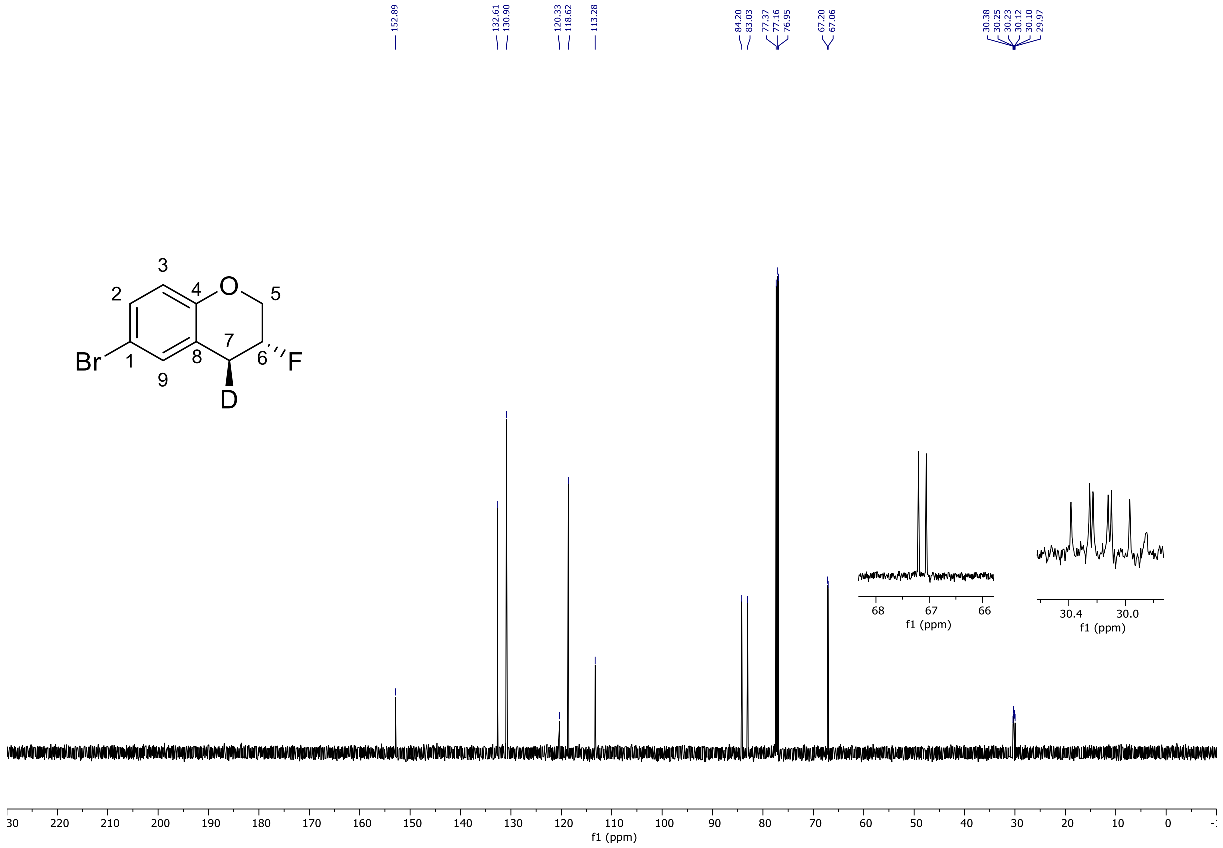

^{19}**F{^{1}H} NMR** (564 MHz, $CDCl_3$):

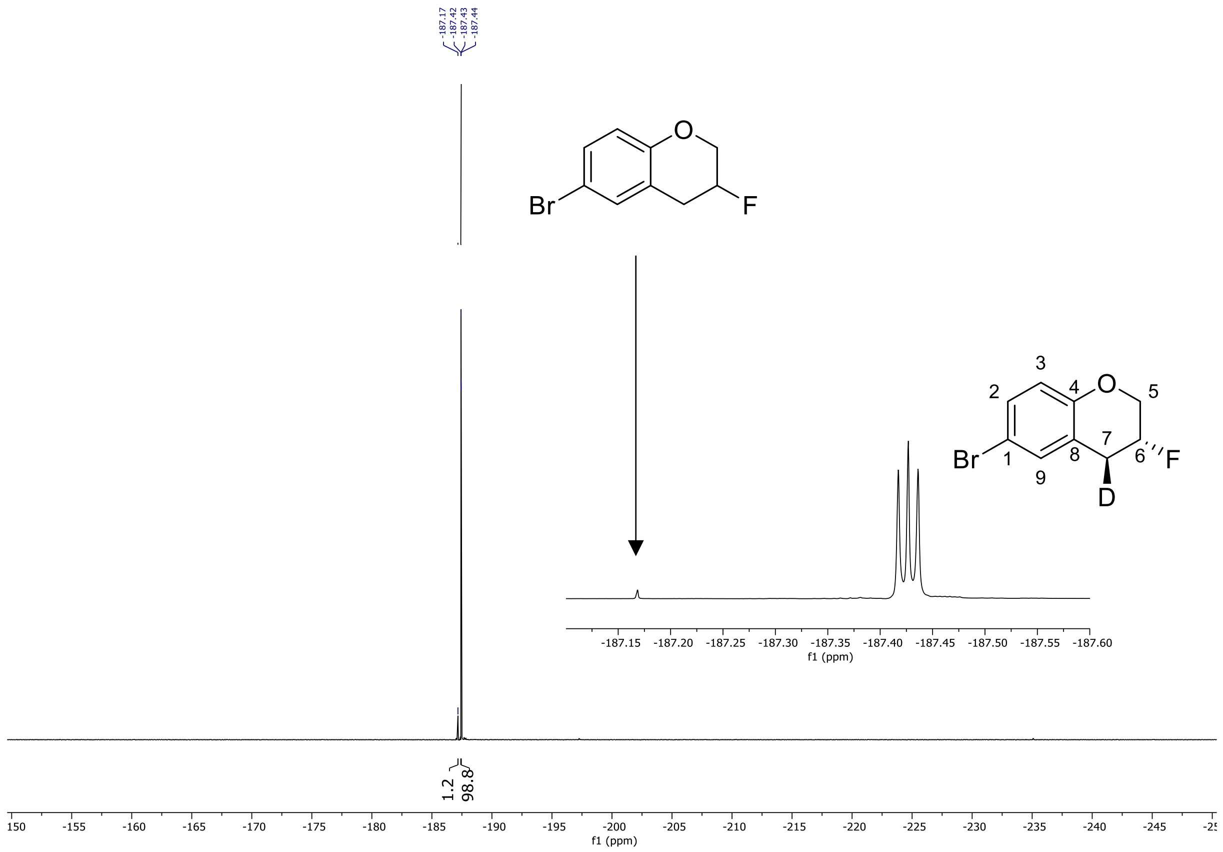

^{19}**F NMR** (564 MHz, $CDCl_3$):

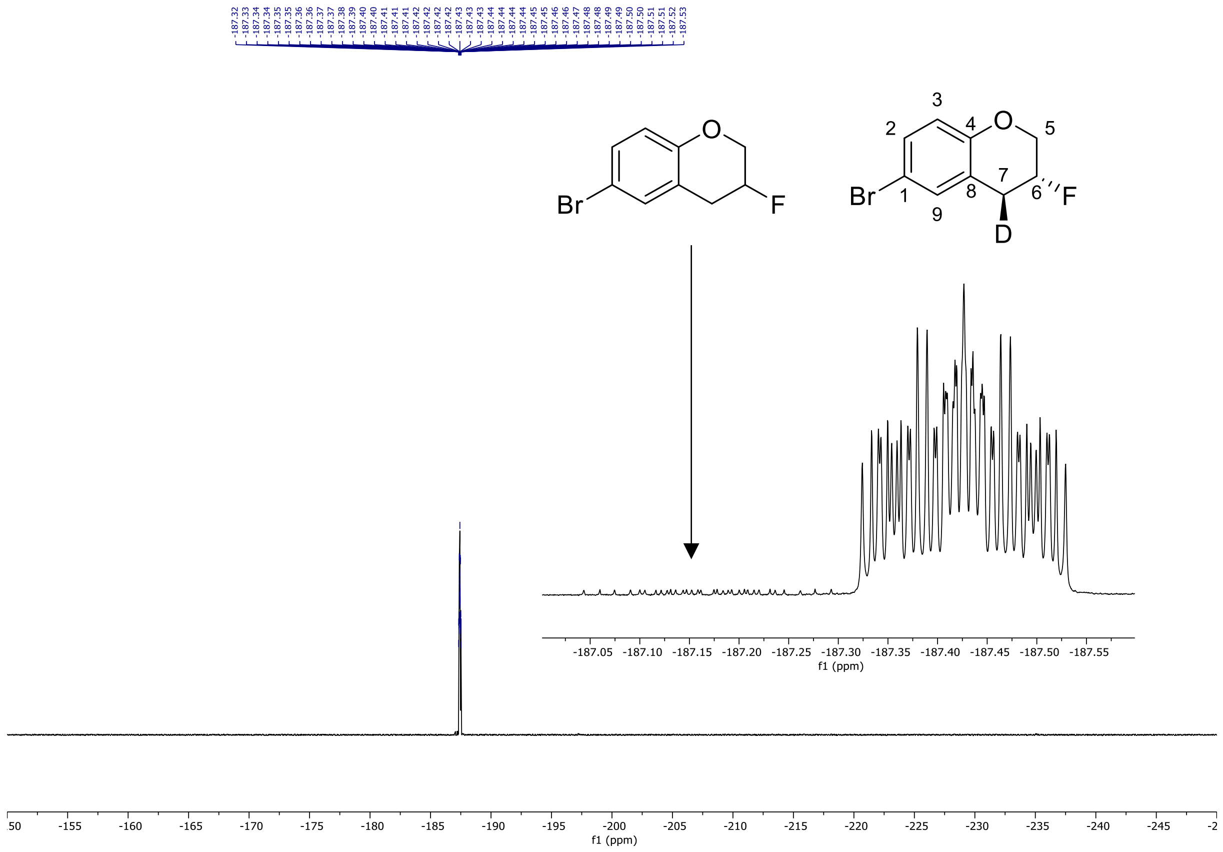

(3*S*,4*R*)-6-Bromo-3-fluorochromane-4-*d* (*ent*-32)

^{1}H NMR (599 MHz, $CDCl_3$):

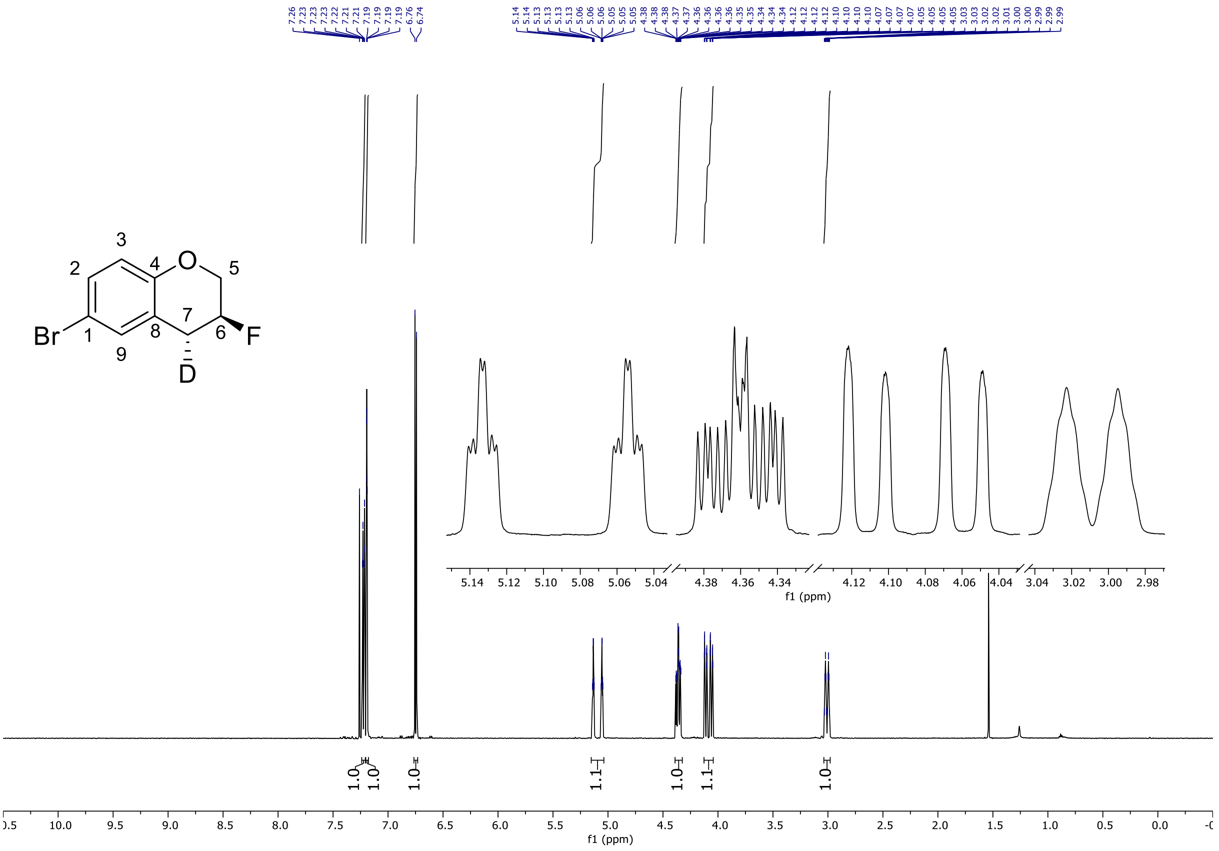

^{13}C{^{1}H} NMR (151 MHz, $CDCl_3$):

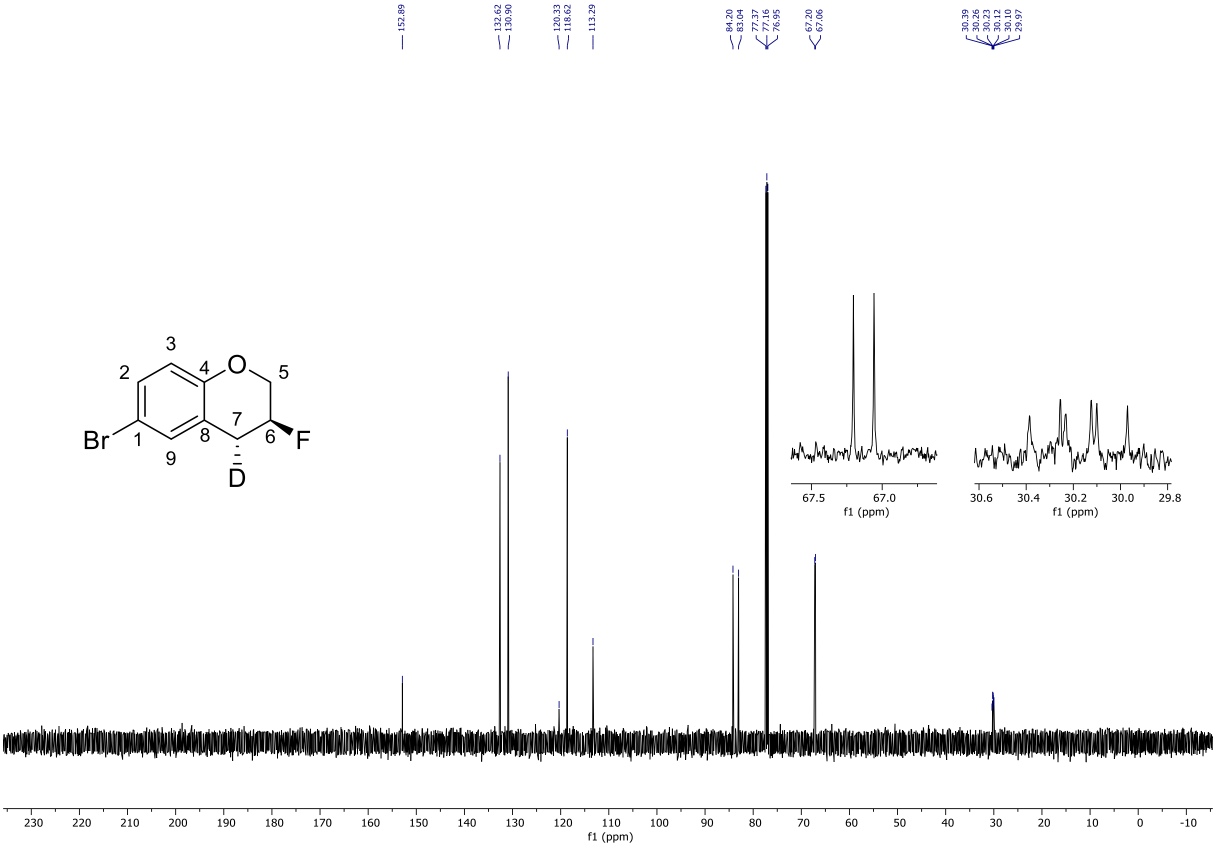

^{19}**F{^{1}H} NMR** (564 MHz, $CDCl_3$):

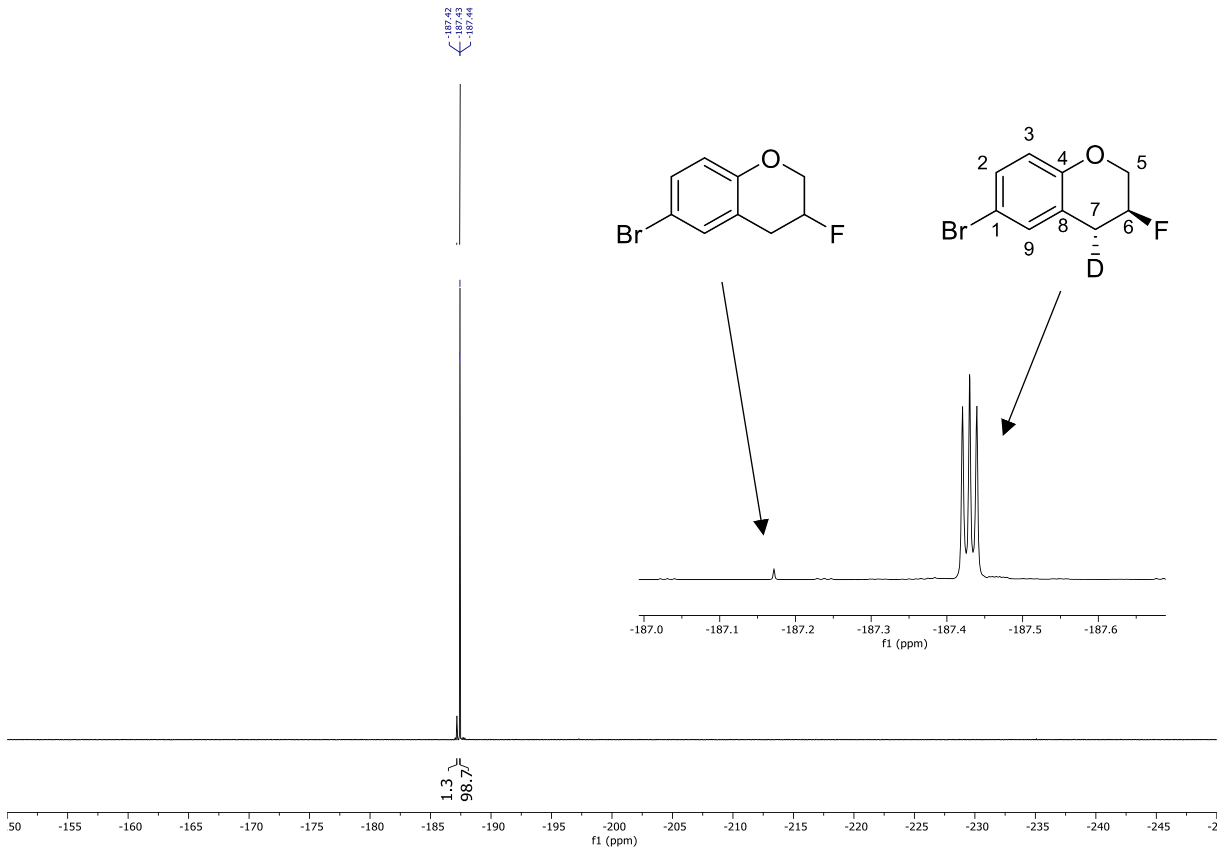

^{19}**F NMR** (564 MHz, $CDCl_3$):

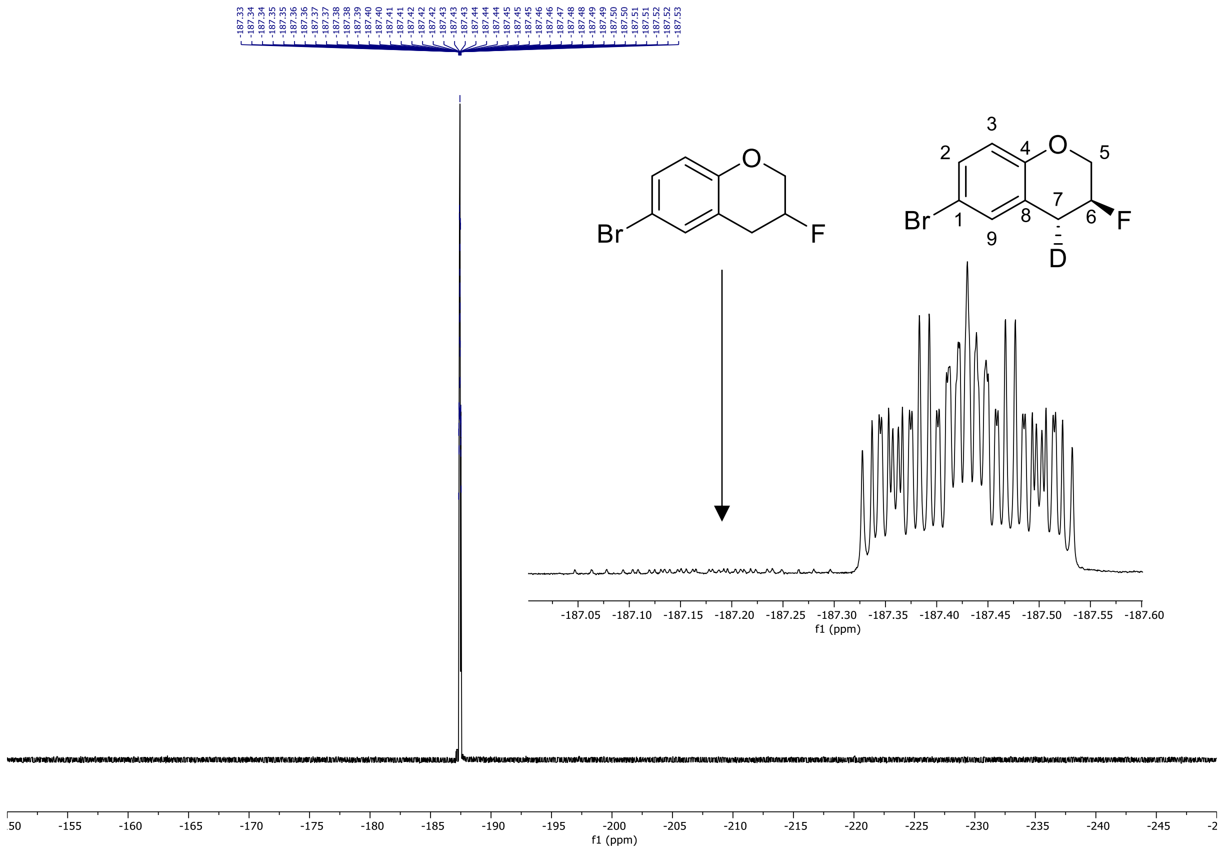

(3*R*,4*R*)-6-Bromo-3-fluorochromane-4-*d* (33)

^{1}H NMR (500 MHz, $CDCl_3$) :

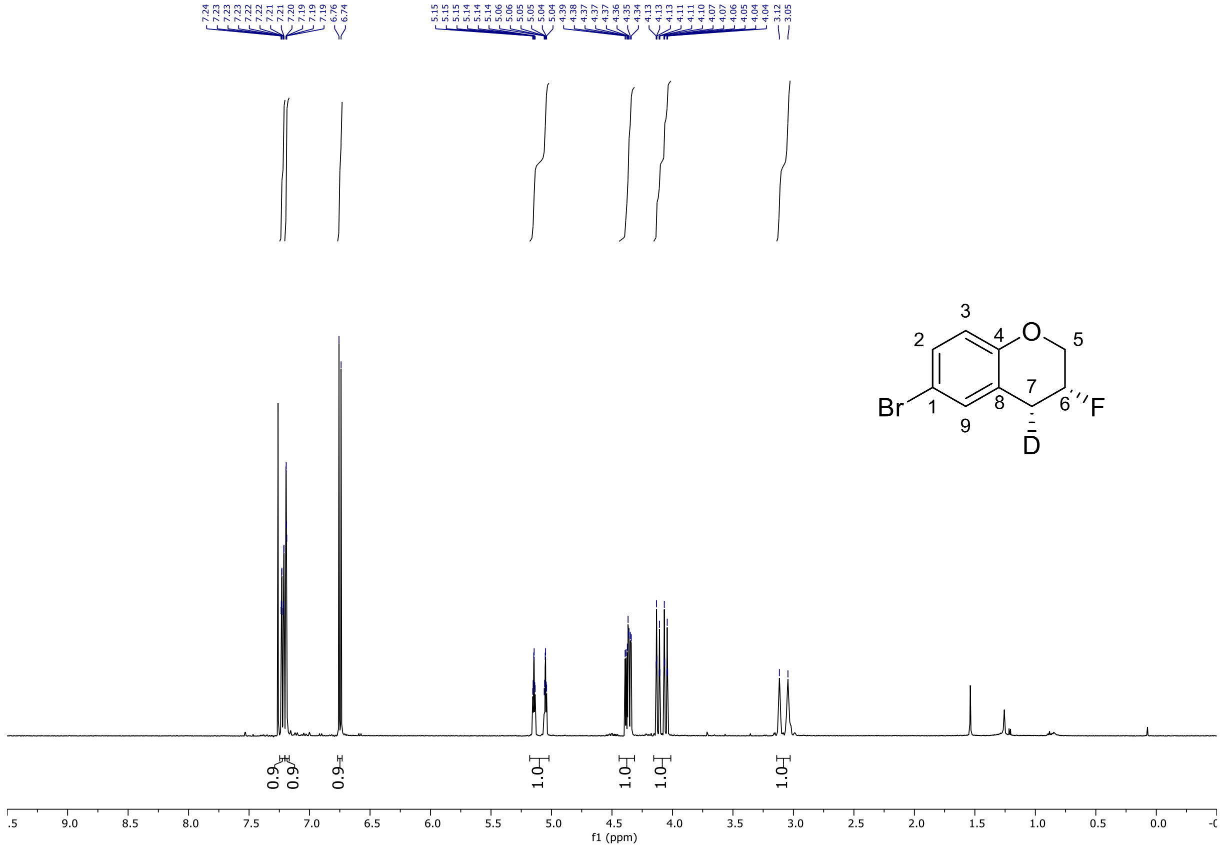

^{13}C{^{1}H} NMR (126 MHz, $CDCl_3$) :

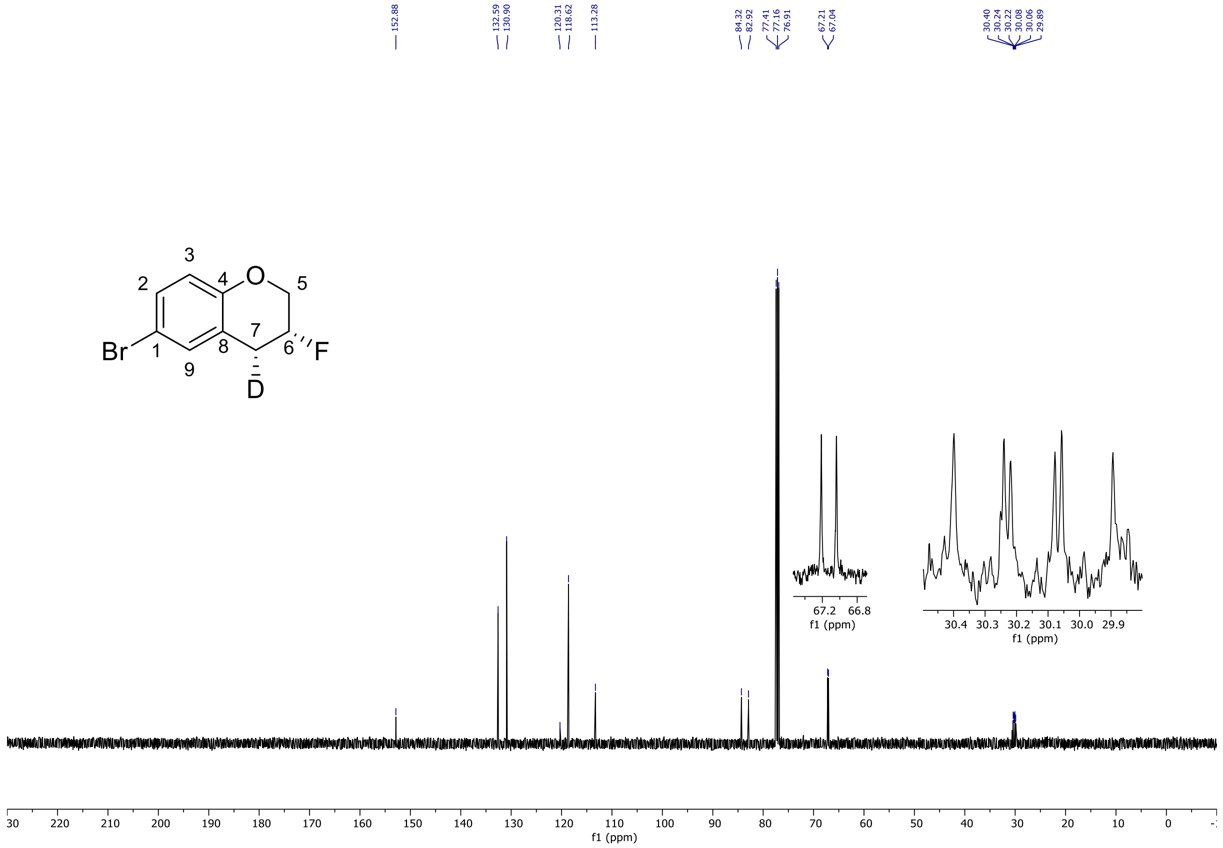

^{19}**F{^{1}H} NMR** (470 MHz, $CDCl_3$):

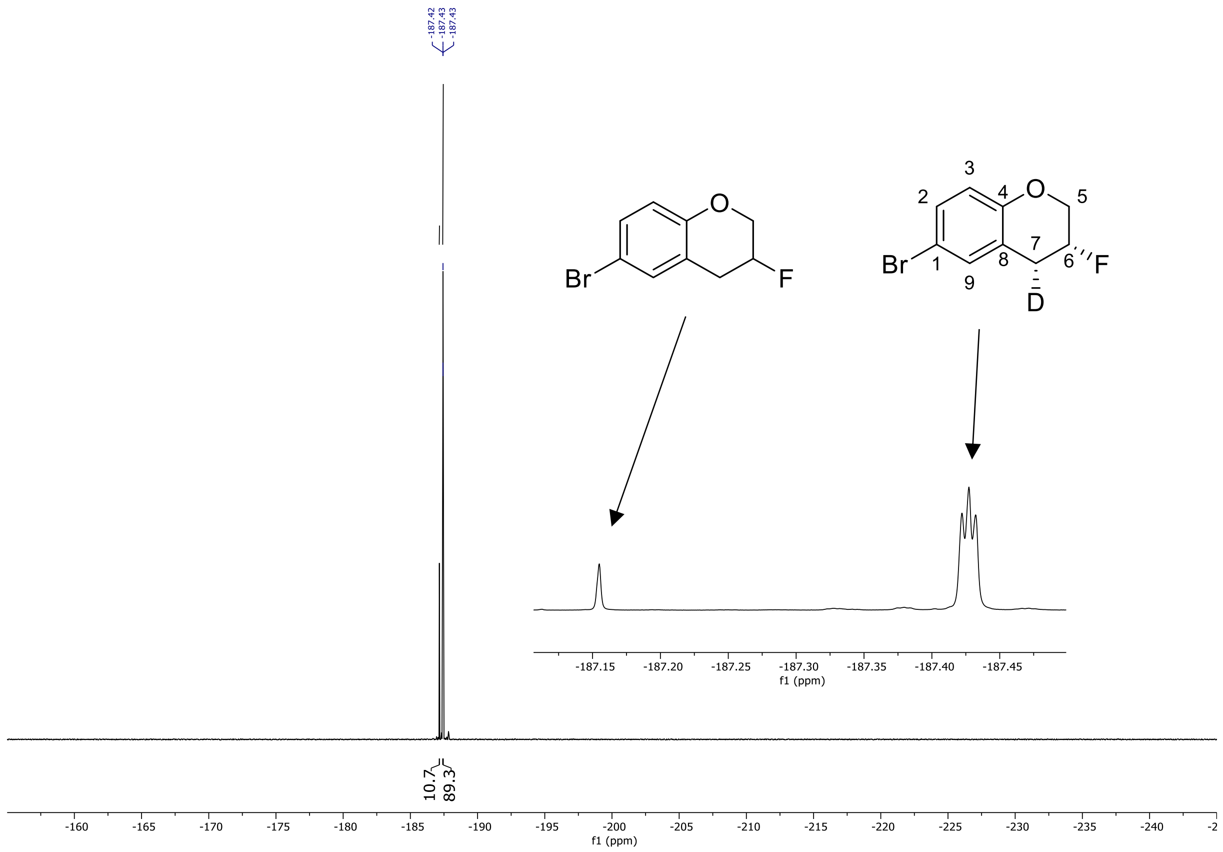

^{19}**F NMR** (470 MHz, $CDCl_3$):

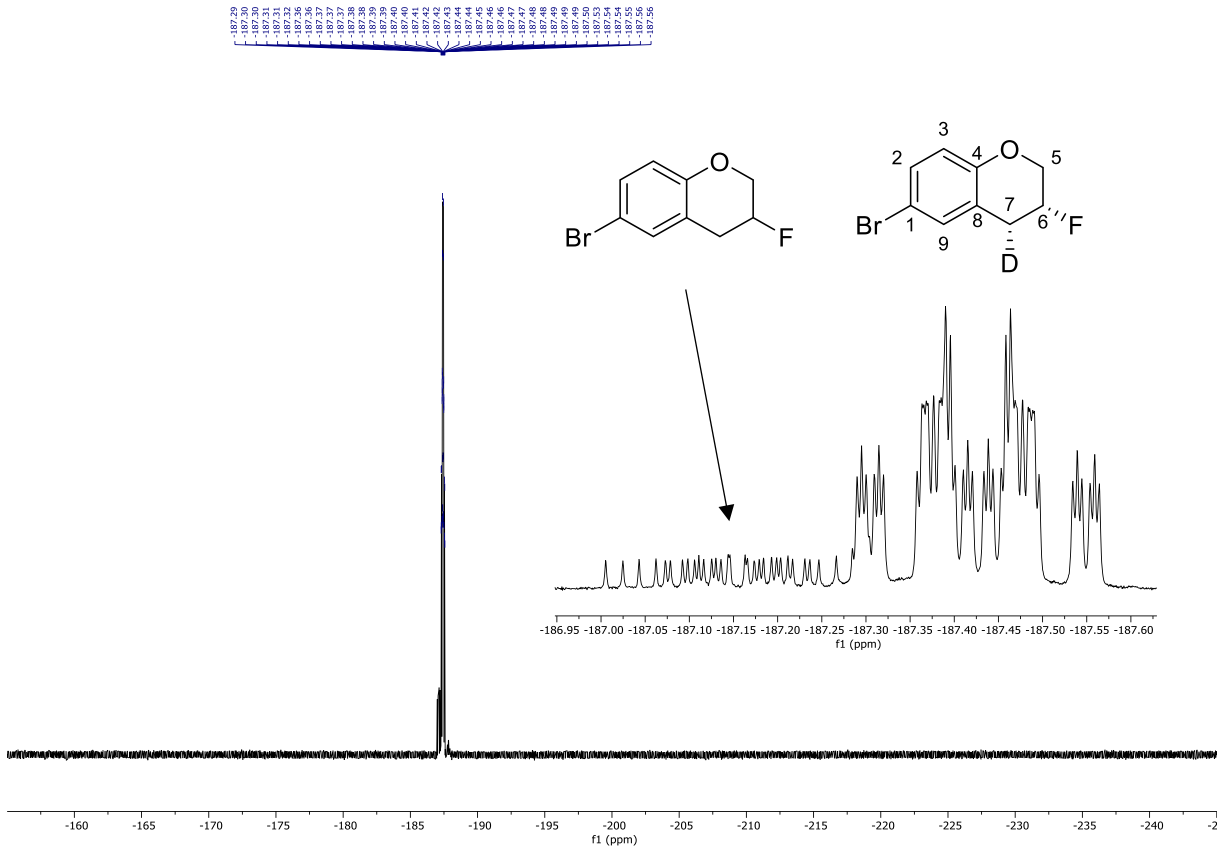

(3*S*,4*S*)-6-bromo-3-fluorochromane-4-*d* (*ent*-33)

^{1}H NMR (300 MHz, $CDCl_3$):

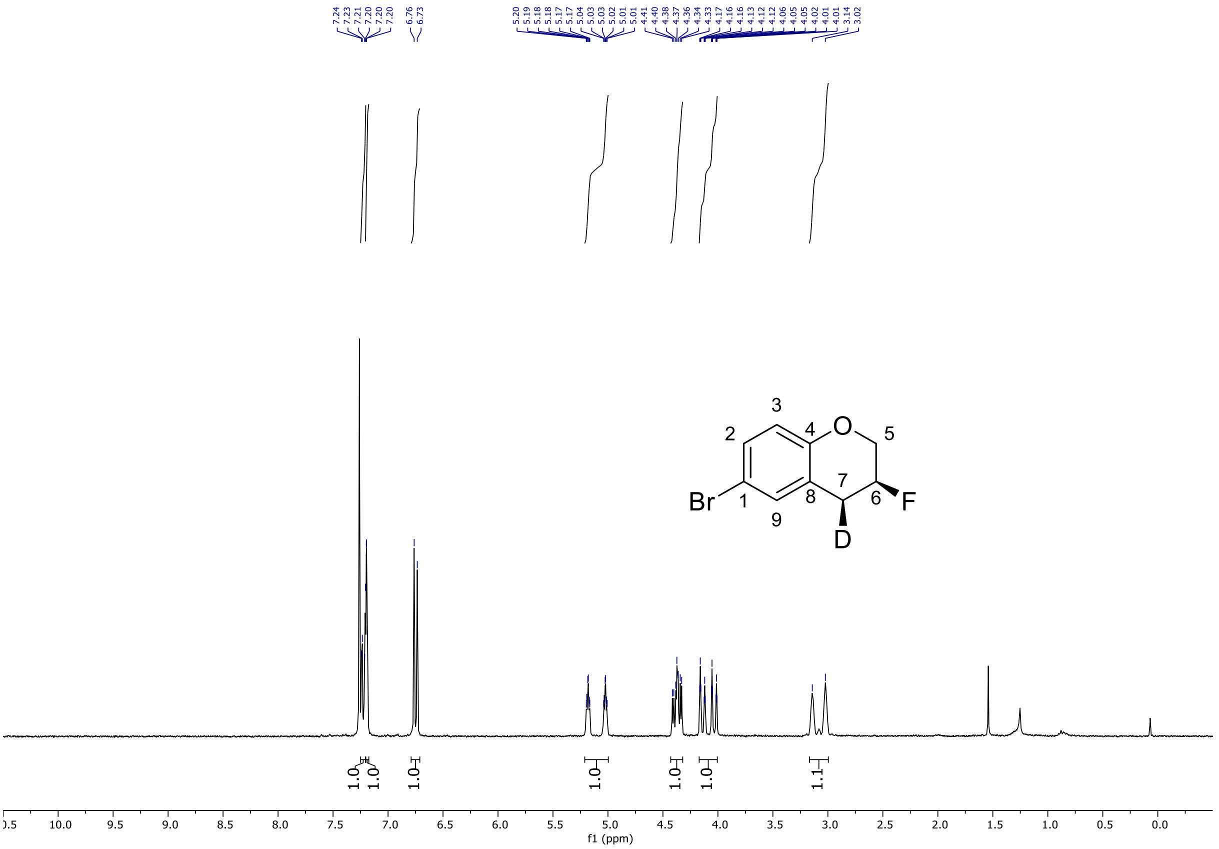

^{19}F{^{1}H} NMR (282 MHz, $CDCl_3$):

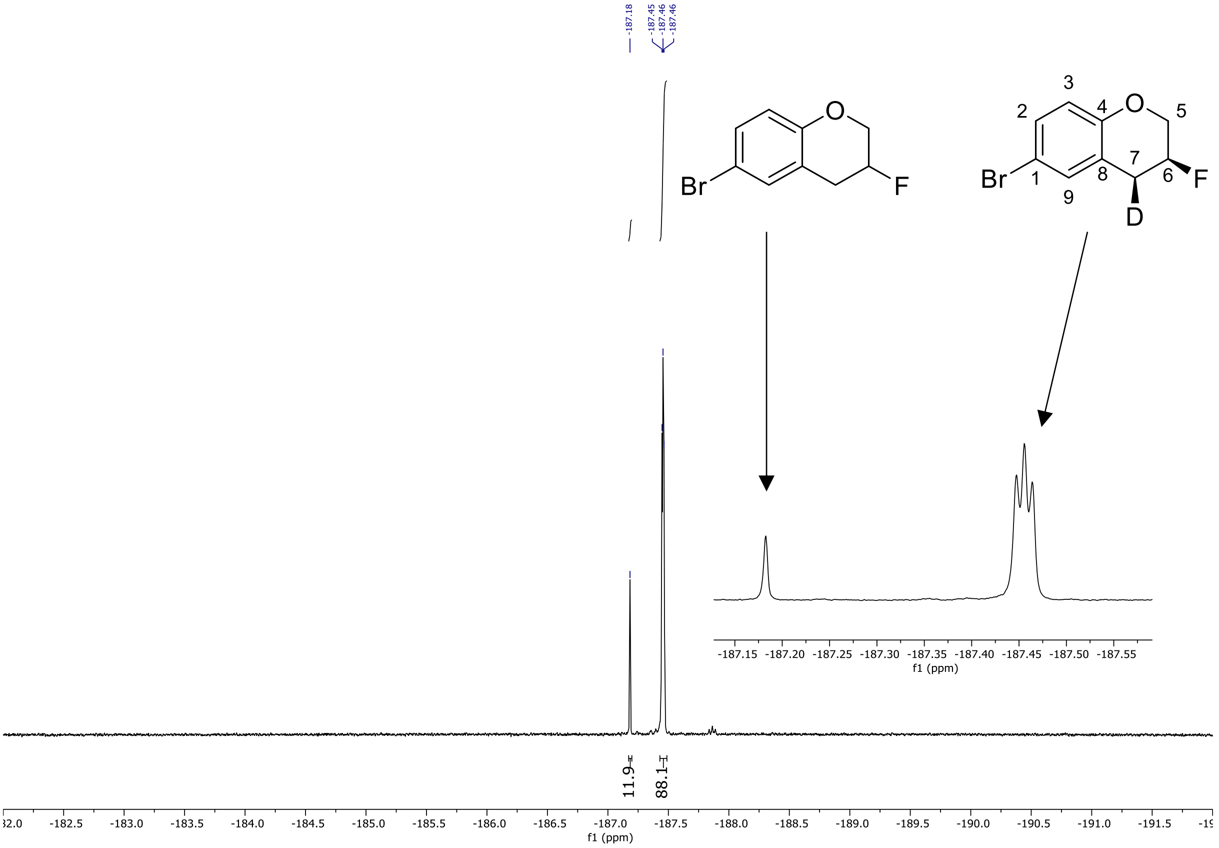

^{19}F NMR (282 MHz, $CDCl_3$):

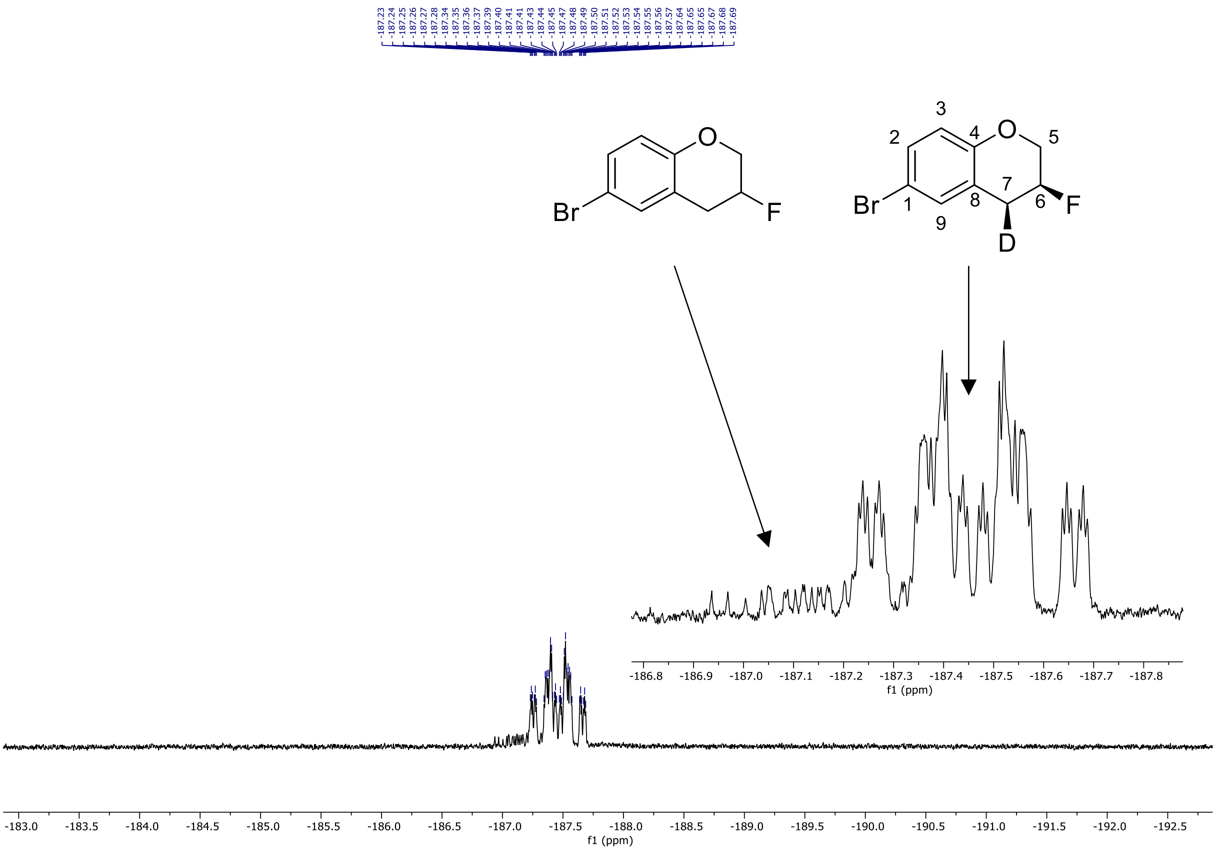

***N*-(4-Bromophenyl)-*N*-(2,3-difluoropropyl)-4-methylbenzenesulfonamide (27)**

^{1}H NMR (599 MHz, $CDCl_3$):

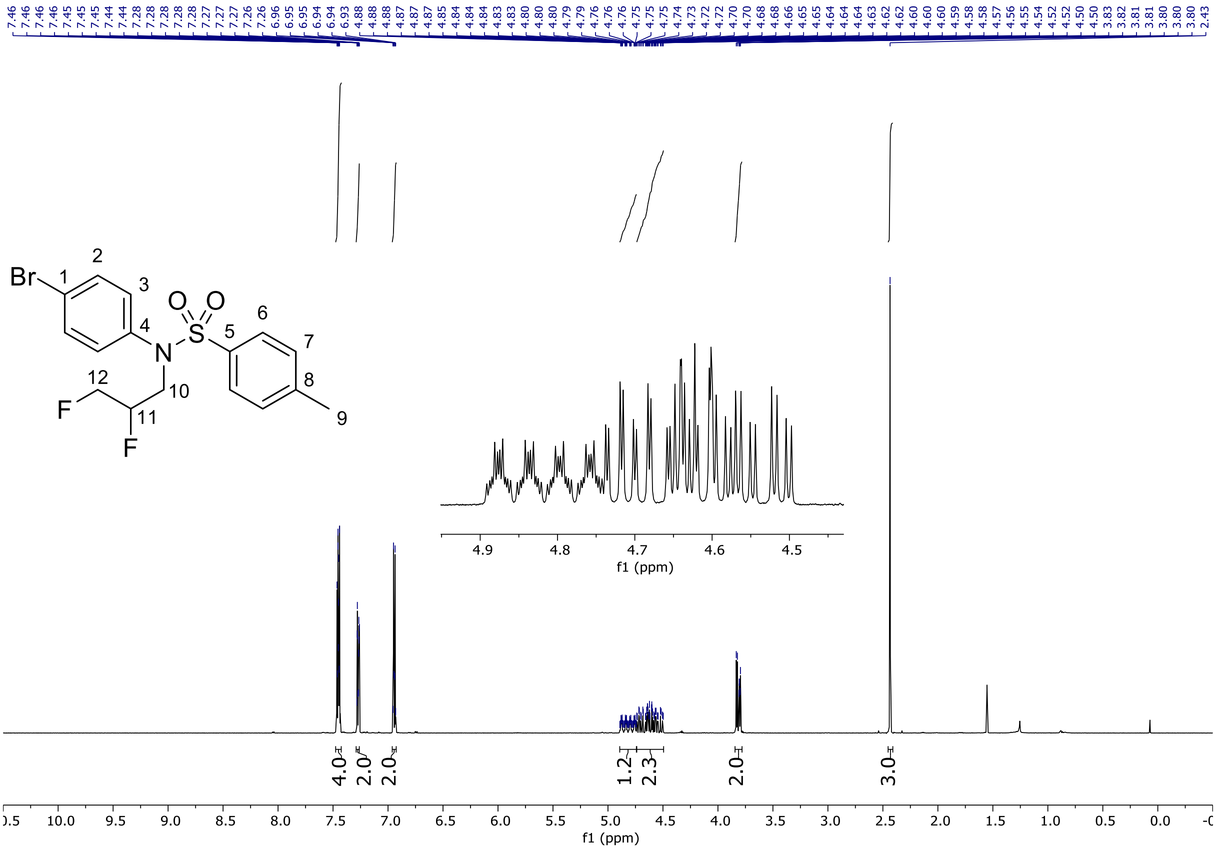

^{1}H{^{19}F} NMR (599 MHz, $CDCl_3$):

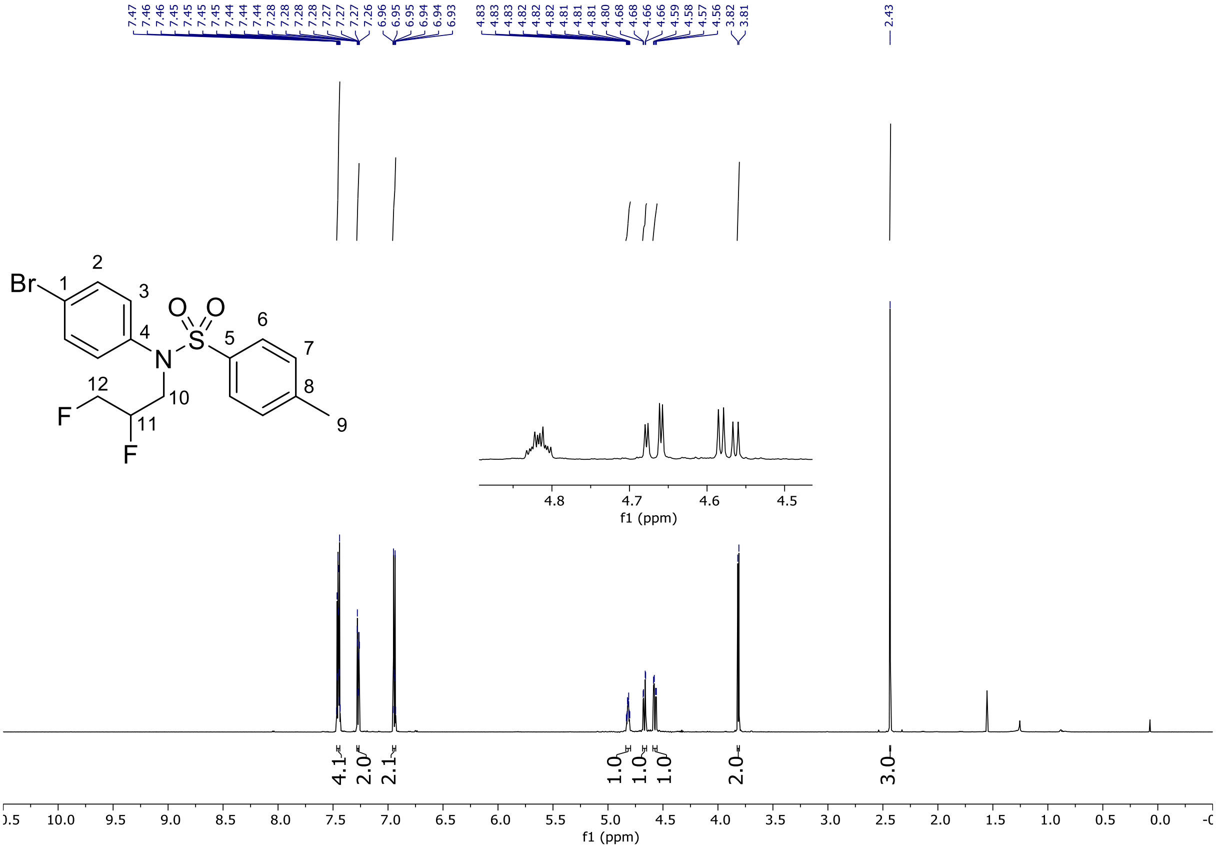

^{13}C{^{1}H} NMR (151 MHz, $CDCl_3$):

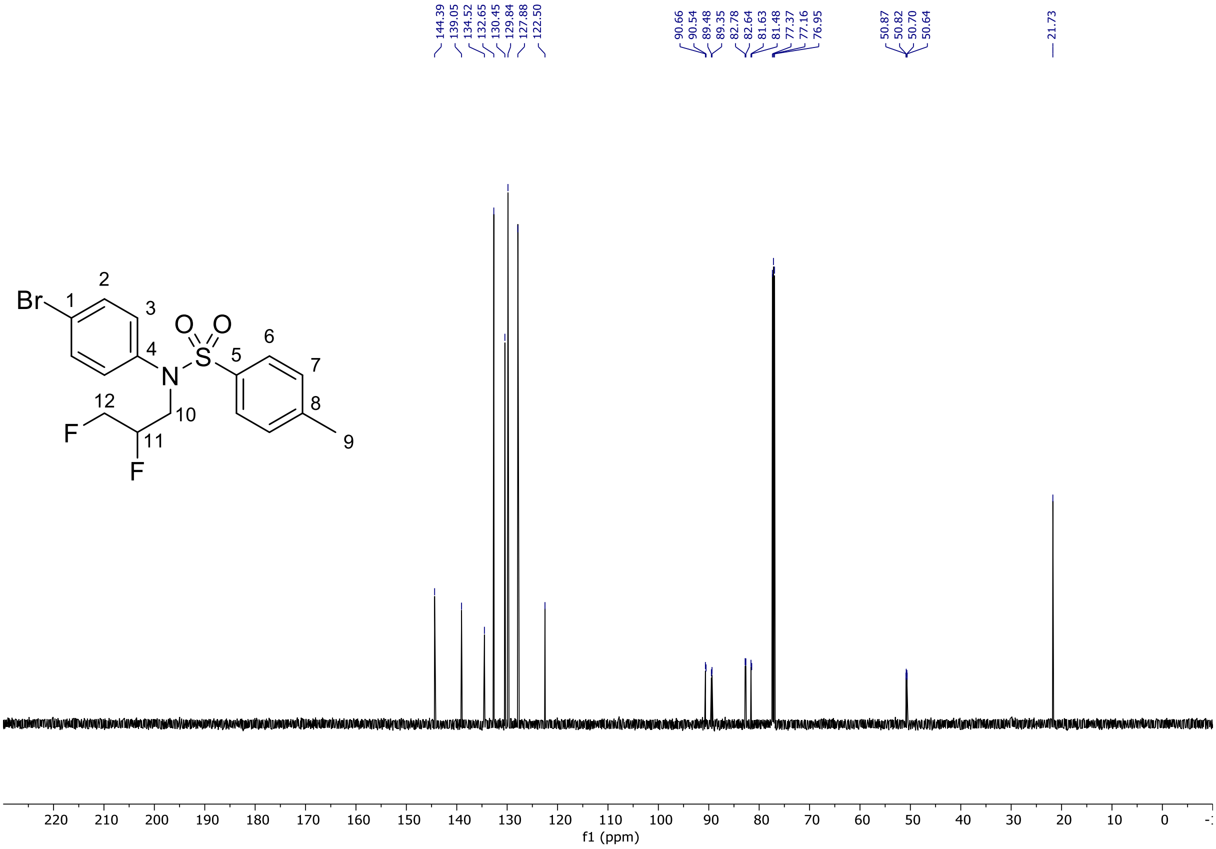

$^{13}C\{^{1}H\}\{^{19}F\}$ NMR (151 MHz, $CDCl_3$):

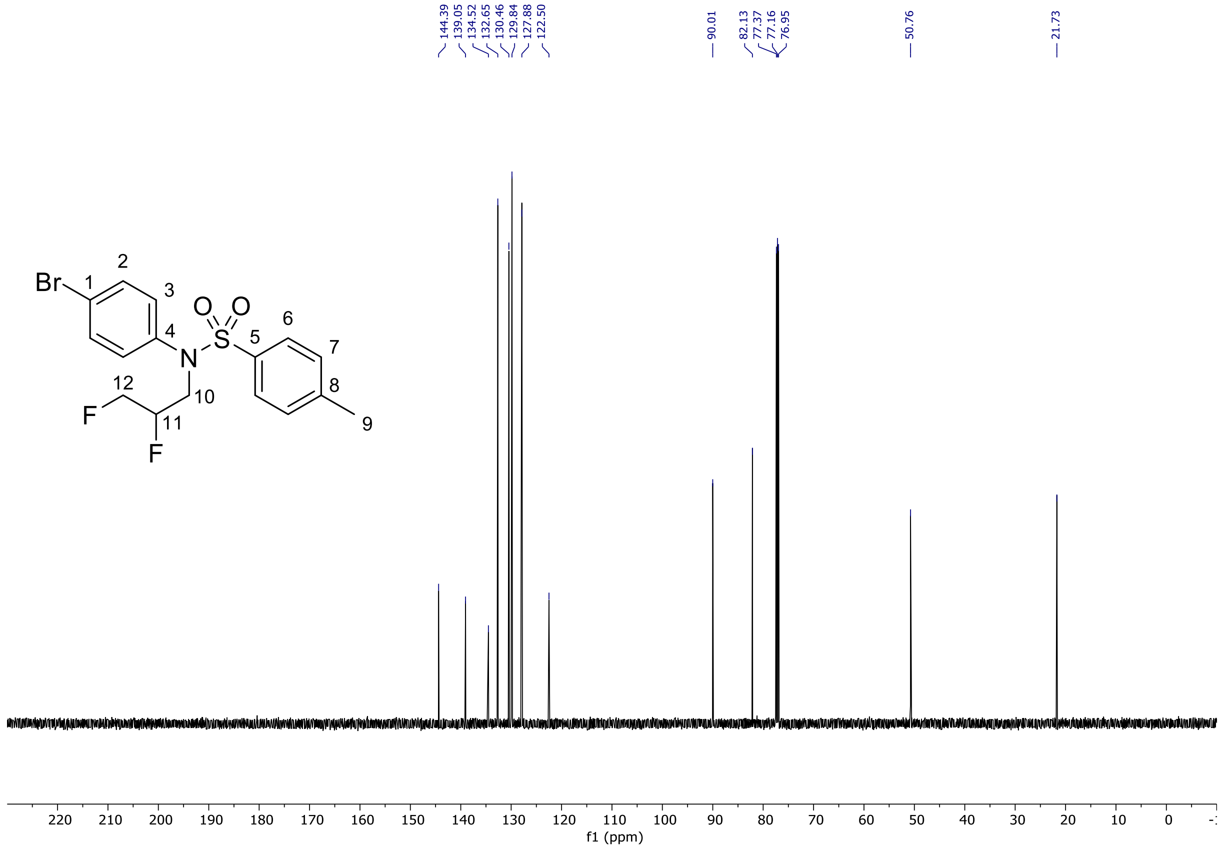

^{19}F NMR (564 MHz, $CDCl_3$, top), **$^{19}F\{^{1}H\}$ NMR** (564 MHz, $CDCl_3$, bottom):

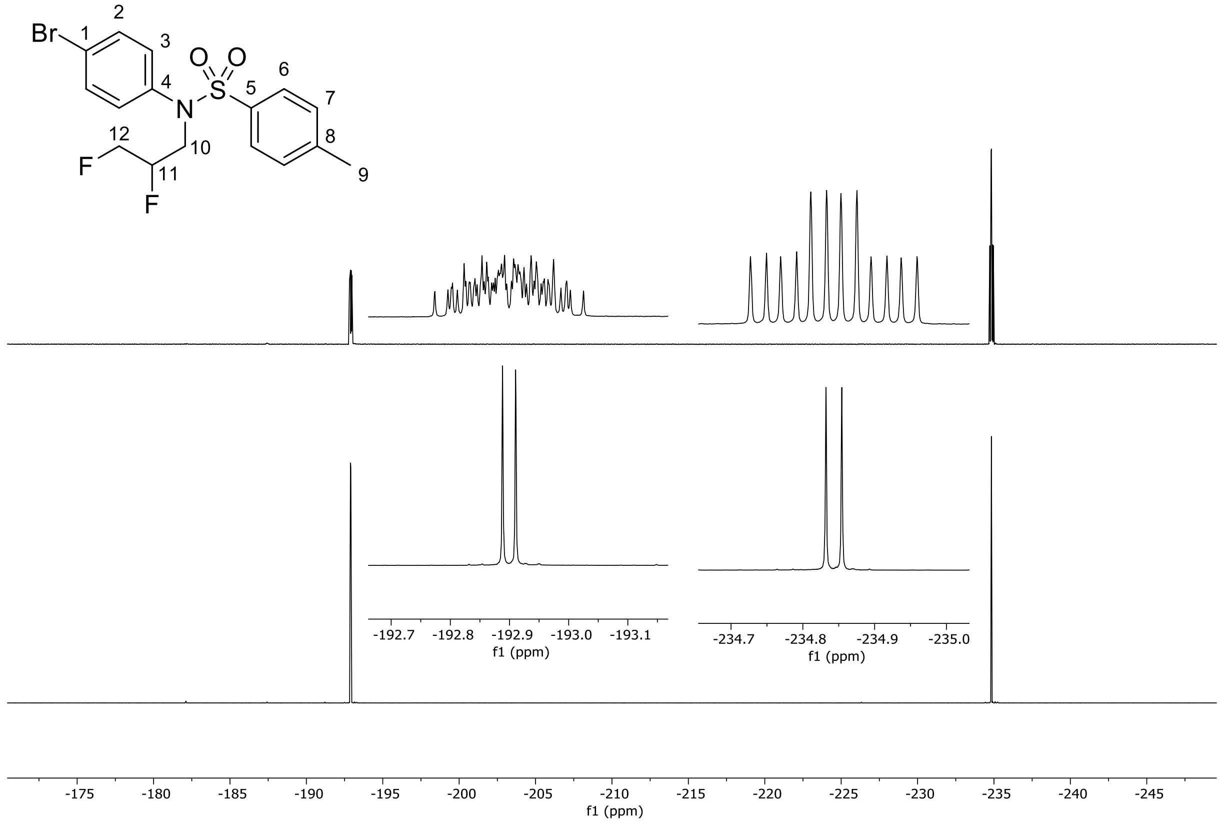

7. References

1. S. Haubenreisser, T. H. Wöste, C. Martínez, K. Ishihara, K. Muñiz, *Angew. Chem. Int. Ed.* **2016**, *55*, 413-417.
2. S.-I. Tsujiyama, K. Suzuki, *Org. Synth.* **2007**, *84*, 272-284.
3. M. A. Berliner, E. M. Cordi, J. R. Dunetz, K. E. Price, *Org. Process Res. Dev*. **2010**, *14*, 180–187.
4. J. Müller, S. C. Feifel, T. Schmiederer, R. Zocher, R. D. Süssmuth, *ChemBioChem* **2009**, *10*, 323-328.
5. S. Porto, J. M. Seco, A. Ortiz, E. Quiñoá, R. Riguera, *Org. Lett.* **2007**, *9*, 5015-5018.
6. T. Hoegberg, P. Stroem, M. Ebner, S. Raemsby, *J. Org. Chem.* **1987**, *52*, 2033-2036.
7. M. Fujita, Y. Yoshida, K. Miyata, A. Wakisaka, T. Sugimura, *Angew. Chem. Int. Ed.* **2010**, *49*, 7068-7071.
8. R. T. Sawant, S. B. Waghmode, *Tetrahedron* **2010**, *66*, 2010-2014.
9. N. K. Jobson, A. R. Crawford, D. Dewar, S. L. Pimlott, A. Sutherland, *Bioorg. Med. Chem. Lett.* **2009**, *19*, 4996-4998.
10. L. Peng, Z. Li, G. Yin, *Org. Lett.* **2018**, *20*, 1880-1883.
11. F. Scheidt, M. Schäfer, J. C. Sarie, C. G. Daniliuc, J. J. Molloy, R. Gilmour, *Angew. Chem. Int. Ed*. **2018**, *57*, 16431-16435.
12. I. A. Sayyed, V. V. Thakur, M. D. Nikaljc, G. K. Dewkar, S.P. Kotkar, A. Sudalai, *Tetrahedron* **2005**, *61*, 2831-2838.
13. R. Bujok, M. Bieniek, M. Masnyk, A. Michrowska, A. Sarosiek, H. Stepowska, D. Arlt, K. Grela, *J. Org. Chem.* **2004**, *69*, 6894-6896.
14. A. K. El-Qisairi, H. A. Qaseer, P. M. Henry, *J. Organomet. Chem.* **2002**, *656*, 168-176.
15. I. Triandafillidi, M. G. Kokotou, C. G. Kokotos, *Org. Lett.* **2018**, *20*, 36-39.
16. K. Kaneko, Y. Miwa, N. Nakamura, *J. Appl. Polym. Sci.* **2007**, *105*, 2472-2481.
17. H. Murakami, T. Minami, F. Ozawa, *J. Org. Chem.* **2004**, *69*, 4482-4486.
18. J. Wen, D. Chennamadhavuni, S. R. Morel, M. K. Hadden, *ACS Med. Chem. Lett.* **2019**, *10*, 1290-1295.
19. R. F. D´Vries, C. D. Grande, M. N. Chaur, J. A. Ellena, R. C. Advincula, *Acta Crystallogr Sect E Struct Rep Online*. **2014**, *70(Pt 7)*, o814–o815.
20. H. Rueeger, R. Lueoend, O. Rogel, J.-M. Rondeau, H. Möbitz, R. Machauer, L. Jacobson, M. Staufenbiel, S. Desrayaud, U. Neumann *J. Med. Chem.* **2012**, *55*, 3364-3386.
21. A. L. Pincock, J. A. Pincock, R. Stefanova, *J. Am. Chem. Soc.* **2002**, *124*, 9768-9778.
22. H. Murakami, T. Minami, F. Ozawa, *J. Org. Chem*. **2004**, *69*, 4482-4486.
23. W. Gu, R. B. Silverman, *Org. Lett*. **2003**, *5*, 415-418.
24. A. P. Combs, T. P. Maduskuie Jr., N. Falahatpisheh, *WO 2015/164480 A1*, **2015**.
25. Q. T. Do, D. Elothmani, J. Simonet, G. Le Guillanton, *Electrochim. Acta* **2005**, *50*, 4792-4799.
26. K. Jeyakumar, R. D. Chakravarthy, D. K. Chand, *Catal. Commun.* **2009**, *10*, 1948-1951.
27. A. Kar, I. A. Sayyed, W. F. Lo, H.M. Kaiser, M. Beller, M. K. Tse, *Org. Lett.* **2007**, *9*, 3405-3408.

28. L. M. Betts, N. C. Tam, S. M. H. Kabir, R. F. Langler, I. Crandall, *Aust. J. Chem.* **2006**, *59*, 277-282.
29. H. Huang, J. Denne, C.-H. Yang, H. Wang, J. Y. Kang, *Angew. Chem. Int. Ed.* **2018**, *57*, 6624-6628.
30. X. Lei, A, Jalla, M. A. A. Shama, J. M. Stafford, B. Cao, *Synthesis* **2015**, *47*, 2578-2585.
31. F.-N. Li, N.-J. Kim, S.-M. Paek, D.-Y. Kwon, K. H. Min, Y.-S. Jeong, S.-Y. Kim, Y.-H. Park, H.-D. Kim, H.-G. Park, Y.-G. Suh, *Bioorg. Med. Chem.* **2009**, *17*, 3557-3567.
32. P. W. Davies, S. J.-C. Albrecht, *Chem. Commun*. **2008**, 238-240.
33. B. Métayer, A. Mingot, D. Vullo, C. T. Supuran, S. Thibaudeau, *Chem. Commun.* **2013**, *49*, 6015-6017.
34. W. Chen, X.-D. Yang, Y. Li, L.-J. Yang, X.-Q. Wang, G.-L. Zhang, H.-B. Zhang, *Org. Biomol. Chem.* **2011**, *9*, 4250-4255.
35. L. Alonso-Marañón, M. Monserrat Martínez, L. A. Sarandeses, J. Pérez Sestelo, *Org. Biomol. Chem.* **2015**, *13*, 379-387.
36. B. H. Lipshutz, R. Keil, E. L. Elsworth, *Tetrahedron Lett*. **1990**, *31*, 7257-7260.
37. I. G. Mólnar, R. Gilmour, *J. Am. Chem. Soc*. **2016**, *138*, 5004-5007.
